# ISGylation-mediated stabilization of CYP4Z1 fuels breast cancer initiation and progression

**DOI:** 10.1038/s12276-026-01766-4

**Published:** 2026-07-02

**Authors:** Yin Yuan, Yu Lu, Xuedan Han, Yidong Zhang, Qianqian Guo, Hong Yao, Qiyi Yu, Manzhen Zhou, Xinting Huang, Hongqi Wang, Miaomiao Niu, Jun Yin, Haibo Rong, Lei Huang, Xiaoman Li, Shengtao Xu, Lufeng Zheng

**Affiliations:** 1 School of Life Science and Technology, Department of Medicinal Chemistry, School of Pharmacy, China Pharmaceutical University, Nanjing, Jiangsu Province China; 2 Department of Biological Sciences, Virginia Tech, Blacksburg, VA USA; 3 Department of Pharmacy, The Affiliated Cancer Hospital of Zhengzhou University & Henan Cancer Hospital, Zhengzhou, P. R. China; 4 Mellon College of Science, Carnegie Mellon University, Pittsburgh, PA USA; 5 Jiangsu Cancer Hospital & Jiangsu Institute of Cancer Research & The Affiliated Cancer Hospital of Nanjing Medical University, Nanjing, Jiangsu Province China; 6 Department of Molecular Cell and Cancer Biology, University of Massachusetts Chan Medical School, Worcester, MA 01605 USA; 7 Jiangsu Key Laboratory for Pharmacology and Safety Evaluation of Chinese Materia Medica, School of Pharmacy, Nanjing University of Chinese Medicine, Nanjing, Jiangsu Province China

**Keywords:** Breast cancer, Molecular biology, Drug discovery

## Abstract

Tumor-initiating cells (TICs) are central to cancer progression and metastasis; however, they remain challenging to target therapeutically. Here, using a breast-specific CYP4Z1-transgenic MMTV-PyMT mouse model, we show that CYP4Z1 notably accelerates tumor initiation, enlarges tumor size, promotes metastasis, and expands TIC populations. Multi-omics analyses reveal that CYP4Z1^+^ epithelial cells exhibit enhanced stemness and reduced differentiation compared with CYP4Z1^−^ counterparts. Notably, ISG15-mediated ISGylation of CYP4Z1 at lysine residues K259, K279, and K502 stabilizes the protein, augments its enzymatic function, promotes endoplasmic reticulum localization, and prevents ubiquitination, thereby amplifying TIC-like properties. This modification further upregulates triglyceride synthesis and promotes lipid droplet formation. Finally, we develop BM-51, a specific CYP4Z1 inhibitor that attenuates TIC-like traits and enhances chemotherapy efficacy. Our findings highlight the therapeutic potential of targeting CYP4Z1 and its ISGylation to disrupt TIC-driven breast cancer progression.

**Figure:**
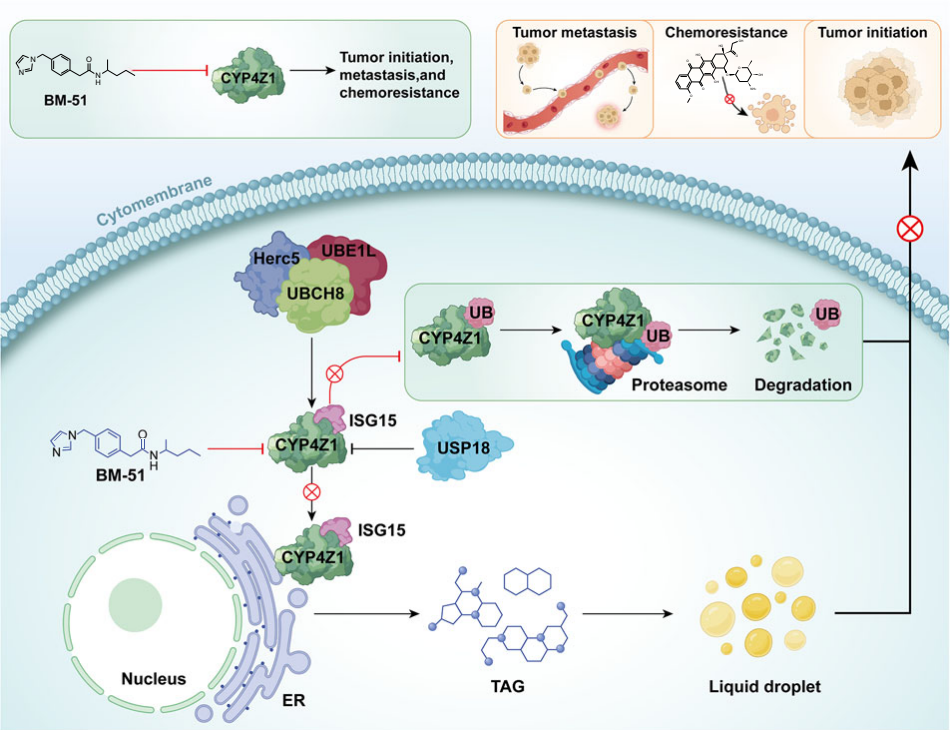
The ISGylation of CYP4Z1 is controlled by enzymes associated with ISGylation, including E1 enzyme UBE1L, E2 enzyme UBCH8, E3 enzyme Herc5, and the de-ISGylation enzyme USP18. The ISGylation of CYP4Z1 enhances the tumorigenic properties of CYP4Z1 in two key ways. First, the ISGylation of CYP4Z1 inhibits its ubiquitination and degradation, resulting in elevated CYP4Z1 protein levels. Second, the ISGylation of CYP4Z1 facilitates the translocation of CYP4Z1 to the endoplasmic reticulum (ER), which promotes downstream triglyceride synthesis and lipid droplet formation. These processes contribute to tumor initiation, metastasis, and chemoresistance. The inhibitor BM-51, designed explicitly for CYP4Z1, can counteract the tumor-promoting effects of CYP4Z1 by suppressing its enzymatic activity. TAG, triglyceride; UB, ubiquitin.

The ISGylation of CYP4Z1 is controlled by enzymes associated with ISGylation, including E1 enzyme UBE1L, E2 enzyme UBCH8, E3 enzyme Herc5, and the de-ISGylation enzyme USP18. The ISGylation of CYP4Z1 enhances the tumorigenic properties of CYP4Z1 in two key ways. First, the ISGylation of CYP4Z1 inhibits its ubiquitination and degradation, resulting in elevated CYP4Z1 protein levels. Second, the ISGylation of CYP4Z1 facilitates the translocation of CYP4Z1 to the endoplasmic reticulum (ER), which promotes downstream triglyceride synthesis and lipid droplet formation. These processes contribute to tumor initiation, metastasis, and chemoresistance. The inhibitor BM-51, designed explicitly for CYP4Z1, can counteract the tumor-promoting effects of CYP4Z1 by suppressing its enzymatic activity. TAG, triglyceride; UB, ubiquitin.

## New inhibitor BM-51 reduces tumor growth and metastasis

Breast cancer has become the most prevalent cancer worldwide, surpassing lung cancer, and is increasingly affecting younger individuals. Despite advancements in treatment, resistance remains a significant challenge. This study investigates the role of CYP4Z1, a cytochrome P450 enzyme, in breast cancer progression, using a transgenic mouse model expressing human CYP4Z1. Researchers identified that ISG15, a ubiquitin-like protein, mediates the ISGylation of CYP4Z1, enhancing its enzymatic activity and promoting tumor-initiating cell traits. The study further developed BM-51, a novel inhibitor targeting CYP4Z1, which demonstrated potent inhibitory activity with an IC_50_ of 91.7 nM and favorable pharmacokinetics. BM-51 effectively reduced tumor-initiating cell-like traits and tumor progression in vivo. These findings highlight the role of CYP4Z1 in breast cancer and suggest BM-51 as a promising therapeutic agent, with future research needed to explore its clinical applications.

This summary was initially drafted using artificial intelligence, then revised and fact-checked by the author.

## Introduction

The incidence of breast cancer now surpasses that of lung cancer, making it the most prevalent cancer globally, with a concerning trend toward affecting younger individuals^1^. Although notable progress has been made in the treatment of breast cancer, a considerable portion of patients still face primary or acquired treatment resistance^2^. It has been indicated that the acquisition of malignant traits by tumor cells is facilitated through the activation of developmental signaling pathways, such as the classical stem cell signals Oct4, Hedgehog, Wnt, and Notch^3^. These signaling pathways are typically upregulated in cancer cells, driving tumor self-renewal and proliferation. Functionally, stem cell signals are often enriched in tumor-initiating cells (TICs), also known as cancer stem cells (CSCs), which are closely associated with chemotherapy resistance and metastasis^4^. Since Clarke and co-workers^5^ first confirmed the existence of breast TICs in 2003, research into these cells has gradually become a focus and various methods have been proposed, such as targeting the CD44^+^/CD24^−^ stem cell population, targeting signaling pathways of breast TICs, antibody therapy based on surface-specific protein molecules of breast TICs, and gene silencing therapy^6^. Nonetheless, the effectiveness of these methods is still limited owing to the lack of identification of vulnerabilities in breast TICs, with potential off-target adverse reactions. Therefore, there is a pressing need to pinpoint essential driver genes responsible for promoting the development of breast TICs and to develop novel drugs capable of targeting these newly identified vulnerabilities with heightened specificity.

CYP4Z1 is a member of the human cytochrome p450 (CYP) enzyme superfamily^7^. Although ours and other studies have indicated the promoting effects of CYP4Z1 on breast cancer progression^8–13^, its roles have never been validated through transgenic breast cancer models and the underlying mechanisms by which *CYP4Z1* functions are still confusing. Here, as CYP4Z1 did not express in mice, we constructed a transgenic mouse model that specifically expresses human *CYP4Z1* in breast tissue of mouse model (*CYP4Z1*^*+/+*^) and crossed with the *MMTV-PyMT* mouse model to generate female cohorts with the genotypes of *MMTV-PyMT*; *CYP4Z1*^*+/+*^ (*PyMT-CYP4Z1*), aiming to validate the roles of *CYP4Z1* in breast cancer progression thoroughly.

ISG15 (interferon-stimulated gene 15) is also known as a ubiquitin-like protein, and ISG15-mediated ubiquitin-like modification (ISGylation) is similar to ubiquitination^14^. The effects triggered by ISGylation vary depending on cell type, physiological status, substrate, and subcellular localization^15^. Studies by Sainz et al.^16,17^ confirmed that ISGylation is necessary for the stemness and plasticity of pancreatic TICs. Subsequent studies have also demonstrated that ISGylation can promote the TIC-like traits of other tumor cells^18–20^. To reveal the underlying mechanisms by which *CYP4Z1* functions during breast cancer progression, we used co-immunoprecipitation (co-IP) combined with interactome proteomics to screen and identify proteins that interact with CYP4Z1. Among the interacting proteins capable of mediating post-translational modifications (PTMs), the ubiquitin-like protein ISG15 showed the highest interaction score with CYP4Z1. These results encourage us to investigate whether ISG15 can mediate the ISGylation of CYP4Z1 and the roles of ISGylation in effects of CYP4Z1 on breast cancer progression.

Currently, we used the *PyMT-CYP4Z1* mice model combined with multi-omics analysis to reveal the underlying mechanisms by which CYP4Z1 functions during breast cancer progression. Furthermore, to develop potent and selective CYP4Z1 inhibitors, a series of novel 1-benzylimidazole (1-Ben) analogs were rationally designed and synthesized from lead compound 1-Ben, among which a promising compound BM-51 was identified. BM-51 exhibited potent inhibitory activity against CYP4Z1 with an IC_50_ value of 91.7 nM and attenuated breast TICs by targeting amino acid residues Arg384, Ser383, Val126, and Ala314 of CYP4Z1. Importantly, BM-51 held a high selectivity against CYP4 isozymes (>100 folds). Additionally, it was found that BM-51 exhibited favorable pharmacokinetic properties which guarantees that it acts as a pharmacological agent for in vivo studies. This study provides compelling evidence that the ISGylation of CYP4Z1 could facilitate the progression of breast TICs, and BM-51 might be a potential drug for suppressing breast TICs through targeting CYP4Z1.

## Materials and methods

### Transgenic mouse strains and tumor monitoring

All animal experiments were performed in strict accordance with the protocols approved by the Animal Welfare and Ethics Committee of China Pharmaceutical University (Approval No. 2021-10-017). The construction of transgenic mice with targeted expression of *CYP4Z1* transgene in the breast tissue and the *MMTV-PyMT* (stock no. 022974) transgenic mice were obtained from Shanghai Nanfang Model Biotechnology Co., Ltd. All mice were on a mixed C57BL/6 background with littermate controls (females) used in all experiments. For spontaneous tumorigenesis studies, weaned females were examined by palpation twice weekly for breast tumor development. After 3 weeks of initial detection of palpable tumors, lungs were collected to determine metastatic nodules. Investigators were not blinded to assignments during the experiment and outcome assessment. No randomization method was used, in which mice were segregated into groups based on genotype only. No statistical methods were used to predetermine sample size. All animals were used following the procedures approved by the Shanghai Nanfang Model Biotechnology Co., Ltd and Use Committee (Licence No. 2019-0026). The mice were kept as follows: *n* = 3 per cage for antitumor study in tumor xenograft mice. They were housed under conventional laboratory conditions at a room temperature maintained at 25 ± 1 °C with a relative humidity range of 40–75% and a regular 12 h light/12 h dark cycle. The mice were fed a standard animal pellet diet and allowed free access to water. At the end of the experiment, the mice were killed by an overdose injection of pentobarbitone (200 mg/kg).

### Isolation of Lin-NECs (neoplastic MECs (mammary epithelial cells)) and pNECs (pre-neoplastic MECs)

The pre-neoplastic glands or primary tumors were dissected into pieces, in which fat was removed. The organoids were enzymatically dissociated at 37 °C for 1 h in lysis solution (DMEM/F12 (Keygen BioTECH, Nanjing, China)) containing 5% fetal bovine serum (OmnimAbs, Shanghai, China), 10 ng/ml epidermal growth factor (Sigma-Aldrich, St Louis, MO, USA), 500 ng/ml hydrocortisone (Sigma-Aldrich), 5 μg/ml insulin (Sigma-Aldrich), and 1% penicillin/streptomycin (Sangon Biotech, Shanghai, China)) supplemented with collagenase/hyaluronidase (Sigma-Aldrich). In addition, the sediment was harvested and incubated with trypsin-EDTA (0.25%) for 2 min, 5 mg/ml Dispase (Thermo Fisher Scientific, Waltham, MA, USA) plus 0.1 mg/ml DNase (Sigma-Aldrich) for 5 min, and 0.64% NH_4_Cl for 5 min at 37 °C. Cells were filtered with a 40-µm cell strainer and re-suspended in Hank’s balanced salt solution buffer containing 0.5% bovine serum albumin. Finally, NEC and pNECs were enriched using the EasySep™ Mouse Epithelial Cell Enrichment Kit II (Catalog no. 19868, StemCell Technologies, Vancouver, British Columbia, Canada).

### Cell culture, transfection, and lentiviral-based gene transduction

Human breast cancer cell lines, including luminal A-type (MCF-7, T47D), basal-like type (MDA-MB-231), human embryonic kidney cells HEK-293T, and mice breast cancer cells 4T1 were purchased from ATCC (Manassas, VA, USA) and archived in our laboratory repository. These cell lines were maintained in DMEM (Keygen BioTECH) supplemented with 10% fetal bovine serum at 37 °C in 5% CO_2_ (v/v). Jet-PRIME (Polyplus Transfection, Illkirch, France) was used for transfection. The lentivirus of CYP4Z1-wt (wild-type) and CYP4Z1^K259R/K279R/K502R^ overexpression was purchased from Corues Biotechnology (Nanjing, China). The final viral titer was 1 × 10^9^ TU/ml. Cells were infected with the lentivirus and selected by puromycin (Sigma-Aldrich, 2 μg/ml).

### Data retrieving

Single-cell dataset GSE225600 was downloaded from the Gene Expression Omnibus database^21^. The dataset encompassed gene expression profiles from a total of 81,683 cells across 4 primary breast tumor samples and 4 paired lymph metastasis samples. Bulk-seq data of patients with breast cancer were acquired from The Cancer Genome Atlas Genomic Data Commons (TCGA GDC) via UCSC Xena (https://xenabrowser.net/, GDC TCGA Breast Cancer)^22^. This gene expression dataset included count data for 60,488 genes across 1,217 samples. Additionally, survival data for 1,260 breast cancer samples and clinical data for 1,248 breast cancer samples were obtained from the same source. The spatial transcriptomic dataset GSE213688, which contains three putative Claudin-low tumors, four genomically unstable triple-negative breast cancer samples, and four metaplastic breast cancer samples, was adopted for spatial transcriptomic analysis^23^.

### Cell clustering and annotation

Single-cell RNA (scRNA) data were processed by “Seurat” package (version 4.3.0)^24^. Briefly, the quality control was performed with parameters set as: 400 < nFeature_RNA < 4,000 and pctMT ≤ 10. Normalization was conducted by the LogNormalize function with default settings, followed by an algorithm named Harmony to integrate all samples and minimize the batch effect. Principal component analysis (PCA) was then carried out for dimensional reduction. Then, the annotation was manually completed based on the conventional markers as reported previously^25^ (Supplementary Table 1).

### Epithelial extraction and re-clustering

Epithelial cells were extracted from annotated scRNA data for dimensional reduction and unsupervised clustering. According to the expression of *CYP4Z1* in different epithelial clusters, the *CYP4Z1*^*+*^ Epi and *CYP4Z1*^−^ Epi cell clusters were annotated manually. Subsequently, via the FindMarkers function, the differential expressed genes between *CYP4Z1*^*+*^ Epi and *CYP4Z1*^−^ Epi cell clusters were screened, and the top 20 significant differential expressed genes were selected as the characteristic genes of *CYP4Z1*^*+*^ Epi.

### Bulk-seq analysis

Patients with breast cancer were separated into high and low groups based on the median value of *CYP4Z1* expression. Then, the Gene Set Variation Analysis (GSVA, version 1.46.0) was conducted to evaluate the enrichment of *CYP4Z1*^*+*^ Epi-related genes in patients with breast cancer^26^. Alternatively, to assess the tumor purity of patients in high and low groups, the “estimate” package was used^27^. Through R package “corrplot” (version 0.92), the Pearson correlations among StromalScore, ImmuneScore, ESTIMATEScore, tumor purity, and GSVA score were calculated.

### Cell stemness analysis

After merging the scRNA data without epithelial cell clusters and new annotated epithelial clusters, 5000 random cells were sampled for further single-cell-level analysis, including cell stemness, pseudotime, and cell–cell communication analysis. To explore the cell stemness of different cell clusters, the “CytoTRACE” package (version 0.3.3) was adopted^28^. CytoTRACE provides an approach to infer the developmental potential of cell clusters independently and robustly. CytoTRACE score reflects the stemness of each selected cell, respectively, which ranged from 0 to 1. The higher the score, the higher the stemness of the cell.

### Pseudo-time analysis and intercellular communication evaluation

The pseudotime analysis was performed via the “monocle” package (version 2.26.0)^29^. Quality control criteria were set as expressions > 0.1 and dispersion empirical > 1 * dispersion fit cells, and then the cell differentiation states of cell clusters were identified. The expression of *CYP4Z1*^*+*^ Epi cell characteristic genes in different cell states was also visualized. Besides, we used the “Cellchat” package (version 1.6.1) to infer the probability of cell–cell communications between specific types of cells in samples, based on prior knowledge of communications between signaling ligand–receptor pairs and cofactors^30^.

### Spatial transcriptomic analysis

Samples in spatial transcriptomic dataset GSE213688 were used to perform spatial transcriptomic analysis^23^. In Seurat package, the quality control was conducted by removing mitochondrial genes and genes expressed in less than 10 spots. Subsequently, the normalization was performed via “SCTransform” function. Further dimensionality reduction and clustering were performed with PCA and uniform manifold approximation and projection methods. On the basis of canonical markers of epithelial cells (EPCAM, CDH1, KRT7, and KRT19) and characteristic genes of *CYP4Z1*^*+*^ Epi cells, the “AddModuleScore” function was applied to evaluate the enrichment of epithelial cell genes and *CYP4Z1*^*+*^ Epi characteristic genes, for estimation of fractions of these two types of cells in spatial spots.

For precise evaluation of the cell proportion of spatial spots in samples, the “SPOTlight” package was used^31^. The deconvolution process was used to match the annotated breast cancer tissue scRNA dataset GSE225600 and spatial transcriptomic data GSE213688. The proportions of *CYP4Z1*^*+*^ Epi cells and *CYP4Z1*^−^ Epi cells in each spatial spot were estimated and visualized thereafter.

### Bayes deconvolution of single-cell data

To further evaluate the cell composition of TCGA-BRCA bulk-seq samples, BayesPrism (https://github.com/Danko-Lab/BayesPrism) package was used. BayesPrism was modeled a prior from cell-type-specific expression profiles from scRNA-seq to jointly estimate the posterior distribution of cell-type composition and cell-type-specific gene expression from bulk RNA-seq expression of tumor or non-tumor samples. After obtaining the cell proportions of each sample, the overall survival of patients with different CYP4Z1^+^ epithelial cell proportions was analyzed using the “survival” package. Specifically, the CYP4Z1 ratio was adopted for survival analysis: ${CYP}4Z1\mathrm{ratio}=\mathrm{prop}({CYP}4Z1+\mathrm{epithelial}\,\mathrm{cell})\ast \exp ({CYP}4Z1)$.

### Plasmids and small interfering RNAs (siRNAs)

Plasmids, chemicals, and siRNAs used in this study were listed in Supplementary Tables 2–4. Plasmids expressing mutant proteins were generated using Mut Express II Fast Mutagenesis Kit V2 (Vazyme, Nanjing, China), following the manufacturer’s instructions. The detailed primer sequences for vector construction were indicated in Supplementary Table 5. Mutations were confirmed by Sanger sequencing. siRNAs were purchased from Invitrogen (Carlsbad, CA, USA).

### Whole-mount staining of mammary gland and histology

The fourth inguinal mammary glands were isolated and fixed in Carnoy’s solution (60% ethanol, 30% CHCl_3_, and 10% glacial acetic acid) for 2–4 h, followed by hydration and carmine red staining overnight. Stained glands were mounted after flattening and dehydration. For histology, 4% paraformaldehyde-fixed tissues were embedded in paraffin and sectioned, followed by hematoxylin and eosin (HE) staining.

### Flow cytometry

Single cells derived from cell lines or purified mouse primary cells were re-suspended and incubated for 30 min on ice with CD44-APC (BioLegend, Catalog no. 338806, Clone no. BJ18, San Diego, CA, USA), CD24-PE (BioLegend, Catalog no. 311106, Clone no. ML5), CD24-APC (BD Biosciences, Catalog no. 562349, Clone no. M1/69), CD29-PE (BD Biosciences, Catalog no. 562801, Clone no. HM β1-1). The aldehyde dehydrogenase (ALDH) activity of purified cells was measured using ALDEFLUOR™ Kit (StemCell Technologies, Catalog no. 01700), following manufacturer’s instructions. Spontaneous single cells were prepared from bone marrow for the hematopoietic stem cell analysis. All antibodies were purchased from BioLegend. The antibodies such as anti-Gr-1-FITC (108405), Ter119-FITC (116205), B220-FITC (103205), CD19-FITC (115505), IgM-FITC (406505), CD127-FITC (121105), and CD3e-FITC (100305) were used for lineage markers. Anti-Sca1-PE (108107), c-Kit-APC (105811), CD34-PE/Cyanine7 (119325) and CD16, and 32-Brilliant Violet 510™ (101333) antibodies were used for hematopoietic progenitor cell population analysis; anti-CD150-Brilliant Violet 421™ (115925) and CD48-PerCP/Cyanine5.5 (103421) antibodies were used for hematopoietic stem cell (HSC) population analysis. All tests were detected by flow cytometer (BD, USA), and FACS analysis was performed using FACS Canto, Diva software, and FlowJo.

### Mammosphere assay

Single cells were seeded into ultralow-attachment plates (Corning, NY, USA) with DMEM/F12 (Keygen BioTECH) with epidermal growth factor (200 ng/ml, MedChem Express, Monmouth Junction, NJ, USA), basic fibroblast growth factor (200 ng/ml, Sigma-Aldrich), and B27 (50×, abs9120, Absin, Shanghai, China). Mammospheres were photographed and counted 8 days later. Spheroids with a diameter greater than 50 μm are considered to be spheroids.

### Western blotting and immunohistochemistry (IHC) analysis

For western blotting analysis, lysates prepared using RIPA buffer (Beyotime, Beijing, China) were subjected to SDS–PAGE and transferred onto polyvinylidene fluoride membranes (Merck Millipore, Billerica, MA, USA). The membranes were blocked with 5% non-fat milk for 2 h. After overnight incubation with primary antibodies at 4 °C, the membranes were incubated with secondary antibodies for 40 min and then developed using the Tanon™ High-sig ECL Western Blotting Substrate (Tanon, Catalog no. 180-5001, Shanghai, China). Gray scale analysis of western blot bands was performed using ImageJ software. For IHC analysis, tissues were fixed in 4% paraformaldehyde for 48 h at room temperature and then embedded in paraffin. Paraffin-embedded sections were deparaffinized and rehydrated, followed by antigen retrieval. After incubation with primary and secondary antibodies, the slides were treated with diaminobenzidine (Dako, USA). Details of the primary antibodies used were listed in Supplementary Table 6. IHC image analysis was conducted using Image-Pro Plus software.

### Co-IP and proteomics assays

Cells were homogenized in RIPA buffer supplemented with phenylmethylsulfonyl fluoride (Beyotime), Phosphatase Inhibitor Cocktail (Beyotime), and E3876 (Sigma-Aldrich). Lysates were cleared by centrifugation at 13,000 rpm for 15 min at 4 °C, and the supernatant was collected for protein quantification using a BCA assay kit. To remove nonspecific proteins and reduce background, 100 μl of Protein A agarose beads was added to 1 ml of total protein and gently shaken at 4 °C for 10 min. After centrifugation at 4 °C, 14,000×*g* for 15 min, the supernatant was transferred to a new tube, and the Protein A beads were discarded. Next, a specific volume of primary antibody was added to 500 μl of total protein, and the antigen–antibody mixture was gently shaken overnight at 4 °C. The antigen–antibody complex was captured by adding 100 μl of Protein A agarose beads, followed by gentle shaking at room temperature for 1 h. The agarose bead–antigen–antibody complex was collected after centrifugation at 14,000 rpm for 5 s. The complex was re-suspended in 2× sample buffer, gently mixed, and then boiled for 5 min. Subsequently, SDS–PAGE was performed. After electrophoresis, the proteins were transferred onto polyvinylidene fluoride membranes. The membranes were then blocked with 5% skimmed milk at room temperature for 1.5 h, incubated with the primary antibody overnight at 4 °C, and washed thrice with TBST. After being incubated with the secondary antibody at room temperature for 1 h, the membranes were washed three times with TBST. Finally, protein expression changes were detected using an enhanced chemiluminescence detection system. Alternatively, after electrophoresis, the gel was stained with Coomassie Brilliant blue, which was sent to Beijing Novogene Science and Technology Co., Ltd (Beijing, China) for proteomic analysis of interacting proteins.

### ISGylation assay and proteomics analysis on ISGylation sites

MCF-7 or MDA-MB-231 cells were treated with 20 μM MG132 for 8 h before harvesting. Cells were lysed by boiling in buffer (50 mM Tris–HCl, 150 mM NaCl, 1% SDS, 0.5% deoxycholate, 10 mM dithiothreitol) for 10 min. The cell lysates were then diluted tenfold with lysis buffer containing 1% NP-40 (Beyotime), phenylmethylsulfonyl fluoride (Beyotime), Phosphatase Inhibitor Cocktail (Beyotime), and E3876 (Sigma-Aldrich). These lysates were subjected to IP analysis using a CYP4Z1 antibody (DF10128, Affinity, Cincinnati, OH, USA). The ISGylation of endogenous CYP4Z1 was detected by western blotting. To analyze the exogenous CYP4Z1 ISGylation, HEK-293T cells were transfected with plasmids encoding Flag-tagged CYP4Z1 and either His-tagged or HA-tagged ISG15. Co-IP assays were performed to assess the ISGylation of CYP4Z1. For the identification of ISGylation sites, the ISG15 coding sequences were inserted into the pcDNA4/HisMax plasmid (pc-ISG15). Site-directed mutagenesis was used to mutate the LRGG amino acid sequence at the C terminus of the ISG15 protein to LKGG, resulting in pc-ISG15-mut. HEK293T cells were co-transfected with pc-ISG15-mut and pc-Z1-wt, followed by a co-IP experiment using an anti-Flag antibody. The Coomassie Brilliant blue-stained gel strips from the co-IP electrophoresis were sent to Beijing Novogene Co., Ltd for qualitative proteomic analysis of PTMs. Endoproteinase Lys-C (Sequencing grade, Catalog no. 11047825001, Sigma-Aldrich), which specifically recognizes the LKGG sequence, was used for enzymatic digestion.

### Quantitative real-time PCR (qRT-PCR)

Total RNA was extracted from cells using the TransZol Up reagent (ET111-01-V2, TransGen Biotech, Beijing, China) according to the manufacturer’s instructions, followed by cDNA preparation using Hiscript III Reverse Transcriptase (Vazyme). The real-time PCR assays were performed in triplicate using SYBR Green Supermix (Vazyme) with the Applied Biosystems QuantStudio 5 (A28139, Thermo Fisher Scientific). The sequences of the primers used for qRT-PCR were described in Supplementary Table 7.

### Immunofluorescence (IF) assay

For IF assays, cells were cultured in chamber slides overnight (if a probe (refer to Supplementary Table 8) was used, it was applied before the cells are fixed, and the use of the probe should follow the instructions provided, incubating at 37 °C for 30 min) and fixed with 3.7% formaldehyde in PBS for 10 min at 4 °C, followed by permeabilization with 0.5% Triton X-100 in PBS for 10 min. Cells were then blocked for nonspecific binding with 10% goat serum in PBS and 0.1% Tween-20 (PBST) for 1 h at room temperature and incubated with the indicated antibody overnight at 4 °C, followed by incubation with Alexa Fluor-labeled or FITC-labeled secondary antibody for 50 min at room temperature. Coverslips were mounted on slides using the anti-fade mounting medium with 4′,6-diamidino-2-phenylindole (Beyotime). IF images were acquired on a Zeiss LSM 800 Confocal Laser Scanning Microscopy (Jena, Germany). For each channel, all images were acquired with the same settings. The relevant probes used in the experiments were listed in Supplementary Table 8.

### Enzyme activity assay for CYP4Z1

Lytic P450-dependent bioluminescence assays were conducted according to the manufacturer’s instructions (Promega, Madison, WI, USA). To detect CYP4Z1 activity, 12.5 μl of an inhibitor mixture was added to each well at the indicated concentrations before the CYP reactions. Then, 12 μl of HEK293T cell lysate, with or without CYP4Z1 overexpression, and 0.5 μl of Luciferin-CEE (Catalog no. V8752, Promega) were mixed with the inhibitor mixture in 96-well plates. The plates were preincubated at 37 °C for 10 min, after which 25 μl of a 2× NADPH regeneration system (Catalog no. V9510, Promega) was added to each well. Once biotransformation was completed, an equal volume of Luciferin detection reagent (Promega) was added to each well, and the plates were gently swirled to facilitate the formation of cell lysates. The plates were then incubated at room temperature for 20 min, and luminescence was measured using an Infinite M200 PRO Multimode Microplate Reader (Tecan; Infinite).

### Wound healing analysis

When cell confluence reached 90% in six-well plates, a wound was created on the cell surface, and cell debris was washed away with PBS twice. To exclude the effects of cell proliferation on cell migration ability, cells were then cultured in a serum-starved medium (≤2%) containing the compounds or solvent. The migration rate was calculated as follows: Migration rate = (*D*_t_ − *D*_0_)/*D*_0_, in which *D*_t_ represents the distance between the wound edges at different time points and *D*_0_ represents the initial wound distance at 0 h.

### Transwell invasion analysis

Millicell® hanging cell culture inserts (Catalog no. MCEP24H48, Merck) precoated with Matrigel matrix (Catalog no. 356234, Corning) were placed into 24-well plates. A total of 2 × 10^5^ MCF-7 or MDA-MB-231 cells in 200 μl of serum-free medium containing different compounds or solvents were added to the upper chamber, and 800 μl of medium containing 20% serum was used as a chemoattractant in the lower chamber. After 24 h for MDA-MB-231 cells and 48 h for MCF-7 cells, the cells were fixed with 70% ethanol for 20 min at room temperature. The invaded cells were then stained with crystal violet staining solution (Catalog no. C0121, Beyotime) and observed under a microscope. Finally, the chambers were washed with 33% glacial acetic acid, and the absorbance was measured at 570 nm using a Bio-Rad iMark (Hercules, CA, USA). The measured values represent the number of invaded cells.

### Flow cytometry analysis of lipid droplet formation

Cells were cultured under the relevant research conditions. At the time point of interest, a 10 μM BODIPY™ 493⁄503 (Catalog no. D2191, Thermo Fisher Scientific) staining solution was prepared in PBS. The cells were washed twice with PBS, followed by adding the BODIPY staining solution and incubating at 37 °C for 15 min. After two additional PBS washes, the cells were trypsinized to obtain a single-cell suspension, which was subsequently analyzed by flow cytometry.

### Lipidomic identification and analysis

Lipidomics analysis was conducted by Beijing Novogene Science and Technology Co., Ltd. The experimental process included sample collection, lipid extraction, and liquid chromatography–mass spectrometry (MS)/MS detection. The raw data obtained from MS were processed using CompoundDiscoverer 3.1 software for spectral analysis and database searching to achieve qualitative and quantitative results of lipid compounds. Quality control measures ensured data accuracy and reliability. Multivariate statistical analyses were performed to identify differences in metabolic patterns between groups, including PCA and partial least-squares discriminant analysis. Hierarchical clustering analysis and correlation analysis were subsequently used to elucidate relationships between samples and lipid compounds.

### Cellular thermal shift assay (CETSA)

Cells were collected and proteins were extracted. The samples were divided into two equal parts and incubated with an equal volume of DMSO or BM-51 (500 μM) at room temperature for 30 min. Subsequently, each group of samples was further divided into 12 portions and incubated at temperatures ranging from 54 °C to 76 °C, with each 2 °C interval, for 5 min. After incubation, the samples were cooled to room temperature, centrifuged, and the supernatant was collected. Loading buffer (5×) was added and the mixture was boiled. The resulting samples were subjected to 10% SDS–PAGE for western blotting.

### Drug Affinity Responsive Target Stability (DARTS)

Cells were collected, and proteins were extracted. The protein concentration was adjusted to 4–6 μg/μl using 10× TNC (Tris–NaCl–Casein) buffer, and the samples were divided into two equal parts. Each portion was incubated with either an equal volume of DMSO or BM-51 (500 μM) at room temperature for 30 min. Subsequently, each group of samples was divided into six parts and incubated with Pronase solution (Catalog no. 10165921001, Roche, Basel, Switzerland) at varying ratios (1 μg Pronase per 100 μg protein and so on) at room temperature for 30 min. The reaction was terminated by adding 5× loading buffer and boiling the mixture. The resulting samples were subjected to 10% SDS–PAGE for western blotting.

### Molecular docking studies

For docking purposes, the homology model of CYP4Z1 was used as a protein receptor, which was generated according to our previous study^32^. The protein and ligand preparation module in Schrodinger was used to deal with the protein and ligand. Grid generation module was used to generate grid box centered around the heme iron atom with 8 × 8 × 8 Å. The dockings were performed using the Glide standard precision (GlideSP) mode with the ligands as flexible, reject poses with Coulomb–van der Waals energy greater than 5 kcal/mol, and all other options were left at their default values. Then the poses were visualized with PyMOL.

### Molecular simulation dynamics

To investigate the interaction dynamics between BM-51 and CYP4Z1, molecular dynamics (MD) simulations were conducted. The initial structure for MD simulations was based on the predicted binding mode of BM-51 to the active site of CYP4Z1 obtained from molecular docking. First, the complexes were prepared using the ProteinPrepWizard module of MAESTRO. The prepared complexes were solvated in a cubic box using the TIP3P water model, with a 15 Å buffering distance between the protein and the box edges. To neutralize the system charge, an appropriate amount of sodium or chloride ions was added, and an additional 0.15 M NaCl was included to mimic physiological conditions. After setting up the solvated system, energy minimization and relaxation were performed using the default protocol in the Desmond module. MD simulations were carried out in the NPT ensemble with periodic boundary conditions. Temperature and pressure were maintained at 300 K and 1 atm, respectively, using the Nose−Hoover thermostat and isotropic scaling. Subsequently, 200 ns MD simulations were executed using the DESMOND simulation engine and the OPLS-AA force field. Trajectories of the complexes were saved every 100 ps for analysis. Finally, the last conformation of the complex was extracted for binding mode analysis, and root-mean-square deviation and root-mean-square fluctuation analyses were conducted using DESMOND.

### Xenograft animal model

Six-week-old BALB/c male nude mice were purchased from GemPharmatech Co., Ltd (Nanjing, China) and used in the xenograft experiments. MCF-7 cells with or without stably-ectopic expression of transcripts were implanted into the dorsal flanking sites of nude mice at 1 × 10^5^, 1 × 10^6^, and 1 × 10^7^ cells in 200 µl PBS. Lin-NECs were implanted into the dorsal flanking sites of nude mice at 1 × 10^4^, 1 × 10^5^, and 1 × 10^6^ cells in 200 µl PBS. Two weeks after injection, mice-bearing tumors were sacrificed for the assessment of tumor size and weight examination, as well as stem cell frequencies through extreme limiting dilution analysis^33^.

### Tumor metastatic analysis

BALB/C nude mice aged 4–6 weeks were selected. The cell suspension was injected into caudal vein (5 × 10^5^ cells/200 μl) twice on 2 days. After 3 weeks, the lung and liver tissues of mice were removed entirely and photographed. After fixation, HE staining and microscopic examination were performed.

### Evaluation of the antitumor effects of BM-51 in vivo

For evaluating the effects of BM-51 on Adriamycin sensitivity of breast cancer cells, all nude mice were inoculated with MCF-7 cells (5 × 10^6^ cells per mouse) mixed 1:1 with Matrigel matrix (BD Biosciences) under the left subcutaneous. When the tumors reached the volume of ~100 mm^3^, we randomly allocated the mice to groups in which they received Adriamycin (0.5 mg/kg, Shenzhen Main Luck Pharmaceuticals Inc., China), PBS, 10 mg/kg BM-51, Adriamycin + 10 mg/kg BM-51. Tumor growth was monitored by caliper measurements. Tumor volume was calculated by the following formula: Volume (mm^3^) = *L* (length) × *W* (width)^2^ × 1/2. Mice were euthanized 14 days after the inoculation. The weight of each tumor was measured and mice organs were used for HE staining (Servicebio, Wuhan, China).

For evaluating the effects of BM-51 on the tumor-initiating ability of breast cancer cells, 6-week-old BALB/c male nude mice were used in the xenograft experiments. MCF-7 and MDA-MB-231 cells were pretreated with BM-51 for 72 h and then were implanted into the dorsal flanking sites of nude mice at 1 × 10^5^, 1 × 10^6^, and 1 × 10^7^ cells in 200 µl PBS. Two weeks after injection, mice-bearing tumors were sacrificed for the assessment of tumor size and weight examination, as well as stem cell frequencies through extreme limiting dilution analysis^33^.

For evaluating the effects of BM-51 on the metastatic ability of breast cancer cells, MDA-MB-231 cells (2 × 10^6^ cells per mouse) were injected (intravenously) into mice, which were randomly allocated to groups with or without BM-51 (10 mg/kg) treatment. After 21 days, mice were euthanized and lung tissues were subjected to HE staining to observe the lung metastatic nodes. Additionally, *PyMT-MMTV* and *PyMT-CYP4Z1* mice were treated with BM-51 from week 9 and lasted for 6 weeks. The process of tumor progression between *PyMT-MMTV* and *PyMT-CYP4Z1* mice with or without BM-51 treatment was compared by observing tumor initiation, growth, metastasis, and whole-mount staining of the mammary gland.

### Chemical synthesis

See Supplementary Note.

### Statistical analysis

Statistical analysis was performed using Prism 8.0 (GraphPad Software, La Jolla, CA, USA). For two-group comparisons, two-tailed unpaired *t* tests were performed; for multiple-group comparison, two-way analysis of variance of post hoc test was performed. *P*-values less than 0.05 were considered significant.

## Results

### Knock-in of CYP4Z1 promotes the formation and metastasis of *PyMT*-induced mammary tumor and increases the expansion of *PyMT*-induced TICs

**Fig. 1:**
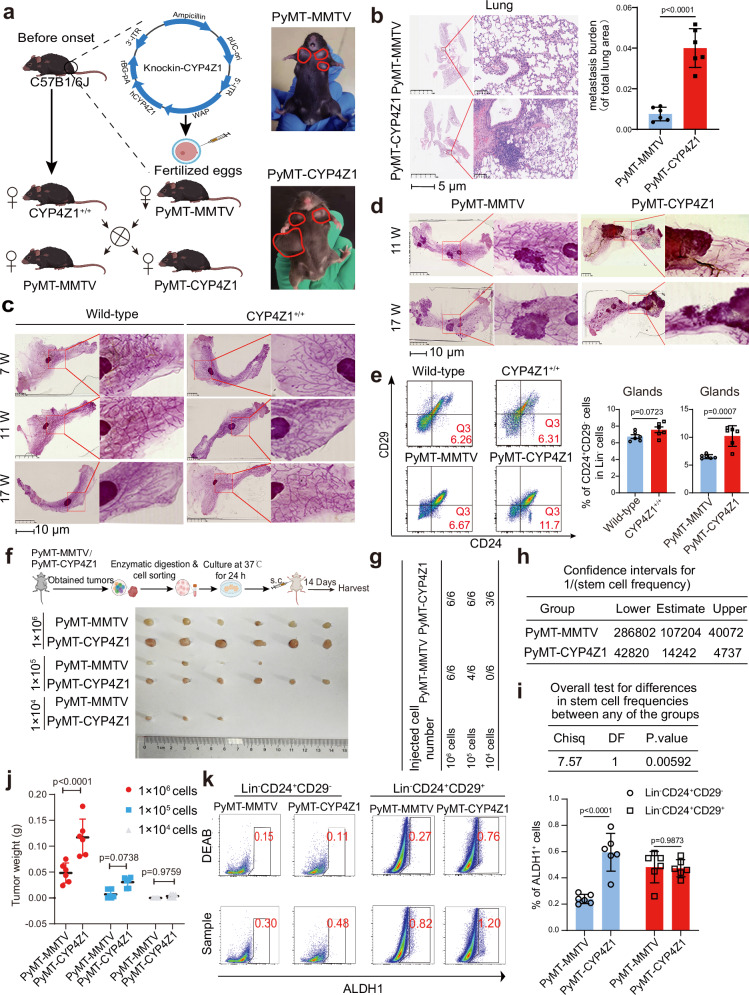
Knock-in of *CYP4Z1* promotes the formation and metastasis of *PyMT*-induced mammary tumors and increases the expansion of *PyMT*-induced TICs. **a** Tumors were palpable in mice at 120 days, with tumors forming in mice in the red circle. **b** Hematoxylin and eosin staining results of metastatic nodules in lung tissue and liver tissue at the age of 120 days in mice. Images were representative of images from six independent experiments. **c** Whole-mount carmine red staining of mammary glands from WT (wild-type) and *CYP4Z1*^*+/+*^ mice at 7, 11, and 17 weeks of age. Images were representative of images from four independent experiments. **d** Whole-mount carmine red staining of mammary glands from *PyMT-MMTV* and *PyMT-CYP4Z1* mice at 11 and 17 weeks of age. Images were representative of images from six independent experiments. **e** Flow cytometry shows the content of progenitor cells in the mammary cavity of mice after sorting. Images were representative of images from three independent experiments. Data were presented as mean ± SEM (*n* = 6 independent experiments), two-sided Student’s *t* test. **f** Lin-mammary epithelial cells were isolated from tumors derived from late-stage *PyMT-MMTV* and *PyMT-CYP4Z1* mice and subjected to tumor-initiating assays. Tumor images derived from cells at different cell numbers were shown. **g–i** The tumor formation ratio was calculated. The 1/(stem cell frequency) was measured using ELDA (extreme limiting diffusion analysis) online tool (http://bioinf.wehi.edu.Au/software/Elda/). The overall test for frequencies between groups was also determined using the ELDA online tool. **j** Tumor weight was detected. Data were presented as mean ± SEM, two-way analysis of variance test. **k** CD29^lo^CD24^+^ALDH^+^ subset was evaluated in Lin-mammary epithelial cells from tumors derived from late-stage *PyMT-MMTV* and *PyMT-CYP4Z1* mice. Images represented three independent experiments. DEAB group, the control of the ALDH1 staining experiment. Sample group, the experimental group for the ALDH1 staining assay. The percentage of ALDH1^+^ cells is obtained by subtracting the deab value from the corresponding sample. Data were presented as mean ± SEM (*n* = 6 independent experiments), two-way analysis of variance test. ALDH, aldehyde dehydrogenase; DF, degree of freedom; s.c., subcutaneous.

To confirm the roles of endogenously-derived *CYP4Z1* during breast cancer progression in vivo, we compared the process of tumor progression between *PyMT-MMTV* and *PyMT-CYP4Z1* mice. In *PyMT-MMTV* mice, palpable tumors developed in 98-day-old animals, with 50% of the mice displaying frank tumor growth by day 120 (Fig. 1a). By contrast, tumor initiation in *PyMT-CYP4Z1* mice advanced significantly, with 50% of mice developing tumors only after 77 days (Fig. 1a and Supplementary Fig. 1a). Additionally, compared with *PyMT-MMTV* mice, *PyMT-CYP4Z1* mice exhibited a significant increase in tumor burden, including tumor number, size, and weight (Fig. 1a and Supplementary Fig. 1a), and developed numerous metastatic nodules in the lung and liver at 120 days (Fig. 1b and Supplementary Fig. 1b,c). Furthermore, *CYP4Z1* knock-in did not affect branching morphogenesis during mice development (Fig. 1c). We then investigated *CYP4Z1* knock-in effects on the expansion of *PyMT*-induced TICs. Mammary gland whole-mounts prepared from *PyMT-MMTV* and *PyMT-CYP4Z1* mice were isolated at hyperplastic/pre-neoplastic stages of growth (10–17 weeks of age) to determine the effect of *CYP4Z1* knock-in on tumor initiation. As shown in Fig. 1d, by 11 weeks of age, glands from *PyMT-CYP4Z1* displayed markedly larger hyperplastic lesions compared with the *PyMT-MMTV* control mice. By 17 weeks of age, the *PyMT-CYP4Z1* mice exhibited increased numbers of hyperplastic foci in the more distal regions of the mammary gland relative to *PyMT-MMTV* control mice (Fig. 1d and Supplementary Fig. 1d). As *PyMT-MMTV* mice exhibit multiple similarities to the luminal B subtype of human breast cancer^34,35^, to elucidate *CYP4Z1*'s role in the expansion of MECs, Lin-epithelial cells were profiled from normal or *PyMT-MMTV* pre-neoplastic mammary glands using surface markers CD29^lo^CD24^+^ representing luminal progenitor cells, which have been reported to display TIC properties in *PyMT*-induced tumors^36,37^. As shown in Fig. 1e, the percentage of CD29^lo^CD24^+^ subpopulation was comparable in normal glands between WT and *CYP4Z1*^*+/+*^ mice, whereas *PyMT-CYP4Z1* expanded more luminal progenitors relative to *PyMT-MMTV* mice. By contrast, the CD29^+^CD24^+^ basal subpopulation^35^, which enriches for mammary stem cells in specific tumor models^36^, was markedly decreased in *PyMT-MMTV* mammary glands relative to *PyMT-CYP4Z1* glands (Fig. 1e). We then wondered whether *CYP4Z1* could maintain TIC function in established tumors, Lin-MECs were isolated from tumors derived from late-stage *PyMT-MMTV* and *PyMT-CYP4Z1* mice and subjected to tumor-formation analysis using the tumor-limiting dilution assay. Tumors were detected at larger frequency and size in mice that received 10^4^, 10^5^, and 10^6^ *PyMT-CYP4Z1* MECs relative to controls (Fig. 1f, g). Consistently, the stem cell frequency was increased in *PyMT-CYP4Z1* MECs (Fig. 1h, i). In addition, the expression of CSC markers (ALDH1A1, Oct4, and Ki67) was also increased in tumors derived from *PyMT-CYP4Z1* mice (Supplementary Fig. 1e). Notably, tumors generated from *PyMT-CYP4Z1* MECs were markedly increased in weight (Fig. 1j). As the CD29^lo^CD24^+^ALDH^+^ subset was considered as the TICs in advanced *PyMT*-induced breast cancers^38^, we measured the percentage of this subset in Lin-MECs and found that it was significantly increased in *PyMT-CYP4Z1* mice (Fig. 1k). Importantly, CYP4Z1 expression was validated in tumors and metastatic nodules (Supplementary Fig. 1f). Our results demonstrate that in addition to promoting the expansion of *PyMT*-induced TICs, *CYP4Z1* can also maintain their function.

### Knock-in of *CYP4Z1* exhibits a decreased differentiated status and enhances cell invasion phenotype

**Fig. 2:**
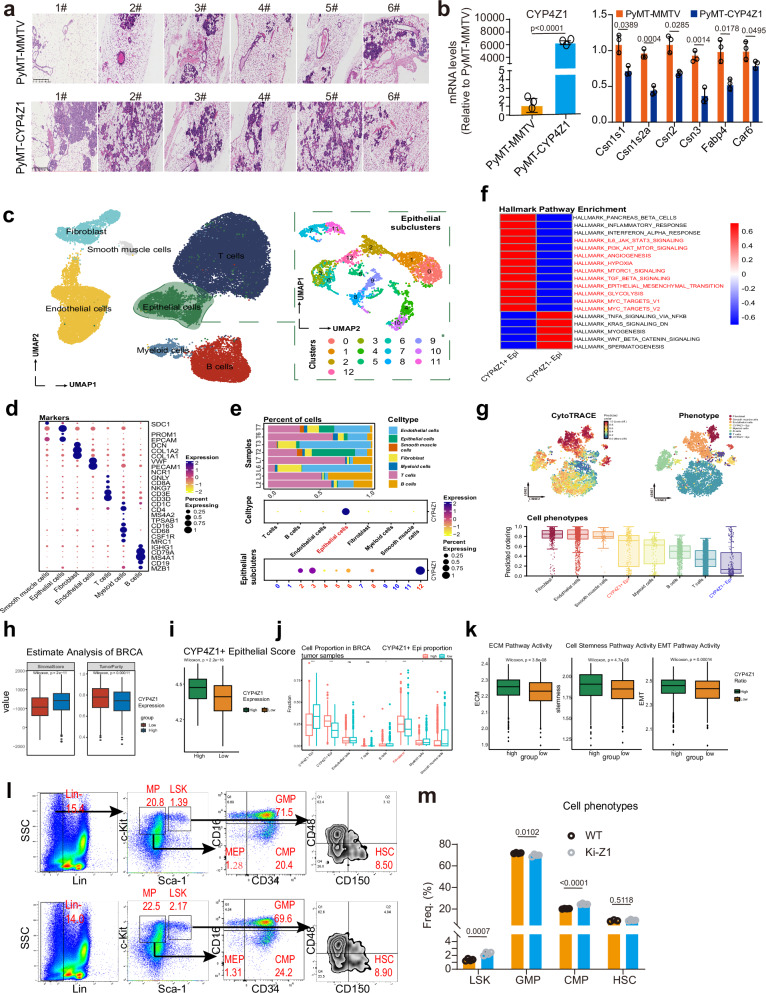
Knock-in of *CYP4Z1* exhibits a decreased differentiated status and enhances cell invasion phenotype. **a** Hematoxylin and eosin staining of glands from 11-week *PyMT-MMTV* and *PyMT-CYP4Z1*. Images were representative of images from six independent experiments. **b** Quantitative real-time PCR of CYP4Z1, Csn1s1, Csn1s2a, Csn2, Csn3, Fabp4, and Car6 in tumor tissues derived from *PyMT-MMTV* and *PyMT-CYP4Z1* mice. Data were presented as mean ± SEM (*n* = 3 independent experiments), two-sided Student’s *t* test or two-way analysis of variance test. **c** Uniform manifold approximation and projection (UMAP) plot of the main cell clusters after dimensional reduction and cell-type annotation of cell clusters via canonical markers from previous studies. In the box, the UMAP plot of epithelial cell clusters after re-dimensional reduction was also visualized. **d** Cell marker expression for cluster annotation. **e** The cell proportions in each sample (upper panel), the expression of CYP4Z1 in main cell clusters (middle panel) and epithelial cell clusters (lower panel). **f** Enriched pathways in CYP4Z1^+^ and CYP4Z1^−^ Epi cells, analyzed by “escape” package. **g** The cell stemness distribution and phenotype of cell clusters in t-distributed stochastic neighbor embedding (t-SNE) dimension reduction plot (upper panel). The boxplot of CytoTRACE score of cell clusters in single-cell samples (lower panel). **h** StromalScore and tumor purity of high and low CYP4Z1 expression groups of TCGA-BRCA dataset were calculated by “estimate”. **i** Evaluation of CYP4Z1^+^ Epi characteristics in high and low CYP4Z1 expression groups of TCGA-BRCA dataset, calculated by Gene Set Variation Analysis. **j** Estimated cell proportions in TCGA-BRCA tumor dataset, performed by Bayes deconvolution. Samples were divided into high and low groups based on the median value of CYP4Z1^+^ Epi proportion. **k** Pathway activities of extracellular matrix (ECM), cell stemness, and epithelial–mesenchymal transition (EMT) in high ratio and low ratio groups, calculated by Gene Set Variation Analysis. **l** Representative flow cytometry profiles showing gating strategy and changes in various bone marrow HSPC subsets. Images were representative of images from three or four independent experiments. **m** Quantification of HSC subsets, data were presented as mean ± SEM. *n* = 3 independent experiments. *Ki-Z1, CYP4Z1*^*+/+*^, two-way analysis of variance of post hoc test. CMP, common myeloid progenitor; GMP, granulocyte–macrophage progenitor; HSC, hematopoietic stem cell; LSK, Lineage^−^/Sca-1^+^/c-Kit; MEP, megakaryocyte–erythroid progenitor; WT, wild type.

At 11 weeks, the glands from *PyMT-CYP4Z1* mice displayed an obvious promotion in tumor progression and a de-differentiated phenotype compared with that of *PyMT-MMTV* mice with progression to differentiated adenocarcinomas (Fig. 2a and Supplementary Fig. 2a). Additionally, we found that the expression of differentiation markers (*Csn1s1*, *Csn1s2a*, *Csn2*, *Csn3*, *Fabp4*, and *Car6*) was decreased in *PyMT-CYP4Z1* glands^35,39^ (Fig. 2b). Furthermore, cytokeratin-14 (K14)-positive (K14^+^) cells were enriched at the protrusive border of *PyMT-CYP4Z1* adenocarcinomas, which have been identified as the invasive leader cancer cells at the tumor–stromal cell interface in both mouse breast cancer models and human patients, forming multicellular strands that invade into the adjacent stromal tissues (Supplementary Fig. 2a,b). By contrast, *PyMT-MMTV* adenocarcinomas displayed a weaker invasive phenotype, and the K14^+^ cells within tumor masses maintained smooth borders with the surrounding stromal interface, as well as the increase of pro-apoptotic (cleaved caspase 3^+^) cells in both hyperplastic and carcinomatous lesions (Supplementary Fig. 2b,c). Moreover, glands from *PyMT-CYP4Z1* mice exhibited increased proliferative (Ki67^+^) cells (Supplementary Fig. 2d,e). We then analyzed CYP4Z1 expression through single-cell data GSE225600, which contains single-cell RNA and spatial transcriptome of four primary tumors and four paired metastatic lymph nodes in breast cancer^40^. Main cell clusters were identified in GSE225600 (Fig. 2c, d). Cell markers for annotations were obtained from previous research articles. The proportions of main types of cells were visualized in Fig. 2e. Moreover, we further explored the expression of *CYP4Z1* in single-cell data and identified that *CYP4Z1* was concentratedly expressed in epithelial cells in breast cancer. Thus, we further extracted and re-clustered these epithelial cells. According to the expression or non-expression of *CYP4Z1* in these clusters, clusters 2, 3, 4, 5, 6, 8, and 12 were identified as the *CYP4Z1*^*+*^ Epi cells, whereas the other clusters were annotated as *CYP4Z1*^−^ Epi cells (Fig. 2c, e). Using “escape” method, we performed the functional enrichment on *CYP4Z1*^+^ and *CYP4Z1*^−^ Epi based on the hallmark datasets downloaded from MsigDB database (Fig. 2f). The results indicated that several pathways related to tumor growth and metastasis were upregulated in the *CYP4Z1*^+^ Epi group, including hypoxia, epithelial–mesenchymal transition (EMT), glycolysis, JAK-STAT3, and transforming growth factor (TGF)-beta pathways. Hypoxia and glycolysis contributed to the formation of tumor microenvironment, EMT promoted the metastasis of tumor cells, and the activated TGF-beta and JAK-STAT3 can regulate tumor metabolism, promoting immune evasion, proliferation, and survival of breast tumor. Knock-in of *CYP4Z1* suggested a decrease differentiated phenotype of breast tumor. We further conducted CytoTRACE analysis on single-cell level to validate the results. Here, compared with *CYP4Z1*^*−*^ Epi cells, *CYP4Z1*^*+*^ Epi cells showed relatively higher stemness and reduced differentiation (Fig. 2g). Generally, we observed that *CYP4Z1* expression in breast cancer epithelial cells is associated with a less-differentiated and more stem-like phenotype in breast cancer. *CYP4Z1*^+^ Epi cells exhibited upregulation of key tumor-promoting pathways, including hypoxia, EMT, glycolysis, JAK-STAT3, and TGF-beta signaling, which collectively contribute to an aggressive tumor microenvironment.

Moreover, most of the *CYP4Z1*^*+*^ Epi characteristic genes showed upregulated expression either at the start or at the end of differentiation, whereas *CYP4Z1* expression was increased and decreased sharply in the middle stage of differentiation (Supplementary Fig. 3a). Correspondingly, two cell differentiation tracks were developed in the middle differentiation stage: one toward *CYP4Z1*^−^ Epi and one toward T cells, which were two types of cells with lowest stemness analyzed by CytoTRACE (Supplementary Fig. 3b). Importantly, compared with *CYP4Z1*^−^ Epi, *CYP4Z1*^*+*^ Epi cell clusters showed higher interaction frequency and intensity with other clusters, including myeloids, B cells, and endothelial cells (Supplementary Fig. 3c,d). Notably, *CYP4Z1*^*+*^ Epi showed widely varied outgoing and incoming communication pathways (Supplementary Fig. 3e).

To further evaluate the characteristics of *CYP4Z1*^+^ Epi in breast cancer, we downloaded TCGA-BRCA bulk-seq data. As *CYP4Z1* was identified to promote the metastasis and invasion of breast cancer, we analyzed the tumor purity of TCGA-BRCA samples with different expression levels of *CYP4Z1*. The results indicated that stromal score was notably higher in the high *CYP4Z1* expression group than in the low expression group, with opposite trend of tumor purity between two groups (Fig. 2h). These results suggested that *CYP4Z1* expression might indicate higher activity of stromal cells (for example, cancer-associated fibroblasts) in tumor microenvironment, facilitating tumor metastasis and migration. Furthermore, we performed “FindMarkers” analysis on *CYP4Z1*^+^ Epi to identify significant differentially expressed genes of *CYP4Z1*^+^ Epi, which were subsequently inputted as the gene set for GSVA on breast cancer bulk-seq samples. The results of GSVA indicated that the characteristic of *CYP4Z1*^+^ Epi cells was significantly higher in samples with *CYP4Z1* expression than those with low expression (Fig. 2i), which might primarily indicate the synthesized variation of *CYP4Z1* expression and *CYP4Z1*^+^ Epi proportion in breast cancer. Thus, we conducted the Bayes deconvolution of single-cell data on TCGA-BRCA dataset to estimate the proportion of cells in breast cancer bulk samples. Here, we divided the tumor samples into two groups, based on the median value of *CYP4Z1*^+^ Epi proportion of them. Interestingly, fibroblasts showed significant differential proportion between two groups, which was corresponding to the tumor purity results. By contrast, we noticed that both in high *CYP4Z1* expression group and in high *CYP4Z1*^+^ Epi proportion group of breast cancer bulk samples, the stromal cell proportion was upregulated (Fig. 2j). However, these results alone were insufficient to determine whether the observed stromal enrichment was directly linked to the expression of *CYP4Z1* in *CYP4Z1*^+^ Epi cells. Considering that gene expression alone may be influenced by tumor burden or contributions from other cell types, and cell proportion alone does not reflect the functional activity of *CYP4Z1*^+^ Epi cells, we integrated both factors by multiplying *CYP4Z1* expression with the *CYP4Z1*^+^ Epi proportion: ${CYP}4Z1+\mathrm{Epi}\,\mathrm{proportion}=\mathrm{prop}({CYP}4Z1+\mathrm{epithelial}\,\mathrm{cell})\ast \exp ({CYP}4Z1)$. This approach allowed us to more accurately assess whether the transcriptional changes and stromal remodeling were specifically associated with *CYP4Z1* in *CYP4Z1*^+^ Epi cells rather than a general feature of bulk tumor composition (Fig. 2k). Subsequently, we downloaded pathway gene sets of extracellular matrix (ECM), EMT, and cell stemness (KEGG_ECM_RECEPTOR_INTERACTION.v2024.1.Hs, HALLMARK_EPITHELIAL_MESENCHYMAL_TRANSITION.v2024.1.Hs, GALIE_TUMOR_STEMNESS_GENES.v2024.1.Hs) from MsigDB database. The GSVA of ECM, EMT, and cell stemness pathway gene sets validated that compared with the low *CYP4Z1*^+^ Epi proportion group, the high *CYP4Z1*^+^ Epi proportion group showed higher activity in ECM, EMT, and cell stemness pathways, which contributes to the decreased differentiated status and upregulated tumor metastasis and invasion phenotypes.

The spatial transcriptomic analysis was further conducted based on the epithelial cell markers and *CYP4Z1*^*+*^ Epi characteristic genes. The results revealed that the *CYP4Z1*^*+*^ Epi gene expression was mainly enriched in border areas of regions with high epithelial marker expression (Supplementary Fig. 3f). After deconvolution of the annotated dataset GSE225600 and spatial transcriptomic dataset, *CYP4Z1*^−^ Epi showed a low fraction in spatial spots, whereas the *CYP4Z1*^*+*^ Epi cells displayed widely distribution in spatial spots, mainly corresponding to the enriched region of epithelial cell markers (Supplementary Fig. 3g). Correspondingly, in border areas, the proportion of *CYP4Z1*^*+*^ Epi was higher than in inner areas. Importantly, we evaluated whether *CYP4Z1* knock-in could result in any detrimental effects on the functions and multilineage development of normal HSC. We analyzed the HSCs by cell surface markers Lineage^−^/Sca-1^+^/c-Kit^+^ (LSK) and HSC compartments (defined by LSK/CD48^−^/CD150^+^) and various bone marrow progenitor cell subsets comprising granulocyte–macrophage progenitors (defined by Lin^−^/Sca-1^−^/c-Kit^+^/CD16^+^/CD34^+^), common myeloid progenitors (CMPs, defined by Lin^−^/Sca-1^−^/c-Kit^+^/CD16^−^/CD34^+^), and megakaryocyte–erythroid progenitors (defined by Lin^−^/Sca-1^−^/c-Kit^+^/CD16^-^/CD34^-^) in *PyMT-MMTV* and *PyMT-CYP4Z1* mice (Fig. 2l). As quantified in Fig. 2m, the bone marrow progenitor cell subsets were modestly changed in *PyMT-CYP4Z1* mice. Taken together, these results indicate that *CYP4Z1* can promote the acquisition of a de-differentiated phenotype in breast cancer without substantial detrimental effects on normal hematopoiesis.

### ISG15 can directly bind to and induce the ISGylation of CYP4Z1 at K259, K279, and K502

**Fig. 3:**
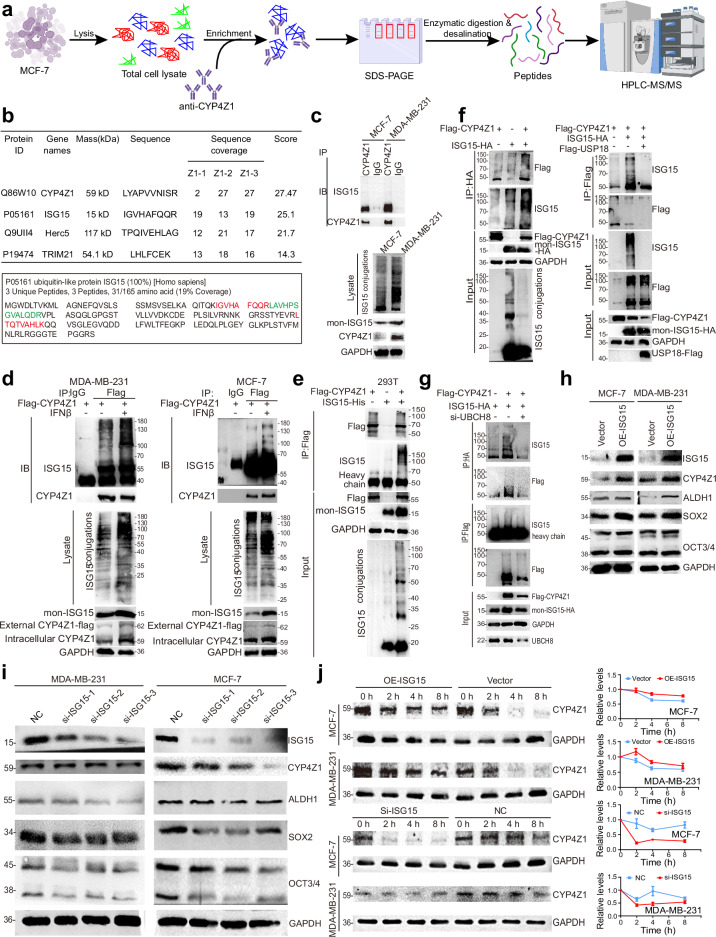
ISG15 can directly bind to and induce the ISGylation of CYP4Z1. **a** The diagram of co-immunoprecipitation (IP) combined with interactome proteomics analysis. **b** The highest-scoring protein was identified by interactive mass spectrometry (MS) and identification peptides derived from ISG15 protein. **c** The endogenous ISGylation level of CYP4Z1 was examined in MCF-7 and MDA-MB-231 cells. **d** ISGylation modification level of CYP4Z1: left MDA-MB-231 and right MCF-7. Samples were collected 48 h after transfection and given to interferon-β (IFNβ) 12 h before adoption. **e–g** Samples of the ISGylation modification level of CYP4Z1 were collected 48 h after transfection. **h**, **i** Immunoblot analysis of ISG15, CYP4Z1, GAPDH, ALDH1A1, SOX2, and OCT3/4 in Vector, OE-ISG15, NC, and si-ISG15 in MCF-7, and MDA-MB-231 in response to ISG15 overexpression or knockdown. **j** CYP4Z1 stability was examined by western blot in MCF-7 and MDA-MB-231 cells with ISG15 overexpression or depletion. The cells were treated with 100 µg/ml cycloheximide (CHX) for the indicated times. Quantitative analysis was performed by measuring the gray values of CYP4Z1 bands and their corresponding GAPDH bands. The CYP4Z1/GAPDH ratios were calculated, normalized, and graphically represented based on the normalized values. *n* = 3. ALDH, aldehyde dehydrogenase; HPLC, high-performance liquid chromatography.

To explore potential PTMs of CYP4Z1, we performed an unbiased proteomic screen by co-IP coupled with MS to identify proteins that interact with CYP4Z1. Among the 218 proteins reproducibly identified across independent replicates, we focussed on those involved in PTM pathways (Fig. 3a). Among the interacting proteins capable of mediating PTMs, the ubiquitin-like protein ISG15 showed the highest interaction score with CYP4Z1 (Fig. 3b). This unbiased observation prompted us to investigate whether ISG15 mediates ISGylation of CYP4Z1. To test this, we examined the ISGylation level of endogenous CYP4Z1 in MCF-7 and MDA-MB-231 cells and found that it was higher in MDA-MB-231 cells than in MCF-7 cells (Fig. 3c). Interferon-β, an inducer of ISG15, was used to induce ISG15 expression in breast cancer cells subjected to detecting CYP4Z1 ISGylation. It was found that the CYP4Z1 ISGylation level was significantly upregulated by interferon-β treatment in breast cancer MCF-7 and MDA-MB-231 cells (Fig. 3d). The exogenous expression of CYP4Z1 and ISG15 in 293T cells also confirmed the ISGylation of CYP4Z1 (Fig. 3e). Additionally, knockdown of UBCH8, an E2 enzyme of ISG15, or overexpression of USP18, a deISGylation modification enzyme, could rescue ISG15-mediated ISGylation of CYP4Z1 (Fig. 3f, g). Furthermore, we showed that ISG15 could positively regulate the expression (Fig. 3h, i) and protein stability (Fig. 3j) of CYP4Z1 in breast cancer cells. Besides, ISG15 ectopic expression positively regulated the expression of stemness markers (Fig. 3h, i). Then we continued to determine the ISGylation sites of CYP4Z1 mediated by ISG15 through modified-qualitative proteomics (Supplementary Fig. 4a) and identified that K220, K248, K259, K279, K309, and K502 were the potential ISGylation sites (Supplementary Fig. 4b). To confirm this result, these lysine sites were first mutated into arginine (R), respectively, and subjected to the detection of CYP4Z1 ISGylation. As shown in Supplementary Fig. 4c, K259, K279, and K502 mutation significantly attenuated the ISGylation of CYP4Z1 mediated by ISG15. Additionally, CYP4Z1 can only be ISGylated when K259, K279, and K502 sites were retained, separately (Supplementary Fig. 4d). Consistently, mutation of K259, K279, and K502, or mutation of these six lysine sites, completely abolished the ISGylation of CYP4Z1 mediated by ISG15 (Supplementary Fig. 4e). Notably, we found that the ISGylation of CYP4Z1 could reduce the ubiquitination of CYP4Z1 and its localization in the proteasome (Supplementary Fig. 4f,g). Collectively, these results demonstrate that ISG15 can induce the ISGylation of CYP4Z1 at K259, K279, and K502.

### CYP4Z1 enhances the TIC-like traits of breast cancer cells dependent on its ISGylation

**Fig. 4:**
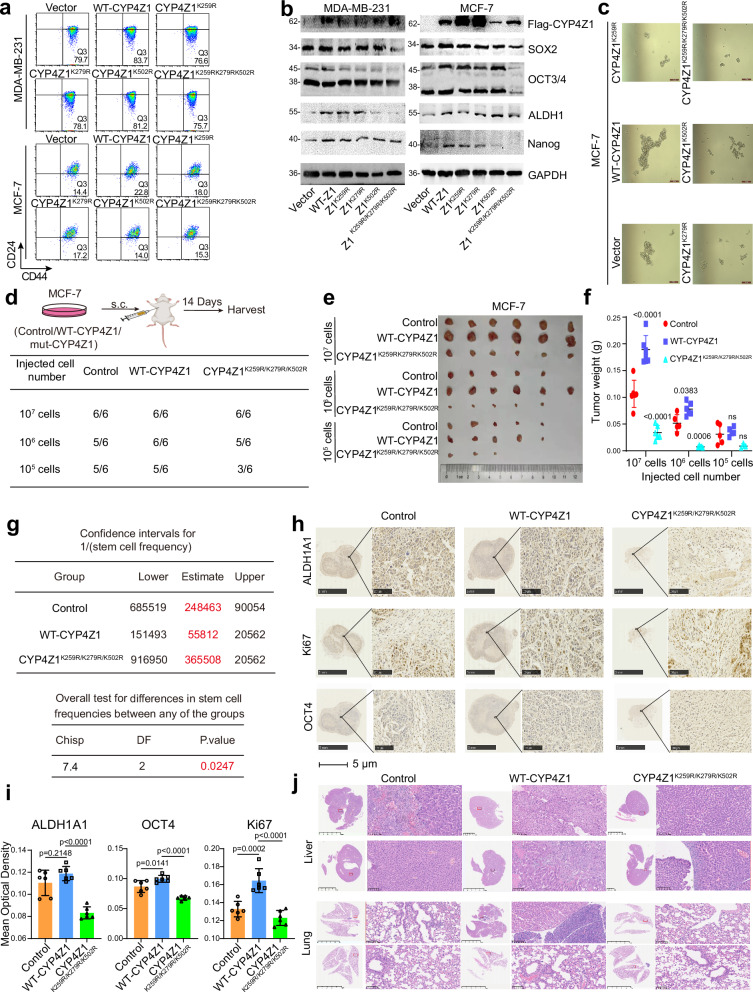
CYP4Z1 enhances the TIC-like traits of breast cancer cells dependent on its ISGylation on K259, K279, and K502 sites. **a** The CD44^+^/CD24^−^ subpopulation with tumor-initiating ability was detected in MCF-7 and MDA-MB-231 cells with CYP4Z1 or CYP4Z1 mutant overexpression. **b** The protein levels of cancer stem cell markers were examined in MCF-7 and MDA-MB-231 cells. **c** Mammary spheroid formation ability was evaluated in MCF-7 cells with CYP4Z1 or CYP4Z1 mutant overexpression. **d** Images of tumors harvested when serially diluted MCF-7 cells with or without wild-type (WT)-CYP4Z1 or mut-CYP4Z1 overexpression, respectively. **e** The tumor formation ratio was calculated. **f** The weight of tumors derived from part **d** was measured. Data were presented as mean ± SEM compared with the control group (*n* = 6 independent experiments). Two-way analysis of variance test. **g** The stem cell frequencies and colligation test for differences in stem cell frequencies were analyzed using the ELDA tool (http://bioinf.wehi.edu.au/software/elda/) based on the results of part **e**). **h**, **i** Immunohistochemistry analysis was performed to detect the expression of stemness markers in tumors from part **d** and quantified in part **i**. The region indicated represents an enlarged view of the specified area. Data were presented as mean ± SEM compared with the control group. One-way analysis of variance test. **j** Images of hematoxylin and eosin staining of mouse liver and lung tissue after metastasis. ALDH, aldehyde dehydrogenase; DF, degree of freedom; ns, not significant; s.c., subcutaneous.

Then, we wondered whether the ISGylation of CYP4Z1 was necessary for CYP4Z1-mediated promoting effects on the TIC-like traits of breast cancer cells. First, it was found that the promoting effects of CYP4Z1 overexpression on the expression of TIC markers (KLF4, Sox2, and ALDH1A1), CD44^+^/CD24^−^ subpopulation with TIC-like traits, and mammary spheroid formation ability were rescued by ISG15 knockdown. Conversely, CYP4Z1 knockdown-mediated inhibitory effects were abolished by ISG15 overexpression (Supplementary Fig. 5). Consistently, both overexpression of USP18 and knockdown of UBCH8 could also attenuate CYP4Z1-induced TIC-like traits of breast cancer cells (Supplementary Fig. 6). Second, we identified that both single and complete mutations of K259, K279, and K502 could weaken the promoting effect of CYP4Z1 on the TIC-like traits of breast cancer cells, as evident by the recovery of CD44^+^/CD24^−^ subpopulation with TIC-like traits (Fig. 4a and Supplementary Fig. 7a,b), the expression of TIC markers (KLF4, Sox2, and ALDH1A1) (Fig. 4b), and mammary spheroid formation ability (Fig. 4c and Supplementary Fig. 7c). We further conducted cell migration and invasion analysis and obtained a consistent result (Supplementary Fig. 7d,e). Third, we performed in vivo tumor-initiation analysis using breast cancer MCF-7 cells with WT CYP4Z1 or CYP4Z1^K259R/K279R/K502R^ overexpression through the tumor-limiting dilution assay. Tumors were detected at larger frequency and size in mice that received 10^5^, 10^6^, and 10^7^ MCF-7 cells with WT-CYP4Z1 overexpression, whereas cells with CYP4Z1^K259R/K279R/K502R^ overexpression displayed a smaller frequency, size, and weight (Fig. 4d–f). Consistently, a reduction in the reciprocal of stem cell frequency was observed in WT-CYP4Z1-overexpressed MCF-7 cells, whereas it was increased in cells with CYP4Z1^K259R/K279R/K502R^ overexpression (Fig. 4g). The detection of the expression of CSC markers (ALDH1A1, Oct4, and Ki67) also obtained a consistent result (Fig. 4h, i). Furthermore, in vivo tumor-metastatic model through tail vein injection also indicated that WT-CYP4Z1 overexpression facilitated the metastatic ability of breast cancer cells, whereas cells with CYP4Z1^K259R/K279R/K502R^ overexpression exhibited a weaker metastatic capability (Fig. 4j and Supplementary Fig. 7f). Notably, a higher CYP4Z1/CYP4Z1 ISGylation level was presented in TICs (CD44^+^/CD24^−^ subpopulation) compared with that in non-TICs (CD44^−^/CD24^+^ subpopulation), both of which were flow-sorted from tumors derived from *PyMT-CYP4Z1* mice (Supplementary Fig. 7g). Altogether, these results suggest that CYP4Z1 enhances the TIC-like traits of breast cancer cells dependent on its ISGylation.

### The ISGylation of CYP4Z1 facilitates its enzyme activity and subcellular localization in endoplasmic reticulum (ER)

**Fig. 5:**
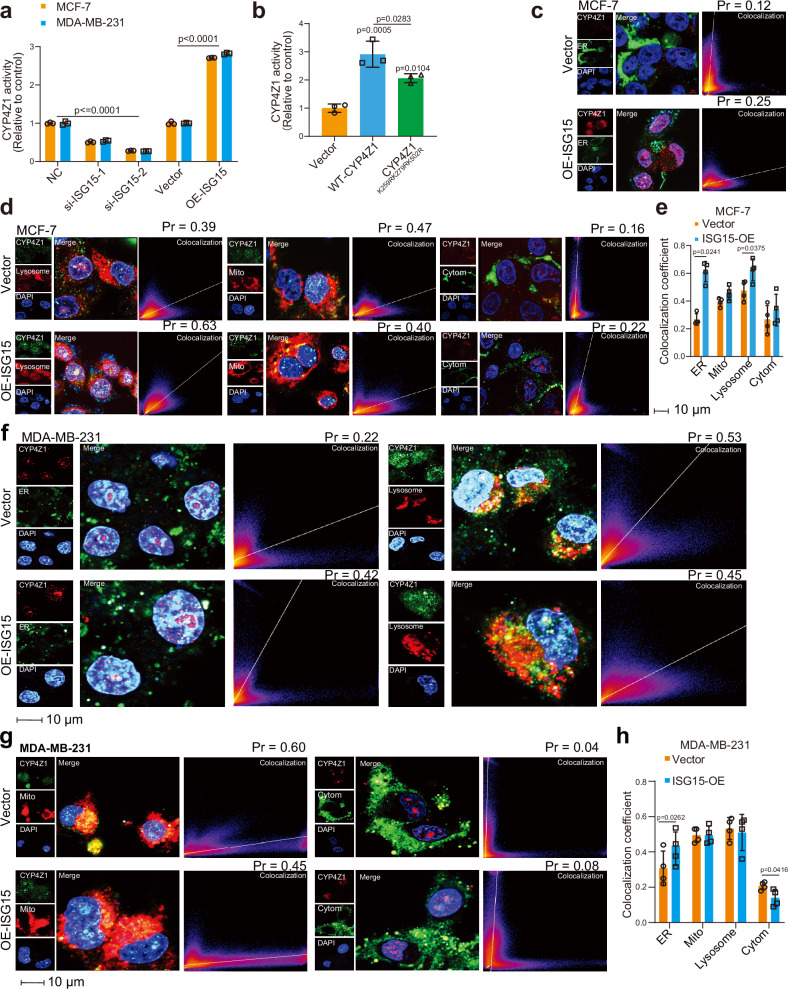
The ISGylation of CYP4Z1 facilitates its enzyme activity and subcellular localization in endoplasmic reticulum. **a** Quantitative results of MCF-7 or MDA-MB-231 cell lysates with or without ISG15 overexpression or knockdown for CYP4Z1 enzyme activity assay. Data were presented as mean ± SEM (*n* = 3 independent experiments), two-way analysis of variance (ANOVA) test. **b** Quantitative results of HEK293T cell lysates with WT-CYP4Z1 or CYP4Z1^K259R/K279R/K502R^ overexpression for CYP4Z1 enzyme activity assay. Data were presented as mean ± SEM compared with the vector group (*n* = 3 independent experiments), one-way ANOVA test. **c**, **d** Colocalization of CYP4Z1 in MCF-7 cells with the endoplasmic reticulum (ER) probe (ER-Tracker™ Green), lysosome probe (LysoTracker™ Red DND-99), mitochondrial probe (MitoTracker™ Deep Red FM), or cytomembrane probe (CellLINK 505). Images were representative of images from four independent experiments. **e** The picture shows the colocalization coefficient between the control and the overexpressed ISG15 groups from parts **c** and **d**. Data were presented as mean ± SEM. *n* = 4 independent experiments, two-way ANOVA test. **f**, **g** Colocalization of CYP4Z1 in MDA-MB-231 cells with the ER probe (ER-Tracker Green), lysosome probe (LysoTracker Red DND-99), mitochondrial probe (MitoTracker Deep Red FM), or cytomembrane probe (CellLINK 505). Images were representative of images from four independent experiments. **h** The picture showed the colocalization coefficient between the control and the overexpressed ISG15 groups from parts **f** and **g**. Data were presented as mean ± SEM. *n* = 4 independent experiments, two-way ANOVA test. Cytom, cell membrane; DAPI, 4′,6-diamidino-2-phenylindole; Mito, mitochondria.

CYP4Z1, as a member of the cytochrome P450 enzyme family, has been shown to have a crucial role in its physiological functions through its enzymatic catalytic activity. Then, we wondered whether the ISGylation of CYP4Z1 could regulate its enzyme activity. It was found that the ectopic expression of ISG15 positively regulated CYP4Z1 enzyme activity in breast cancer cells (Fig. 5a). Notably, K259, K279, and K502 mutations reduced its enzyme activity (Fig. 5b). Furthermore, we determined the effects of the ISGylation of CYP4Z1 on its cellular localization in breast cancer cells. As shown in Fig. 5c–e, overexpression of ISG15 could improve the localization of CYP4Z1 in the ER and lysosome in MCF-7 cells, whereas there was no significant difference in the localization of CYP4Z1 in mitochondria (Mito) and cell membrane (Cytom). Similarly, overexpression of ISG15 to increase the ISGylation level of CYP4Z1 promoted its localization in ER but reduced its localization in Cytom and had no substantial effect in other organelles in MDA-MB-231 cells (Fig. 5f–h). These results suggest that the ISGylation of CYP4Z1 can facilitate its enzyme activity and ER subcellular localization in breast cancer cells.

### The ISGylation of CYP4Z1 promotes the TIC-like traits of breast cancer cells by enhancing TAG production and subsequent formation of lipid droplets

**Fig. 6:**
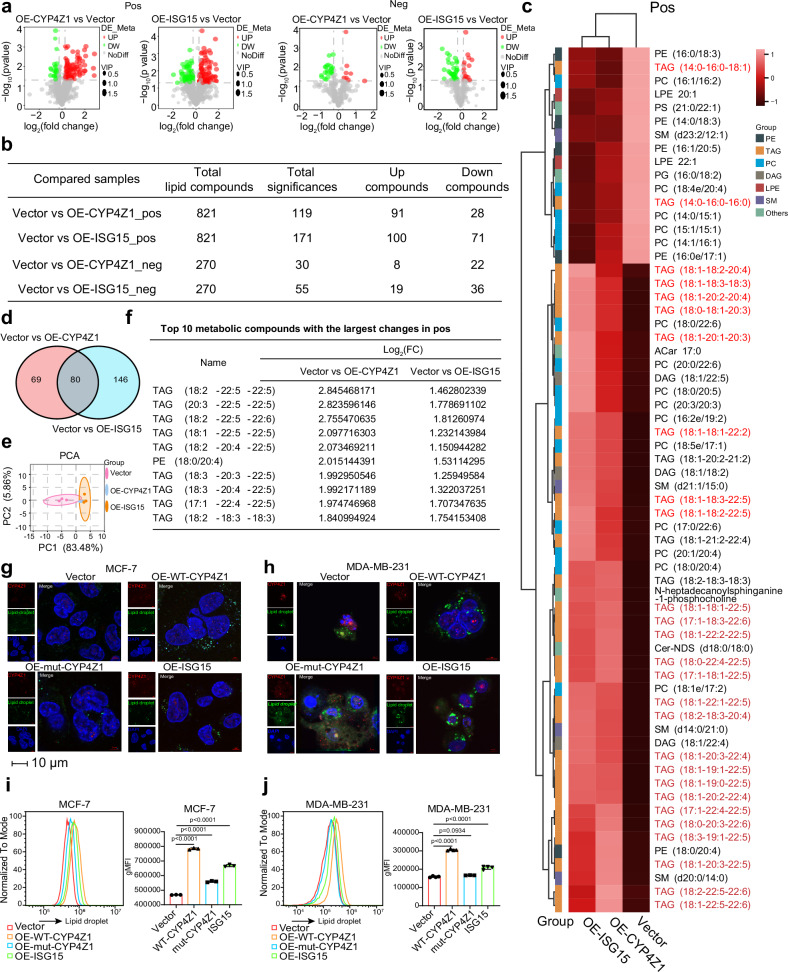
The ISGylation of CYP4Z1 enhances TAG production and thus the formation of lipid droplets. **a**, **b** Results of differential lipid compound analysis. Screening of differential lipid compounds mainly referred to the three parameters VIP, FC, and *P*-value. VIP refers to the variable projection importance in the project value of the first principal component of the PLS-DA model. VIP value represents the contribution of lipid compounds to the grouping; FC refers to fold change as the ratio of the means of all biological replicate quantitation values in the comparison group for each lipid compound; *P*-value was calculated by *t* test and represents the level of significance. Setting thresholds VIP > 1.0, FC > 1.2 or FC < 0.833, and P < 0.05. Each dot in the volcano plot represents a lipid compound, red dots indicate the significantly upregulated lipid compounds, green dots indicated the significantly downregulated lipid compounds, and the size of the dot represents the VIP value. **c** Clustering heat map of total differential lipid compounds in pos. **d** Venn diagram of differential lipid compounds. **e** Principal component analysis (PCA) of all lipid compounds in pos and neg. **f** The 10 lipid compounds with the largest fold changes in pos. **g**, **h** Detection of lipid droplet formation levels in different groups in MCF-7 and MDA-MB-231 cells by immunofluorescence. **i**, **j** Detection of lipid droplet formation levels in different groups in MCF-7 and MDA-MB-231 cells by flow cytometry and quantified. Data were presented as mean ± SEM (*n* = 3 or 4 independent experiments), two-way analysis of variance test. TAG, triglyceride.

We then performed lipidomics analysis on breast cancer MCF-7 cells overexpressing CYP4Z1 or ISG15. The results were shown in Fig. 6a, b; in total, 1091 lipid metabolites were identified through lipidomics. Among these metabolites, overexpression of CYP4Z1 led to differential expression in 149 compounds, with 99 compounds upregulated and 50 compounds downregulated. We further analyzed the types of differentially expressed lipid metabolites. Among the top 20 compounds with the greatest differences, it was observed that overexpression of CYP4Z1 or ISG15 predominantly upregulated members of the triglyceride (TAG) family, whereas several representative metabolites of cardiolipin were downregulated (Fig. 6c and Supplementary Fig. 8a). This suggests that the regulatory function of CYP4Z1 in breast cancer stemness is likely dependent on TAGs. We further investigated the commonalities in downstream metabolic changes caused by overexpression of ISG15 and CYP4Z1. A total of 80 metabolites exhibited similar changes downstream of both overexpressed CYP4Z1 and ISG15, as depicted in Fig. 6d. Additionally, PCA showed that the compositional profiles of compounds treated with OE-ISG15 and OE-CYP4Z1 demonstrated a notable degree of similarity (Fig. 6e). To identify common features among these metabolites, we performed differential analysis of the differentially expressed compounds, evaluating the top 10 compounds with the greatest differences in both cation and anion modes. In the cation mode, the family member of TAG exhibited significant changes, with most of them being upregulated (Fig. 6f). This suggests that CYP4Z1 promotes the generation of TAG family members, and ISG15 may also contribute to this process by regulating CYP4Z1. Another notably changed metabolite was PE (18:0/20:4), a member of the phosphatidylethanolamine family. This family has essential roles in signal transduction and maintenance of cellular functions, and its members have been reported to undergo ubiquitination modification similar to protein modification. In the anion mode, most of the significantly changed metabolites were downregulated, with only *N*-acyl taurine showing a clear upregulation (Supplementary Fig. 8a,b). Among the downregulated compounds, there were many members of the cardiolipin family. Notably, the KEGG analysis also revealed that lipid, cholesterol, and glycerolipid metabolism were enriched in the common differential metabolites (Supplementary Fig. 8c). We also examined the correlation between the expression of various metabolites, as shown in Supplementary Fig. 8d, which reveals a potential correlation between TAG family metabolites and SM (d18:0/26:0), PC (14:1(9Z)/16:1(9Z)), and PC (18:4e/20:4), suggesting a potential mutual inhibition pathway among these metabolites.

ER is the central place for forming lipid droplets, and studies have shown that TAGs were one of the main components of lipid droplets^41^. Thus, we speculated that the ISGylation of CYP4Z1 could promote the formation of lipid droplets. As expected, it was found that whether overexpressing ISG15 or CYP4Z1 promoted the formation of lipid droplets in breast cancer cells, whereas CYP4Z1^K259R/K279R/K502R^ overexpression exerted a weaker effect (Fig. 6g–j). Consistently, the formation of lipid droplets was increased in tumors derived from *PyMT-CYP4Z1* mice compared with that in *PyMT-MMTV* mice (Supplementary Fig. 9a,b). Additionally, CYP4Z1^K259R/K279R/K502R^ overexpression had little effect on the formation of lipid droplets (Supplementary Fig. 9b). Notably, the promoting effects of CYP4Z1 overexpression on the formation of lipid droplets were rescued by ISG15 knockdown and A922500, an inhibitor of TAG production (Supplementary Fig. 9b). Therefore, we speculate that CYP4Z1, as a metabolic enzyme, may promote and maintain the TIC-like traits of breast cancer cells by regulating the downstream fatty acid metabolism, especially the metabolism of TAG. To verify this view, we conducted a series of recovery experiments, and the results were shown in Supplementary Fig. 10a–d; the promoting effects of CYP4Z1 overexpression on the TIC-like traits of breast cancer cells, including CSC makers expression, the ratio of CD44^+^/CD24^−^ subpopulation, and migration ability were rescued by A922500. Conversely, the supplement of TAG (glyceryl tripalmitate) partially reversed the reduction of TIC-like traits of breast cancer cells resulting from CYP4Z1 knockdown (Supplementary Fig. 10e–h). Taken together, our results demonstrate that the ISGylation of CYP4Z1 promotes the TIC-like traits of breast cancer cells by enhancing TAG production and thus the formation of lipid droplets.

### Discovery of a potent CYP4Z1 inhibitor BM-51 that attenuates the TIC-like traits of breast cancer cells

**Fig. 7:**
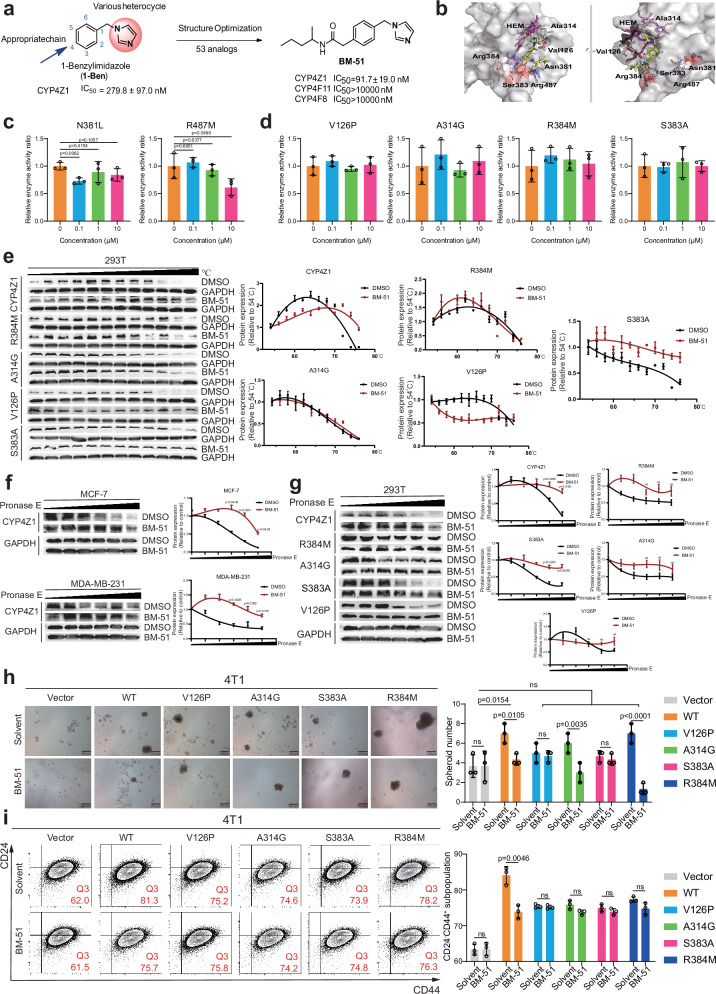
BM-51 can target CYP4Z1 at Arg384, Ser383, Val126, and Ala314 and attenuate the TIC-like traits of breast cancer cells. **a** BM-51 was selected from 53 analogs of 1-ben. **b** The suggested binding model of BM-51 into the homology model of CYP4Z1 was generated from the published structure of CYP4B1 (PDB accession codes, 5T6Q). Hydrogen bonds with key residues were illustrated as red dashed lines. **c**, **d** Enzyme activity assay revealed that BM-51 could reduce the activity of CYP4Z1 with mutated Asn381 or Arg487 but had no effect on mutants at positions Arg384, Ser383, Val126, and Ala314. Data were presented as mean ± SEM (*n* = 4 independent experiments), one-way analysis of variance test. **e**–**g** Cellular thermal shift assay and drug affinity responsive target stability assay revealed that BM-51 could bind to Arg384, Ser383, Val126, and Ala314 sites in CYP4Z1. **h** Mammary spheroid formation ability was evaluated in 4T1 cells with CYP4Z1 or mutants’ overexpression plus BM-51 treatment or not. **i** The CD44^+^/CD24^−^ subpopulation with tumor-initiating ability was detected in 4T1 cells with CYP4Z1 or mutants’ overexpression plus BM-51 treatment or not. Data were presented as mean ± SEM. *n* = 3 independent experiments, two-sided Student’s *t* test. ns, not significant; WT, wild-type.

All of the aforementioned results suggest that CYP4Z1 can be a potential target for breast cancer treatment. Hence, to develop potent and selective CYP4Z1 inhibitors, a series of novel 1-Ben analogs were rationally designed and synthesized from lead compound 1-Ben, which was identified in our previous work^42^. By changing the imidazole ring to various heterocycles and attending the appropriate chain to the benzene ring to increase CYP4Z1 inhibitory activity and selectivity, 53 analogs of 1-Ben were prepared, and their CYP4Z1 inhibitory activities were evaluated (Fig. 7a and Supplementary Table 9), and the detailed structure−activity relationships were summarized (Supplementary Fig. 11a). After carefully evaluating the off-target inhibition effects toward hepatic CYP isozymes (Supplementary Tables 10 and 11), preliminary druggability (Supplementary Table 12), and pharmacokinetics parameters (Supplementary Table 13), a promising compound BM-51 was identified, which exhibited potent inhibitory activity against CYP4Z1 with an IC_50_ value of 91.7 nM and high selectivity against CYP4 isozymes (>100-folds). Furthermore, it was found that BM-51 exhibited favorable pharmacokinetic properties, which guarantees that it acted as a pharmacological agent for in vivo study. As shown in Supplementary Fig. 11b, BM-51 had little effect on cell viability. Additionally, the effects of BM-51 on breast cancer stemness were confirmed through in vitro experiments (Supplementary Fig. 11c– e), which were comparable to that of 1-Ben. In vivo experiments also showed that BM-51 significantly inhibited the tumor-initiating ability of breast cancer cells (Supplementary Fig. 12a–f) and expression of stemness markers (Supplementary Fig. 12g,h). Consistently, BM-51 also exerted significant effects on breast cancer metastasis through in vitro and in vivo experiments with little toxicity on other organs (Supplementary Fig. 13). Then, the homology model combined with molecular docking was applied to the in silico binding hypothesis of BM-51 with CYP4Z1, and the results in Fig. 7b implied that Asn381, Arg487, Arg384, Ser383, Val126, and Ala314 might be crucial for its binding. The further MD simulation experiments confirmed that BM-51 can form a stable bond with the active pocket of CYP4Z1 (Supplementary Fig. 14). Amino acid mutation experiments shown in Fig. 7c, d demonstrated that BM-51 could still reduce the activity of CYP4Z1 with N381L or R487M mutation, whereas it did not affect mutants at positions V126P, R384M, A314G, and S383A. Then we speculated that BM-51 might bind to Arg384, Ser383, Val126, and Ala314 sites in CYP4Z1. CETSA and DARTS results demonstrated that BM-51 could indeed be bound to Arg384, Ser383, Val126, and Ala314 sites in CYP4Z1 (Fig. 7e–g). As mice breast cancer cell line 4T1 did not express *CYP4Z1* gene, we performed rescue experiments and revealed that overexpression of CYP4Z1 indeed enhanced the TIC-like traits of 4T1 cells, which was also attenuated by BM-51 (Supplementary Fig. 15 and Fig. 7h, i). Notably, the mutants (V126P, R384M, A314G, and S383A) of CYP4Z1 could also increase the TIC-like traits of 4T1 cells, but the effects were not rescued by BM-51 treatment (Supplementary Fig. 15 and Fig. 7h, i). Notably, BM-51 held little effect on the stemness of 4T1 cells (Supplementary Fig. 15 and Fig. 7h, i). Thus, our results demonstrate that compound BM-51 can target CYP4Z1 at Arg384, Ser383, Val126, and Ala314 sites and can attenuate the TIC-like traits of breast cancer cells.

### Compound BM-51 suppresses the progression and the function of *PyMT*-induced TICs in *PyMT-CYP4Z1-KI* mice and chemosensitivity of breast cancer cells

**Fig. 8:**
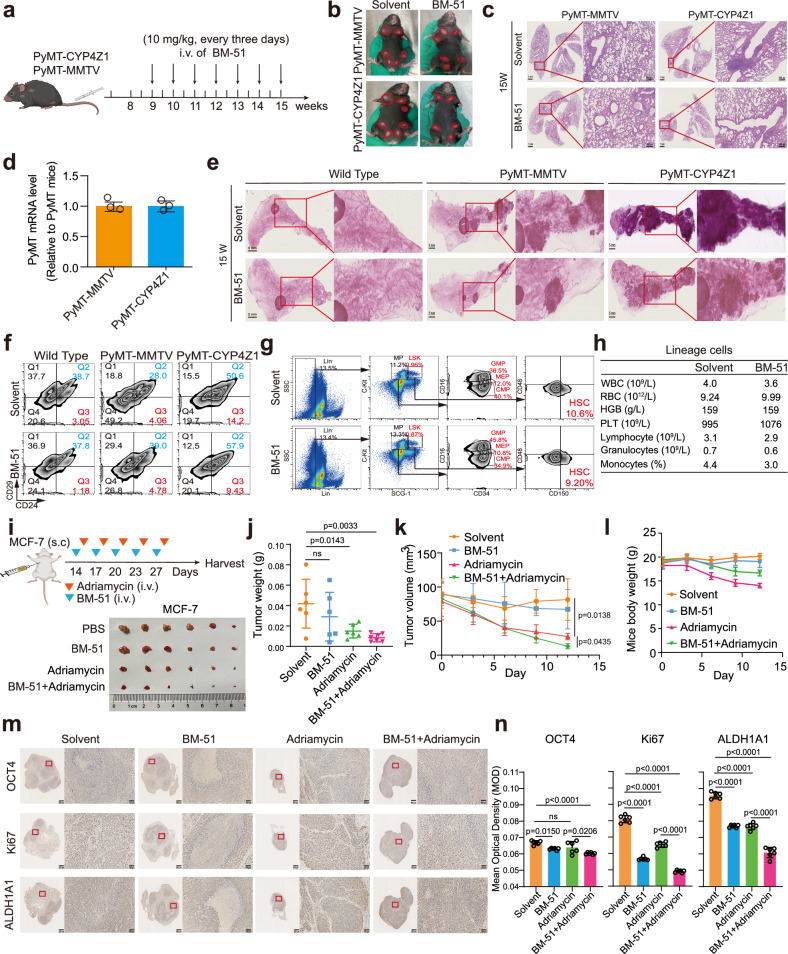
BM-51 can suppress the progression and the function of *PyMT*-induced TICs in *PyMT-CYP4Z1-KI* mice and chemosensitivity of breast cancer cells. **a** *PyMT-MMTV* and *PyMT-CYP4Z1* mice were treated with 10 mg/kg i.v. of BM-51 every 3 days, and i.v. of saline was used as the control group. **b** Tumors palpable in mice at the age of 15 weeks, with tumors formed in mice in the red circle. **c** Hematoxylin and eosin staining results of metastatic nodules in lung tissue at the age of 15 weeks in mice. Images were representative of images from three independent experiments. **d** Quantitative real-time-PCR of *PyMT* in tumor tissue derived from *PyMT-MMTV* and *PyMT-CYP4Z1* mice. Data were presented as mean ± SEM. *n* = 3 independent experiments. Two-sided Student’s *t* test. **e** Whole-mount carmine red staining of mammary glands from *WT*, *PyMT-MMTV*, and *PyMT-CYP4Z1* mice at 15 weeks of age. Images were representative of images from three independent experiments. **f** Flow cytometry shows the content of progenitor cells in the mammary cavity of mice after sorting. Images were representative of images from three independent experiments. **g** Representative flow cytometry profiles showing gating strategy and changes in various bone marrow HSC subsets. Images are representative of images from three or four independent experiments. **h** Peripheral blood and complete blood count of *PyMT-CYP4Z1* mice treated with BM-51 or not. **i** Images of MCF-7 cell tumors harvested with the treatment of saline, BM-51, Adriamycin, or BM-51 combined with Adriamycin. **j**, **k** Tumor weight and volume of part **i**. **l** Mice body weight of part **i**. **m**, **n** Immunohistochemical analysis was performed to detect the expression of stemness markers in tumors from part **i**. Data were presented as mean ± SEM, one-way analysis of variance test. ALDH, aldehyde dehydrogenase; CMP, common myeloid progenitor; GMP, granulocyte–macrophage progenitor; HSC, hematopoietic stem cell; i.v., intravenous; LSK, Lineage^−^/Sca-1^+^/c-Kit; MEP, megakaryocyte–erythroid progenitor; RBC, red blood cell; s.c., subcutaneous; WBC, white blood cell.

To further confirm BM-51 effects on breast cancer progression, we further treated *PyMT-MMTV* and *PyMT-CYP4Z1* mice with BM-51 (Fig. 8a). We compared the process of tumor progression between *PyMT-MMTV* and *PyMT-CYP4Z1* mice with or without BM-51 treatment. It was found that BM-51 had little effect on the initiation of *PyMT-MMTV* mice, whereas tumor initiation in *PyMT-CYP4Z1* mice was significantly delayed by BM-51 treatment with developing tumors (Fig. 8b). Compared with *PyMT-CYP4Z1* mice treated with solvent control, *PyMT-CYP4Z1* mice treated with BM-51 displayed significant reductions in tumor size and mount (Fig. 8b) and tumor volume and weight (Supplementary Fig. 16a,b). Additionally, smaller and fewer numbers of metastatic lung nodules were observed in BM-51-treated *PyMT-CYP4Z1* mice (Fig. 8c). Importantly, *PyMT* mRNA levels were unchanged in *PyMT-CYP4Z1* mice tumors treated with or without BM-51 (Fig. 8d). We then investigated BM-51-mediated effects on the expansion of *CYP4Z1*-induced TICs. Mammary gland whole-mounts prepared from *PyMT-CYP4Z1* mice treated with or without BM-51 were isolated at hyperplastic/pre-neoplastic stages of growth to determine the effect of BM-51 on tumor initiation. As shown in Fig. 8e and Supplementary Fig. 16c, by 15 weeks of age, glands from *PyMT-CYP4Z1* treated with BM-51 displayed markedly fewer and smaller hyperplastic lesions compared with the *PyMT-CYP4Z1* mice treated with solvent control, whereas BM-51 held no effect on mammary gland development of normal (wild-type) mice. As shown in Fig. 8f, *PyMT-CYP4Z1* treated with BM-51 expands fewer luminal progenitors (CD29^lo^CD24^+^) relative to *PyMT-CYP4Z1* mice treated with solvent control, whereas BM-51 had little effect in normal and *PyMT-MMTV* mice. By contrast, the CD29^+^CD24^+^ basal subpopulation was markedly increased in *PyMT-CYP4Z1* mice mammary glands treated with BM-51 relative to *PyMT-CYP4Z1* glands treated with solvent control (Fig. 8f and Supplementary Fig. 16d,e). Furthermore, we evaluated whether BM-51 could exert any detrimental effects on the functions and multilineage development of HSC. First, we analyzed the LSK and HSC compartments and various bone marrow progenitor cell subsets comprising granulocyte–macrophage progenitors, CMPs, and megakaryocyte–erythroid progenitors in normal mice treated with or without BM-51. As shown in Fig. 8g (also Supplementary Fig. 16f), except for CMP which was modestly reduced in BM-51-treated mice, the other bone marrow progenitor cell subsets were upregulated or barely changed. We also analyzed the complete blood count in peripheral blood (Fig. 8h) and found no notable changes in any differentiated lineage cells. Finally, we investigated the influence of BM-51 on the chemotherapeutic effects of Adriamycin. BM-51 exerted no obvious suppressive effect on tumor progression derived from breast cancer MCF-7 cells comparable to that of Adriamycin, but it enhanced the tumor-suppressive effects of Adriamycin, as evidenced by the change in tumor weight and volume (Fig. 8i–k). Notably, the constant mice weight indicated its low toxicity of BM-51 (Fig. 8l). Importantly, examining the stemness marker expression in tumors obtained a consistent result, indicating the suppression of BM-51 on stemness (Fig. 8m, n). These results indicate that BM-51 could suppress the progression and function of *PyMT*-induced TICs in *PyMT-CYP4Z1* mice.

## Discussion

These findings reveal a critical role of CYP4Z1 in the progression and metastasis based on the *PyMT*-induced mammary tumors and multi-omics analysis, highlighting its potential as a therapeutic target in breast cancer. Our data demonstrate that knock-in of *CYP4Z1* notably enhances the TIC population, tumor initiation, and size and increases the metastatic burden in *PyMT-CYP4Z1* mice compared with *PyMT-MMTV* controls. This suggests that CYP4Z1 promotes a more aggressive tumor phenotype, possibly by enhancing the TIC population.

The expression of CYP4Z1 is closely related to the malignant degree of tumor development and the degree of lymph node metastasis^43,44^. In addition, there was a shred of evidence that the expression of CYP4Z1 was highly correlated with the expression of Her2, Ki67, and p53 (ref. ^45^). This evidence further consolidates our results, showing the critical promoting roles of CYP4Z1 in breast tumor progression. The accelerated tumor development observed in *PyMT-CYP4Z1* mice indicates that CYP4Z1 has a role in the early stages of tumorigenesis. The increased number and size of hyperplastic lesions and hyperplastic foci in *PyMT-CYP4Z1* mammary glands further support this. The expansion of the CD29^lo^CD24^+^ luminal progenitor cell population, which has TIC properties, in *PyMT-CYP4Z1* mice suggests that CYP4Z1 promotes the expansion of cells with stem-like features, contributing to tumor initiation and growth. Additionally, CYP4Z1 also appears to maintain TIC function in established tumors, as evidenced by the increased tumor frequency and size in limiting dilution assays with *PyMT-CYP4Z1* MECs. The enhanced expression of CSC markers (ALDH1A1, Oct4, and Ki67) and the increased weight of tumors derived from *PyMT-CYP4Z1* MECs further corroborate the role of CYP4Z1 in sustaining TIC properties. Furthermore, *CYP4Z1* knock-in promotes a de-differentiated state. Given the role of CYP4Z1 in promoting de-differentiation, the observed decrease in tumor purity may be attributed to CYP4Z1-induced tumor cell de-differentiation and a higher fraction of CYP4Z1^+^ epithelial cells corresponding to a stronger de-differentiation effect (Fig. 2g, h). These findings were further confirmed through spatial transcriptomic analysis, which indicated that the high expression of *CYP4Z1* in cell trajectory might be responsible for the differentiation of *CYP4Z1*^*+*^ epithelial cells and the development of two respective cell differentiation tracks (Supplementary Fig. 3), suggesting that CYP4Z1 not only promotes the expansion of TICs but also maintains their functionality, which may contribute to tumor recurrence and resistance to therapy. Notably, the tumor border is the area where tumor cells directly contact and invade non-tumor cells and tissue, which is the most active area for tumor microenvironment infiltration and invasion^10^. The high abundance of *CYP4Z1*^*+*^ Epi cells in the tumor border might indicate the function of *CYP4Z1*^*+*^ Epi in tumor invasion and development (Supplementary Fig. 3h,i). Importantly, although a recent study has found that breast-specific knock-in of CYP4Z1 is not sufficient to induce the occurrence of breast cancer, it has been observed that breast-specific knock-in of CYP4Z1 can notably upregulate the expression levels of estrogen receptor α (ERα) and lead to a significant decrease in birth rate and body weight in mice^46^. Previous research has confirmed that ERα is essential for the expansion of breast TICs^47^, further supporting the notion that CYP4Z1 is a vital enhancer in the amplification and activity of breast TICs.

The PTM manners of CYP4Z1 protein had never been revealed before. The interaction between CYP4Z1 and ISG15 and the subsequent ISGylation of CYP4Z1 add another layer of complexity regulating the function of CYP4Z1. Currently, we found that ISGylation enhanced CYP4Z1 protein stability and activity, and the specific lysine residues K259, K279, and K502 were identified as crucial ISGylation sites, and their mutation notably attenuated the TIC-promoting effects of CYP4Z1, highlighting the importance of ISGylation in CYP4Z1 function. However, the regulation of this function seems to be different in MCF-7 and MDA-MB-231 cells, especially in subcellular localization. This might be due to the fact that MDA-MB-231 cells represent a more malignant tumor and the stemness of MDA-MB-231 cells is higher than that of MCF-7 cells, and there might be other factors impacting CYP4Z1 localization in breast cancer cells, which should be further explored in the future. In past researches, ISG15 was thought to have an essential role in antiviral immunity^48–50^. It is also found that ISG15/ISGylation seems to have a vital role in tumor development and tumor immunity^18,51,52^. Additionally, a study has shown that patients with breast cancer can produce autoantibodies against CYP4Z1 (ref. ^10^). However, whether the ISGylation of CYP4Z1 contributes to regulating tumor microenvironment and immunotherapy is still unclear. Interestingly, CYP4Z1 presents a clear nuclear localization, but the functional significance of this nuclear targeting remains unknown. This suggests that CYP4Z1 may have distinct yet unidentified functions depending on its subcellular localization, warranting further investigation.

Lipidomics analysis revealed that CYP4Z1 and ISG15 overexpression predominantly upregulates TAGs, suggesting a role for CYP4Z1 in metabolic reprogramming. The similarity in metabolic changes induced by CYP4Z1 and ISG15 supports the idea that ISGylation of CYP4Z1 may contribute to lipid metabolism alterations, which were known to support the energetic and biosynthetic needs of rapidly proliferating cancer cells. In our study, we found that CYP4Z1 can promote the formation of TAG. However, the mechanism of CYP4Z1 regulating TAG formation is unclear. Previous studies have indicated that the CYP4Z1-catalyzed fatty acid metabolite 20-hydroxyeicosatetraenoic acid was considered as a functional regulator downstream of CYP4Z1 (refs. ^53,54^). But unfortunately, we did not find this metabolite in our lipidomics, which warrants further investigation. Notably, although our current study focusses on TAG accumulation as a functional readout, multiple lines of evidence support the direct role of lipid droplets in modulating cancer stemness, for example, manipulating lipid droplet-mediated numb degradation confers colorectal cancer cell to acquire stem cell-like features^55^. A recent single-cell analysis revealed that lipid droplet-rich tumor cells exhibit a distinct stem-like transcriptional profile^56^, and pharmacological disruption of lipid droplet formation using C17O (a peroxisome proliferator-activated receptor α-agonist) notably reduced CSC frequency in breast cancer models^57^. These studies collectively establish lipid droplet accumulation as a critical mediator of CSC maintenance beyond merely serving as a biomarker of metabolic reprogramming. We, therefore, posit that CYP4Z1-induced TAG accumulation creates a metabolic environment that both sustains lipid droplet biogenesis and directly enhances stemness through these droplet-associated pathways.

The ability of CYP4Z1 to promote TIC expansion, maintain TIC function, enhance invasion, and reprogram metabolism underscores its potential as a therapeutic target. Here, we discovered a compound, BM-51, which exhibits improved drug-like properties, reduced toxicity, and higher specificity. Notably, the results from in silico binding, CETSA, and DARTS indicate that BM-51 binds to the Arg384, Ser383, Val126, and Ala314 sites of CYP4Z1, with the Val126 site possibly not being critical (Fig. 7). Structurally, the ISGylation sites (K259, K279, and K502) are distal from the BM-51-binding pocket, suggesting that BM-51 acts independently of the ISGylation machinery. This mechanistic distinction supports the use of BM-51 as a pharmacological tool to specifically probe CYP4Z1 catalytic activity in TIC regulation (Supplementary Fig. 17). Subsequent experiments, such as co-crystallization, should be conducted to verify the specific binding mode between BM-51 and CYP4Z1. Regardless, this compound provides a superior lead for the development of CYP4Z1 inhibitors, thereby offering a promising approach for targeting breast TICs in breast cancer therapy. Notably, although our study incorporated multiple breast cancer models (for example, ER^+^ MCF7, TNBC MDA-MB-231) and pan-subtype datasets (GSE225600, TCGA) to capture shared mechanisms, we recognize that the resultant biological heterogeneity (ER^+^, HER2-positive, TNBC) may obscure subtype-specific nuances. This broad approach was intentionally chosen to identify common therapeutic vulnerabilities, but future work should implement subtype-stratified analyses to disentangle hormone-receptor-driven pathways from basal-like metastatic programs for precise clinical translation.

In summary, CYP4Z1 promotes breast cancer progression by enhancing TIC-like traits, promoting a de-differentiated and invasive phenotype, and reprogramming lipid metabolism. The ISGylation of CYP4Z1 by ISG15 is crucial for its function, providing a potential target for therapeutic intervention. These findings highlight the multifaceted role of CYP4Z1 in breast cancer and its potential as a target for preventing tumor progression and metastasis.

### Study approval

The Ethics Committee approved all animal experiments conducted in this study for Animal Experimentation of China Pharmaceutical University (2021-10-017).

## Supplementary information

Supplementary Information

## Data Availability

All data were presented within the article, as well as supplementary online data.

## References

[1] Siegel, R. L., Miller, K. D., Wagle, N. S. & Jemal, A. Cancer statistics, 2023. *CA Cancer J Clin* **73**, 17–48 (2023). 36633525 10.3322/caac.21763

[2] Arnold, M. et al. Current and future burden of breast cancer: global statistics for 2020 and 2040. *Breast* **66**, 15–23 (2022). 36084384 10.1016/j.breast.2022.08.010 PMC9465273

[3] Yang, Y. et al. Emerging agents that target signaling pathways in cancer stem cells. *J Hematol Oncol* **13**, 60 (2020). 32456660 10.1186/s13045-020-00901-6 PMC7249421

[4] Yuan, S. et al. Ras drives malignancy through stem cell crosstalk with the microenvironment. *Nature* **612**, 555–563 (2022). 36450983 10.1038/s41586-022-05475-6 PMC9750880

[5] Al-Hajj, M., Wicha, M. S., Benito-Hernandez, A., Morrison, S. J. & Clarke, M. F. Prospective identification of tumorigenic breast cancer cells. *Proc. Natl Acad. Sci. USA* **100**, 3983–3988 (2003). 12629218 10.1073/pnas.0530291100 PMC153034

[6] Shibata, M. & Hoque, M. O. Targeting cancer stem cells: a strategy for effective eradication of cancer. *Cancers* **11**, 732 (2019). 31137841 10.3390/cancers11050732 PMC6562442

[7] Rieger, M. A. et al. Identification of a novel mammary-restricted cytochrome P450, CYP4Z1, with overexpression in breast carcinoma. *Cancer Res.* **64**, 2357–2364 (2004). 15059886 10.1158/0008-5472.can-03-0849

[8] Li, C. et al. The competing endogenous RNA network of CYP4Z1 and pseudogene CYP4Z2P exerts an anti-apoptotic function in breast cancer. *FEBS Lett.* **591**, 991–1000 (2017). 28236635 10.1002/1873-3468.12608

[9] Yang, X., Hutter, M., Goh, W. W. B. & Bureik, M. CYP4Z1 — a human cytochrome P450 enzyme that might hold the key to curing breast cancer. *Curr. Pharm. Des.* **23**, 2060–2064 (2017). 28176668 10.2174/1381612823666170207150156

[10] Nunna, V., Jalal, N. & Bureik, M. Anti-CYP4Z1 autoantibodies detected in breast cancer patients. *Cell Mol Immunol* **14**, 572–574 (2017). 28435160 10.1038/cmi.2017.21 PMC5518819

[11] Yu, W. et al. Increased expression of CYP4Z1 promotes tumor angiogenesis and growth in human breast cancer. *Toxicol. Appl. Pharmacol.* **264**, 73–83 (2012). 22841774 10.1016/j.taap.2012.07.019 PMC3439529

[12] Zheng, L. et al. Competing endogenous RNA networks of CYP4Z1 and pseudogene CYP4Z2P confer tamoxifen resistance in breast cancer. *Mol. Cell. Endocrinol.* **427**, 133–142 (2016). 26980484 10.1016/j.mce.2016.03.012

[13] Zheng, L., Li, X., Gu, Y., Lv, X. & Xi, T. The 3’UTR of the pseudogene CYP4Z2P promotes tumor angiogenesis in breast cancer by acting as a ceRNA for CYP4Z1. *Breast Cancer Res Treat* **150**, 105–118 (2015). 25701119 10.1007/s10549-015-3298-2

[14] Wilkinson, N. A., Mnuskin, K. S., Ashton, N. W. & Woodgate, R. Ubiquitin and ubiquitin-like proteins are essential regulators of DNA damage bypass. *Cancers* **12**, 2848 (2020). 33023096 10.3390/cancers12102848 PMC7600381

[15] Yuan, Y. et al. The functional roles of ISG15/ISGylation in cancer. *Molecules* **28**, 1337 (2023). 36771004 10.3390/molecules28031337 PMC9918931

[16] Sainz, B. Jr, Martín, B., Tatari, M., Heeschen, C. & Guerra, S. ISG15 is a critical microenvironmental factor for pancreatic cancer stem cells. *Cancer Res* **74**, 7309–7320 (2014). 25368022 10.1158/0008-5472.CAN-14-1354

[17] Alcalá, S. et al. ISG15 and ISGylation is required for pancreatic cancer stem cell mitophagy and metabolic plasticity. *Nat Commun* **11**, 2682 (2020). 32472071 10.1038/s41467-020-16395-2 PMC7260233

[18] Xu, T. et al. ISG15 and ISGylation modulates cancer stem cell-like characteristics in promoting tumor growth of anaplastic thyroid carcinoma. *J Exp Clin Cancer Res* **42**, 182 (2023). 37501099 10.1186/s13046-023-02751-9 PMC10373324

[19] Sun, J. et al. Loss of TRIM29 suppresses cancer stem cell-like characteristics of PDACs via accelerating ISG15 degradation. *Oncogene* **39**, 546–559 (2020). 31501523 10.1038/s41388-019-0992-2

[20] Zhang, Q. et al. ISG15 is downregulated by KLF12 and implicated in maintenance of cancer stem cell-like features in cisplatin-resistant ovarian cancer. *J Cell Mol Med* **25**, 4395–4407 (2021). 33797839 10.1111/jcmm.16503 PMC8093991

[21] Ali, H. R. et al. PD-L1 protein expression in breast cancer is rare, enriched in basal-like tumours and associated with infiltrating lymphocytes. *Ann Oncol* **26**, 1488–1493 (2015). 25897014 10.1093/annonc/mdv192

[22] Goldman, M. J. et al. Visualizing and interpreting cancer genomics data via the Xena platform. *Nat Biotechnol* **38**, 675–678 (2020). 32444850 10.1038/s41587-020-0546-8 PMC7386072

[23] Coutant, A. et al. Spatial transcriptomics reveal pitfalls and opportunities for the detection of rare high-plasticity breast cancer subtypes. *Lab Invest* **103**, 100258 (2023). 37813278 10.1016/j.labinv.2023.100258

[24] Hao, Y. et al. Integrated analysis of multimodal single-cell data. *Cell* **184**, 3573–3587.e29 (2021). 34062119 10.1016/j.cell.2021.04.048 PMC8238499

[25] Liu, Y.-M. et al. Combined single-cell and spatial transcriptomics reveal the metabolic evolvement of breast cancer during early dissemination. *Adv Sci (Weinh)* **10**, e2205395 (2023). 36594618 10.1002/advs.202205395 PMC9951304

[26] Hänzelmann, S., Castelo, R. & Guinney, J. GSVA: gene set variation analysis for microarray and RNA-seq data. *BMC Bioinform* **14**, 7 (2013). 10.1186/1471-2105-14-7 PMC3618321 23323831

[27] Becht, E. et al. Estimating the population abundance of tissue-infiltrating immune and stromal cell populations using gene expression. *Genome Biol* **17**, 218 (2016). 27908289 10.1186/s13059-016-1113-y PMC5134277

[28] Frede, J. et al. Dynamic transcriptional reprogramming leads to immunotherapeutic vulnerabilities in myeloma. *Nat. Cell Biol* **23**, 1199–1211 (2021). 34675390 10.1038/s41556-021-00766-y PMC8764878

[29] Trapnell, C., Cacchiarelli, D. & Qiu, X. Monocle: cell counting, differential expression, and trajectory analysis for single-cell RNA-Seq experiments. *Bioconductor* https://www.bioconductor.org/packages/release/bioc/html/monocle.html (2017).

[30] Jin, S. et al. Inference and analysis of cell-cell communication using CellChat. *Nat Commun* **12**, 1088 (2021). 33597522 10.1038/s41467-021-21246-9 PMC7889871

[31] Elosua-Bayes, M., Nieto, P., Mereu, E., Gut, I. & Heyn, H. SPOTlight: seeded NMF regression to deconvolute spatial transcriptomics spots with single-cell transcriptomes. *Nucleic Acids Res* **49**, e50 (2021). 33544846 10.1093/nar/gkab043 PMC8136778

[32] Yuan, Y. et al. Identification of a novel potent CYP4Z1 inhibitor attenuating the stemness of breast cancer cells through lead optimization. *J Med Chem* **65**, 15749–15769 (2022). 36414390 10.1021/acs.jmedchem.2c01320

[33] Hu, Y. & Smyth, G. K. ELDA: extreme limiting dilution analysis for comparing depleted and enriched populations in stem cell and other assays. *J Immunol Methods* **347**, 70–78 (2009). 19567251 10.1016/j.jim.2009.06.008

[34] Guy, C. T., Cardiff, R. D. & Muller, W. J. Induction of mammary tumors by expression of polyomavirus middle T oncogene: a transgenic mouse model for metastatic disease. *Mol Cell Biol* **12**, 954–961 (1992). 1312220 10.1128/mcb.12.3.954-961.1992 PMC369527

[35] Kouros-Mehr, H. et al. GATA-3 links tumor differentiation and dissemination in a luminal breast cancer model. *Cancer Cell* **13**, 141–152 (2008). 18242514 10.1016/j.ccr.2008.01.011 PMC2262951

[36] Wan, L. et al. MTDH–SND1 interaction is crucial for expansion and activity of tumor-initiating cells in diverse oncogene- and carcinogen-induced mammary tumors. *Cancer Cell* **26**, 92–105 (2014). 24981741 10.1016/j.ccr.2014.04.027 PMC4101059

[37] Malanchi, I. et al. Interactions between cancer stem cells and their niche govern metastatic colonization. *Nature* **481**, 85–89 (2011). 22158103 10.1038/nature10694

[38] Ni, T. et al. Snail1-dependent p53 repression regulates expansion and activity of tumour-initiating cells in breast cancer. *Nat Cell Biol* **18**, 1221–1232 (2016). 27749822 10.1038/ncb3425 PMC6038146

[39] Asselin-Labat, M. L. et al. Gata-3 is an essential regulator of mammary-gland morphogenesis and luminal-cell differentiation. *Nat Cell Biol* **9**, 201–209 (2007). 17187062 10.1038/ncb1530

[40] Liu, Y. M. et al. Combined single-cell and spatial transcriptomics reveal the metabolic evolvement of breast cancer during early dissemination. *Adv Sci (Weinh)* **10**, e2205395 (2023). 36594618 10.1002/advs.202205395 PMC9951304

[41] van Dierendonck, X. et al. Triglyceride breakdown from lipid droplets regulates the inflammatory response in macrophages. *Proc. Natl Acad. Sci. USA* **119**, e2114739119 (2022). 35302892 10.1073/pnas.2114739119 PMC8944848

[42] Qin, H. et al. 1-Benzylimidazole attenuates the stemness of breast cancer cells through partially targeting CYP4Z1. *Environ Toxicol* **39**, 1505–1520 (2024). 37994574 10.1002/tox.24050

[43] Al-Saraireh, Y. M. et al. Profiling of CYP4Z1 and CYP1B1 expression in bladder cancers. *Sci Rep* **11**, 5581 (2021). 33692504 10.1038/s41598-021-85188-4 PMC7946900

[44] Majumder, A. et al. Enhanced expression of histone chaperone APLF associate with breast cancer. *Mol Cancer* **17**, 76 (2018). 29580241 10.1186/s12943-018-0826-9 PMC5870250

[45] Al-Saraireh, Y. M. et al. Screening of cytochrome 4Z1 expression in human non-neoplastic, pre-neoplastic and neoplastic tissues. *Ecancermedicalscience* **14**, 1114 (2020). 33144882 10.3332/ecancer.2020.1114 PMC7581338

[46] Khayeka-Wandabwa, C., Zhao, J., Pathak, J. L., Wu, H. & Bureik, M. Upregulation of estrogen receptor alpha (ERα) expression in transgenic mice expressing human CYP4Z1. *Breast Cancer Res Treat* **191**, 319–326 (2022). 34725776 10.1007/s10549-021-06435-w

[47] Britschgi, A. et al. The Hippo kinases LATS1 and 2 control human breast cell fate via crosstalk with ERα. *Nature* **541**, 541–545 (2017). 28068668 10.1038/nature20829 PMC6726477

[48] Sarkar, L., Liu, G. & Gack, M. U. ISG15: its roles in SARS-CoV-2 and other viral infections. *Trends Microbiol* **31**, 1262–1275 (2023). 37573184 10.1016/j.tim.2023.07.006 PMC10840963

[49] Lin, C. et al. Regulation of STING activity in DNA sensing by ISG15 modification. *Cell Rep* **42**, 113277 (2023). 37864791 10.1016/j.celrep.2023.113277

[50] Perng, Y. C. & Lenschow, D. J. ISG15 in antiviral immunity and beyond. *Nat Rev Microbiol* **16**, 423–439 (2018). 29769653 10.1038/s41579-018-0020-5 PMC7097117

[51] Qu, T. et al. ISG15 targets glycosylated PD-L1 and promotes its degradation to enhance antitumor immune effects in lung adenocarcinoma. *J Transl Med*. **21**, 341 (2023). 37217923 10.1186/s12967-023-04135-1 PMC10204161

[52] Nguyen, H. M. et al. Interferon stimulated gene 15 (ISG15) in cancer: an update. *Cancer Lett* **556**, 216080 (2023). 36736853 10.1016/j.canlet.2023.216080

[53] Wu, W. et al. Drivers and suppressors of triple-negative breast cancer. *Proc Natl Acad Sci USA* **118**, e2104162118 (2021). 34389675 10.1073/pnas.2104162118 PMC8379974

[54] Jarrar, Y. B. & Lee, S. J. Molecular functionality of cytochrome P450 4 (CYP4) genetic polymorphisms and their clinical implications. *Int J Mol Sci* **20**, 4274 (2019). 31480463 10.3390/ijms20174274 PMC6747359

[55] Liu, H. et al. *Fusobacterium nucleatum* promotes colorectal cancer cell to acquire stem cell-like features by manipulating lipid droplet-mediated numb degradation. *Adv Sci (Weinh)* **9**, e2105222 (2022). 35170250 10.1002/advs.202105222 PMC9035998

[56] Hershey, B. J., Vazzana, R., Joppi, D. L. & Havas, K. M. Lipid droplets define a sub-population of breast cancer stem cells. *J Clin Med* **9**, 87 (2019). 31905780 10.3390/jcm9010087 PMC7019257

[57] Kuramoto, K. et al. Inhibition of the lipid droplet-peroxisome proliferator-activated receptor α axis suppresses cancer stem cell properties. *Genes* **12**, 99 (2021). 33466690 10.3390/genes12010099 PMC7828779

