# Supplementary material for: ISGylation-mediated stabilization of CYP4Z1 fuels breast cancer initiation and progression
Source: Exp Mol Med. 2026 Jul 2;58(7):2202–22. doi: 10.1038/s12276-026-01766-4 (PMC13434766; doi:10.1038/s12276-026-01766-4)
Supplement: Supplementary file 1 [file 12276_2026_1766_MOESM1_ESM.pdf]

Supplementary Table 1. The canonical markers for cell annotation

Markers	Celltype
CD19, CD79A, MS4A1	B cells
PECAM1, VWF	Endothelial cells
EPCAM, KRT19, PROM1	Epithelial cells
COL1A1, COL1A2, DCN	Fibroblast
CD68, CD163, CSF1R, MRC1, TPSAB1, MS4A2, CD1C	Myeloid cells
NCR1, GNLY, NKG7	NK cells
IGHG1, MZB1, SDC1	Plasma
CD3D, CD3E, CD8A, CD4	T cells
MYLK, ACTA2, PDGFRB, MYH11	Smooth muscle cells

Supplementary Table 2. The detailed information about the used plasmids.

REAGENT or RESOURCE	SOURCE	IDENTIFIER
p-3×Flag-CMV-10	Sigma	E7658
p-3×Flag-CMV-10-CYP4Z1	This paper	N/A
p-3×Flag-CMV-10-USP18	This paper	N/A
p-3×Flag-CMV-10-ISG15	This paper	N/A
pcDNA4/hismaxB	Thermo Fisher	V86420
pcDNA4/hismaxB-CYP4Z1	This paper	N/A
pcDNA4/hismaxB-ISG15	This paper	N/A
pcDNA3.1-HA	Addgene	128034
pcDNA3.1-HA-ISG15	This paper	N/A
p-3×Flag-CMV-10-CYP4Z1 K259R	This paper	N/A
p-3×Flag-CMV-10-CYP4Z1 K248R	This paper	N/A
p-3×Flag-CMV-10-CYP4Z1 K309R	This paper	N/A
p-3×Flag-CMV-10-CYP4Z1 K502R	This paper	N/A
p-3×Flag-CMV-10-CYP4Z1 K279R	This paper	N/A
p-3×Flag-CMV-10-CYP4Z1 K220R	This paper	N/A
p-3×Flag-CMV-10-CYP4Z1 K248RK259RK279RK309RK502R	This paper	N/A
p-3×Flag-CMV-10-CYP4Z1 K220RK259RK279RK309RK502R	This paper	N/A
p-3×Flag-CMV-10-CYP4Z1 K220RK248RK279RK309RK502R	This paper	N/A
p-3×Flag-CMV-10-CYP4Z1 K220RK248RK259RK309RK502R	This paper	N/A
p-3×Flag-CMV-10-CYP4Z1 K220RK248RK259RK279RK502R	This paper	N/A
p-3×Flag-CMV-10-CYP4Z1 K220RK248RK259RK279RK309R	This paper	N/A
p-3×Flag-CMV-10-CYP4Z1 K220RK248RK259RK279RK309RK502R	This paper	N/A

Supplementary Table 3. The detailed information about the chemicals, peptides, and recombinant proteins used in this work.

REAGENT or RESOURCE	SOURCE	IDENTIFIER
EGF	MCE	CAT#HY-P7109
Hydrocortisone	MCE	CAT#HY-N0583
Insulin	Yeasen Biotechnology (Shanghai, China)	CAT#40112ES25
Collagenase	Sigma	C8051
Hyaluronidase	Sigma	H1136
Dispase II	Yeasen Biotechnology (Shanghai, China)	CAT#40104ES60
DNase I	Yeasen Biotechnology (Shanghai, China)	CAT#10608ES25
bFGF	Yeasen Biotechnology (Shanghai, China)	CAT#91315ES10
A922500	Sigma	A1737
capric triglyceride	Sigma	T7517
DAPI	Beyotime	C1006

Supplementary Table 4. Sequences of siRNA against specific target in this study.

CYP4Z1 siRNA	Sense (5'-3')	CAUUACCUUUCCAGAUGGAdTdT
	Anti-Sense (5'-3')	UCCAUCUGGAAAGGUAAUGdTdT
si-ISG15-1	Sense (5'-3')	GCGCAGAUCACCCAGAAGAdTdT
	Anti-Sense (5'-3')	UCUUCUGGGUGAUCUGCGCdTdT
si-ISG15-2	Sense (5'-3')	CUGAGCAUCCUGGUGAGGAdTdT
	Anti-Sense (5'-3')	UCCUCACCAGGAUGCUCAGdTdT
si-ISG15-3	Sense (5'-3')	GUGGUGGACAAAUGCGACGdTdT
	Anti-Sense (5'-3')	CGUCGCAUUUGUCCACCACdTdT
UBCH8 siRNA	Sense (5'-3')	CGUGAAAGUGUUCAGCAAGdTdT
	Anti-Sense (5'-3')	CUUGCUGAACACUUUCACGdTdT
si-NC	Sense (5'-3')	UUCUCCGAACGUGUCACGUdTdT
	Anti-Sense (5'-3')	ACGUGACACGUUCGGAGAAdTdT

Supplementary Table 5. Sequences of PCR primers used in this study.

Name		Sequences
pcDNA™4/HisMax B-CYP4Z1	Forward (5'-3')	CGATAAGGTACCTAAGGATCCAATGGAGCCCTCCTGGCTTC
	Reserve (5'-3')	AACGGGCCCTCTAGACTCGAGTTAGCAAACTTTTTTTGCAAACACA
p3×FLAG-CMV-10-CYP4Z1	Forward (5'-3')	CAAGCTTGCGGCCGCGAATTCAATGGAGCCCTCCTGGCTTC
	Reserve (5'-3')	CAGGGATGCCACCCGGGATCCTTAGCAAACTTTTTTTGCAAACACA
pcDNA™4/HisMax B-ISG15	Forward (5'-3')	CGATAAGGTACCTAAGGATCCAATGGGCTGGGACCTGACG
	Reserve (5'-3')	AACGGGCCCTCTAGACTCGAGTTAGCTCCGCCCGCCAGG
pcDNA3.1+ N-HA-ISG15	Forward (5'-3')	ACTACGCCGGATCCGGAATTCATATGGGCTGGGACCTGACG
	Reserve (5'-3')	CCCTCTAGATGCATGCTCGAGTTAGCTCCGCCCGCCAGG
p3×FLAG-CMV-10-USP18	Forward (5'-3')	CAAGCTTGCGGCCGCGAATTCTATGAGCAAGGCGTTTGGGC
	Reserve (5'-3')	CAGGGATGCCACCCGGGATCCTTAGCACTCCATCTTCATGTAAACCA
CYP4Z1-K259R	Forward (5'-3')	TCACAGAGCGCGTAATCCAGGACCGGAAGGAG
	Reserve (5'-3')	GATTACGCGCTCTGTGAACTGATGAAGTTCTTGG
CYP4Z1-K309R	Forward (5'-3')	TGAAGTGCGCACGTTCATGTTTGCAGGACATG
	Reserve (5'-3')	TGAACGTGCGCACTTCAGCCTGGAGATCTGCT
CYP4Z1-K279R	Forward (5'-3')	ATACTACTCAGCGCAGGCGCTGGGATTTTCTGG
	Reserve (5'-3')	CCTGCGCTGAGTAGTATCTTGTTTTAGCTTATCCTTA
CYP4Z1-K220R	Forward (5'-3')	CTTAGCCGCATCTCCAACCAGCGCATGAACAA
	Reserve (5'-3')	TTGGAGATGCGGCTAAGGTTGAACACTGCTTTCAG
CYP4Z1-K502R	Forward (5'-3')	GTGTTTGCACGCAAAGTTTGCTAAGAATTCATCGATAGA
	Reserve (5'-3')	ACTTTGCGTGCAAACACATGGATTCCATTCTT
CYP4Z1-K248R	Forward (5'-3')	GCCAAATCTTTTCTCGCTTTAACCAAGAACTTCATCAGTTCACA
	Reserve (5'-3')	GCGAGAAAAGATTTGGCCTTGAGAGCTGAATT

Supplementary Table 6. The detailed information about the antibodies used in this work.

REAGENT or RESOURCE	SOURCE	IDENTIFIER
Cytochrome P450 4Z1/2 Antibody	Affinity	DF10128
GAPDH Monoclonal antibody	Proteintech	Cat No : 60004-1-Ig
DYKDDDDK tag Monoclonal antibody (Binds to FLAG® tag epitope)	Proteintech	Cat No : 66008-4-Ig
DYKDDDDK tag Recombinant antibody (Binds to FLAG® tag epitope)	Proteintech	Cat No : 80010-1-RR
6*His, His-Tag Monoclonal antibody	Proteintech	Cat No : 66005-1-Ig
HA Tag Monoclonal antibody	Proteintech	Cat No : 66006-2-Ig
HA-Tag Antibody (F-7)	Santa Cruz Biotechnology	sc-7392
ISG15 Polyclonal antibody	Proteintech	Cat No : 15981-1-AP
SOX2	Wanleibio	WL00982a
OCT3/4	Wanleibio	WL01728
ALDH1A1 Polyclonal antibody	Proteintech	Cat No : 15910-1-AP
NANOG Polyclonal antibody	Proteintech	Cat No : 14295-1-AP
UBE2E2 Polyclonal antibody	Proteintech	Cat No : 11844-1-AP
USP18 (D4E7) Rabbit mAb	Cell Signaling Technology (CST)	CAT#4813
KLF4	Wanleibio	WL02532
CoraLite® Plus 488-conjugated PSMD5 Monoclonal antibody	Proteintech	Cat No. CL488-67682
APC anti-mouse CD117 (c-Kit) Antibody	Biolegend	Cat#155107
PE/Cyanine7 anti-mouse CD34 Antibody	Biolegend	Cat#128618
PerCP/Cyanine5.5 anti-mouse CD48 Antibody	Biolegend	Cat#103421
PE anti-mouse Ly-6A/E (Sca-1) Antibody	Biolegend	Cat#163803
Brilliant Violet 421™ anti-mouse CD150 (SLAM) Antibody	Biolegend	Cat#115925
Brilliant Violet 510™ anti-mouse CD16/32 Antibody	Biolegend	Cat#156625
FITC anti-mouse CD3ε Antibody	Biolegend	Cat#100305
FITC anti-mouse CD127 (IL-7Rα) Antibody	Biolegend	Cat#159905

FITC anti-mouse IgM Antibody	Biolegend	Cat#406505
FITC anti-mouse CD19 Antibody	Biolegend	Cat#152403
FITC anti-mouse/human CD45R/B220 Antibody	Biolegend	Cat#103205
FITC anti-mouse TER-119/Erythroid Cells Antibody	Biolegend	Cat#116205
FITC anti-mouse Ly-6G/Ly-6C (Gr-1) Antibody	Biolegend	Cat#108405
CD44-APC	Biolegend	Cat#338806
CD24-PE	Biolegend	Cat#311106
CD24-APC	BD Biosciences	Cat#562349
CD29-PE	BD Biosciences	Cat#562801

Supplementary Table 7. The detailed information of qRT-PCR primers used in this work.

Name		Sequences
GAPDH-M	Forward (5'-3')	GAGCTGAACGGGAAGCTCAC
	Reserve (5'-3')	AGTGTAGCCCAAGATGCCCT
OCT-M	Forward (5'-3')	GGGCTCTGTGGTCAAGTCCG
	Reserve (5'-3')	CGCTCTGGTAGTGCTGGGC
SOX2-M	Forward (5'-3')	CAAGTTGGCGTGGAGACTTTGC
	Reserve (5'-3')	CCCCAAGGTGATCCTCTTCTGC
CYP4Z1-M	Forward (5'-3')	CTTTCCAGATGGACGCTCCTTACCT
	Reserve (5'-3')	GGCAAAATGCTGCCCAATGCAGTTC
ALDH1-M	Forward (5'-3')	CCCGGATTTTTGTTGAGGAG
	Reserve (5'-3')	GAGAACACTGTGGGCTGCAC
Csn1s1-M	Forward (5'-3')	CCAGTGCACCCTGGAACAGCT
	Reserve (5'-3')	GGGAAGCTTGCTGTACCAGGG
Csn1s2a-M	Forward (5'-3')	GCACACCTCTTCACCAGCAGC
	Reserve (5'-3')	GCTGATGGATTCCTGGGTGGG
Csn2-M	Forward (5'-3')	CCTTGTGGCCCTTGCTCTTGC
	Reserve (5'-3')	GGCTGTGACTGGATGCTGGAG

Supplementary Table 8. The detailed information about the probes used in this work.

REAGENT or RESOURCE	SOURCE	IDENTIFIER
MitoTracker™ Deep Red FM	ThermoFisher	M22426
LysoTracker™ Deep Red	ThermoFisher	L12492
ER-Tracker™ Green (BODIPY® FL Glibenclamide)	Cell Signaling Technology (CST)	CAT#8787
CellLINK 505/555	tingoscience	CL012100210

Supplementary Table 9. Structure of modified 1-benzylimidazole analogs and their CYP4Z1 inhibitory activity.

Cmpd.	Structure	Inhibition rate %[a] (10 μM)	Inhibition rate % (1 μM)	Inhibition rate % (0.1 μM)	IC_{50} (nM)
**1-Ben**	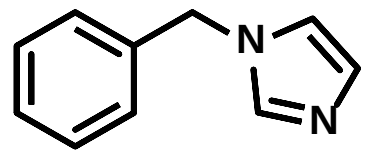	96.5	87.1	33.6	279.8±97.0
**BM-1**	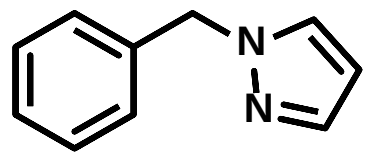	4.1	1.4	-0.7	/ [b]
**BM-2**	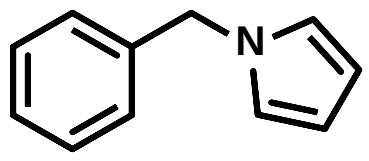	-3.0	9.8	-0.8	/
**BM-3**	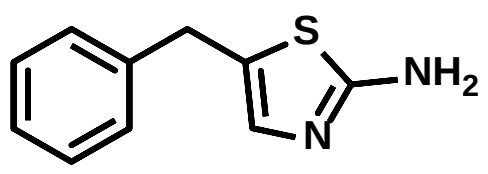	36.9	8.6	4.2	/
**BM-4**	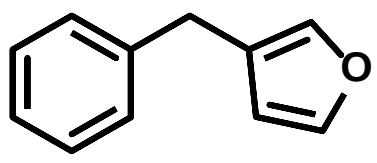	15.8	/	/	/
**BM-5**	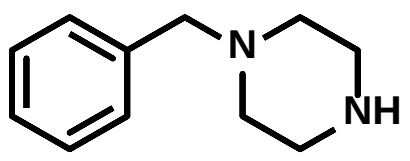	14.29	/	/	/
**BM-6**	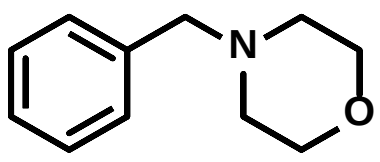	2.4	-1.3	-2.2	/
**BM-7**	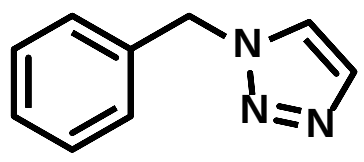	20.3	-2.6	-3.5	/
**BM-8**	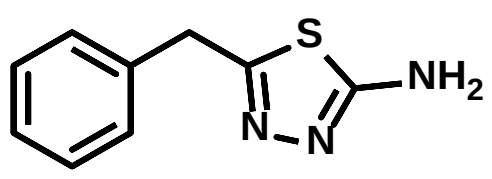	15.1	-10.5	-4.3	/
**BM-9**	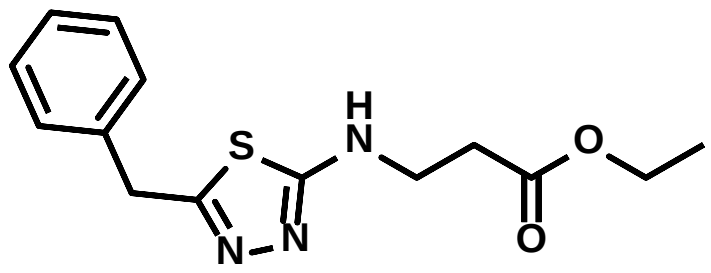	17.9	-4.7	-11.5	/
**BM-10**	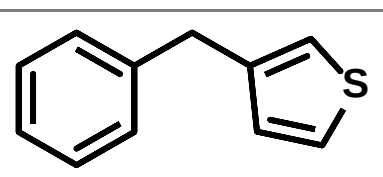	7.8	3.5	6.9	/
**BM-11**	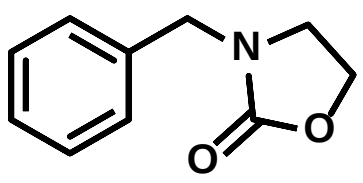	14.6	9.0	22.5	/

**BM-12**	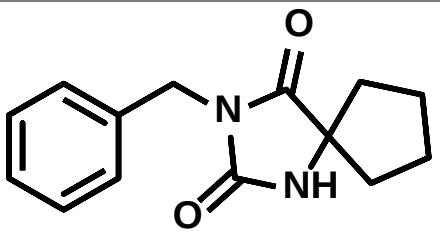	18.7	49.3	45.4	/
**BM-13**	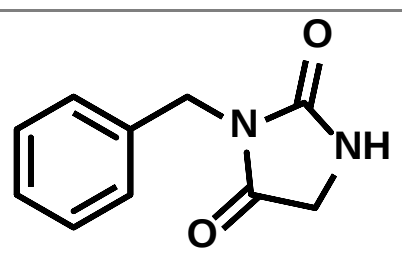	13.0	5.9	20.2	/
**BM-14**	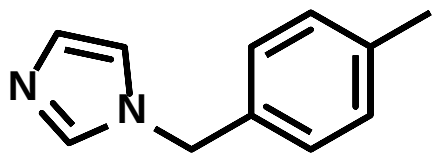	95.2	82.9	43.7	/
**BM-15**	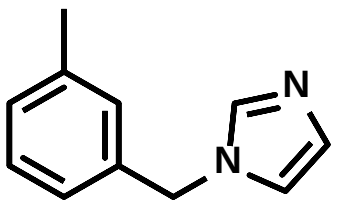	91.7	68.5	45.8	/
**BM-16**	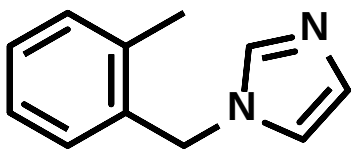	84.8	67.2	46.8	/
**BM-17**	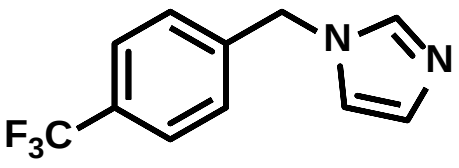	52.8	25.3	13.7	/
**BM-18**	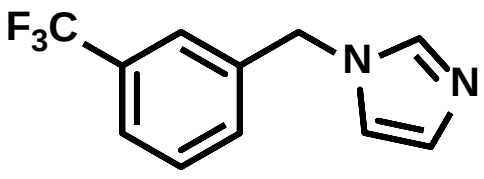	48.4	35.9	12.1	/
**BM-19**	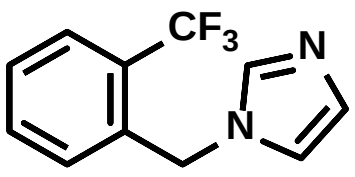	86.7	61.4	14.8	/
**BM-20**	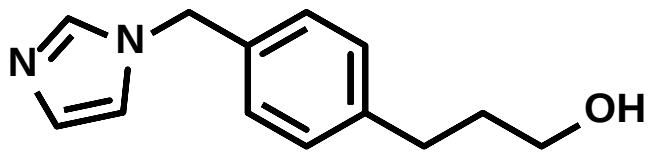	64.4	68.0	30.8	/
**BM-21**	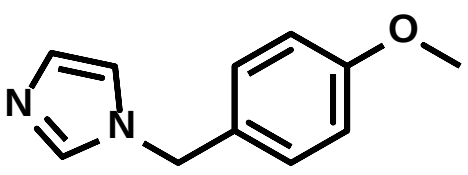	94.4	82.0	44.4	/
**BM-22**	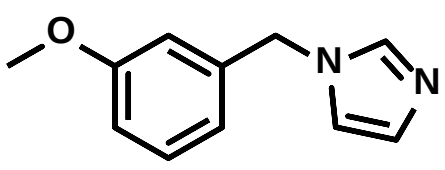	84.2	73.3	43.9	/
**BM-23**	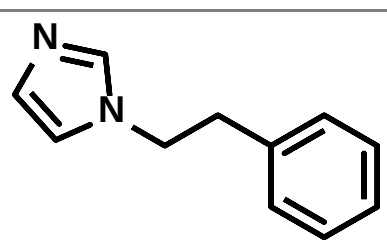	87.5	79.1	49.8	/
**BM-24**	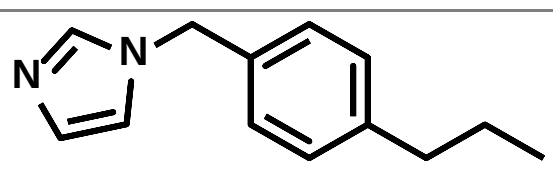	97.1	85.3	56.5	225.6±13.5

**BM-25**	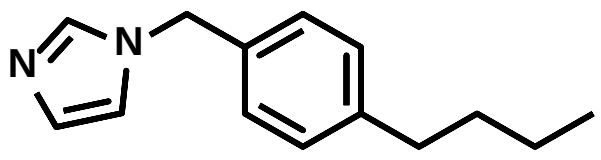	94.6	87.4	54.1	299.2±72.0
**BM-26**	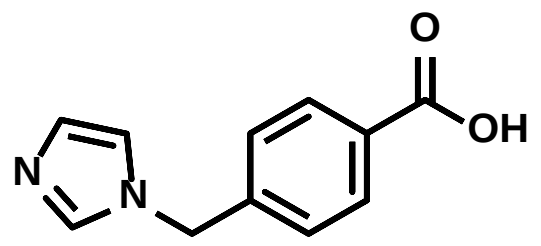	88.6	64.3	10.1	/
**BM-27**	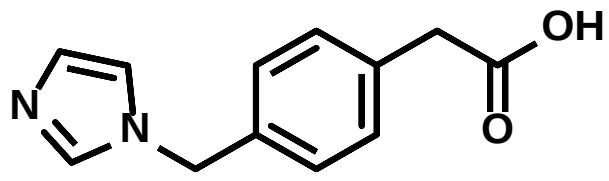	93.5	80.4	36.6	/
**BM-28**	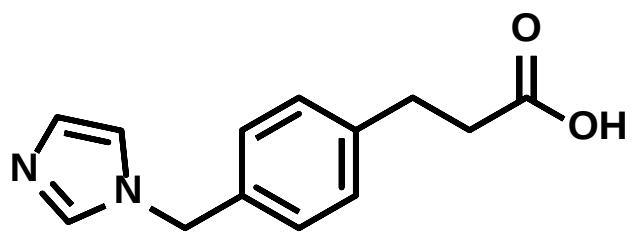	91.0	85.4	51.3	395.5±36.3
**BM-29**	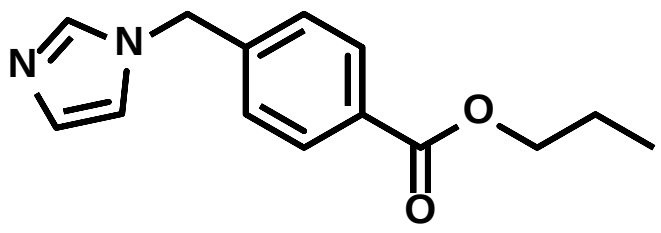	89.0	76.3	30.1	/
**BM-30**	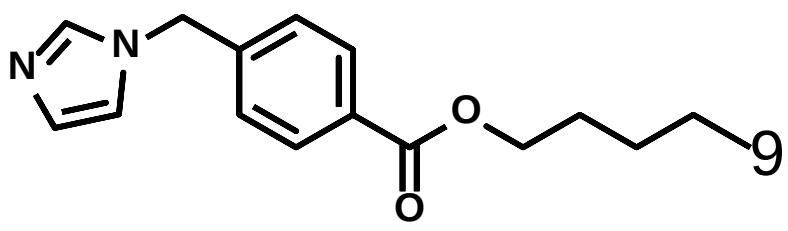	95.9	94.8	80.6	125.5±12.9
**BM-31**	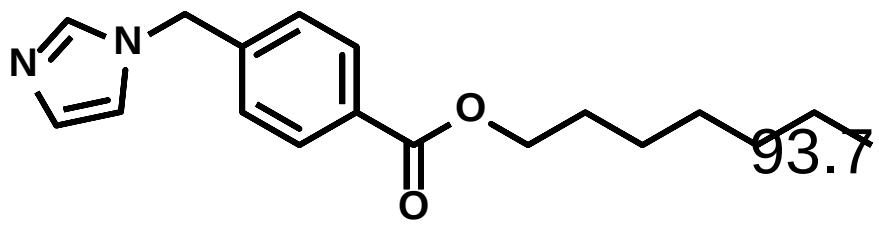	93.7	58.0	58.9	192.5±77.0
**BM-32**	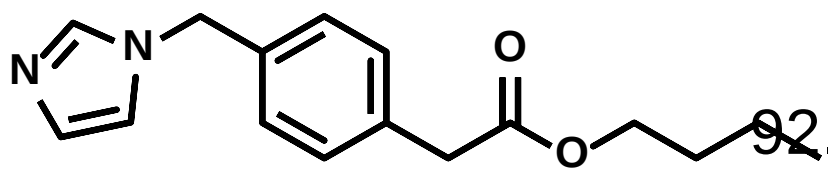	92.1	98.3	93.0	36.9±4.6
**BM-33**	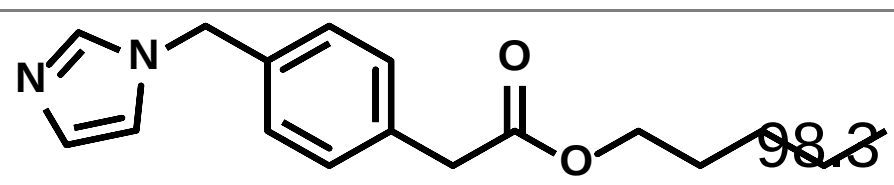	98.3	95.1	82.3	68.5±10.0
**BM-34**	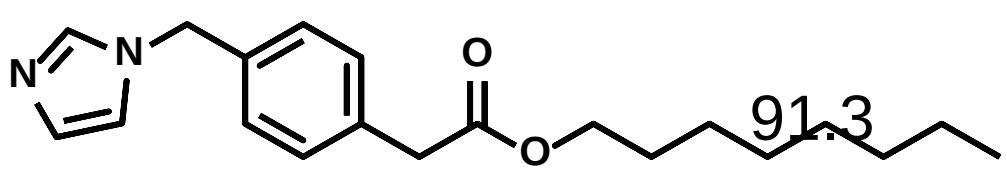	91.3	62.9	31.7	/
**BM-35**	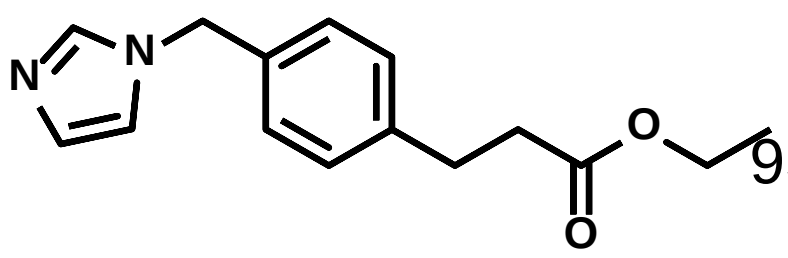	93.4	93.7	69.5	172.2±41.3
**BM-36**	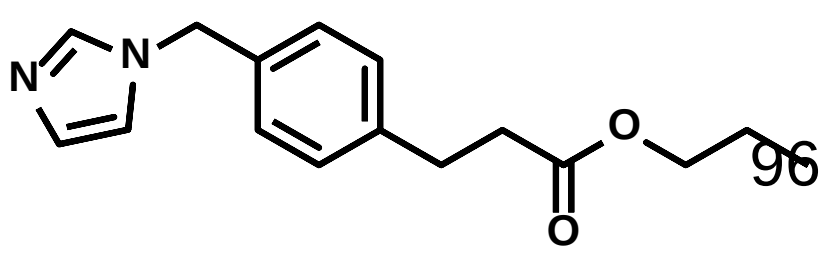	96.3	93.5	84.2	100.9±14.2

**BM-37**	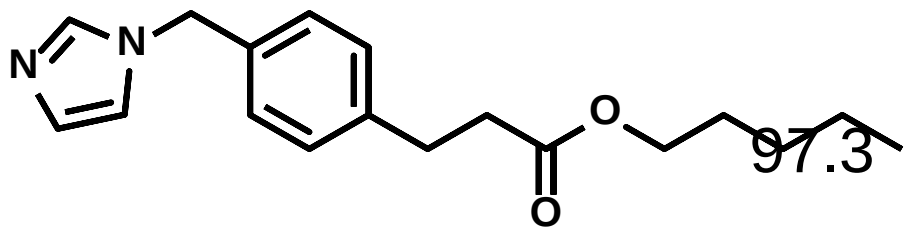	97.3	91.1	65.6	125.1±8.2
**BM-38**	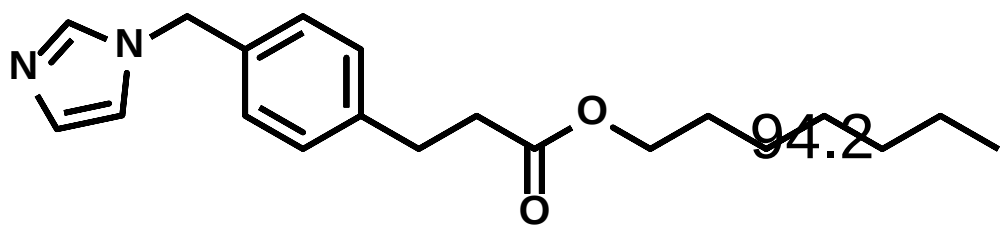	94.2	78.2	61.5	110.8±3.3
**BM-39**	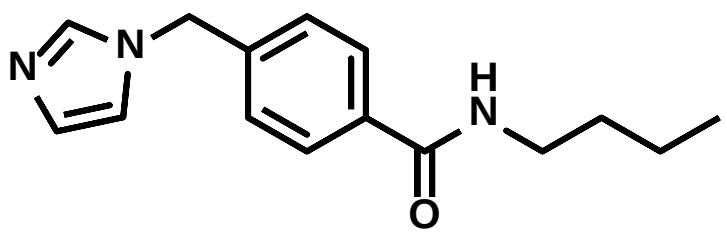	93.4	84.0	37.7	/
**BM-40**	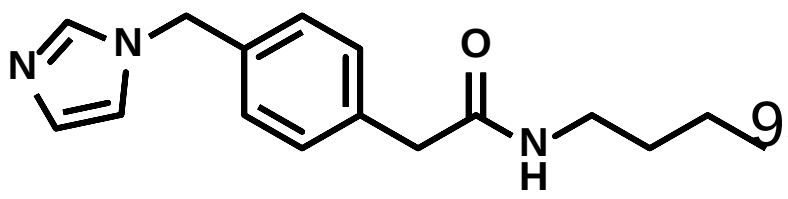	95.2	87.7	76.4	155.9±13.4
**BM-41**	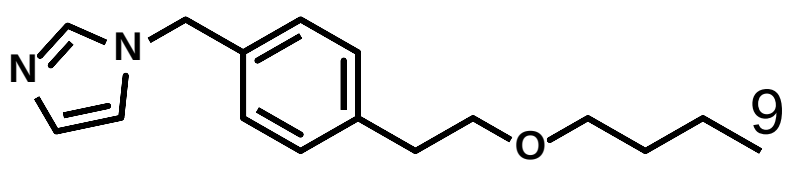	97.7	93.3	73.9	119.0±18.7
**BM-42**	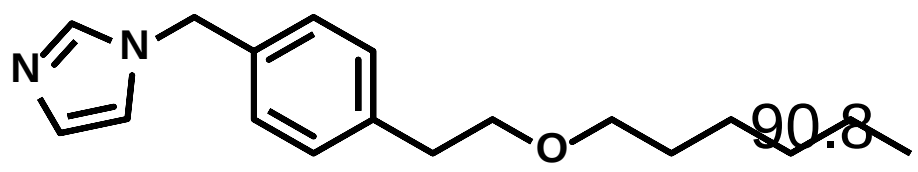	90.8	90.8	69.5	121.0±27.7
**BM-43**	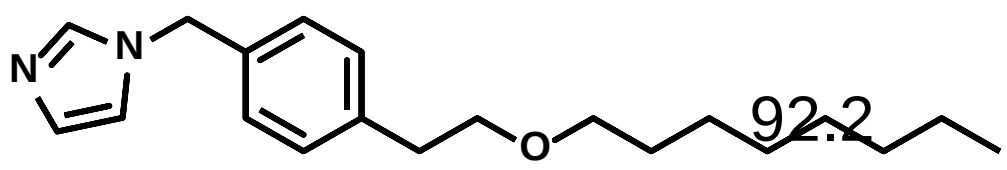	92.2	73.8	31.2	/
**BM-44**	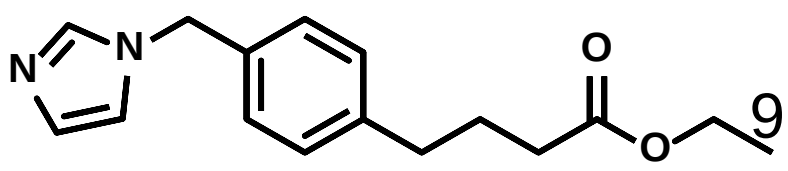	96.6	89.5	58.0	124.2±12.8
**BM-45**	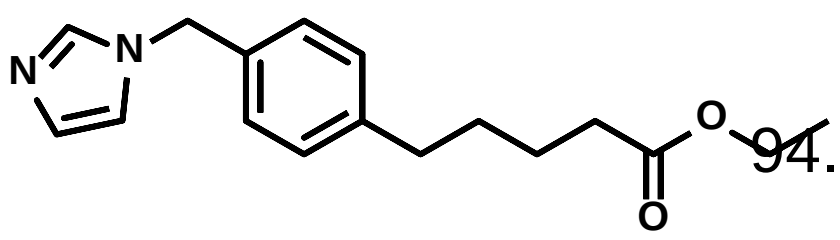	94.0	85.2	43.3	/
**BM-46**	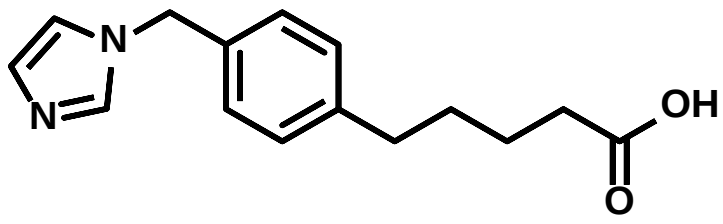	68.8	25.0	46.5	/
**BM-47**	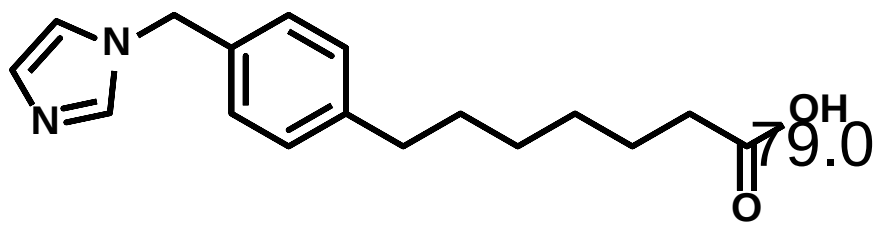	79.0	44.8	34.4	/

**BM-48**	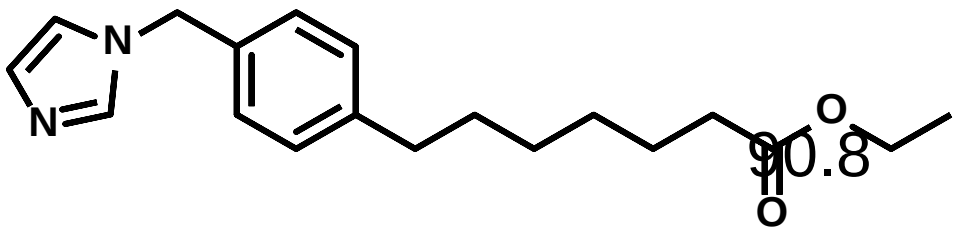	90.8	65.9	20.9	/
**BM-49**	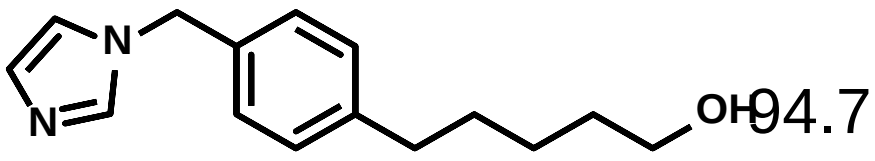	94.7	74.1	34.9	/
**BM-50**	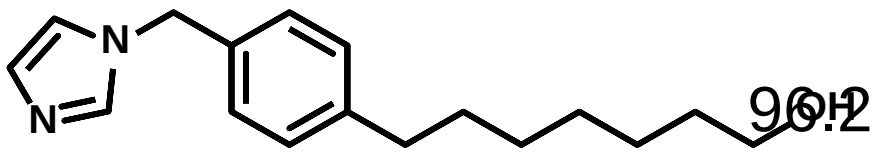	96.2	91.9	74.4	128.3±24.0
**BM-51**	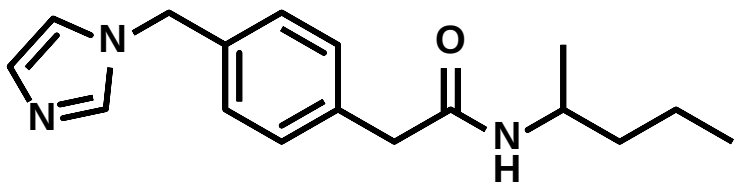	91.6	62.5	73.6	91.7±19.0
**BM-52**	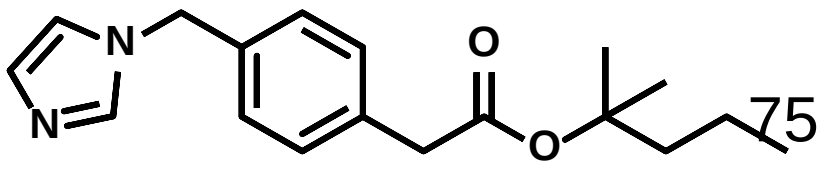	75.0	70.1	33.7	/
**BM-53**	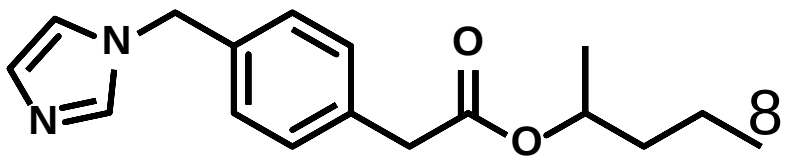	86.6	60.6	63.1	158.1±27.2

[a] Values are presented as the mean of two independent experiments.
[b] Not determined.

Supplementary Table 10. IC_{50} values of selected compounds for inhibition of CYP4F11, CYP4F8 and CYP3A4.

Compd.	IC_{50} (nM)		
	CYP4F11	CYP4F8	CYP3A4
**1-Ben**	>10000	>10000	>10000
**BM-24**	5532	/	/
**BM-25**	5532	/	/
**BM-28**	>10000	>10000	>10000
**BM-30**	6179	/	/
**BM-31**	>10000	>10000	>10000
**BM-32**	4781	/	/
**BM-33**	>10000	>10000	1517
**BM-35**	>10000	>10000	>10000
**BM-36**	4715	/	/
**BM-37**	4426	/	/
**BM-38**	>10000	>10000	2691
**BM-40**	>10000	>10000	>10000
**BM-41**	2705	/	/
**BM-42**	2382	/	/
**BM-44**	6141	/	/
**BM-50**	3122	/	/
**BM-51**	>10000	>10000	>10000
**BM-53**	598	/	/

Supplementary Table 11. IC_{50} values for inhibition of hepatic CYP-mediated substrate cocktail metabolism by BM-51

CYPs	CYP2B6	CYP2C9	CYP2C19	CYP2C8	CYP3A4
IC_{50} (μM)	5.53	0.98	0.709	1.46	12.65

Supplementary Table 12. Plasma stability of selectivity compound.

Compd. Species	Mouse (CD-1)			
	Parent remaining at the highest incubation time 3h (%)	$T_{1/2}$ (h)	Parent remaining at the highest incubation time 3h (%)	$T_{1/2}$ (h)
**1-Ben**	126	>3.0	76	>3.0
**BM-28**	106	>3.0	/	/
**BM-31**	0.02	0.05	/	/
**BM-35**	0.00	<0.05	/	/
**BM-40**	114	>3.0	/	/
**BM-51**	109	>3.0	101	>3.0
**Bisacodyl**	0.06	<0.5	13	NA

Supplementary Table 13. Pharmacokinetics parameters of BM-51 after p.o (10 mg/kg) and i.v. (1 mg/kg) administration in mouse.

Route	Animal No.	$T_{1/2}$ (h)	C_{max} (ng/mL)	AUC_{last} (h*ng/mL)	AUC_{INF_obs} (h*ng/mL)	CL_obs (mL/min/kg)	Vss_obs (L/kg)	F (%)
i.v.	1	0.52	-	170.67	175.41	121.0	9.17	96.5
	2	0.51	-	167.12	171.15	134.2	9.14	
	3	0.58	-	189.20	195.79	84.0	7.49	
	Mean	0.54	-	175.7	180.8	113.1	8.06	
	SD	0.04	-	11.86	11.17	26.05	0.96	
p.o.	1	0.44	1544.5	1916.8	1922.1	86.71	-	
	2	0.38	1520.4	1481.7	1482.9	112.4	-	
	3	0.47	1648.0	1688.1	1693.9	98.39	-	
	Mean	0.43	1571.0	1695.5	1699.7	99.16	-	
	SD	0.05	67.79	217.6	219.6	12.86	-	

Supplementary Fig. 1

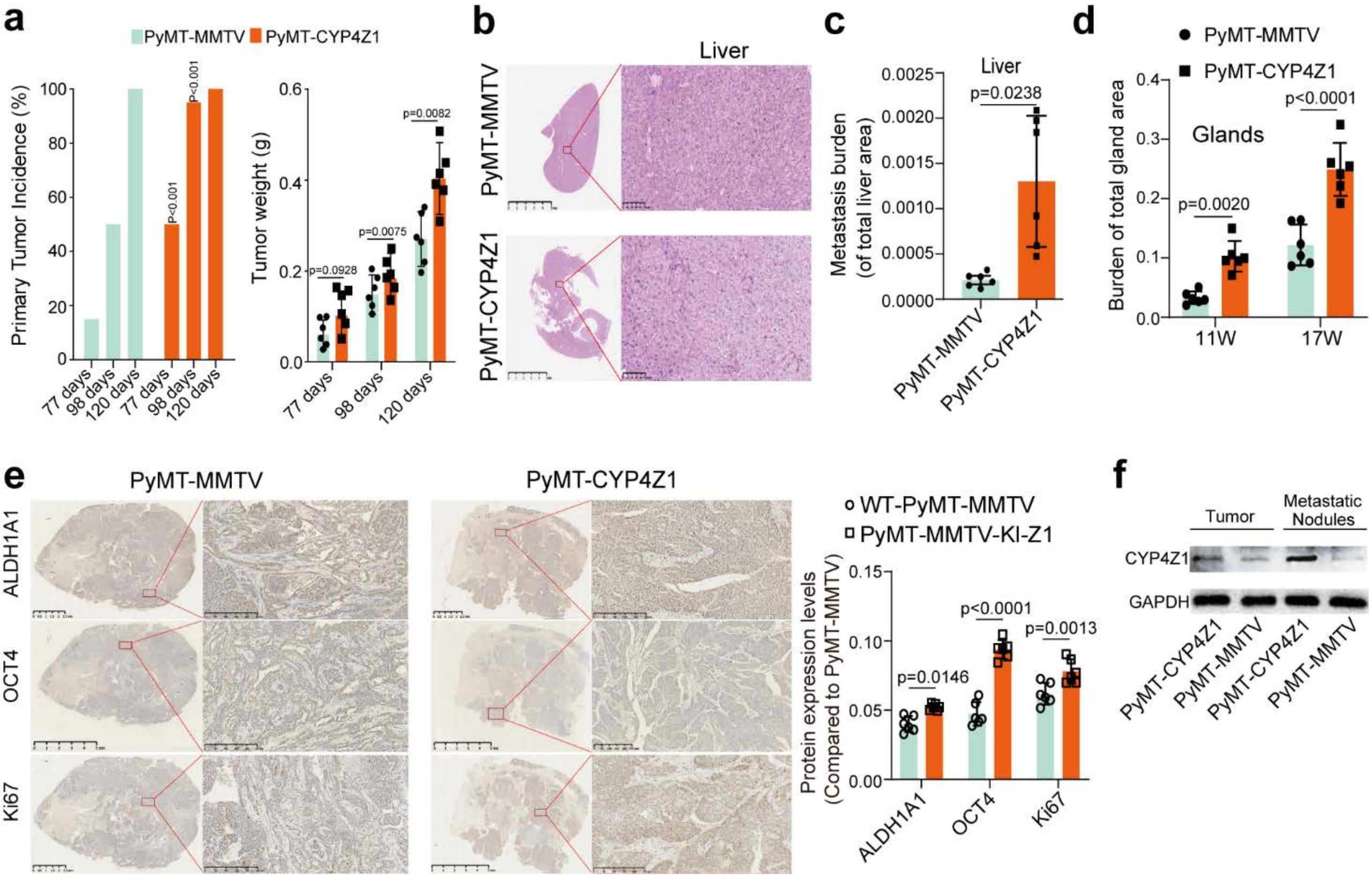

Supplementary Fig. 1 CYP4Z1 knock-in accelerates PyMT-driven mammary tumor progression, metastasis, and tumor-initiating cell (TIC) expansion.

(**a**) The quantitative assessment of metastatic burden in lung tissue or liver tissue. Data were presented as mean ± SEM (n = 6 independent experiments), two-sided Student's t-test.

(**b - d**) HE analyses of gland tissues derived from *PyMT-MMTV* and *PyMT-CYP4Z1* mice, and quantified. 11W, 11 weeks. 17W, 17weeks. Data were presented as mean ± SEM (n = 6 independent experiments), two-way ANOVA test.

(**e**) IHC analyses of Ki67, OCT4, and ALDH1A1 in tumor tissues derived from *PyMT-MMTV* and *PyMT-CYP4Z1* mice, and quantified (relative protein level). Cells were counted in 6 random fields of each slide. Data were presented as mean ± SEM (n = 6 independent experiments), two-way ANOVA test.

(**f**) Western blot was performed to examined CYP4Z1 expression in tumor and metastatic nodules derived from *PyMT-MMTV* and *PyMT-CYP4Z1* mice.

Supplementary Fig. 2

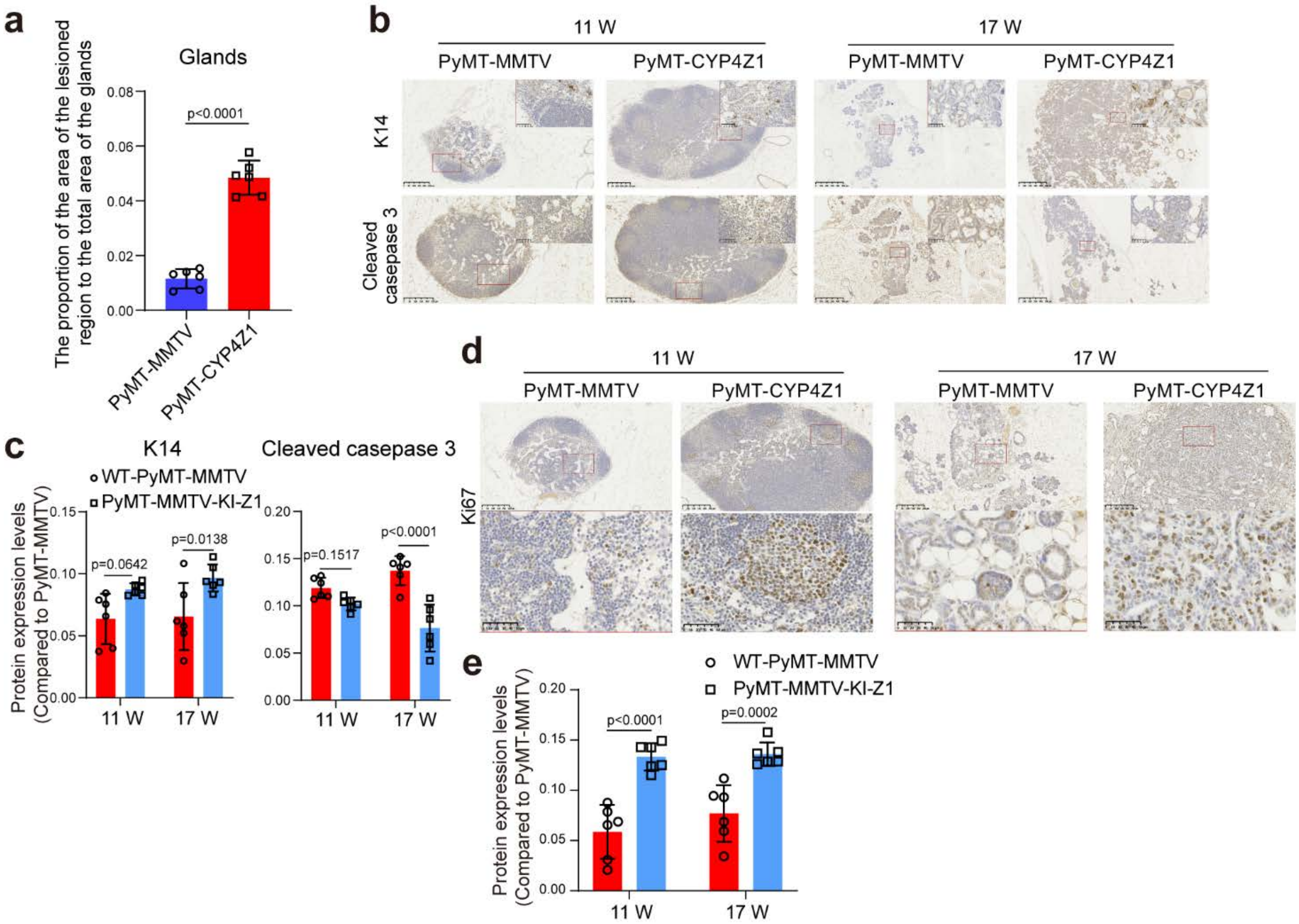

Supplementary Fig. 2 An analysis on the lesion and expression of K14, cleaved caspase 3, and Ki67 in glands or tumors derived from *PyMT-MMTV* and *PyMT-CYP4Z1* mice.

(**a**) Quantify the area of the nodule-presenting region in the gland and calculate its proportion relative to the total area of the gland, cell clusters with a diameter greater than 50 micrometers are deemed to be tumor nodules. Data were presented as mean ± SEM (n = 6 independent experiments), two-sample student's t-test.

(**b - e**) IHC analyses of K14, cleaved caspase 3, and Ki67 in hyperplastic mammary glands derived from *PyMT-MMTV* and *PyMT-CYP4Z1* mice, and quantified (relative protein level). 11W, 11 weeks. 17W, 17 weeks. Cells were counted in 6 random fields of each slide. Data were presented as mean ± SEM (n = 6 independent experiments), two-way ANOVA test.

Supplementary Fig. 3

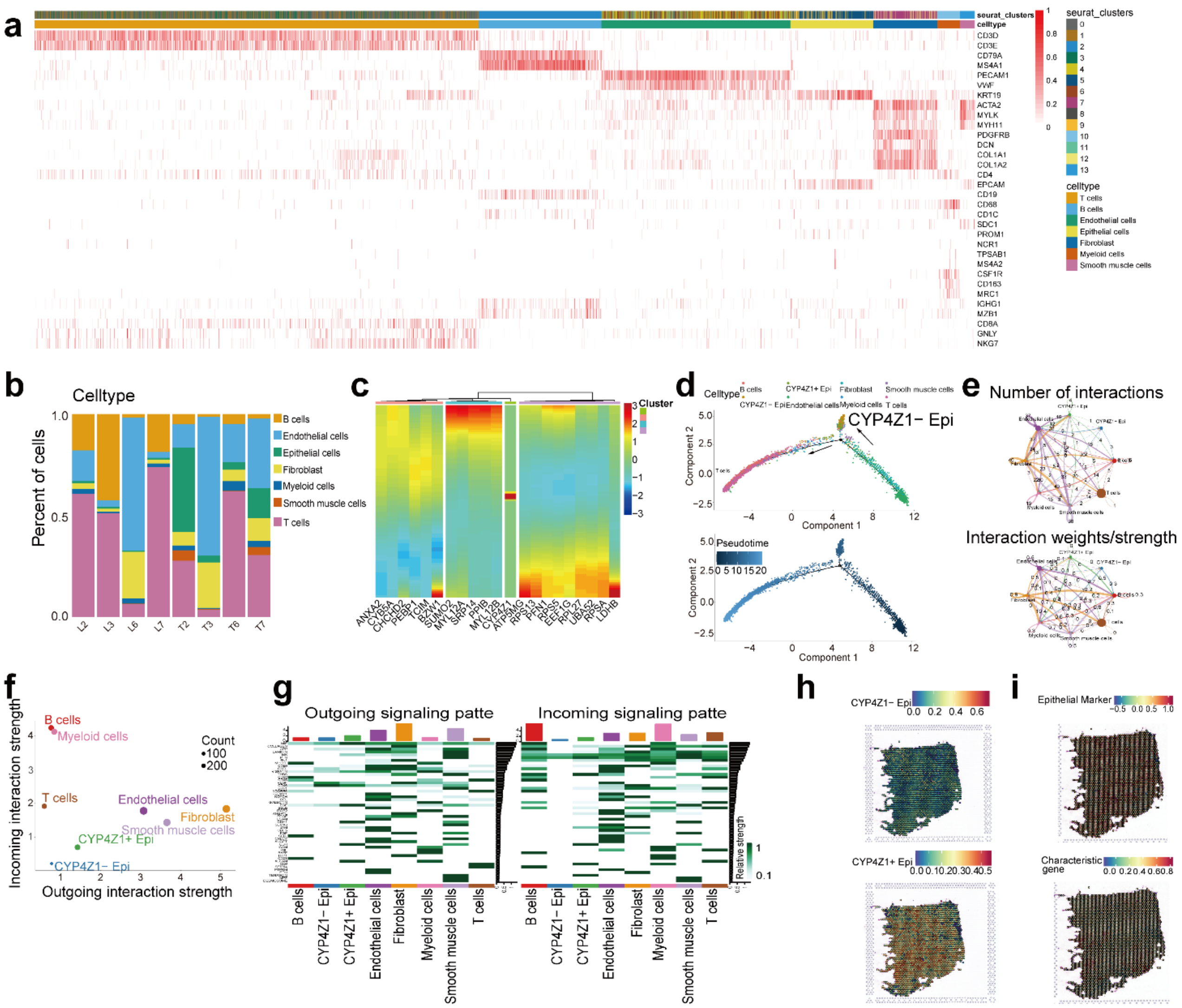

Supplementary Fig. 3 The bioinformatic analysis of CYP4Z1 in breast cancer.

(**a**) Cell differentiation trajectories of cell clusters, the arrows represented two differentiation directions, darker the blue, earlier the differentiation.

(**b**) Interaction number and weight of cell-cell communications between cell clusters.

(**c**) Communication intensity evaluation of different cell clusters.

(**d**) Communication intensities of cell clusters via specific pathways.

(**e**) The enrichment level of epithelial marker expression and CYP4Z1^{+} Epi characteristic genes in spatial spots of breast cancer tissue, calculated by AddModuleScore.

(**f**) The enrichment level of epithelial marker expression and CYP4Z1^{+} Epi characteristic genes in spatial spots of breast cancer tissue, calculated by AddModuleScore.

(**g**) Proportions of CYP4Z1^{+} Epi and CYP4Z1^{-} Epi cell clusters in each spatial spot, estimated by SPOTlight.

(h, i) Spatial transcriptomic analysis of CYP4Z1 + epithelial cell distribution in breast tumor tissue.

Supplementary Fig. 4

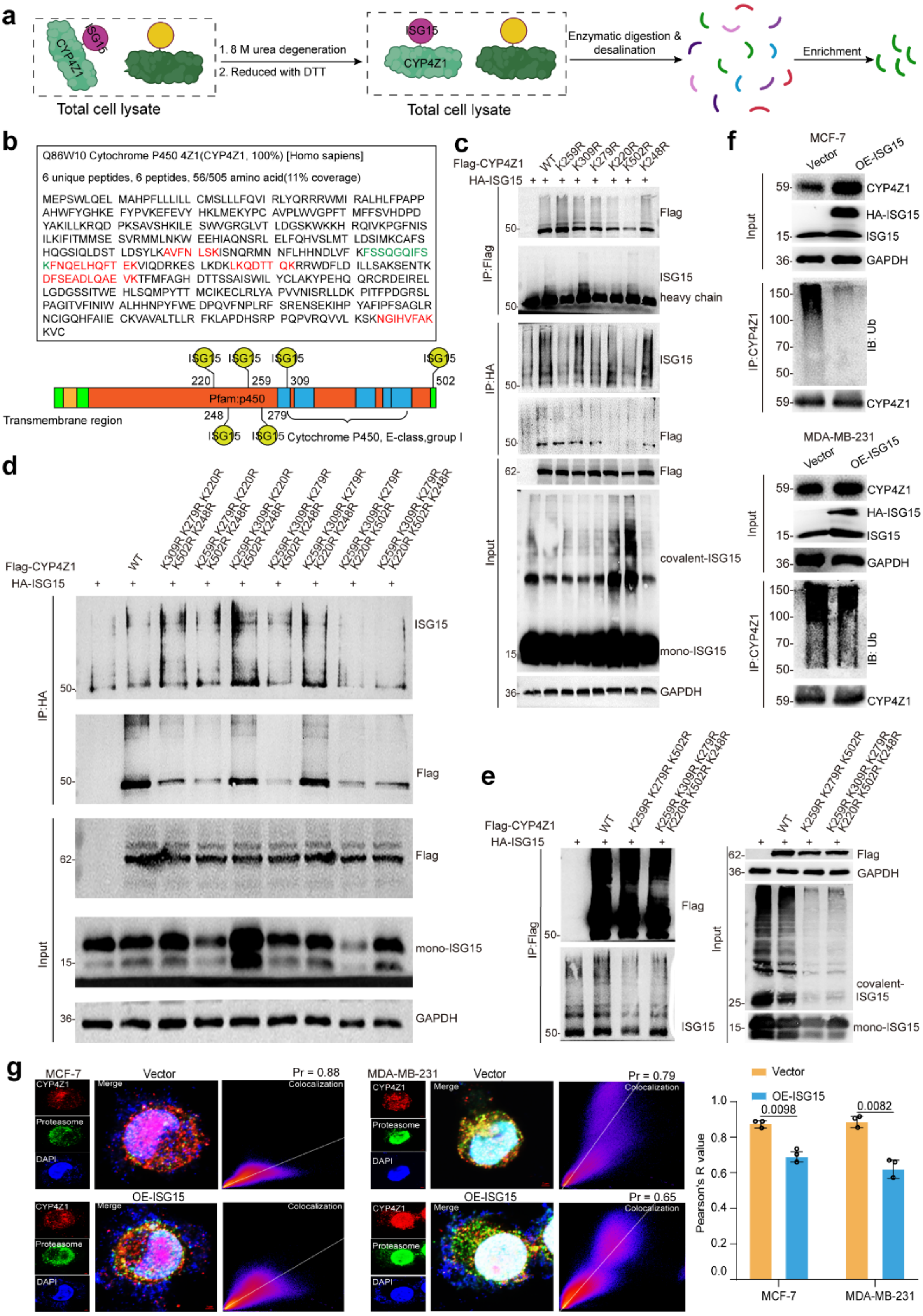

Supplementary Fig. 4 ISG15 can induce the ISGylation of CYP4Z1 at K259, K279, and K502 sites, and reduce the localization of CYP4Z1 in proteasome and its ubiquitination.

(**a**) The diagram of modified-qualitative proteomics analysis.

(**b**) Protein sequence of CYP4Z1, peptides from protein modification mass spectrometry were marked in red or green, and the figure was a schematic of the possible ISGylation site on the CYP4Z1 protein.

(**c - e**) Denatured co-IP analysis of ISGylation of WT CYP4Z1 and CYP4Z1 mutants in HEK293T cells transfected with plasmids as indicated.
(**f**) Denatured co-IP analysis of ubiquitination of Vector and ISG15 overexpression in MCF-7 and MDA-MB-231 cells transfected with plasmids as indicated.
(**g**) Co-localization of CYP4Z1 in MCF-7 and MDA-MB-231 cells with the proteasome. Data were presented as mean ± SEM (n = 3 independent experiments), two-way ANOVA test.

Supplementary Fig. 5

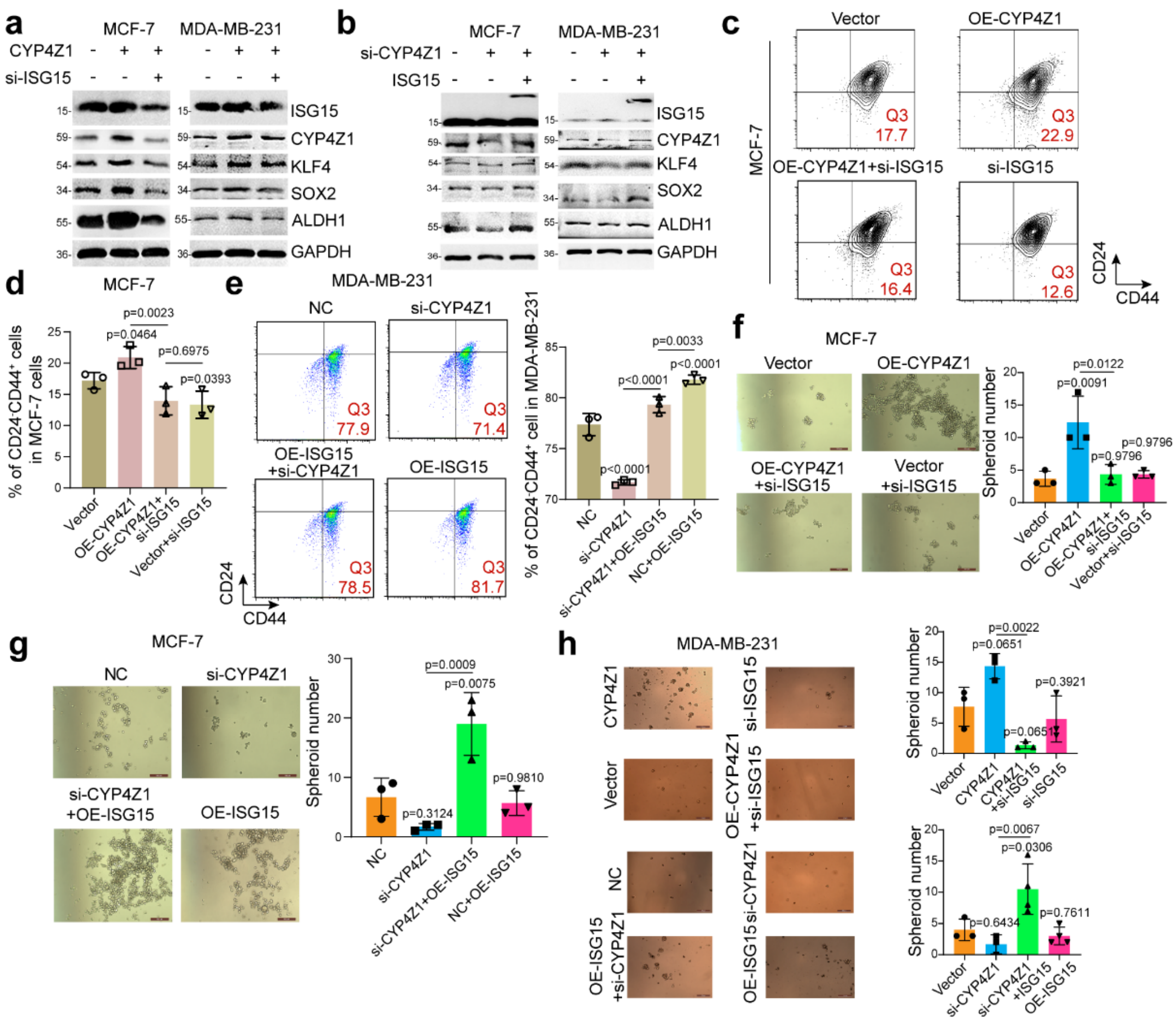

Supplementary Fig.5 CYP4Z1 enhances the TIC-like traits of breast cancer cells dependent on ISG15 expression.

(**a**) The protein levels of CSC markers were examined in MCF-7 and MDA-MB-231 cells with CYP4Z1 overexpression as well as ISG15 knockdown or not.

(**b**) The protein levels of CSC markers were examined in MCF-7 and MDA-MB-231 cells with CYP4Z1 knockdown plus ISG15 overexpression or not.

(**c**) The $CD44^{+}/CD24^{-}$ subpopulation was detected in MCF-7 cells with CYP4Z1 overexpression plus ISG15 knockdown or not.

(**d**) The $CD44^{+}/CD24^{-}$ subpopulation described in (**c**) was quantified. Data were presented as mean ± SEM (n = 3 independent experiments), VS Vector group, one-way ANOVA test.

(**e**) The $CD44^{+}/CD24^{-}$ subpopulation was detected in MDA-MB-231 cells with CYP4Z1 knockdown plus ISG15 knockdown or not. Data were presented as mean ± SEM (n = 3 independent experiments), VS Vector group, one-way ANOVA test.

(**f**) Mammary-spheroid formation ability was evaluated in MCF-7 cells with CYP4Z1 overexpression plus ISG15 knockdown or not, and quantified. Data were presented as mean ± SEM (n = 3 independent experiments), VS Vector group, one-way ANOVA test.

(**g**) Mammary-spheroid formation ability was evaluated in MCF-7 cells with CYP4Z1 knockdown plus ISG15 overexpression or not.

(**h**) Mammary-spheroid formation ability was evaluated in MDA-MB-231 cells, up, CYP4Z1 overexpression plus ISG15 knockdown or not; down, CYP4Z1 knockdown plus ISG15 overexpression or not. Data were presented as mean ± s.e.m. (n = 4 independent experiments), VS Vector group, one-way ANOVA test.

Supplementary Fig. 6

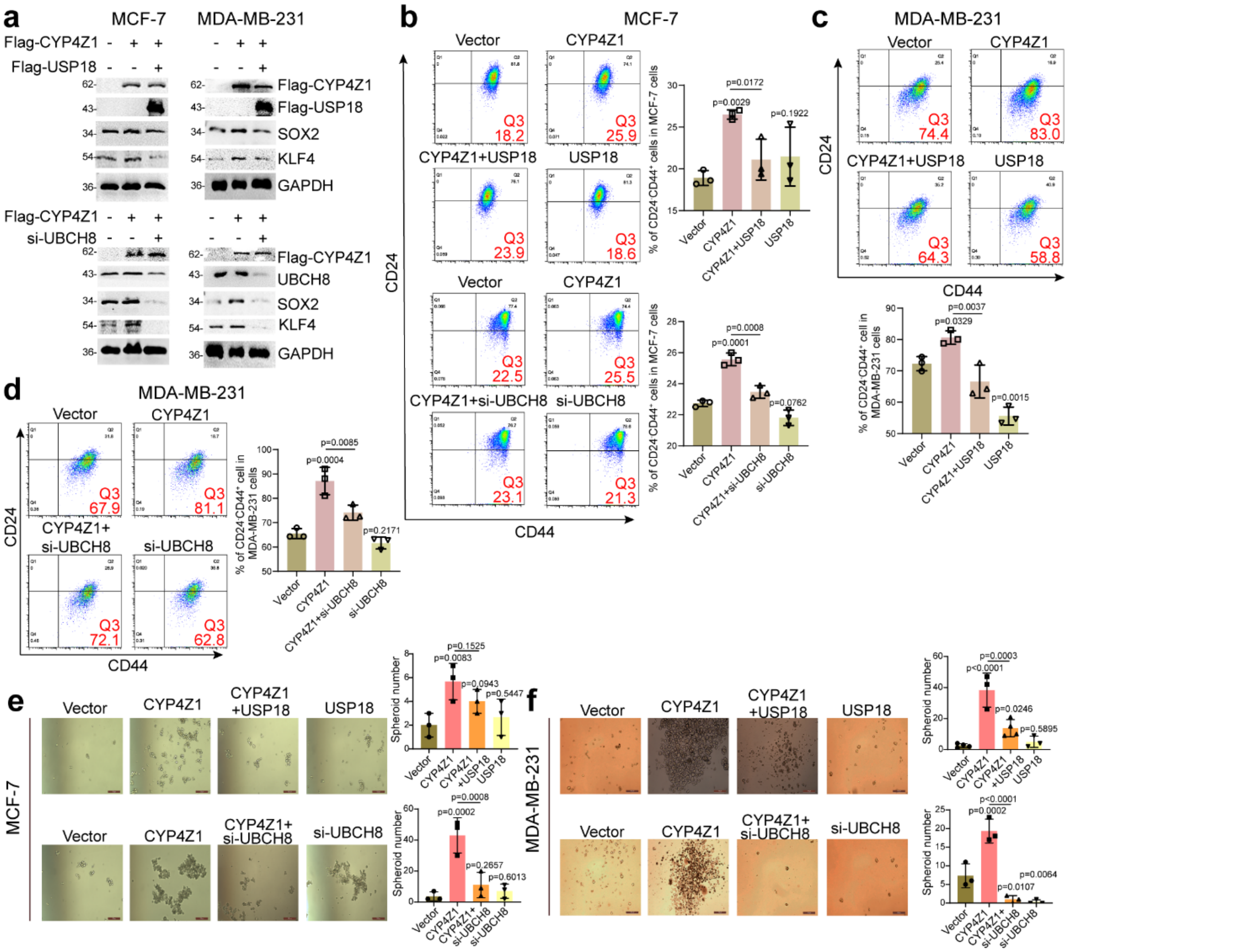

Supplementary Fig. 6 CYP4Z1-mediated effects on the TIC-like traits of breast cancer cells are dependent on ISGylation-related E2 (UBCH8) and deISGylation modification enzyme (USP18).

(**a**) The protein levels of CSC markers were examined in MCF-7 and MDA-MB-231 cells with CYP4Z1 overexpression plus USP18 overexpression (or UBCH8 knockdown) or not.

(**b - d**) The $CD44^+/CD24^-$ subpopulation with tumor-initiating ability was detected in MCF-7 and MDA-MB-231 cells with CYP4Z1 overexpression plus USP18 overexpression (or UBCH8 knockdown) or not. Data were presented as mean ± SEM (n = 3 independent experiments), one-way ANOVA test.

(**e - f**) Mammary-spheroid formation ability was evaluated in MCF-7 and MDA-MB-231 cells with CYP4Z1 overexpression plus USP18 overexpression or not. Data were presented as mean ± SEM (n = 3 independent experiments), one-way ANOVA test.

Supplementary Fig. 7

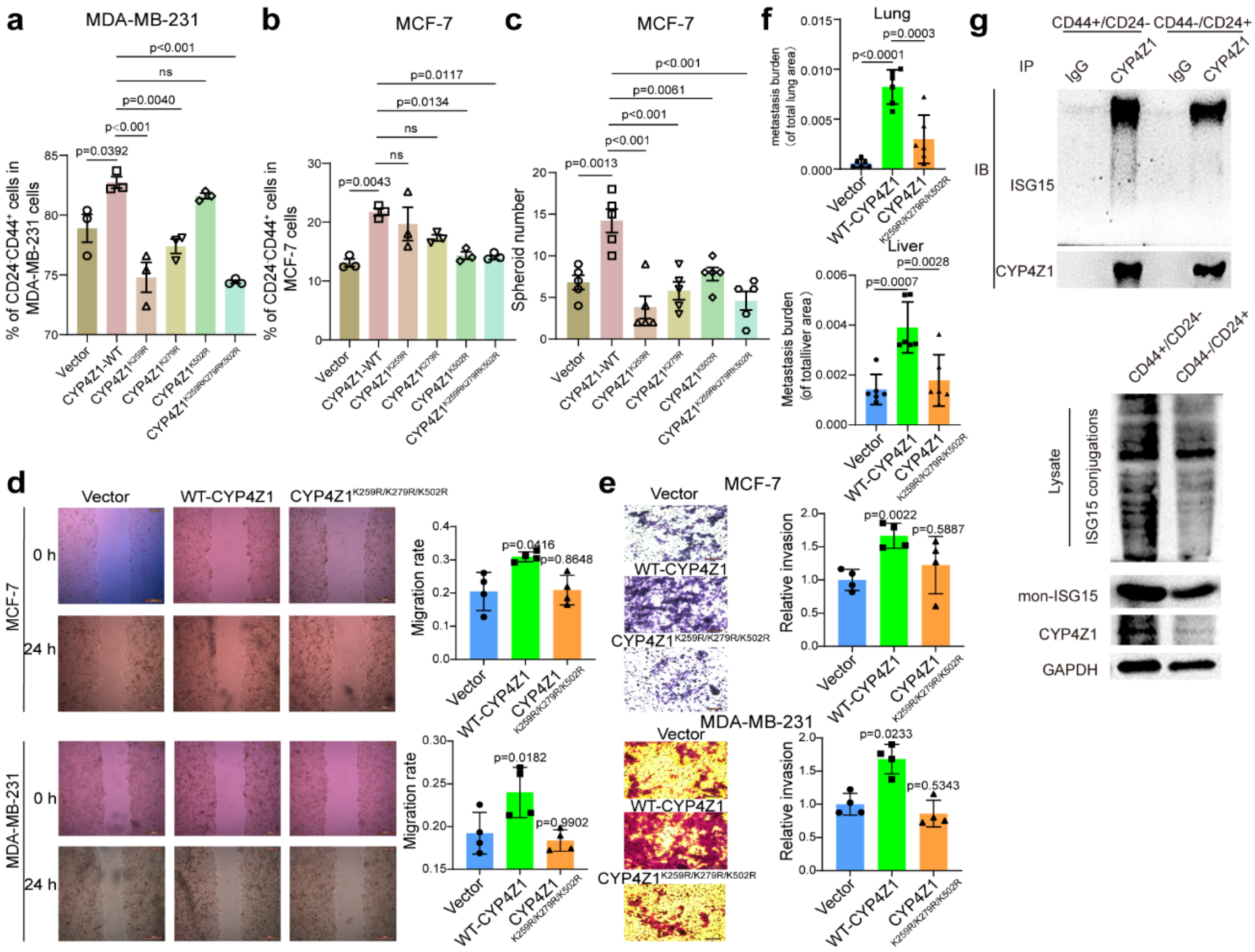

Supplementary Fig. 7 CYP4Z1 enhances the stemness, migrative and invasive ability of breast cancer cells dependent on its ISGylation on K259, K279, and K520 sites.

(**a**) Quantitative analysis of % of $CD24^-CD44^+$ cells in MDA-MB-231 cells in **Fig. 4a**, Data were presented as mean ± s.e.m. (n = 3 independent experiments), one-way ANOVA test.

(**b**) Quantitative analysis of % of $CD24^-CD44^+$ cells in MCF-7 cells in **Fig. 4a**, Data were presented as mean ± SEM. (n = 3 independent experiments), one-way ANOVA test.

(**c**) Quantitative analysis of number of spheroid in **Fig. 4c**, Data were presented as mean ± SEM. (n = 5 independent experiments), one-way ANOVA test.

(**d**) CYP4Z1 promoted breast cancer cells migration as analyzed by scratch wound assay, but this effect was unaffected by mutant ISGylation sites. Scale bar, 200 μm. Quantitative analysis of the migration rates in figure, n = 4 per group, one-way ANOVA test.

(**e**) CYP4Z1 promoted breast cancer cells invasion as analyzed by transwell assay, but this effect was unaffected by mutant ISGylation sites. Scale bar, 100 μm. Quantitative analysis of the migration rates in figure, n = 4 per group, one-way ANOVA test.

(**f**) The quantification of lung and live metastatic nodules described in **Fig. 4j**. n = 6 per group, one-way ANOVA test.

(**g**) The ISGylation level of CYP4Z1 was examined in TICs and non-TICs isolated from tumors derived from derived from *PyMT-CYP4Z1* mice.

Supplementary Fig. 8

a

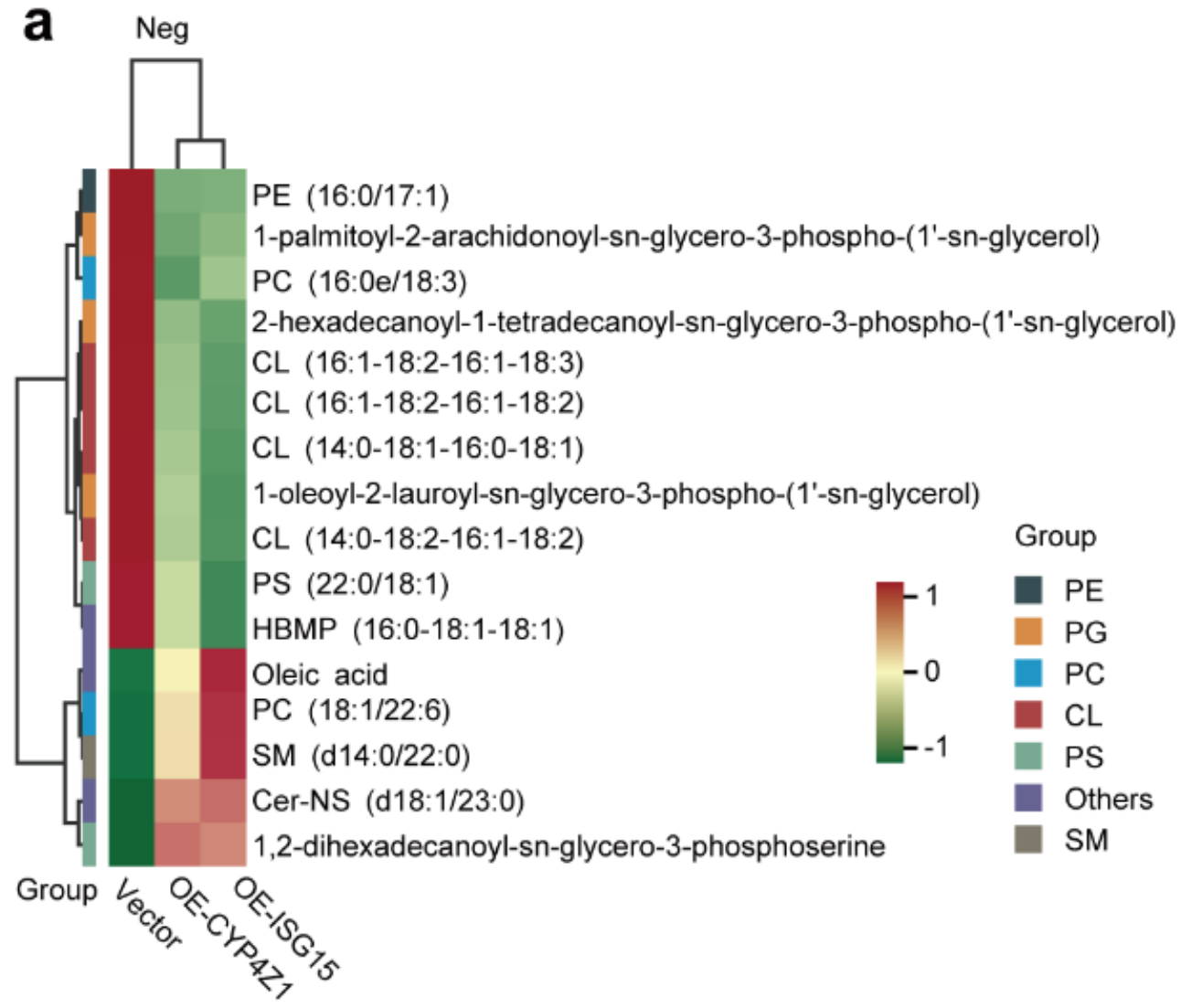

b

Top 10 metabolic compounds with the largest changes in neg

Name	$Log_2(FC)$	
	Vector vs OE-CYP4Z1	Vector vs OE-ISG15
N - Oleoyl Taurine	1.500621776	1.276584973
CL (16:0 - 18:0 - 16:0 - 18:1)	- 1.172451292	1.477818072
1-oleoyl-2-lauroyl-sn -glycero-3-phospho-(1'-sn-glycerol)	0.966610021	1.46937669
2-hexadecanoyl-1-tetradecanoyl-sn-glycero-3-phospho-(1'-sn-glycerol)	- 0.947082697	1.12325489
CL(14:0 -18:1-16:0 -18:1)	0.775821491	1.066517209
1-palmitoyl-2-arachidonoyl-sn-glycero-3-phospho-(1'-sn-glycerol)	- 0.745472954	0.678076903
CL(16:1-18:3-18:2-18:3)	- 0.740947175	0.576202973
PC (14:1(9Z)/14:0)	- 0.712595744	0.809816544
SM (d14:0/22:0)	0.712385117	1.037784184
1-arachidonoyl-sn-glycero-3-phospho-L-serine	- 0.709544375	0.710516892

c

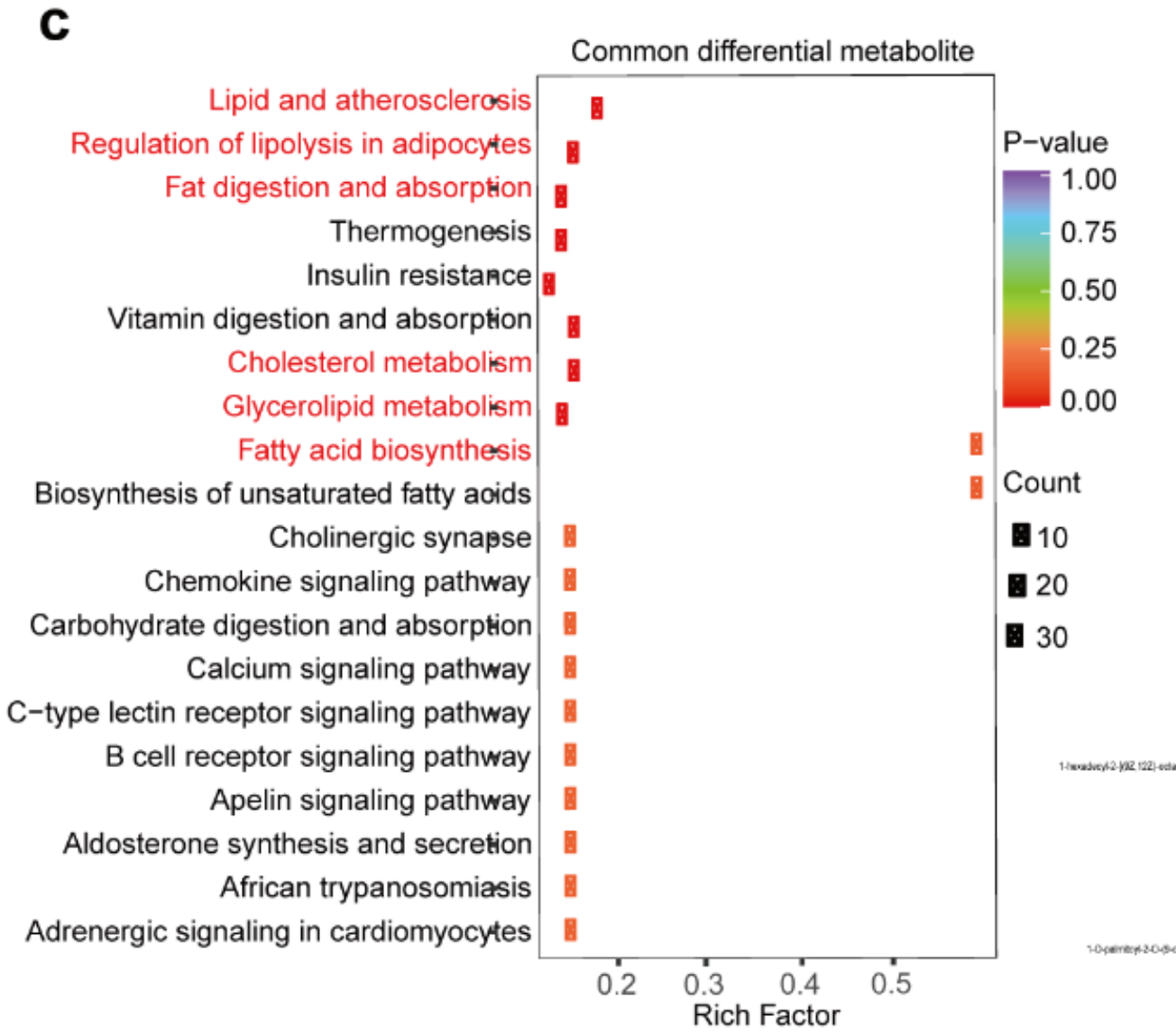

d

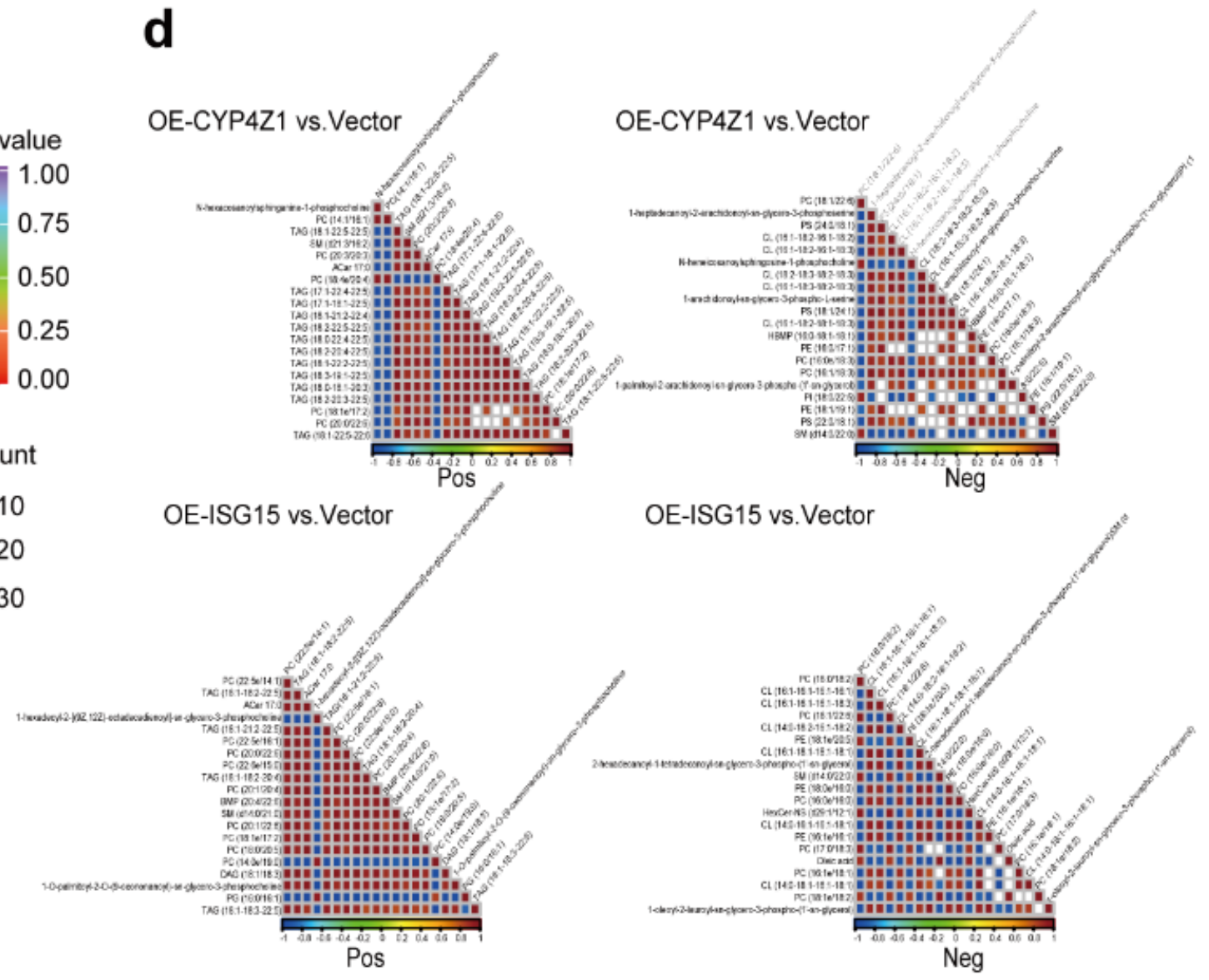

Supplementary Fig. 8 Overexpression of CYP4Z1 and ISG15 causes upregulation of triglyceride metabolites in breast cancer cells.

(**a**) Clustering heat map of total differential lipid compounds in neg. Perform hierarchical cluster analysis (HCA) on the differential lipid compounds between the obtained comparison pairs, and normalize and cluster the relative quantitative value of the differential lipid compounds.

(**b**) The 10 lipid compounds with the largest changes in neg.

(**c**) KEGG enrichment analysis of all differential compounds was performed using the Metware Cloud, a free online platform for data analysis (https://cloud.metware.cn).

(**d**) Correlation diagram of differential lipid compounds. Correlations were highest at 1, complete positive correlation (red) and lowest at - 1, complete negative correlation (blue), the parts without color indicate p-value > 0.05, and the plots showed the differences in p-value values from small to large top20 correlations for lipid compounds.

Supplementary Fig. 9

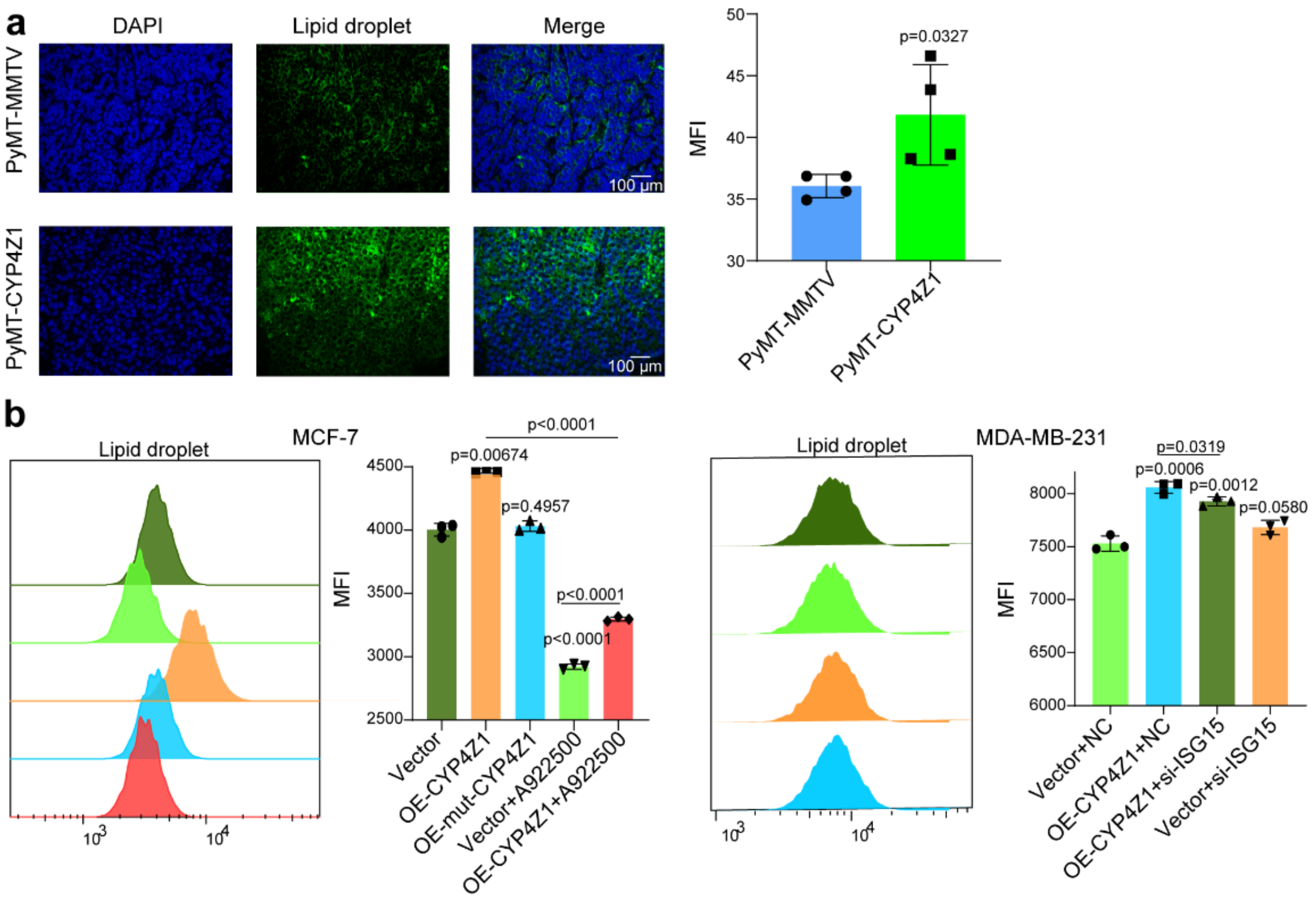

Supplementary Fig. 9 CYP4Z1 promotes lipid droplet formation by promoting triglyceride synthesis.

(**a**) Detection of lipid droplet formation levels in tumors from *PyMT-MMTV* and *PyMT-CYP4Z1* mice by immunofluorescence and quantified. Data were presented as mean ± SEM (n = 6 independent experiments), two-sided Student's t-test.

(**b**) Detection of lipid droplet formation levels by flow cytometry and quantified. Left, CYP4Z1 or CYP4Z1 mutant overexpression plus A922500 or not. Right, CYP4Z1 overexpression plus ISG15 knockdown or not. Data were presented as mean ± SEM (n = 3 independent experiments), one-way ANOVA test.

Supplementary Fig. 10

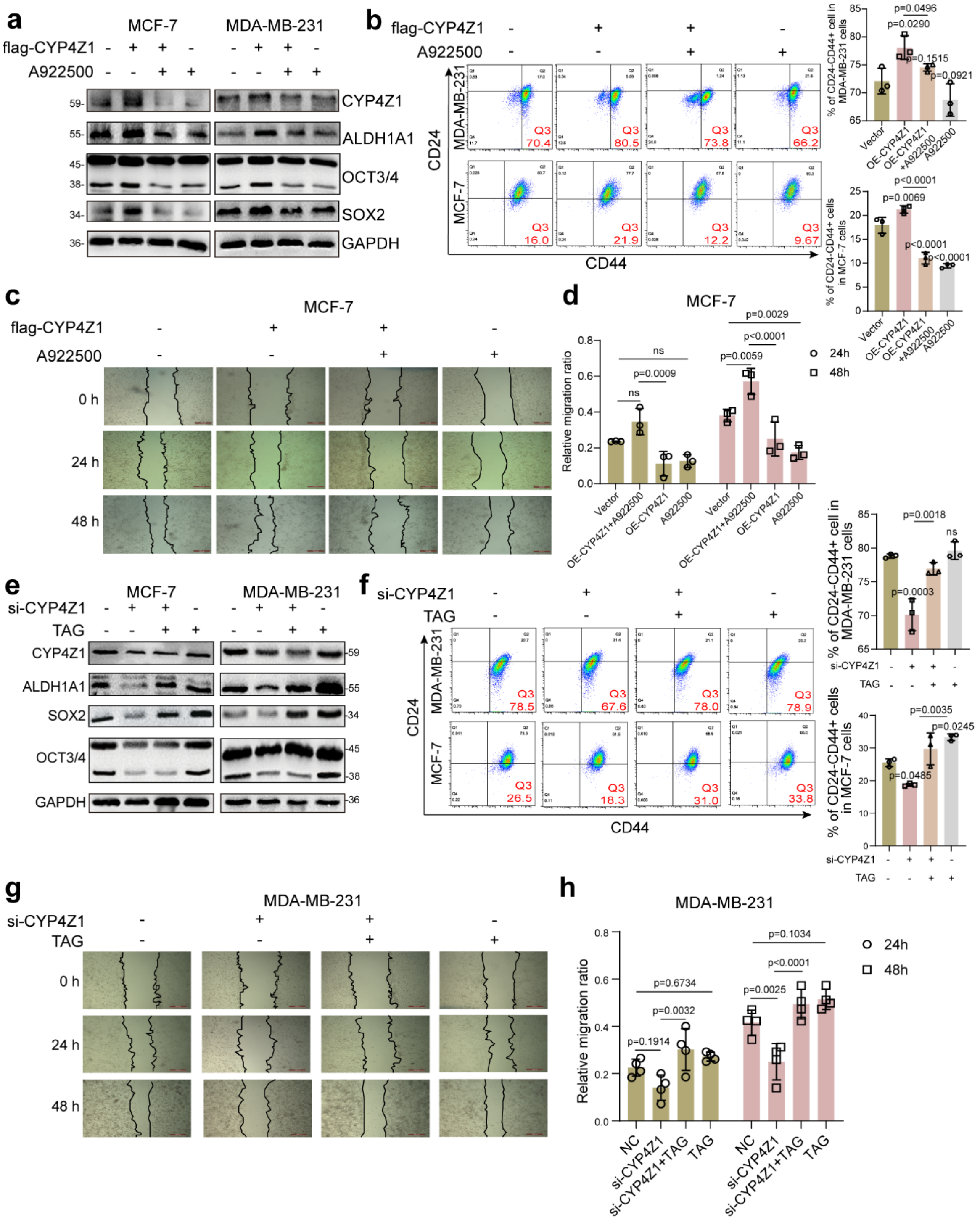

Supplementary Fig. 10 CYP4Z1 enhances the migrative and initial ability of breast cancer cells dependent on TAG synthesis.

(**a**) The protein levels of CSC markers were examined in MCF-7 and MDA-MB-231 cells with CYP4Z1 overexpression plus 50 μM A922500 treatment or not.

(**b**) The CD44$^+$/CD24$^-$ subpopulation was detected in MCF-7 or MDA-MB-231 cells with CYP4Z1 overexpression plus A922500 treatment or not. Data were presented as mean ± SEM (n = 3 independent experiments), VS Vector group, one-way ANOVA test.

(**c** and **d**) Wound healing analysis was performed to detect the migration ability of MCF-7 cells with or without CYP4Z1 overexpression plus the treatment of A922500 and quantified. Data were presented as mean ± SEM (n = 3 independent experiments), two-way ANOVA test.

(**e**) The protein levels of CSC markers were examined in MDA-MB-231 and MCF-7 cells with CYP4Z1 knockdown plus 300 μM TAG treatment or not.
(**f**) The $CD44^{+}/CD24^{-}$ subpopulation was detected in MCF-7 or MDA-MB-231 cells with CYP4Z1 knockdown plus TAG treatment or not. Data were presented as mean ± SEM (n = 3 independent experiments), one-way ANOVA test.
(**g** and **h**) Wound healing analysis was performed to detect the migration ability of MDA-MB-231 cells with or without CYP4Z1 knockdown plus the treatment of TAG and quantified. Data were presented as mean ± SEM (n = 4 independent experiments), two-way ANOVA test.

Supplementary Fig. 11

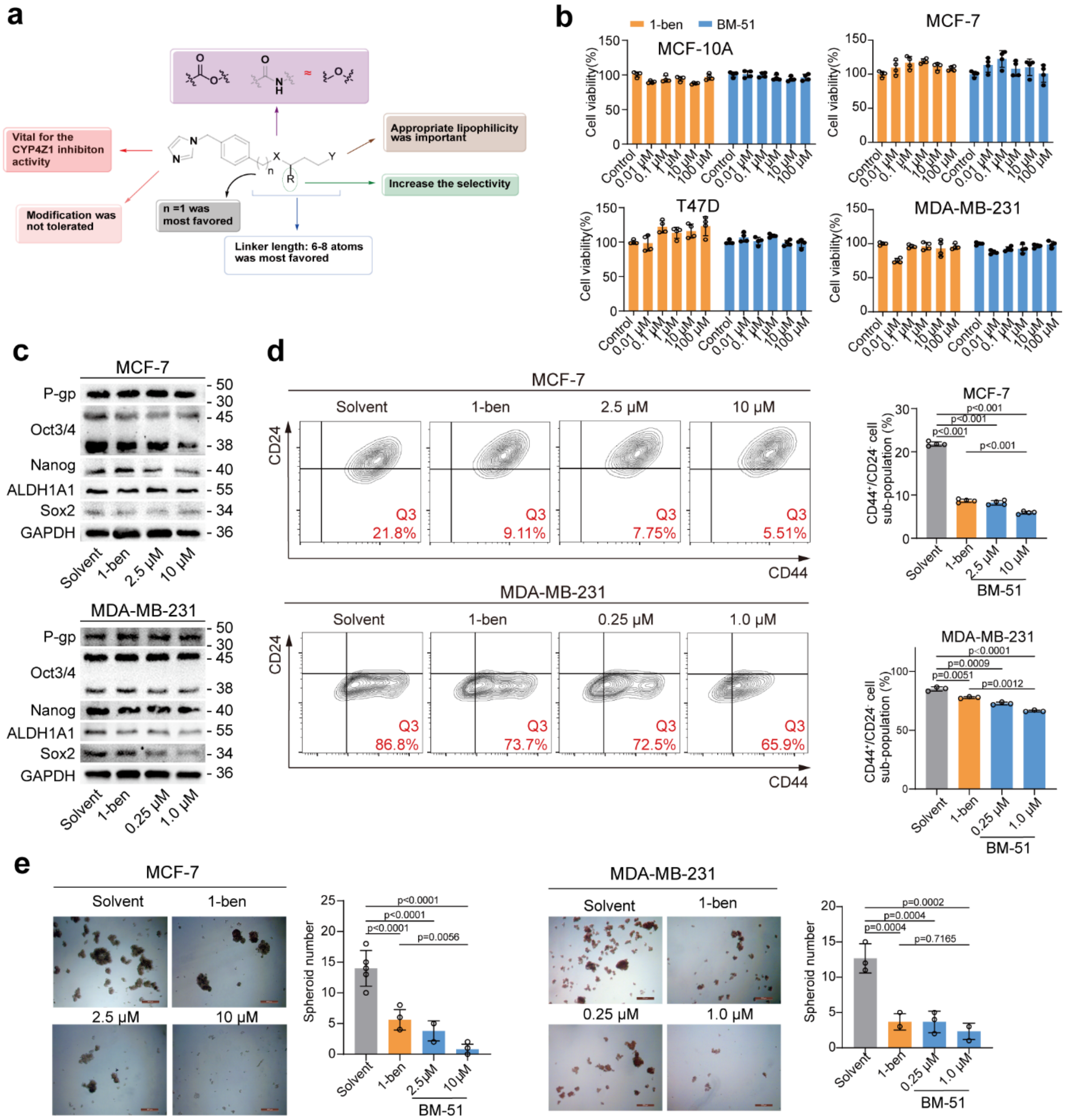

Supplementary Fig. 11 BM-51 significantly suppressed the stemness of breast cancer cells *in vitro*.

(**a**) The detailed structure-activity relationships (SARs) of CYP4Z1 inhibitors.

(**b**) CCK-8 analysis was performed to detect the cell viabilities of MCF-10A, MCF-7, T47D, and MDA-MB-231 cells with 1-ben or **BM-51** treated.

(**c**) Immunoblot analysis of P-gp, Oct3/4, ALDH1A1, Sox2, and GAPDH in MCF-7 and MDA-MB-231 with 1-ben or **BM-51** treated.

(**d**) The $CD44^{+}/CD24^{-}$ subpopulation was detected in MCF-7 and MDA-MB-231 cells with 1-ben or **BM-51** treated. Data were presented as mean ± SEM. (n = 3 or 4 independent experiments), one-way ANOVA test.

(**e**) Mammary-spheroid formation ability was evaluated in MCF-7 and MDA-MB-231 cells with 1-ben or **BM-51** treated. Data were presented as mean ± SEM. (n = 3 - 5 independent experiments), one-way ANOVA test.

Supplementary Fig. 12

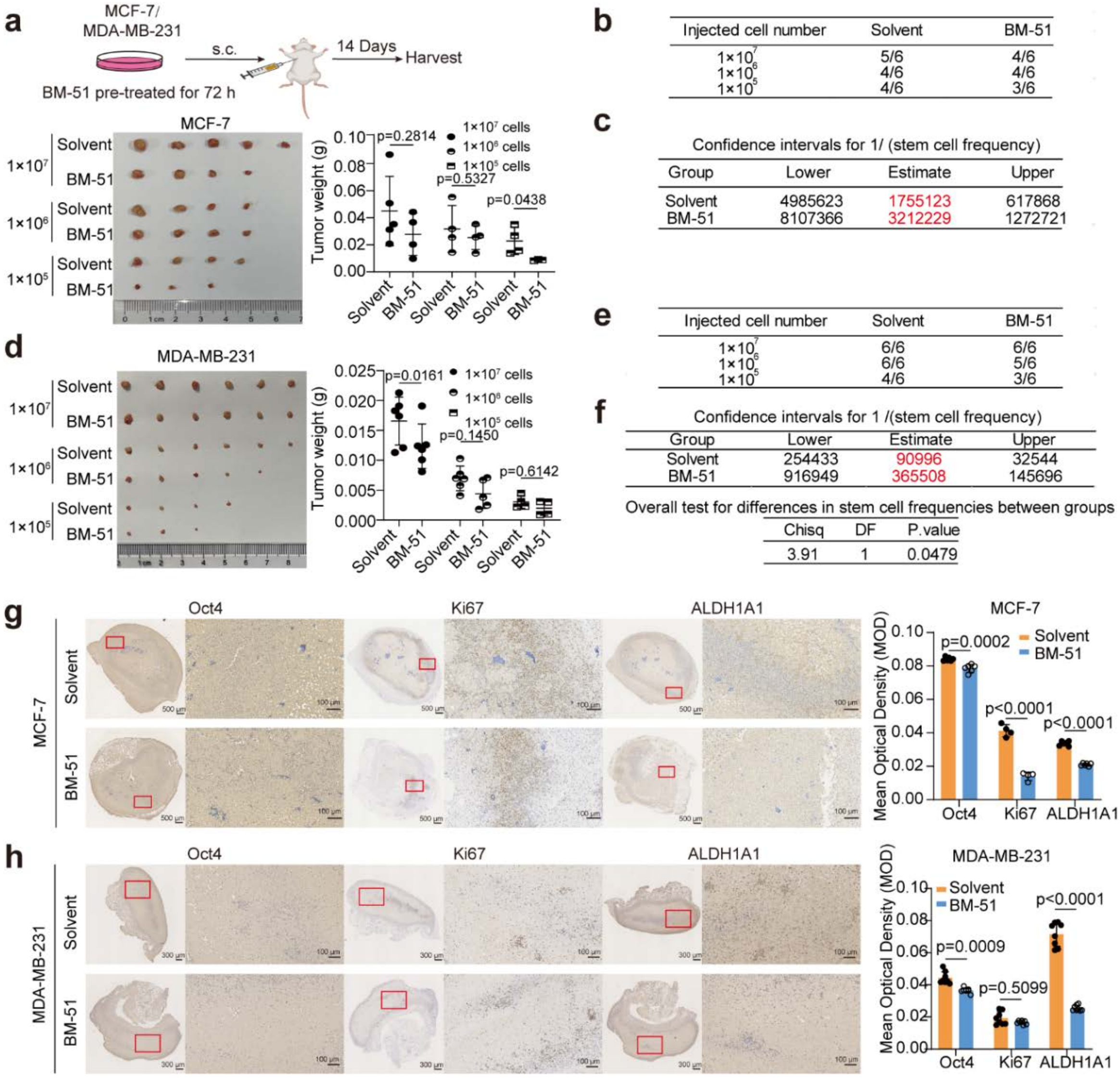

Supplementary Fig. 12 BM-51 significantly suppressed the stemness of breast cancer cells *in vivo*.

(**a**) Images of tumors harvested when serially diluted MCF-7 cells with or without **BM-51** treated. Data are presented as mean ± SEM (n = 6 independent experiments), two-way ANOVA test.

(**b**) Tumor formation ratio was calculated.

(**c**) The stem cell frequencies and colligation test for differences in stem cell frequencies were analyzed using the ELDA tool (http://bioinf.wehi.edu.au/software/elda/) based on the results of (**a**).

(**d**) Images of tumors harvested when serially diluted MDA-MB-231 cells with or without **BM-51** treated. Data are presented as mean ± SEM (n = 6 independent experiments), two-way ANOVA test.

(**e**) Tumor formation ratio was calculated.

(**f**) The stem cell frequencies and colligation test for differences in stem cell frequencies were analyzed using the ELDA tool (http://bioinf.wehi.edu.au/software/elda/) based on the results of (**d**).

(**g**) IHC analysis was performed to detect the expression of stemness markers in tumors from (**a**).

(**h**) IHC analysis was performed to detect the expression of stemness markers in tumors from (**d**). Data are presented as mean ± SEM (n = 6 independent experiments), two-way ANOVA test.

Supplementary Fig. 13

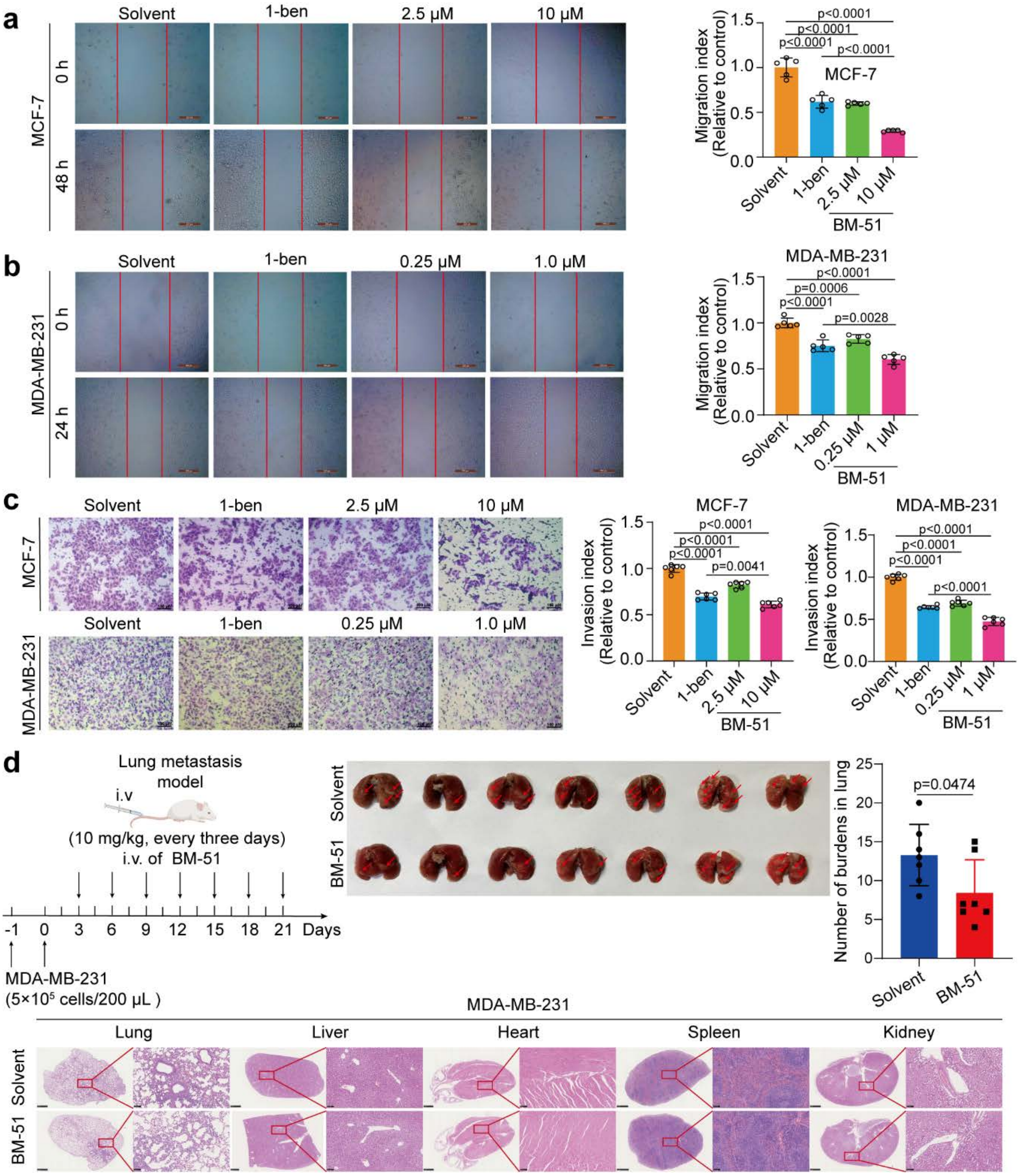

Supplementary Fig. 13 BM-51 significantly suppressed the metastasis of breast cancer cells.
(**a - b**) **BM-51** inhibited breast cancer cells migration as analyzed by scratch wound assay. Scale bar, 200 µm. Quantitative analysis of the migration rates in figure, n = 5 per group, one-way ANOVA test.
(**c**) **BM-51** inhibited breast cancer cells invasion as analyzed by transwell assay. Scale bar, 100 µm. Quantitative analysis of the migration rates in figure, n = 6 per group, one-way ANOVA test.
(**d**) **BM-51** exerted significant effects on breast cancer metastasis through lung metastasis experiment with little toxicity on other organs.

Supplementary Fig. 14

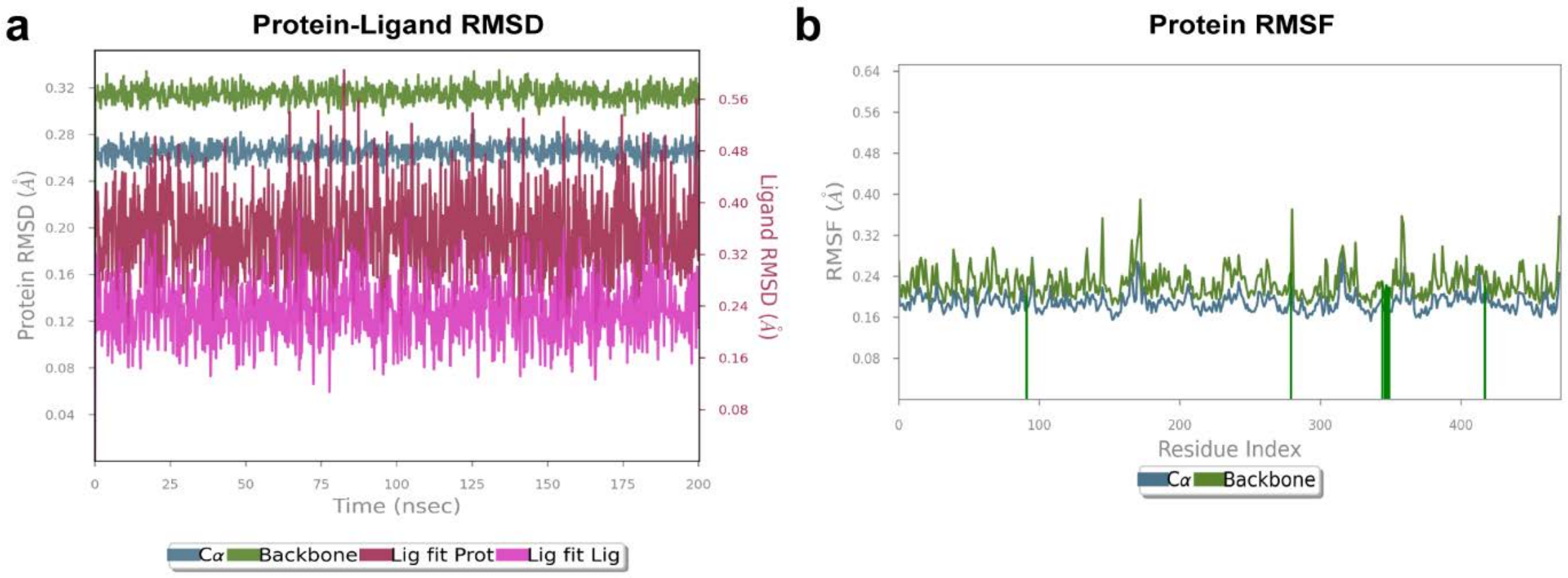

Supplementary Fig. 14 Molecular simulation dynamics of the ideal active pocket of CYP4Z1 throughout a 200 ns period.

(**a**) The root mean square deviation (RMSD) and (**b**) root mean square fluctuation (RMSF) curves.

Supplementary Fig. 15

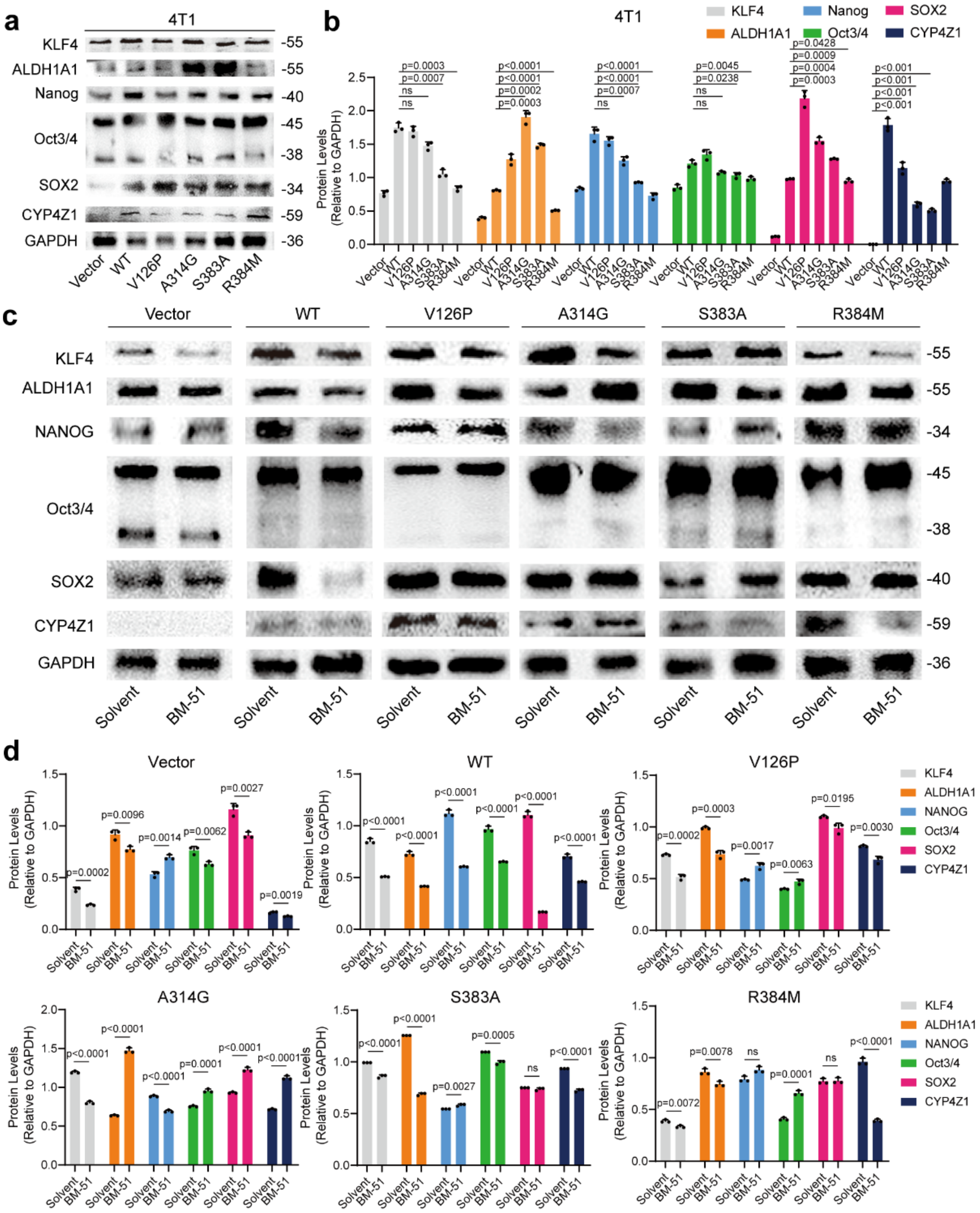

Supplementary Fig. 15 BM-51 attenuates the TIC-like traits of breast cancer cells by targeting CYP4Z1 at Arg384, Ser383, Val126, Ala314.

(**a** and **b**) The protein levels of CSC markers were examined in 4T1 cells with CYP4Z1 or mutants' overexpression.

(**c** and **d**) The protein levels of CSC markers were examined in 4T1 cells with CYP4Z1 or mutants' overexpression plus **BM-51** treatment or not. Data were presented as mean ± SEM. (n = 3 independent experiments), two-sided Student' s t-test.

Supplementary Fig. 16

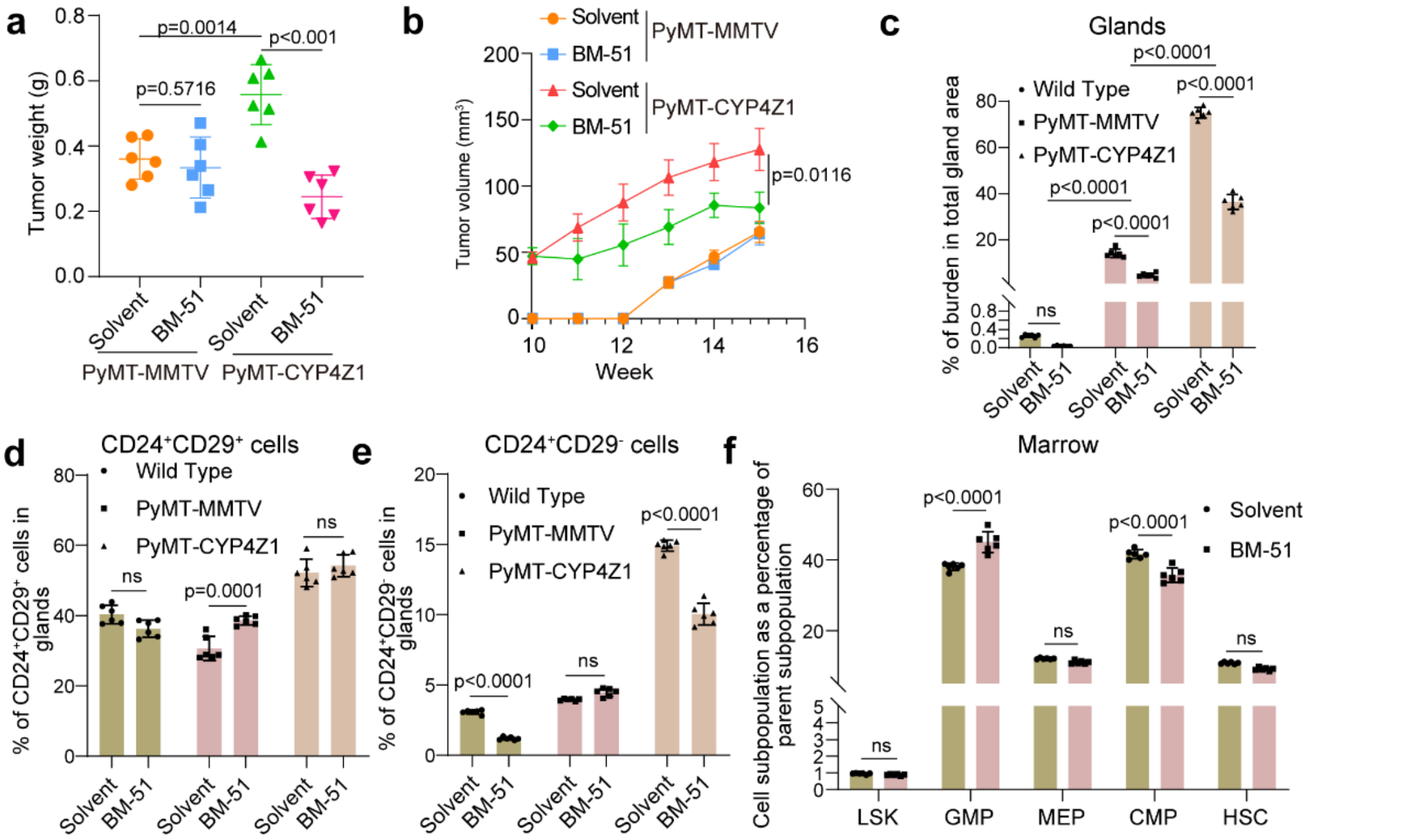

Supplementary Fig. 16 BM-51 inhibits the proportion of stemness subpopulations of mouse breast cancer cells.

(**a**) Tumor weight derived mice depicted in **Fig. 8a**. mean ± SEM (n = 6 independent experiments), two-way ANOVA test.

(**b**) Tumor volume derived mice depicted in **Fig. 8a**. mean ± SEM (n = 6 independent experiments), two-way ANOVA test.

(**c**) The quantification of HE analyses of gland tissues derived from PyMT-MMTV and PyMT-CYP4Z1 mice. Data were presented as mean ± SEM (n = 6 independent experiments), two-way ANOVA test.

(**d**) and (**e**) are the quantitative results of **Fig. 8f**. Data were presented as mean ± SEM, two-way ANOVA test.

(**f**) Quantitative results of **Fig. 8f**. Data were presented as mean ± SEM, two-way ANOVA test.

Supplementary Fig. 17

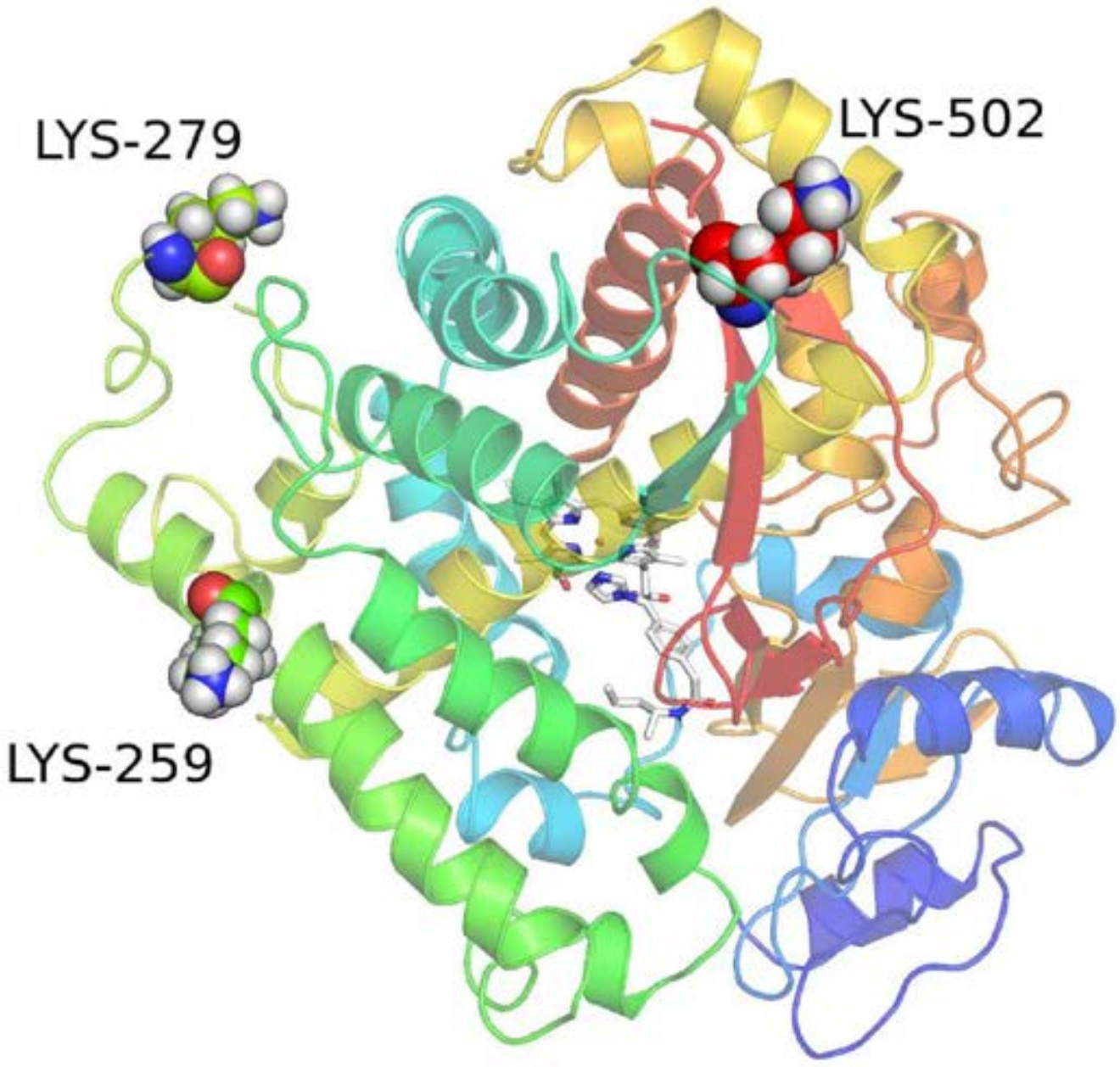

Supplementary Fig. 17 Spatial separation of BM-51 binding pocket and ISGylation sites (K259/K279/K502) on CYP4Z1.

Supplementary Note

Synthetic scheme

Synthetic routes of designed compounds BM-1-BM-53

Scheme 1. Synthesis of compound **3**

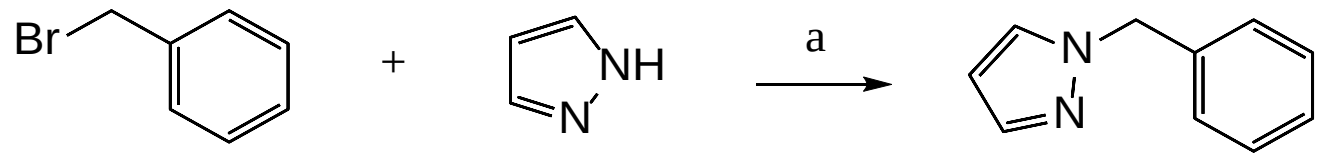

1 2 3 (BM-1)

Reagents and conditions: (a) CsF, CH_3CN, 85℃, 4 h, 85%.

Scheme 2. Synthesis of compounds **5a - 5f**

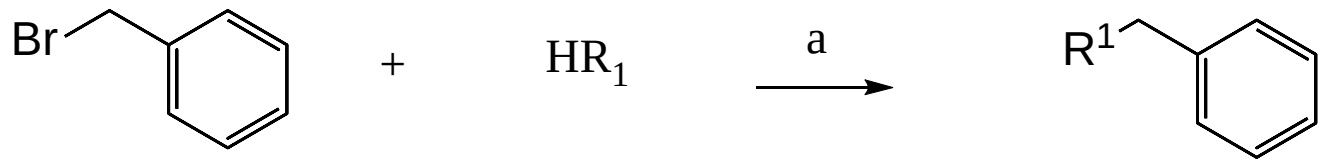

1 4a-4f 5a-5f

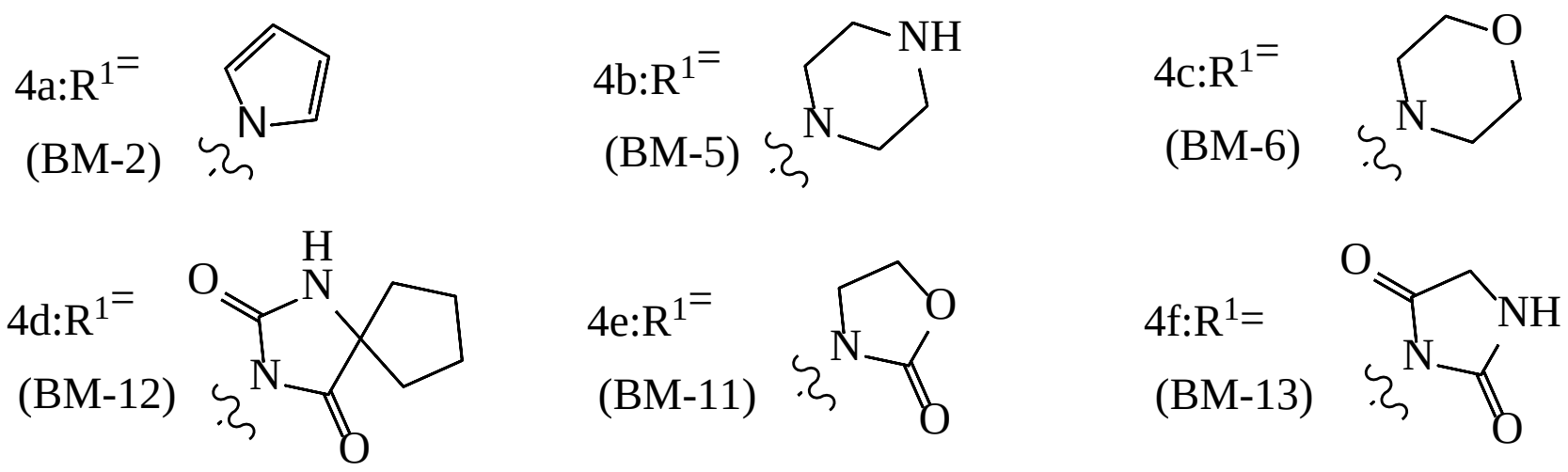

Reagents and conditions: (a) K_2CO_3, DMF, 65℃, 2 h, 68-90%.

Scheme 3. Synthesis of compound **7**

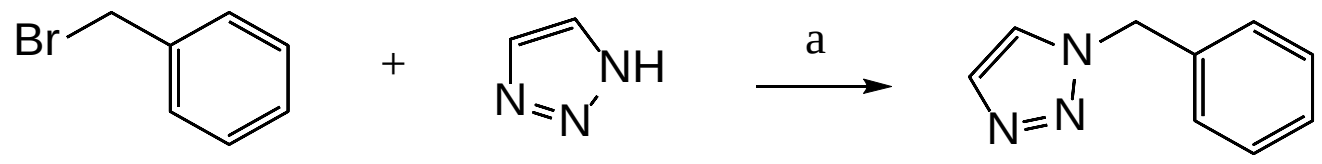

1 6 7 (BM-7)

Reagents and conditions: (a) K_2CO_3, KI, DMF, 65℃, 2 h, 76%.

Scheme 4. Synthesis of compounds **9a - 9b**

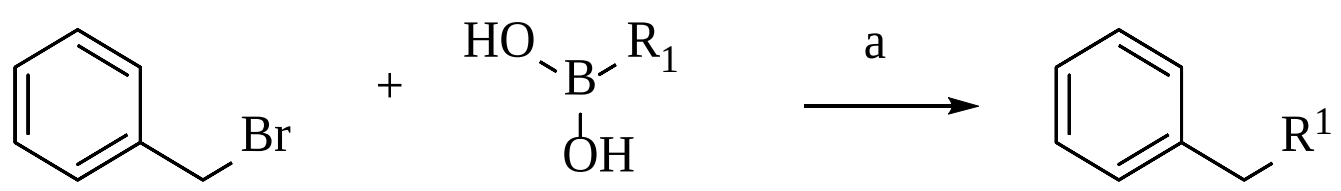

1 8a-8b 9a-9b

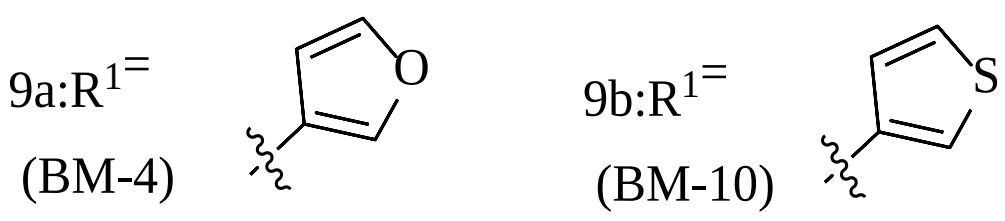

Reagents and conditions: (a) Tetrakis(triphenylphosphine)palladium, Na_2CO_3, Toluene, 80℃, 8h, 80-85%.

Scheme 5. Synthesis of compound **12**

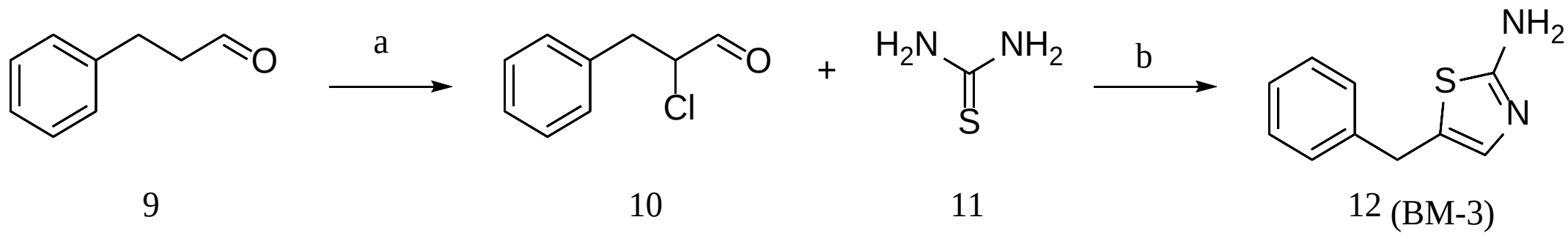

Reagents and conditions: (a) *N*-Chlorosuccinimide, *L*-Proline, DCM, r.t, 2 h, 90%; (b) $POCl_3$, 80℃, 6 h, 75%.

Scheme 6. Synthesis of compounds **15a - 15i**

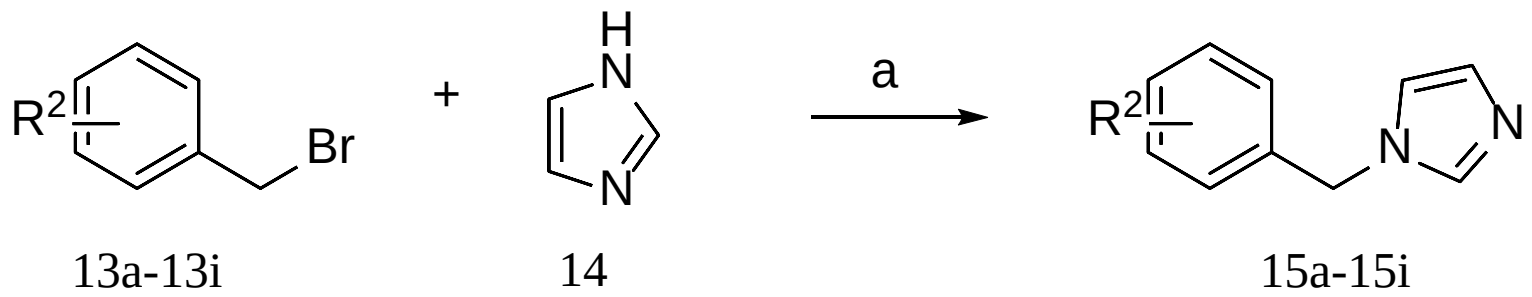

15a (BM-14): R^2=*p*-CH_3
15b (BM-15): R^2=*m*-CH_3
15c (BM-16): R^2=*o*-CH_3
15d (BM-17): R^2=*p*-CF_3
15e (BM-18): R^2=*m*-CF_3
15f (BM-19): R^2=*o*-CF_3
15g (BM-24): R^2=*p*-propyl
15h (BM-21): R^2=*p*-OCH_3
15i (BM-22): R^2=*m*-OCH_3

Reagents and conditions: (a) K_2CO_3, DMF, 65℃, 2 h, 65-90%.

Scheme 7. Synthesis of compounds **19a - 19b**

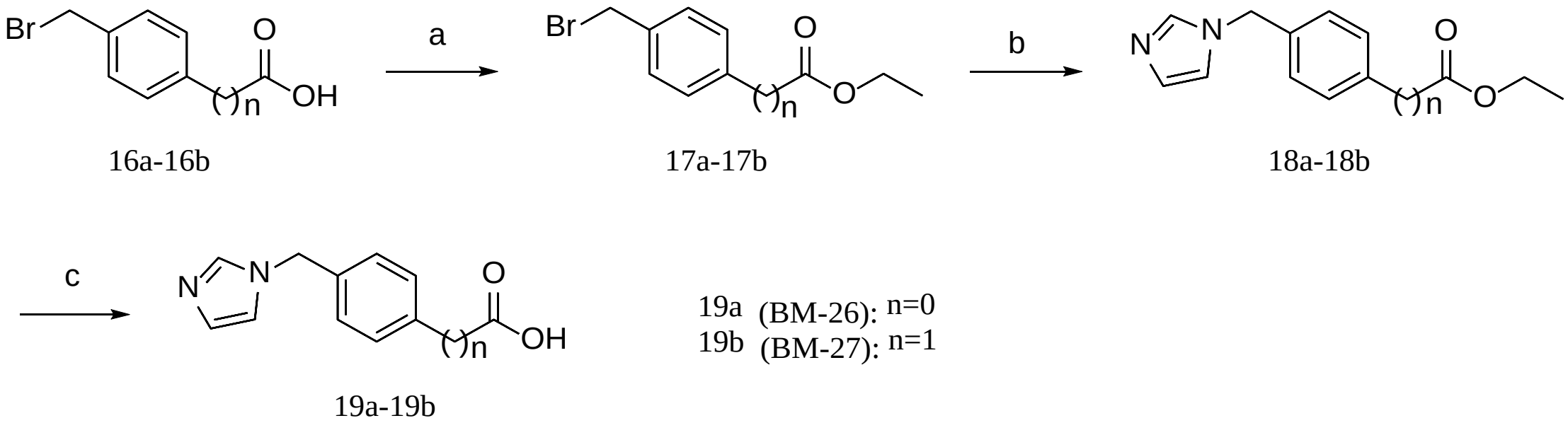

19a (BM-26): n=0
19b (BM-27): n=1

Reagents and conditions: (a) $SOCl_2$, EtOH, 80℃, 2h, 95%; (b) K_2CO_3, DMF, 65℃, 2 h, 65-85%; (c) NaOH, THF:H_2O=4:1, r.t, 2 h, 87%.

Scheme 8. Synthesis of compounds **23a - 23c**

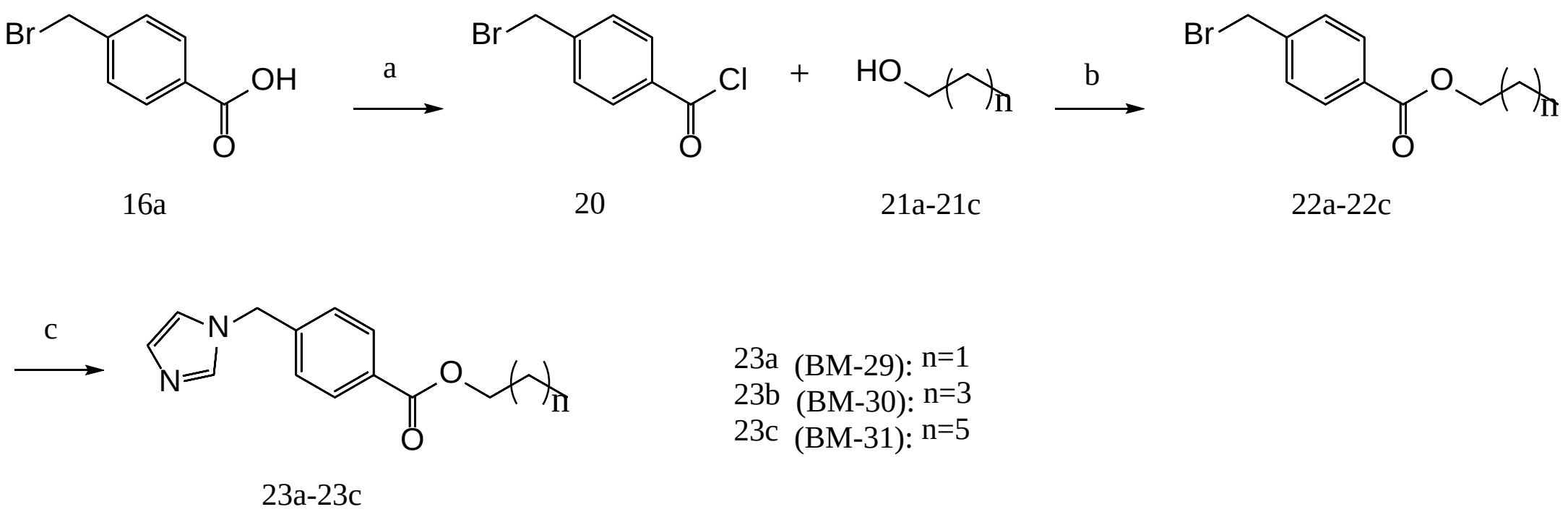

23a (BM-29): n=1
23b (BM-30): n=3
23c (BM-31): n=5

Reagents and conditions: (a) $SOCl_2$, 80℃, 2 h, 95%; (b) DCM, r.t, 2 h, 80-89%; (c) K_2CO_3, DMF, 65℃, 2 h, 65-85%.

Scheme 9. Synthesis of compounds **27a - 27c**

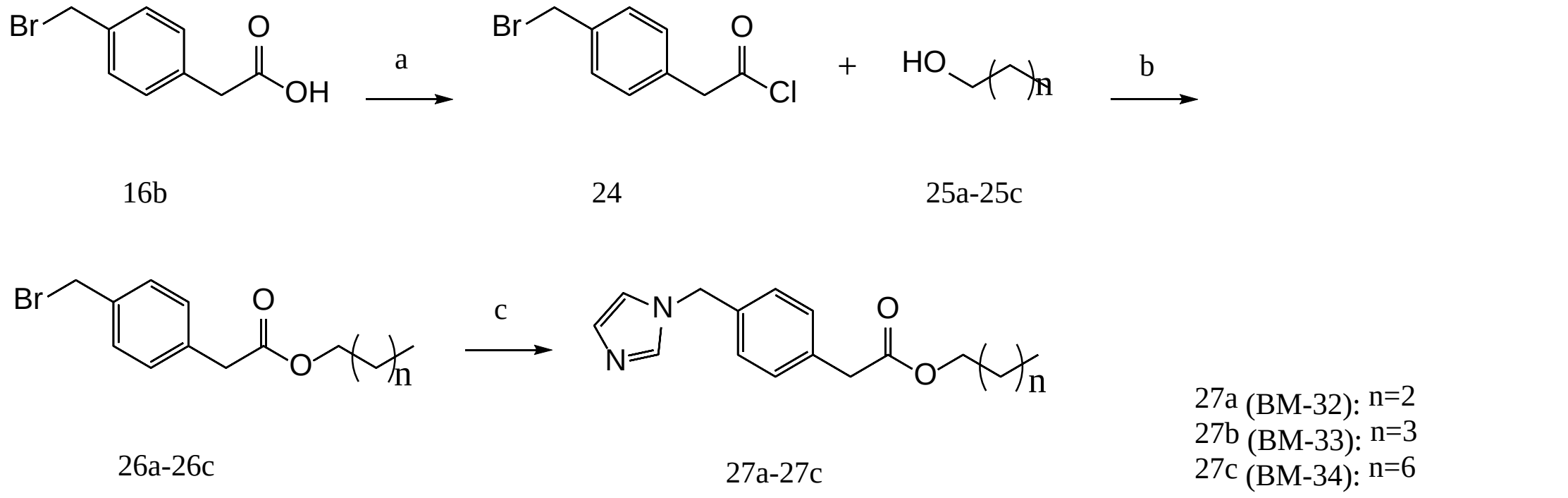

Reagents and conditions: (a) $SOCl_2$, 80℃, 2 h, 96%; (b) DCM, r.t, 2 h, 75-83%; (c) K_2CO_3, DMF, 65℃, 2 h, 65-79%.

Scheme 10. Synthesis of compounds **29a - 29b**

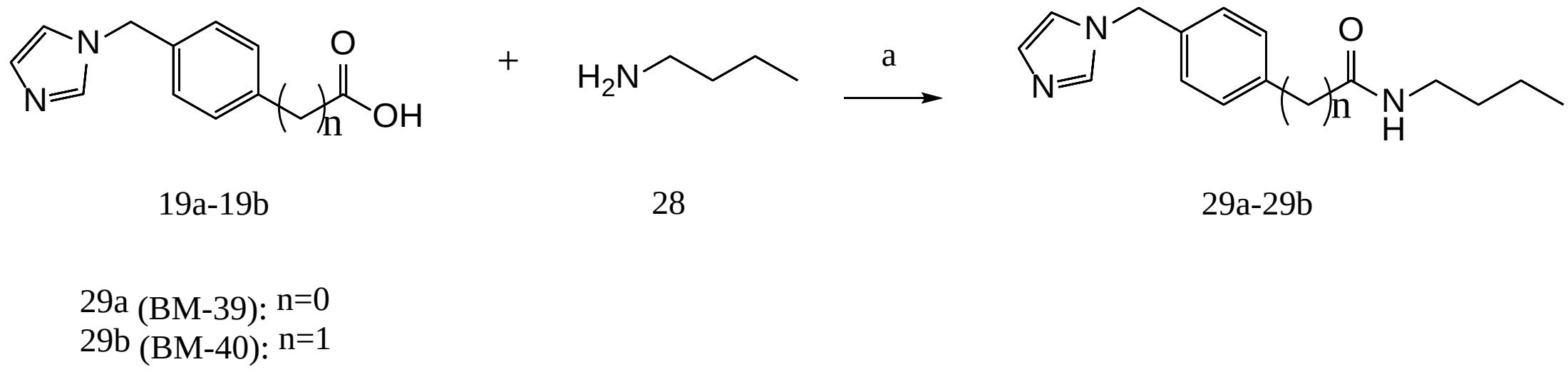

Reagents and conditions: (a) HATU, DIPEA, DMF, r.t, 80-88%.

Scheme 11. Synthesis of compounds **38a - 38c**

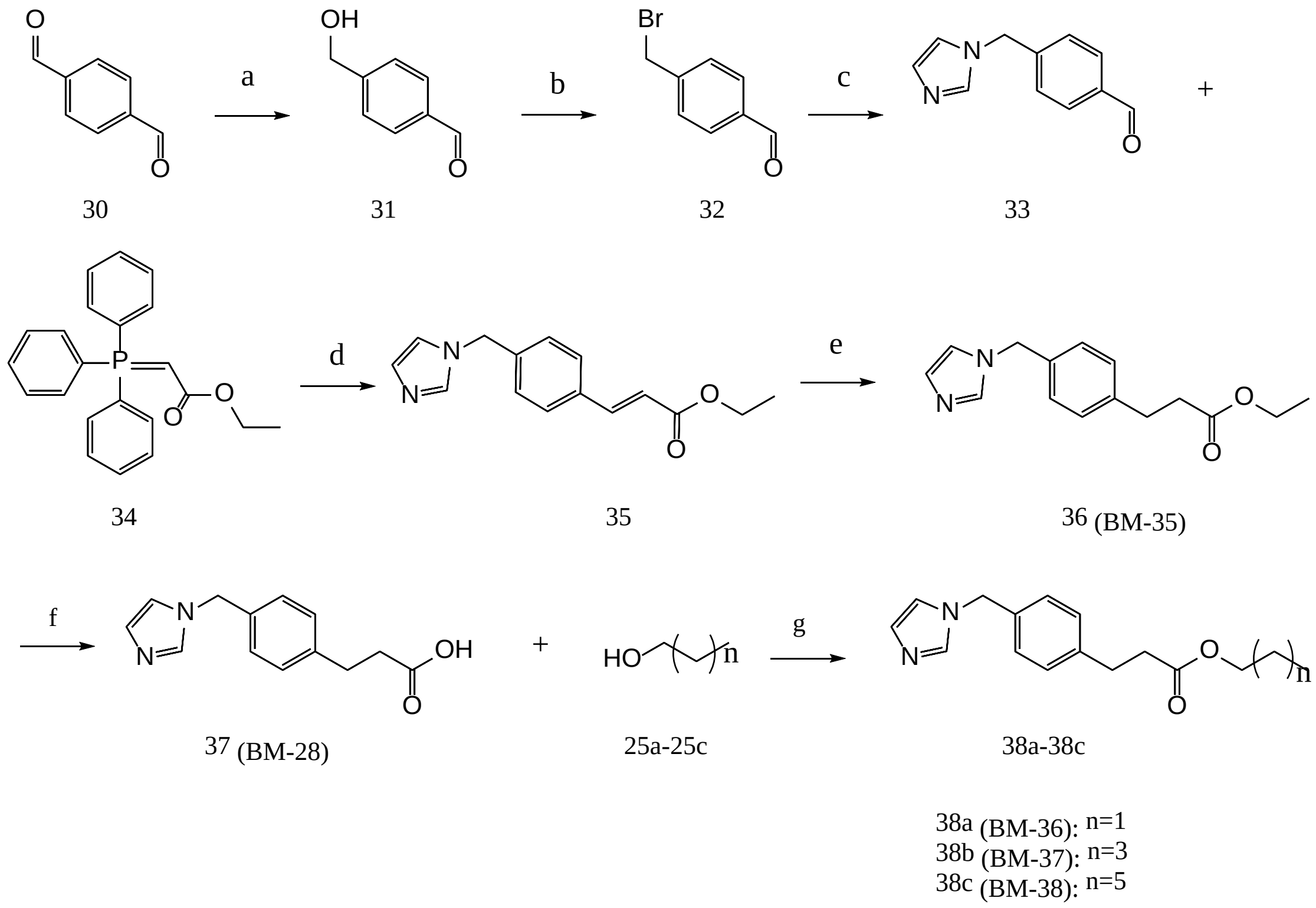

Reagents and conditions: (a) $NaBH_4$, THF, r.t, 1 h, 50%; (b) NBS,

Triphenylphosphine, DCM, r.t, 2 h, 83%; (c) K_2CO_3, DMF, 65℃, 2 h, 76%; (d) Toluene, 110℃, 8 h, 75%; (e) H_2, Pd/C, MeOH, r.t, 8 h, 89%; (f) NaOH, THF:H_2O=4:1, r.t, 2 h, 78%; (g) DMAP, EDCI, DMF, r.t, 2 h, 85%.

Scheme 12. Synthesis of compounds **41a - 41c**

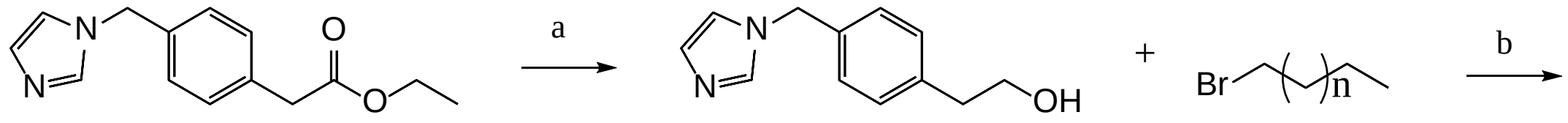

18b 39 40a-40c

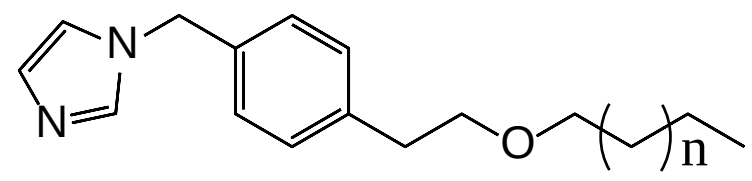

41a-41b

41a (BM-41): n=1
41b (BM-42): n=3
41c (BM-43): n=5

Reagents and conditions: (a) $LiAlH_4$, THF, r.t, 2 h, 88%; (b) NaH, DMF, Ar, r.t, 2 h, 50-60%.

Scheme 13. Synthesis of compound **44**

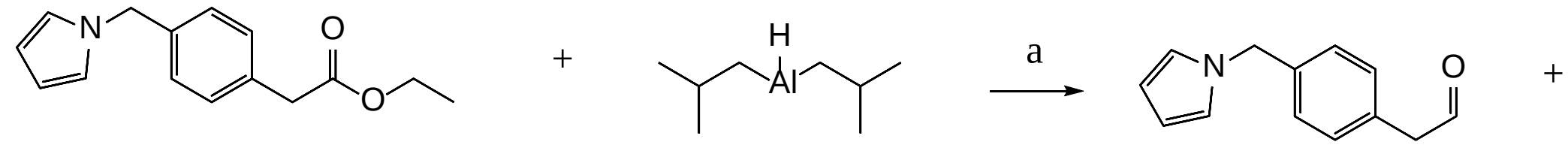

18b 41 42

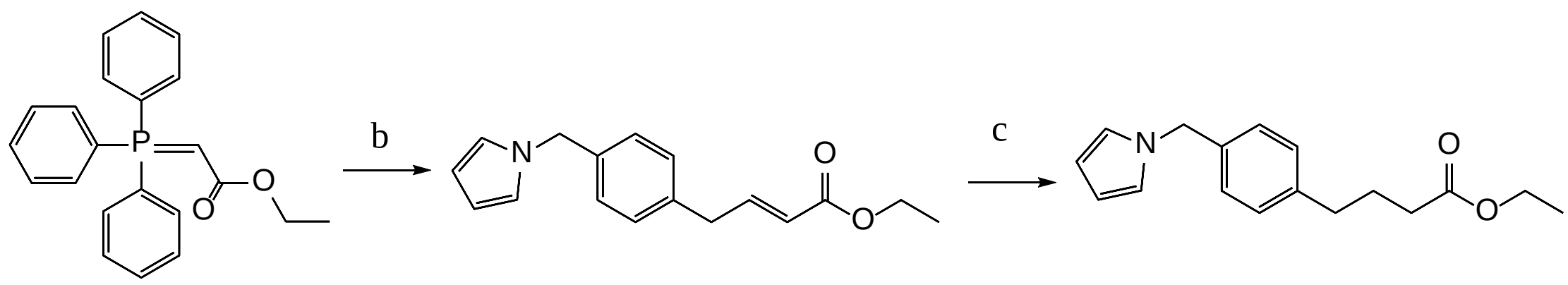

34 43 44 (BM-44)

Reagents and conditions: (a) DCM, Ar, -78℃, 2 h, 60%; (b) Toluene, 110℃, 8 h, 87%; (c) H_2, Pd/C, r.t, 8 h, 90%.

Scheme 14. Synthesis of compounds **47**, **48**, **52**, **and 53**.

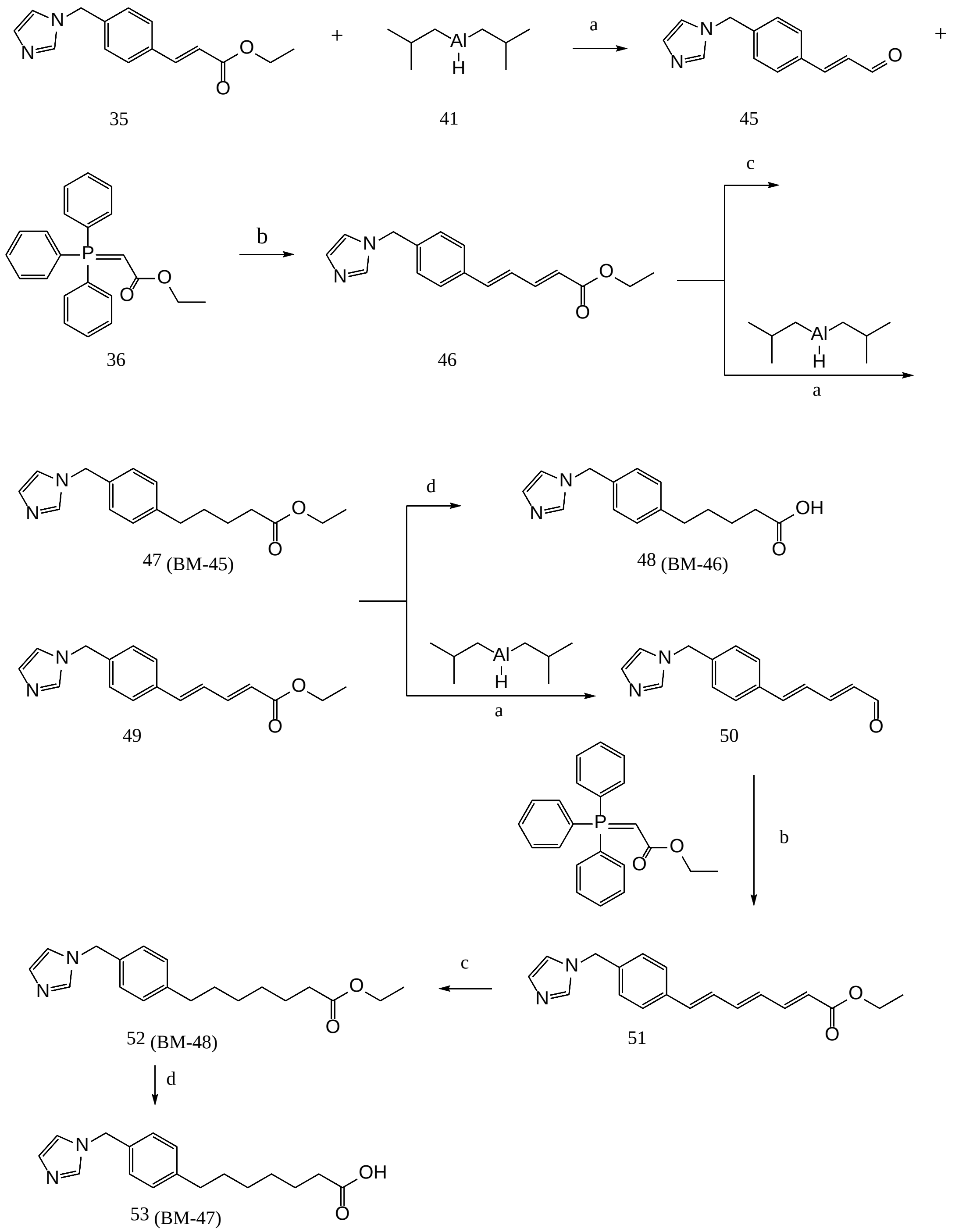

Reagents and conditions: (a) DCM, Ar, -78℃, 2h, 65%; (b) Toluene, 110℃, 8 h, 85%; (c) H_2, Pd/C, r.t, 8 h, 92%; (d) NaOH, THF:H_2O=4:1, r.t, 2 h, 86%.

Scheme 15. Synthesis of compounds **54a - 54c**.

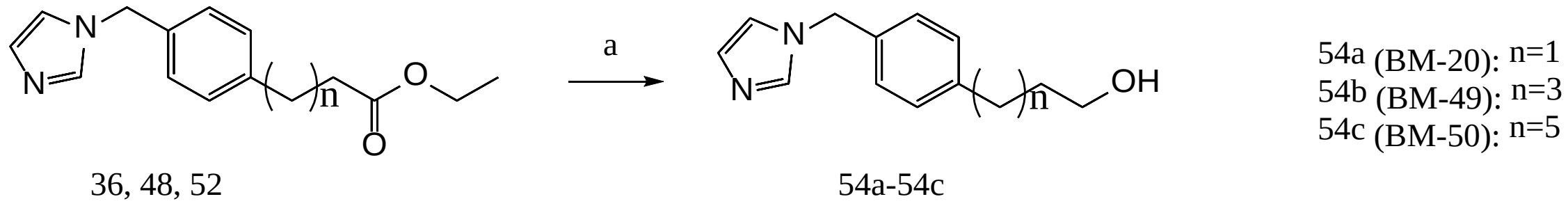

Reagents and conditions: (a) $LiAlH_4$, THF, r.t, 2 h, 85%.

Scheme 16. Synthesis of compound **59**.

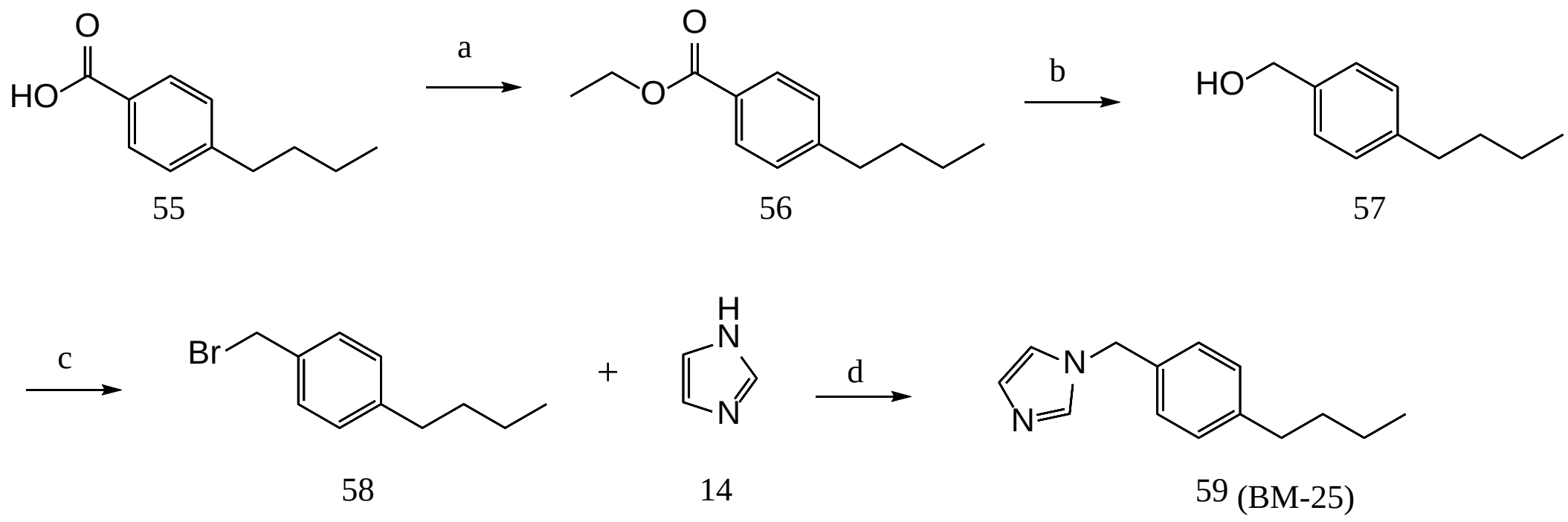

Reagents and conditions: (a) $SOCl_2$, EtOH, 80℃, 2 h, 96%; (b) $LiAlH_4$, THF, r.t, 2 h, 87%; (c) NBS, Triphenylphosphine, DCM, r.t, 2 h, 73%; (d) K_2CO_3, DMF, 65℃, 2 h, 64%.

Scheme 17. Synthesis of compounds **61a - 61c**.

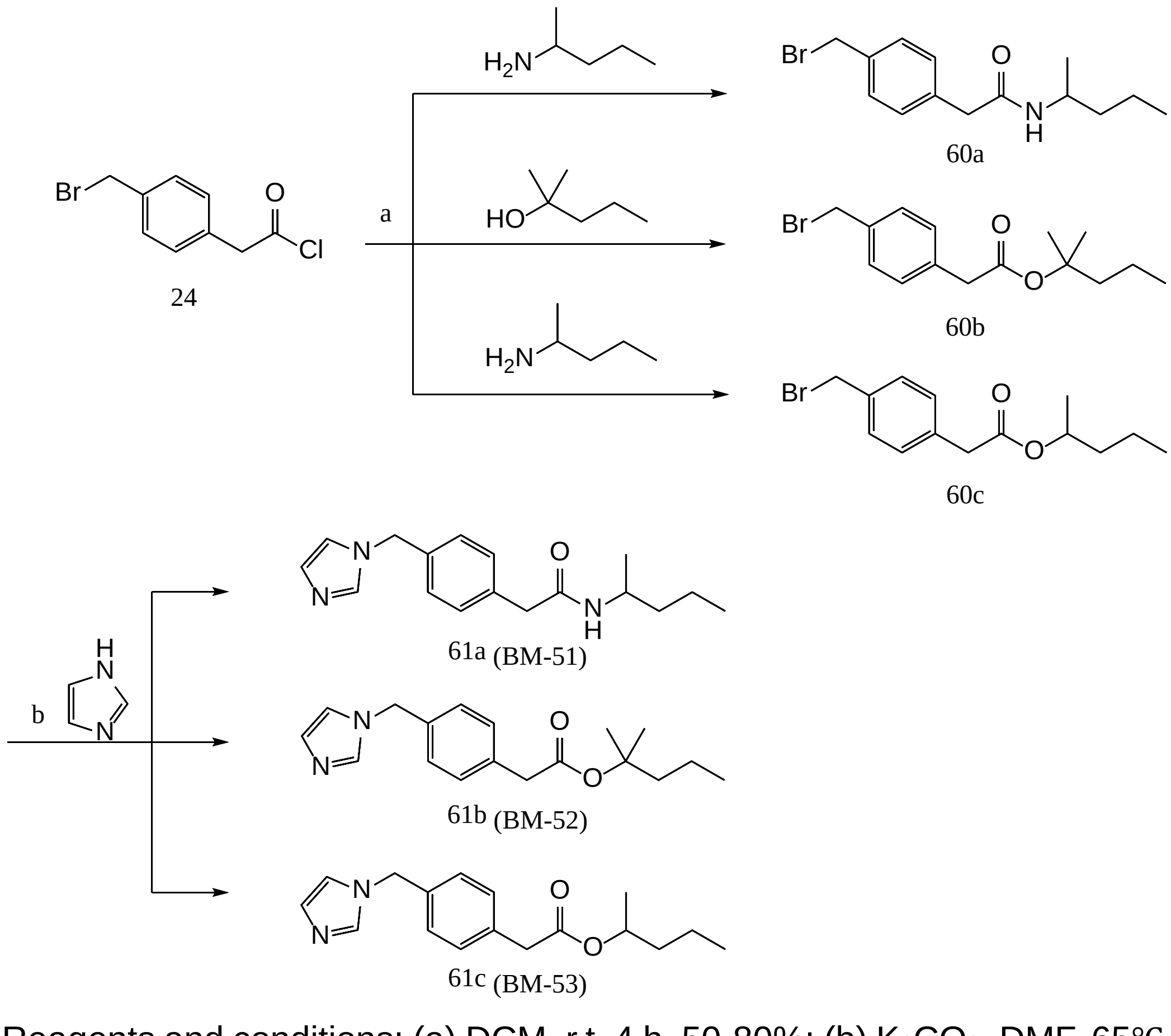

Reagents and conditions: (a) DCM, r.t, 4 h, 50-80%; (b) K_2CO_3, DMF, 65℃, 2 h, 58-78%.

Scheme 18. Synthesis of compounds **64** and **66**.

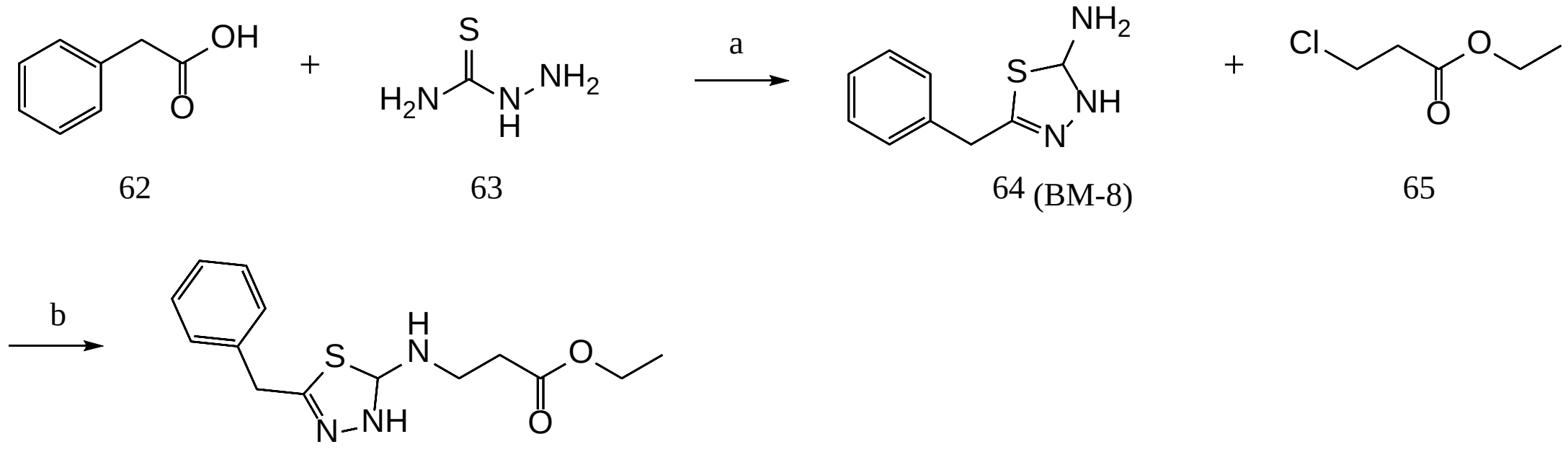

62 63 64 (BM-8) 65

66 (BM-9)

Reagents and conditions: (a) $POCl_3$, 80℃, 4 h, 48%; (b) NaH, THF, r.t, 4 h, 60%.

Scheme 19. Synthesis of compound **68**.

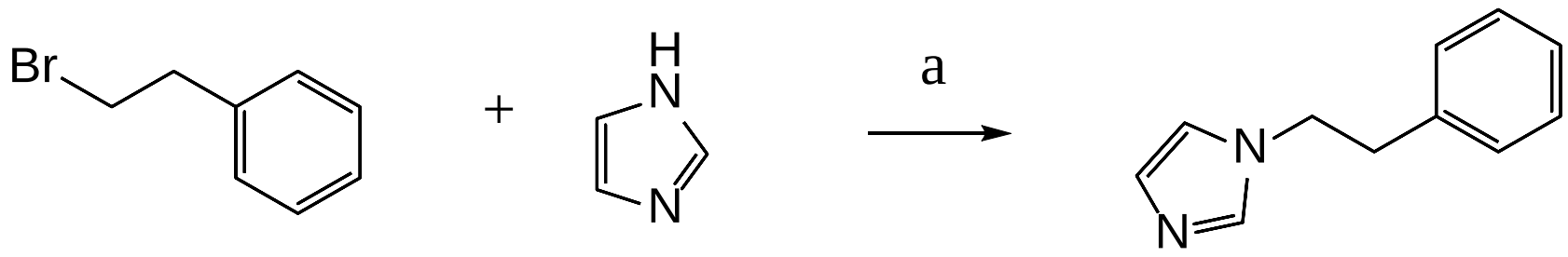

67 14 68 (BM-23)

Reagents and conditions: (a) K_2CO_3, THF, 70℃, 2 h, 67%.

^{1}H NMR, and ^{13}C NMR spectra of all title compounds

BM-1

^{1}H NMR

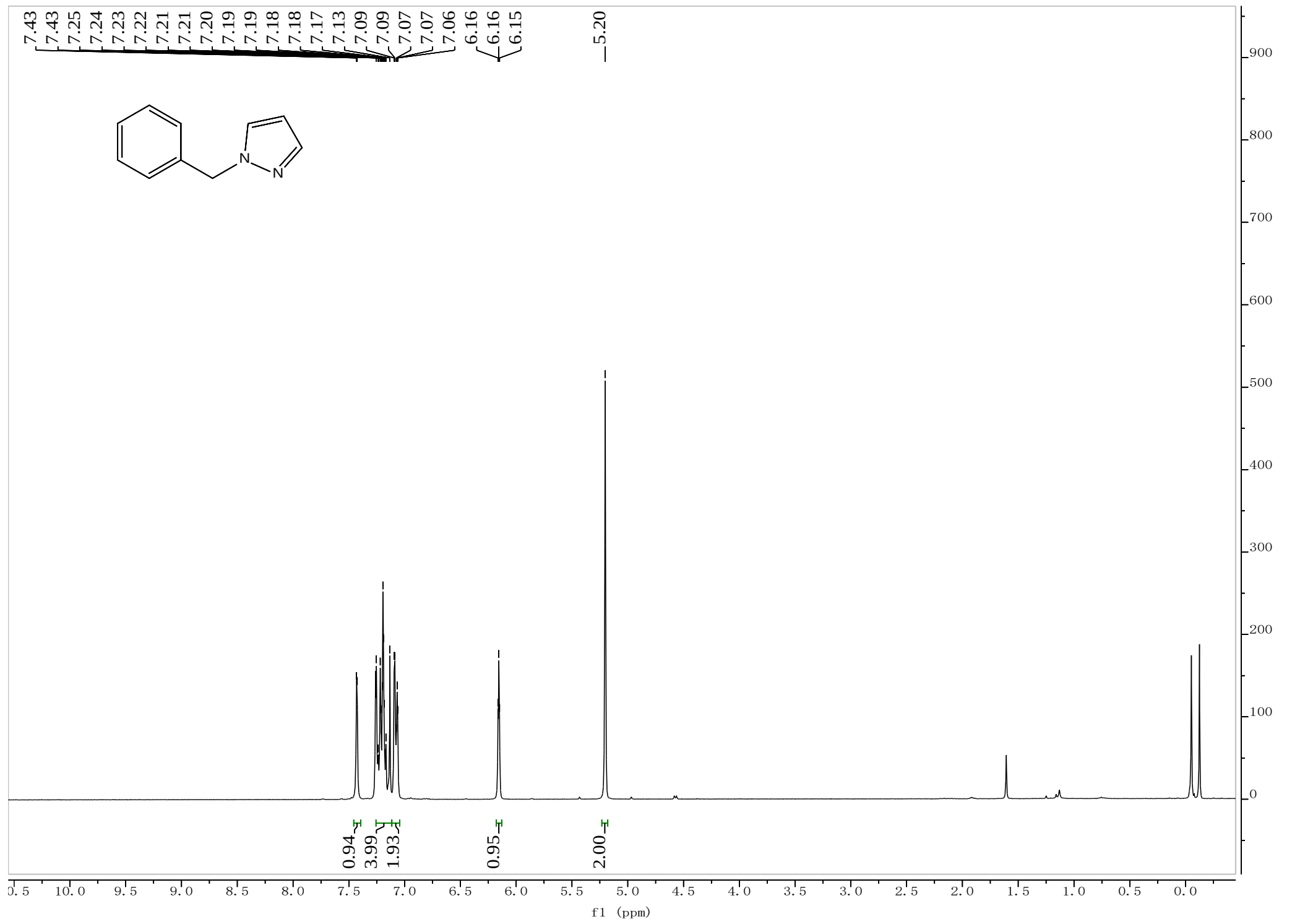

^{1}H NMR (300 MHz, Chloroform-*d*) δ 7.43 (d, J = 1.9 Hz, 1H), 7.26-7.11 (m, 4H), 7.08 (dd, J = 7.8, 1.8 Hz, 2H), 6.16 (t, J = 2.1 Hz, 1H), 5.20 (s, 2H).

^{13}C NMR

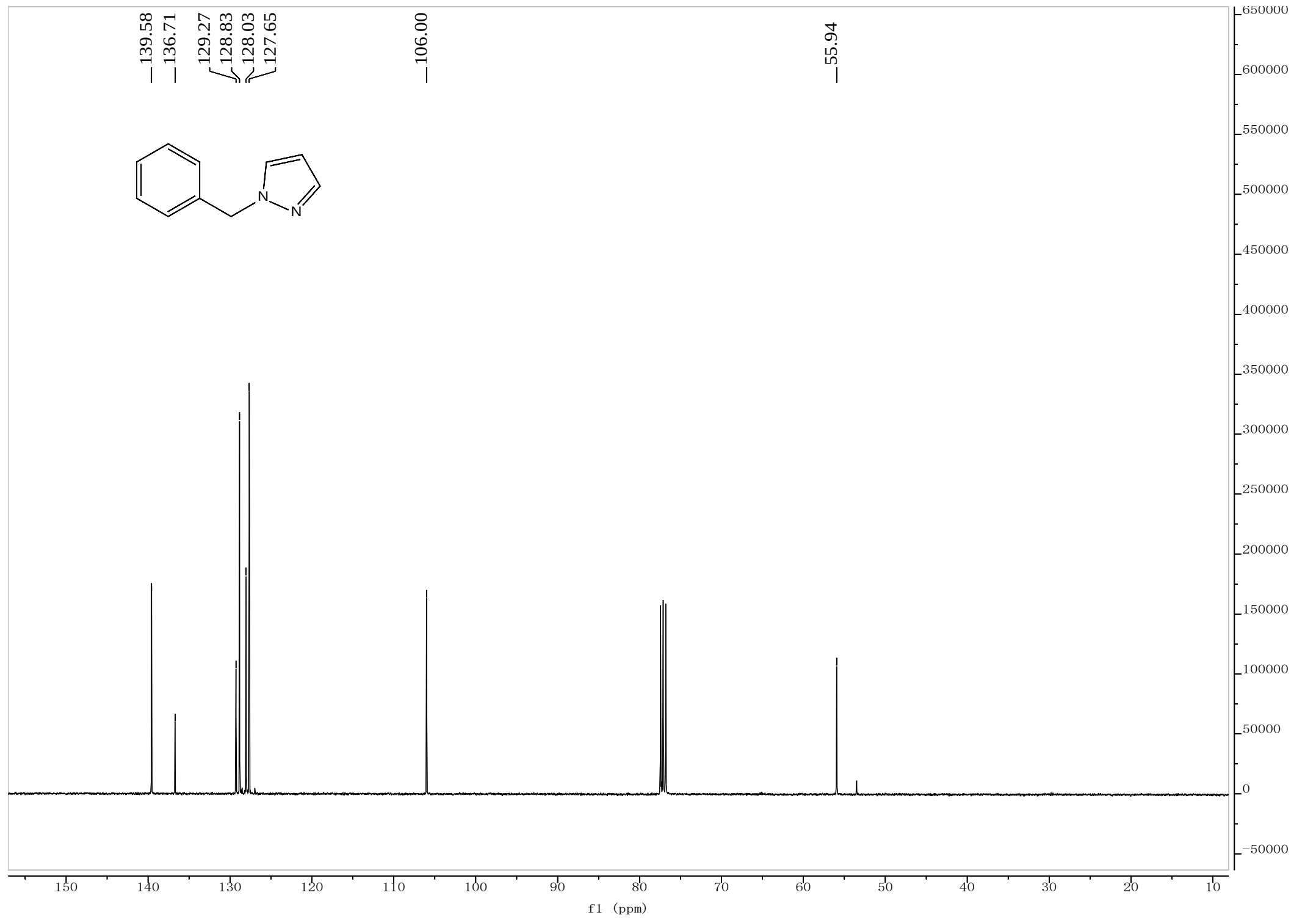

^{13}C NMR (101 MHz, Chloroform-*d*) δ 139.58, 136.71, 129.27, 128.83, 128.03, 127.65, 106.00, 55.94.

BM-2

^{1}H-NMR

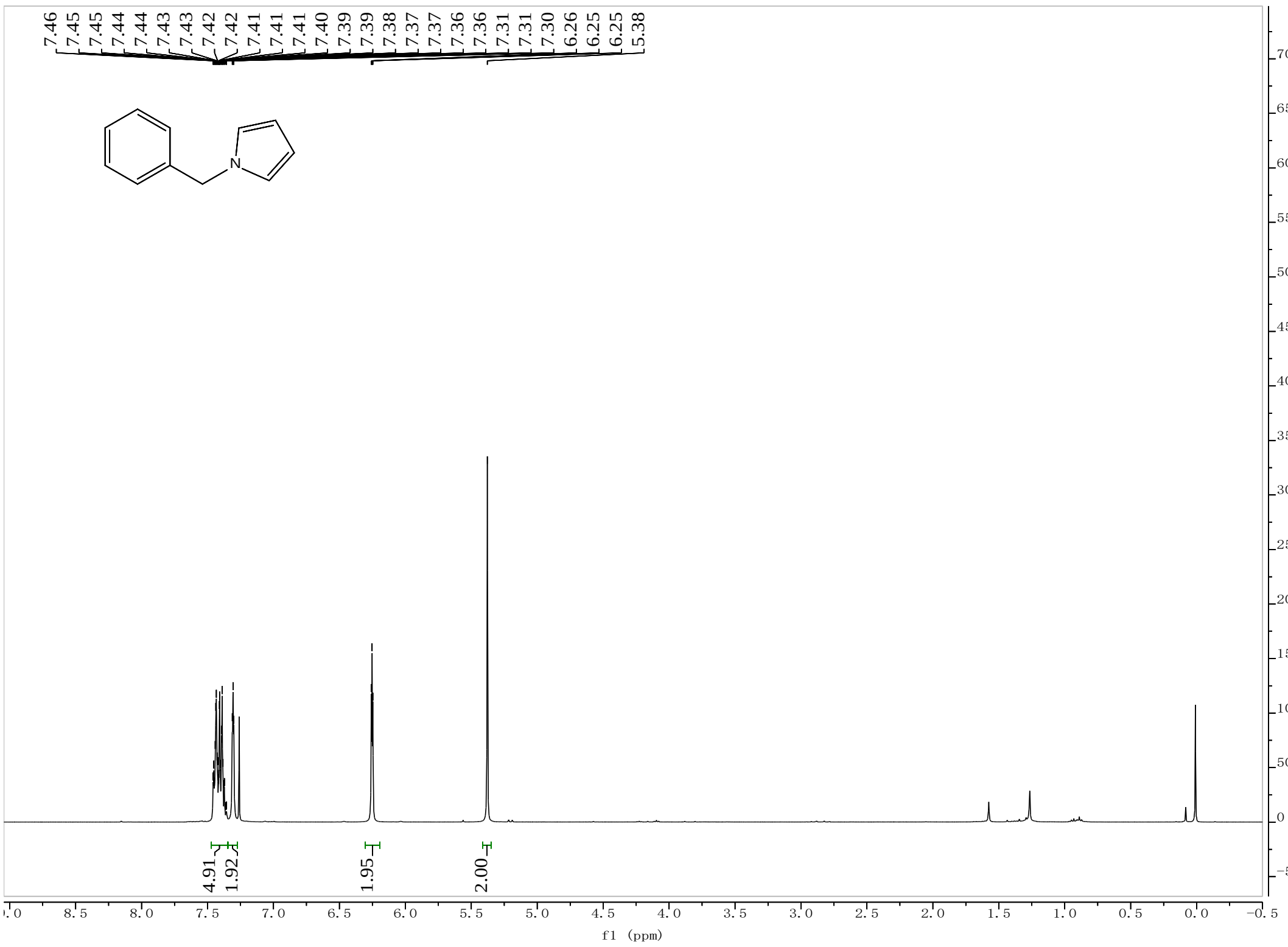

^{1}H NMR (400 MHz, Chloroform-*d*) *δ* 7.47-7.35 (m, 5H), 7.34-7.27 (m, 2H), 6.30-6.19 (m, 2H), 5.38 (s, 2H).

^{13}C-NMR

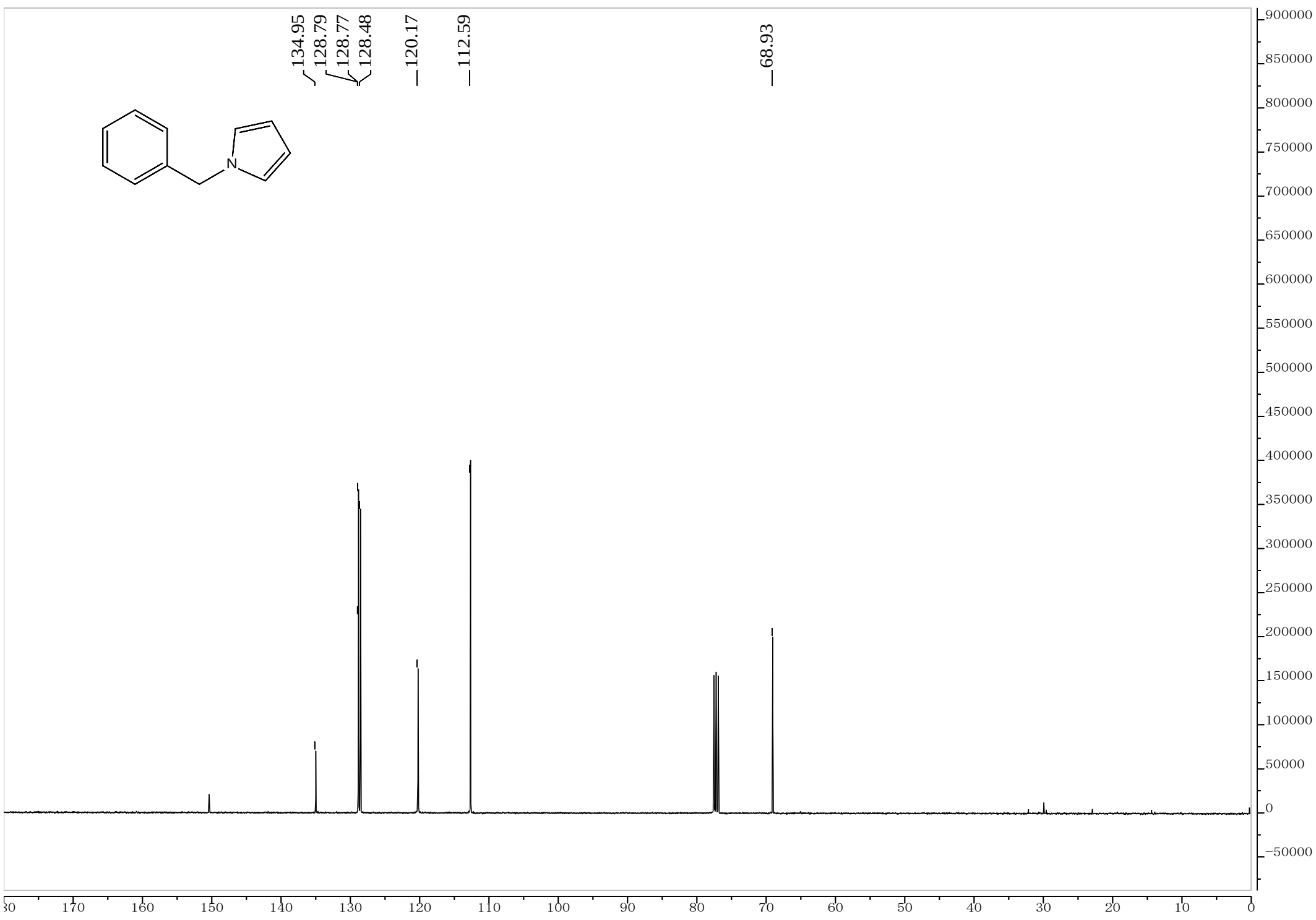

^{13}C NMR (101 MHz, Chloroform-*d*) *δ* 134.95, 128.79, 128.77, 128.48, 120.17,

112.59, 68.93.

BM-3

^{1}H NMR

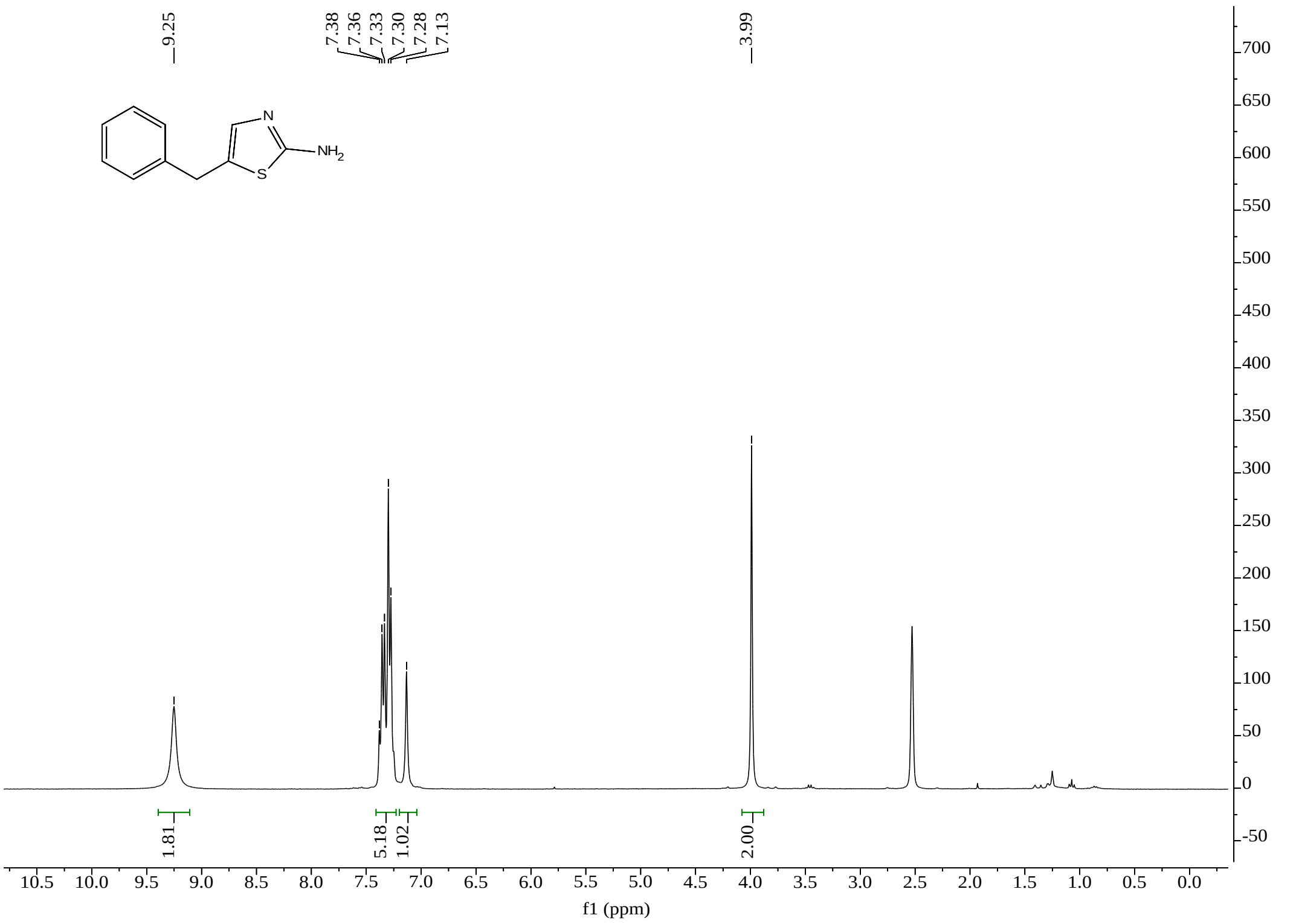

^{1}H NMR (300 MHz, DMSO-d_6) δ 9.25 (s, 2H), 7.32 (dd, J = 17.2, 6.9 Hz, 5H), 7.13 (s, 1H), 3.99 (s, 2H).

^{13}C NMR

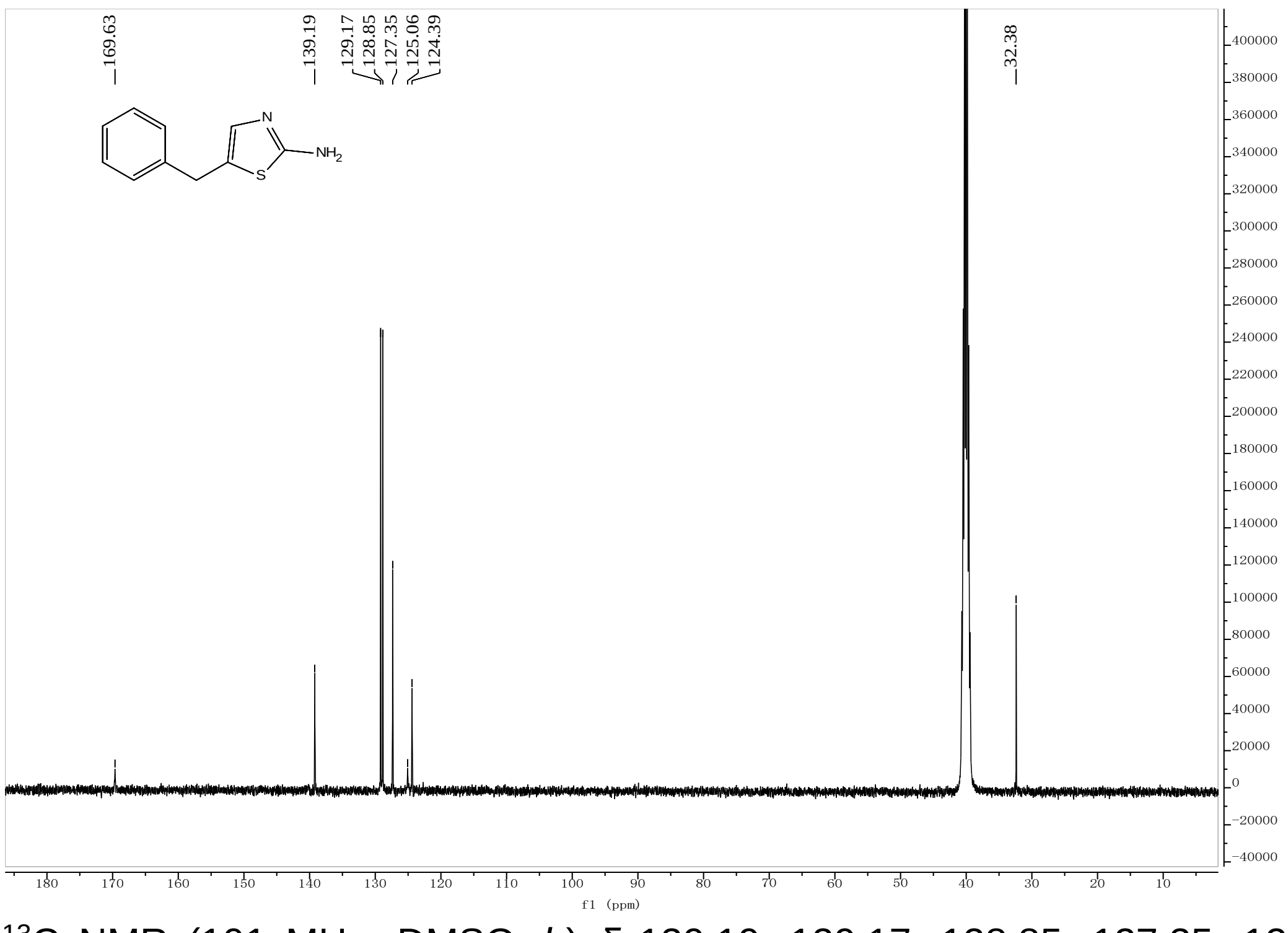

^{13}C NMR (101 MHz, DMSO-d_6) δ 139.19, 129.17, 128.85, 127.35, 124.39, 32.38.

BM-4

^{1}H-NMR

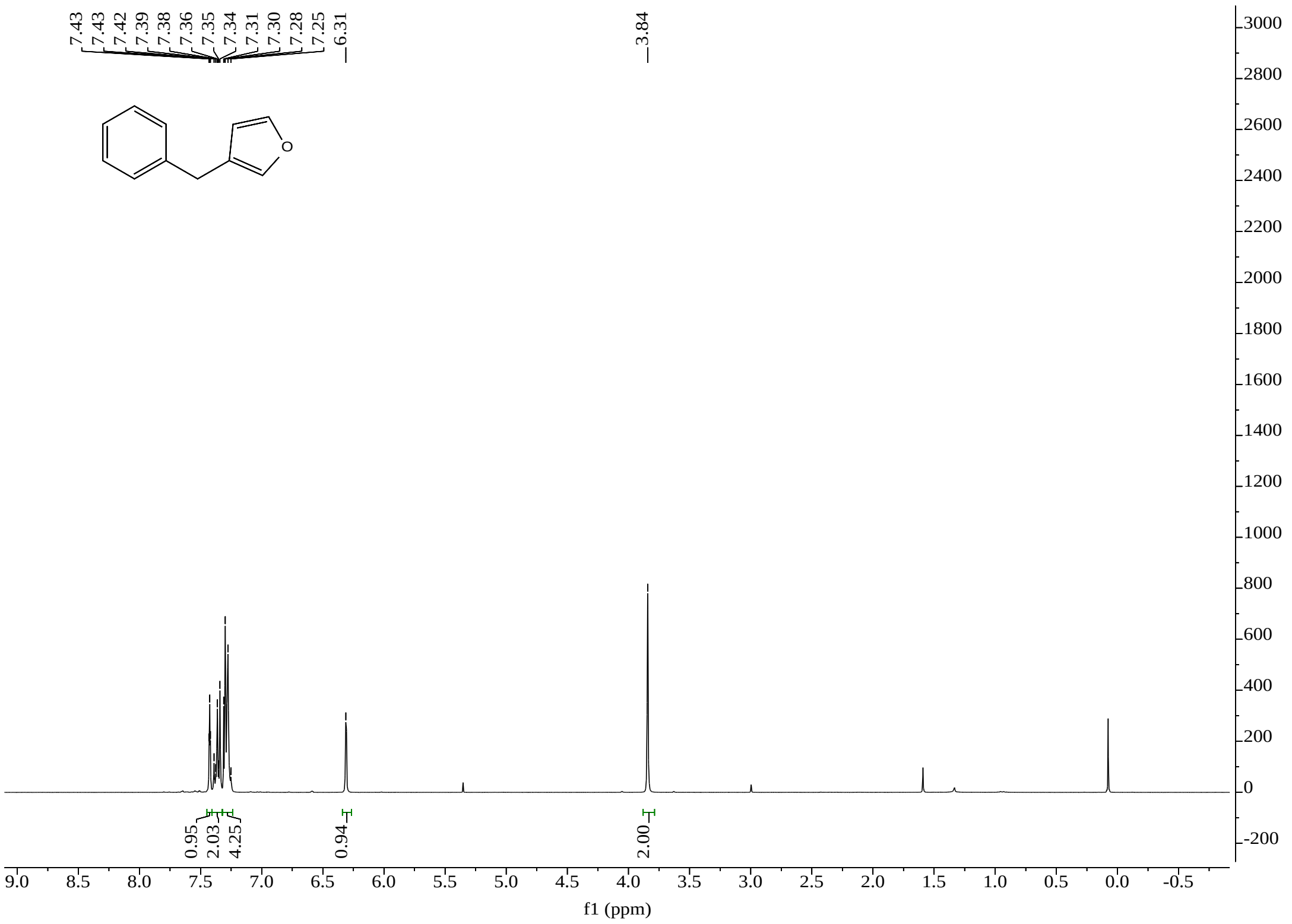

^{1}H NMR (300 MHz, Chloroform-*d*) δ 7.43 (t, J = 1.6 Hz, 1H), 7.41-7.32 (m, 2H), 7.32-7.24 (m, 4H), 6.31 (s, 1H), 3.84 (s, 2H).

^{13}C NMR

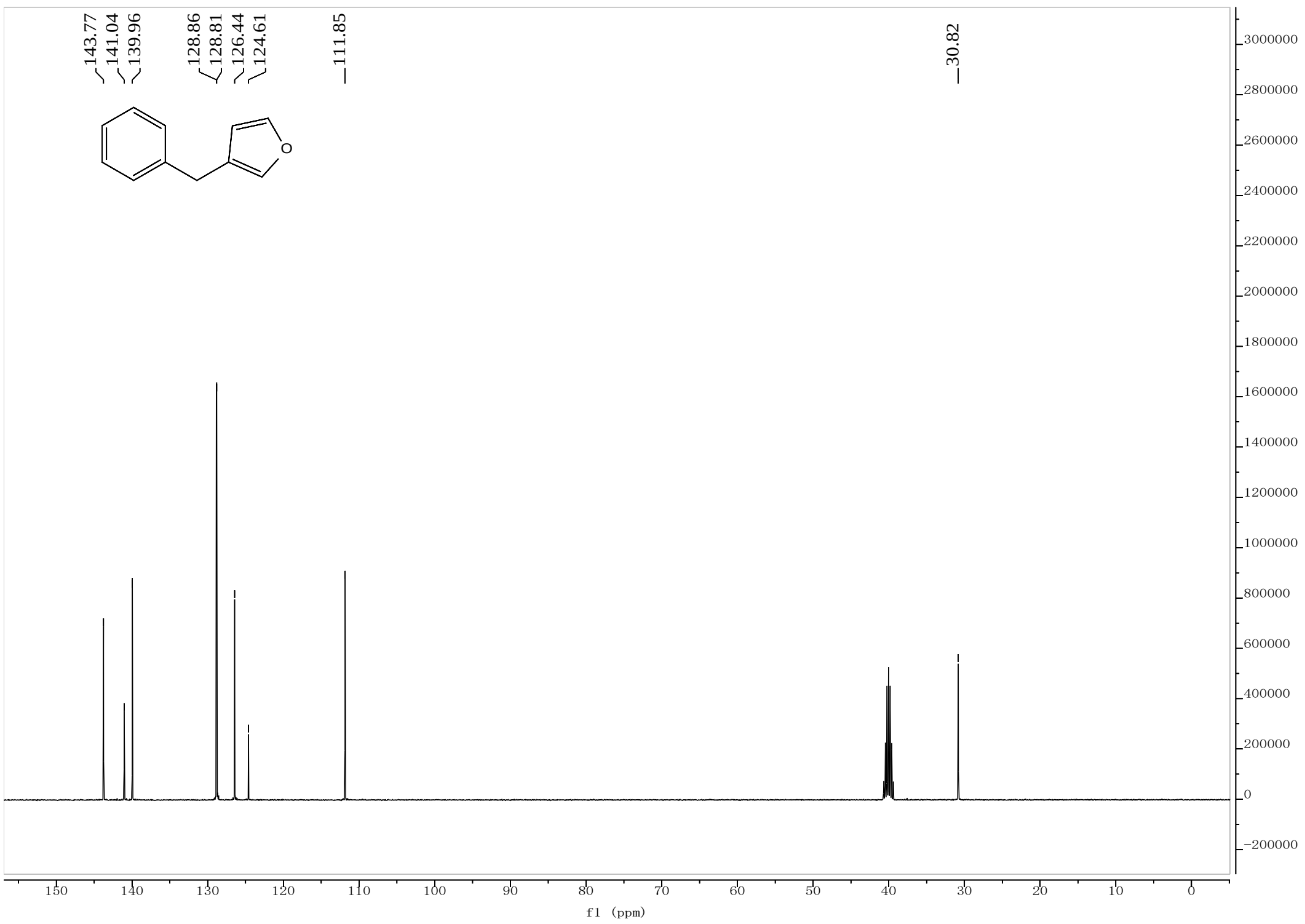

^{13}C NMR (101 MHz, DMSO-d_6) δ 143.77, 141.04, 139.96, 128.86, 128.81, 126.44, 124.61, 111.85, 30.82.

BM-5

^{1}H-NMR

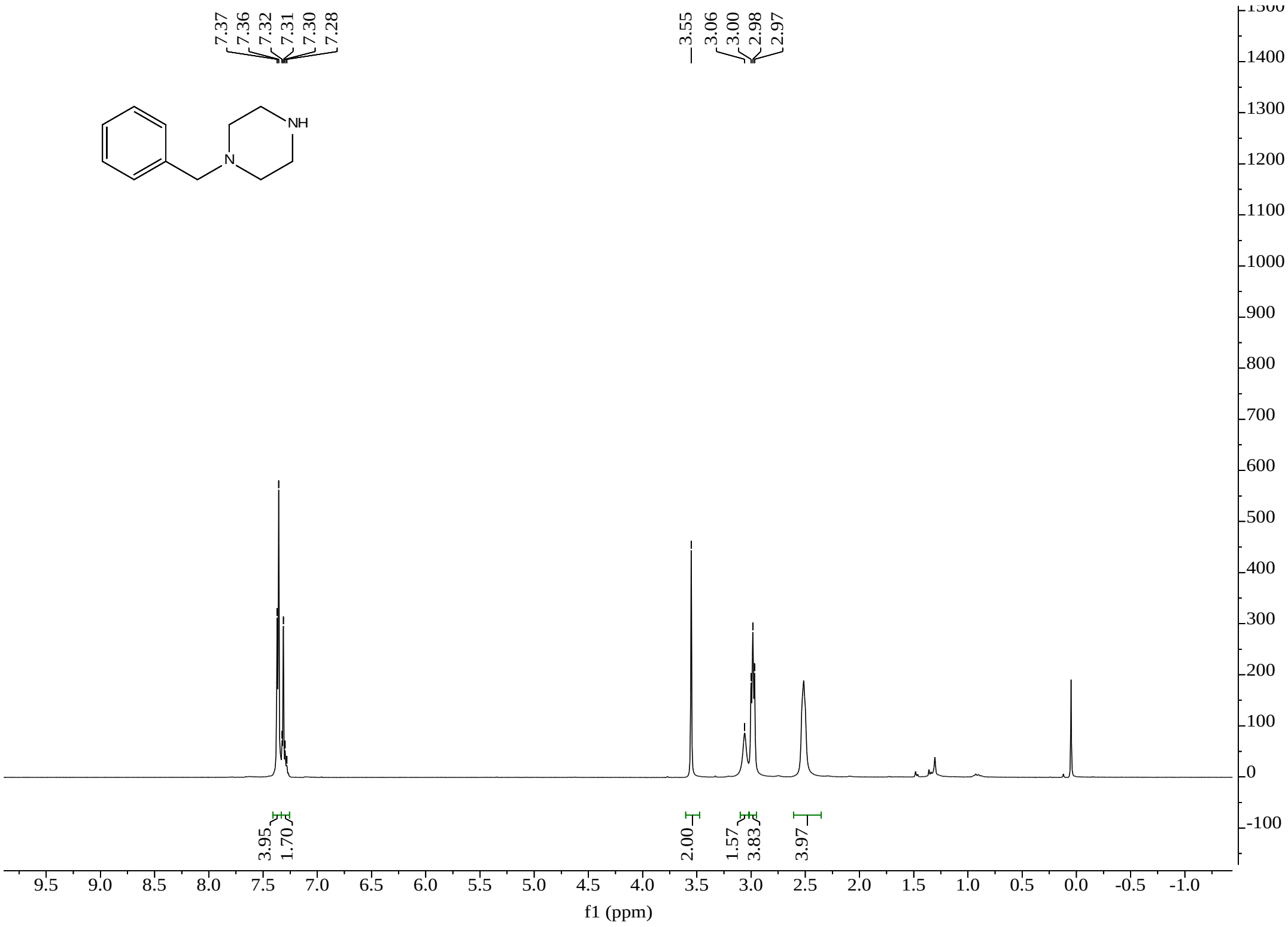

^{1}H NMR (300 MHz, Chloroform-*d*) δ 7.36 (d, *J* = 4.2 Hz, 4H), 7.30 (q, *J* = 4.9, 4.2 Hz, 2H), 3.55 (s, 2H), 3.06 (s, 2H), 3.02-2.95 (m, 4H), 2.51 (t, *J* = 4.9 Hz, 4H).

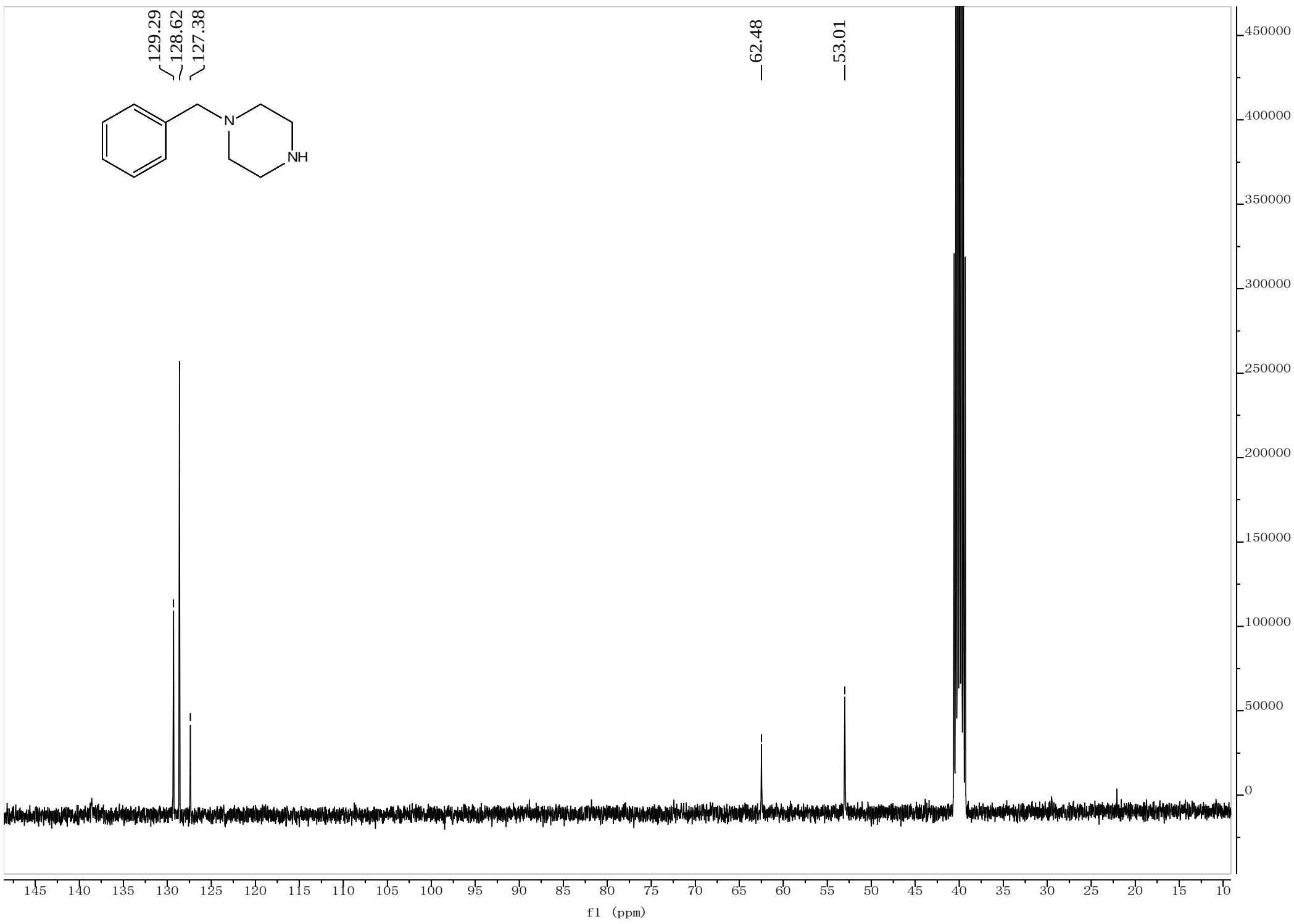

^{13}C NMR

^{13}C NMR (101 MHz, DMSO-d_6) δ 129.29, 128.62, 127.38, 62.48.

BM-6

^{1}H-NMR

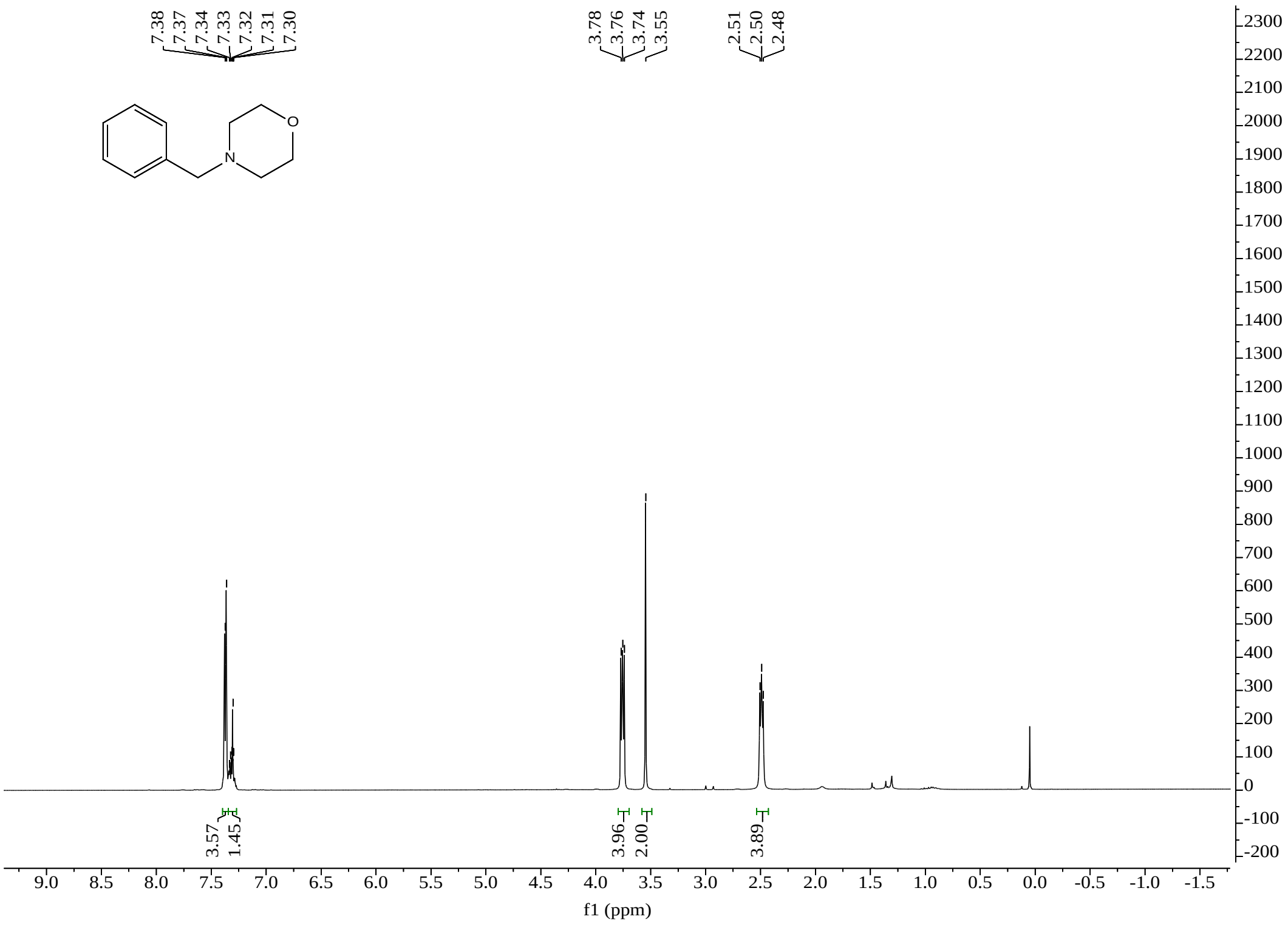

^{1}H NMR (300 MHz, Chloroform-*d*) *δ* 7.37 (d, J = 4.3 Hz, 4H), 7.35-7.28 (m, 1H), 3.80-3.70 (m, 4H), 3.55 (s, 2H), 2.54-2.43 (m, 4H).

^{13}C NMR

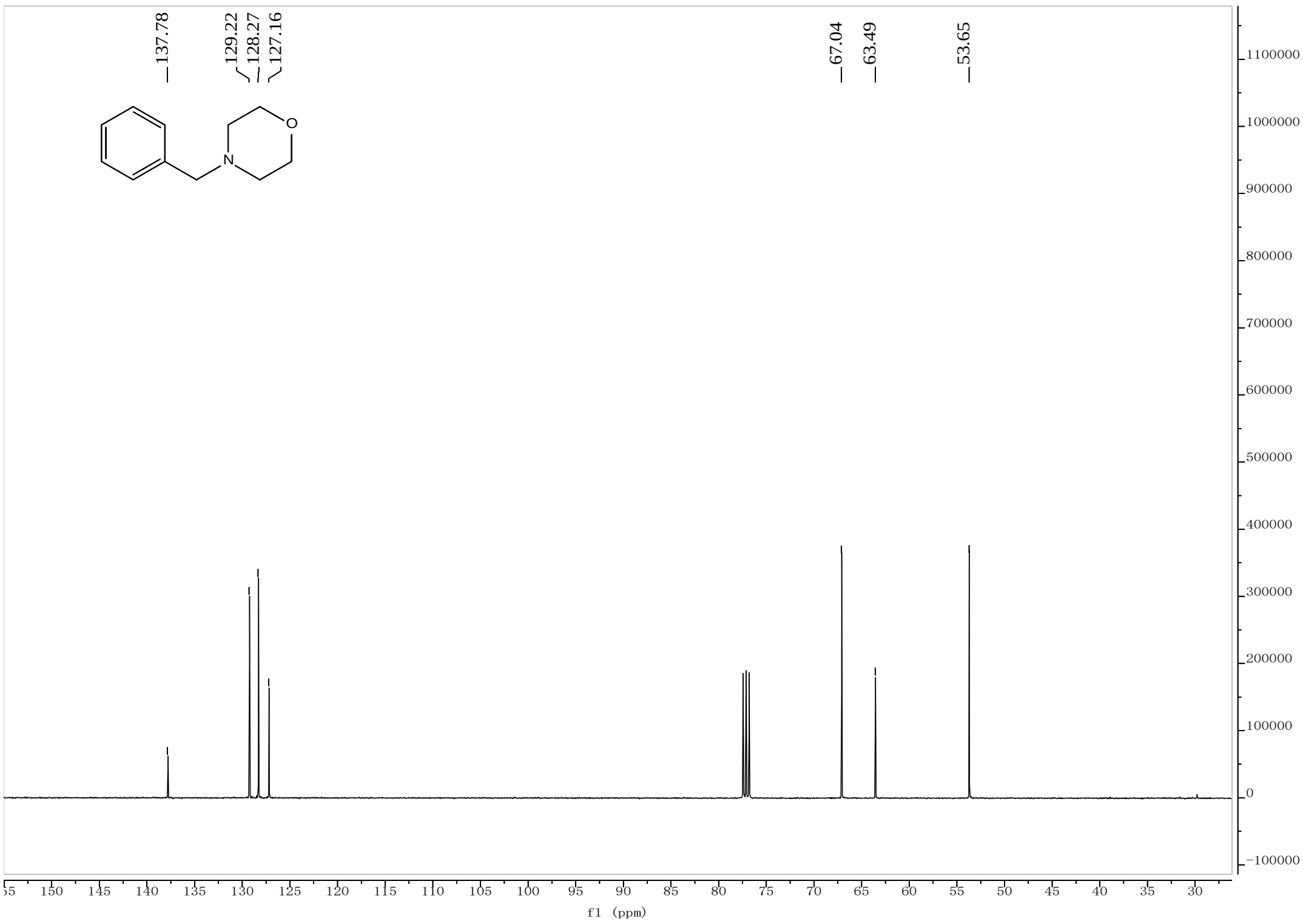

^{13}C NMR (101 MHz, Chloroform-*d*) *δ* 137.78, 129.22, 128.27, 127.16, 67.04, 63.49, 53.65.

BM-7

^{1}H NMR

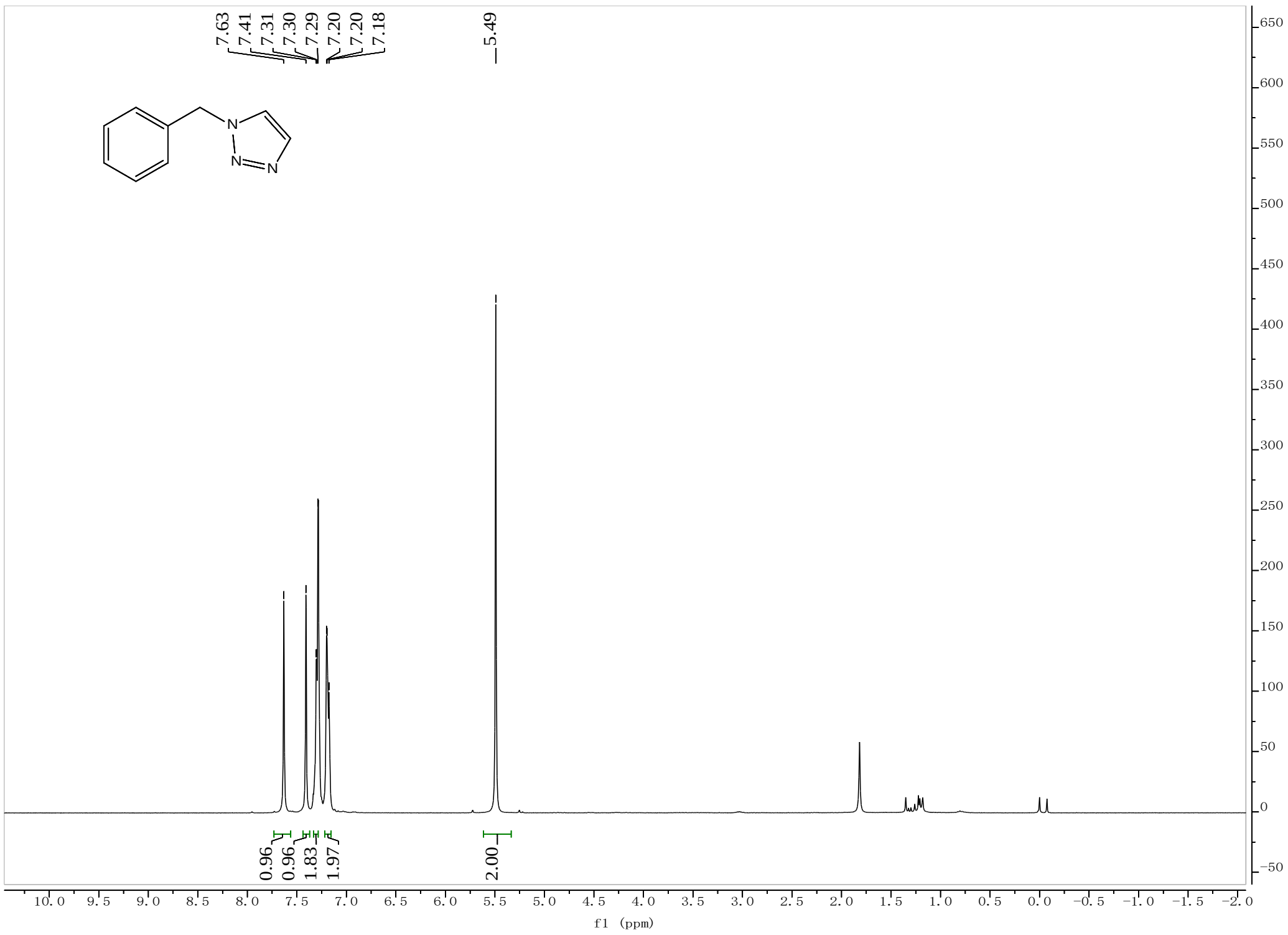

^{1}H NMR (300 MHz, Chloroform-*d*) δ 7.63 (s, 1H), 7.41 (s, 1H), 7.33-7.28 (m, 2H), 7.19 (d, J = 7.4 Hz, 2H), 5.49 (s, 2H).

^{13}C NMR

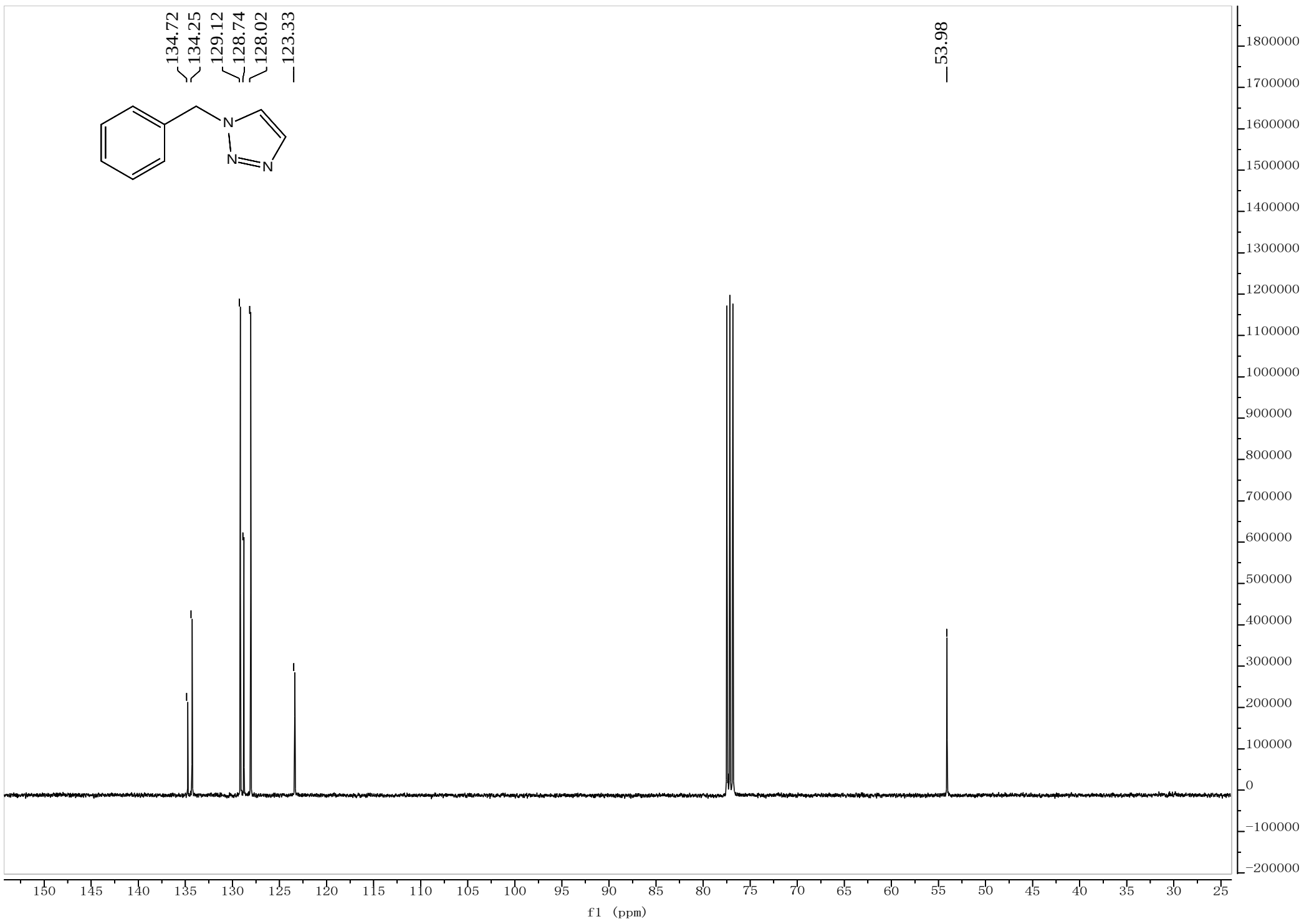

^{13}C NMR (101 MHz, Chloroform-*d*) δ 134.72, 134.25, 129.12, 128.74, 128.02, 123.33, 53.98.

BM-8

^{1}H NMR

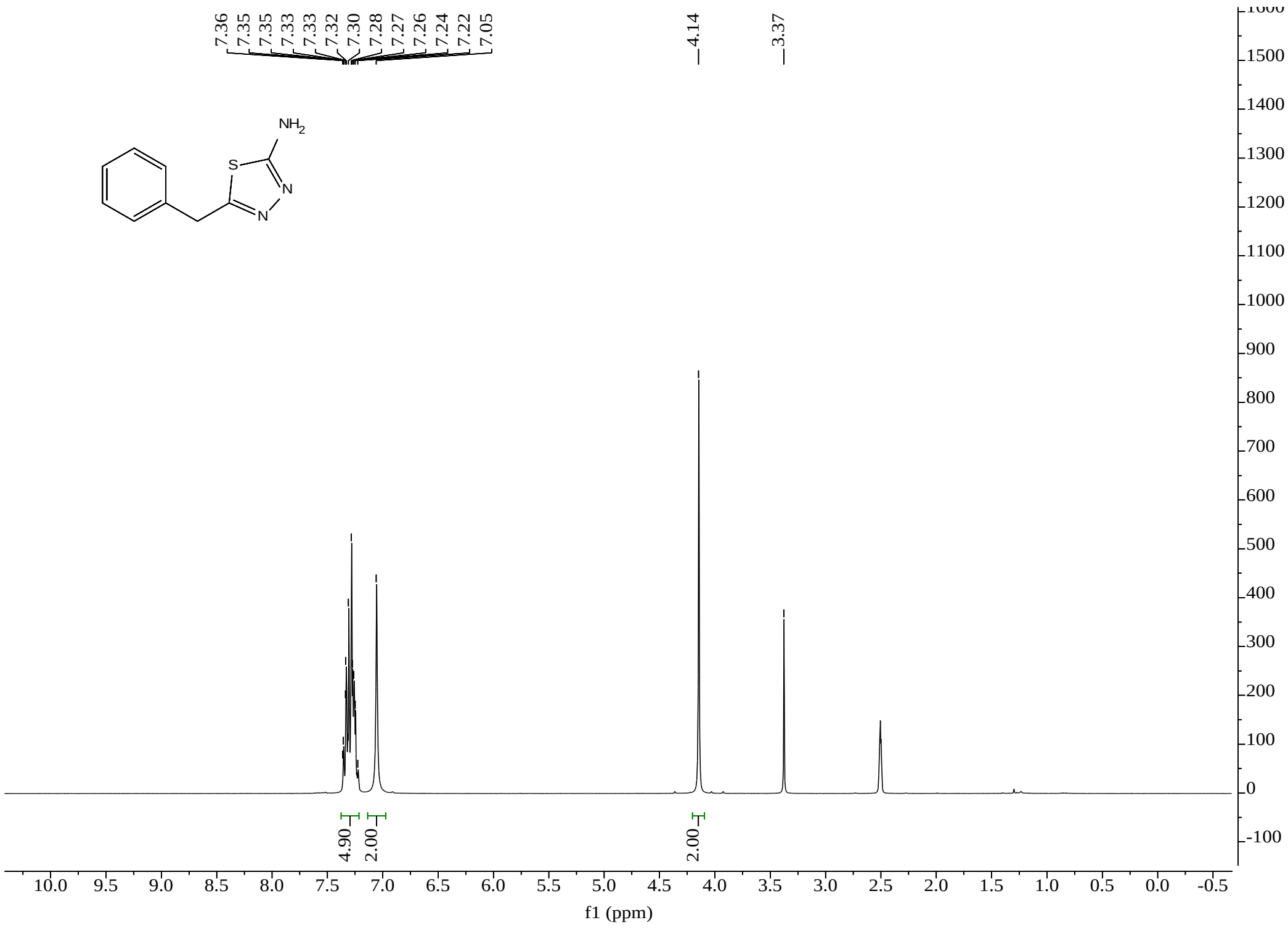

^{1}H NMR (300 MHz, DMSO-d_6) δ 7.37-7.21 (m, 5H), 7.05 (s, 2H), 4.14 (s, 2H).

^{13}C NMR

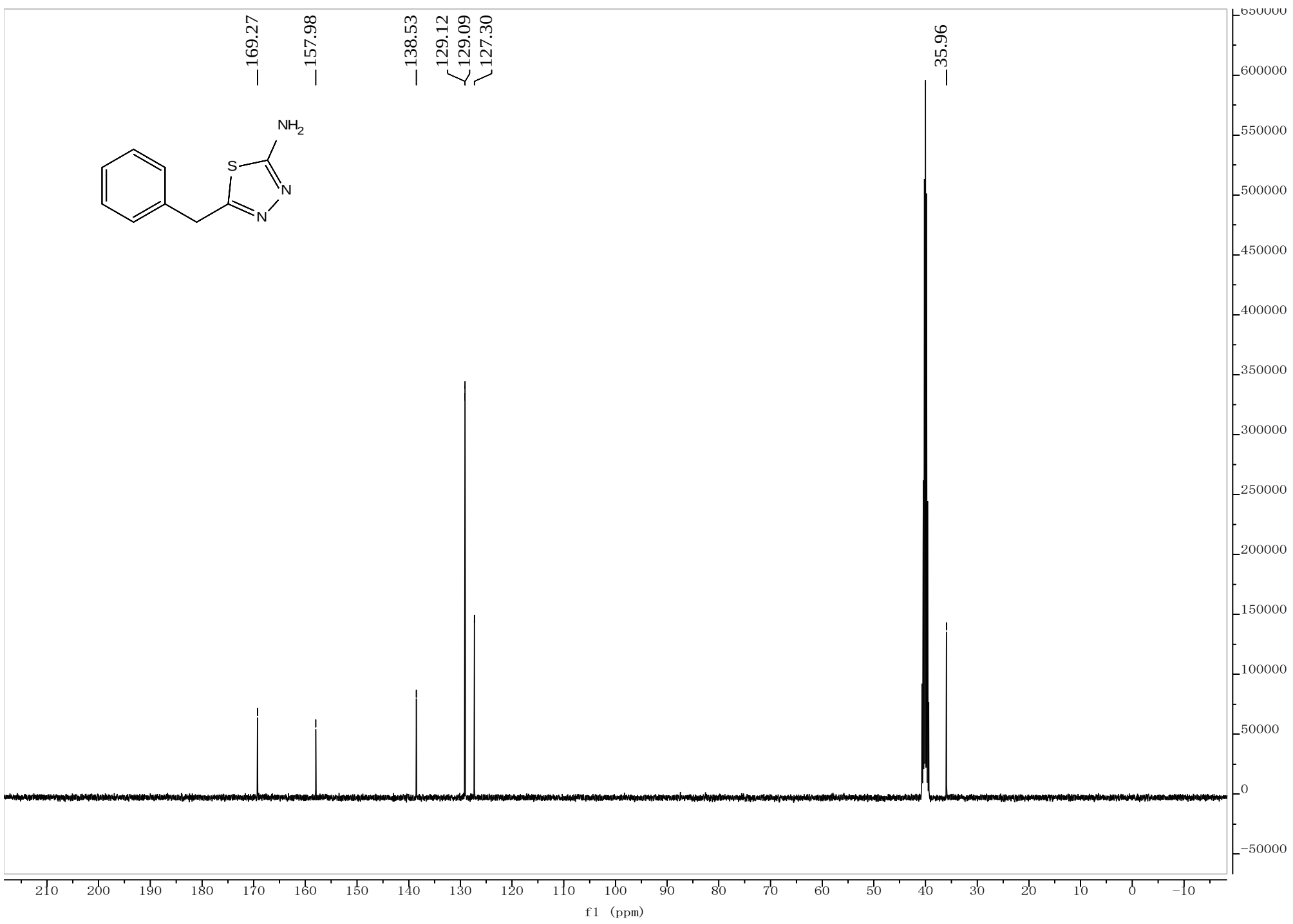

^{13}C NMR (101 MHz, DMSO-d_6) δ 169.27, 157.98, 138.53, 129.12, 129.09, 127.30, 35.96.

BM-9

^{1}H NMR

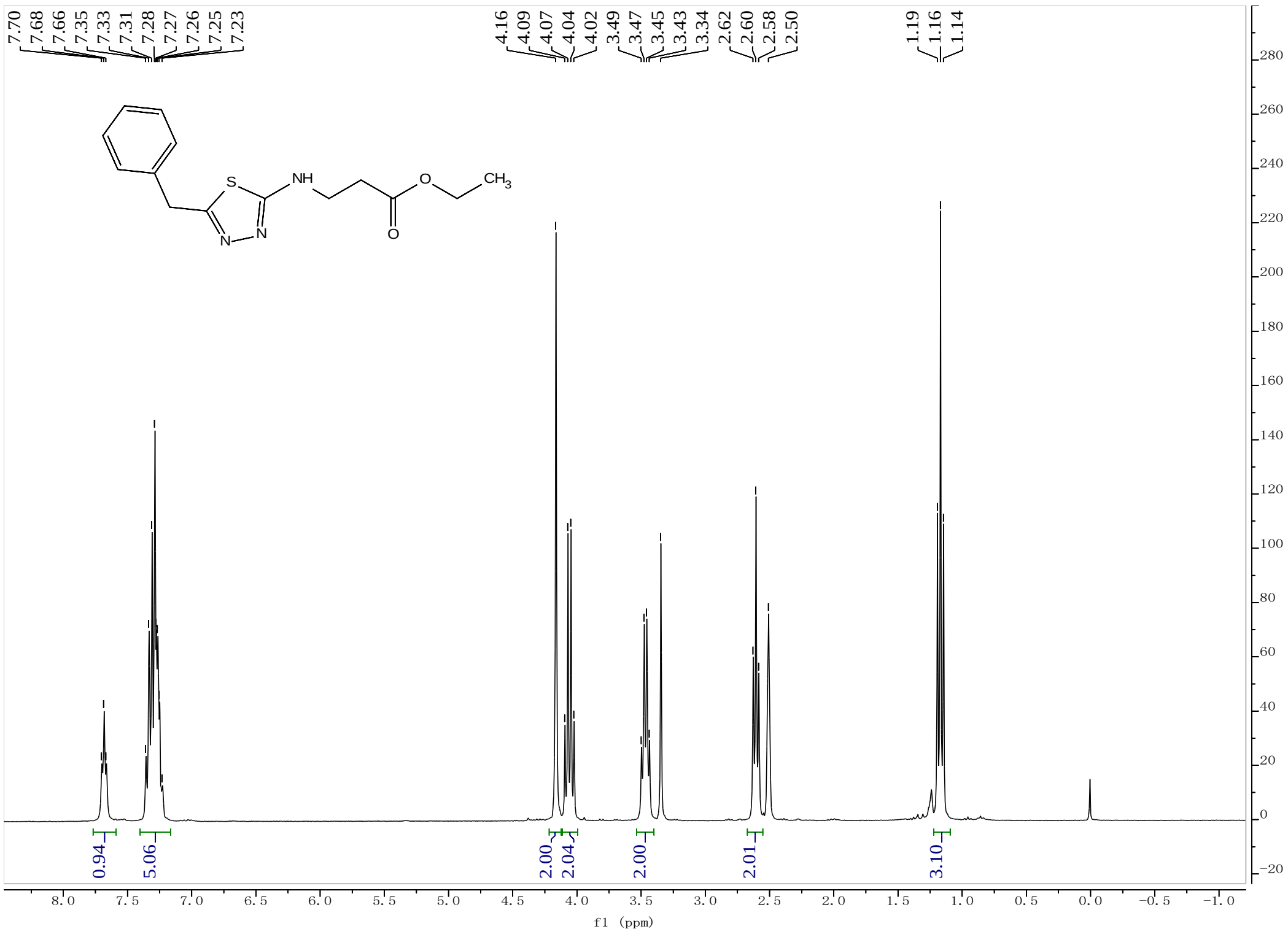

^{1}H NMR (300 MHz, Chloroform-*d*) *δ* 7.68 (t, *J* = 5.4 Hz, 1H), 7.40-7.16 (m, 5H), 4.16 (s, 2H), 4.05 (q, *J* = 7.1 Hz, 2H), 3.46 (q, *J* = 6.4 Hz, 2H), 2.60 (t, *J* = 6.6 Hz, 2H), 1.16 (t, *J* = 7.1 Hz, 3H).

^{13}C NMR

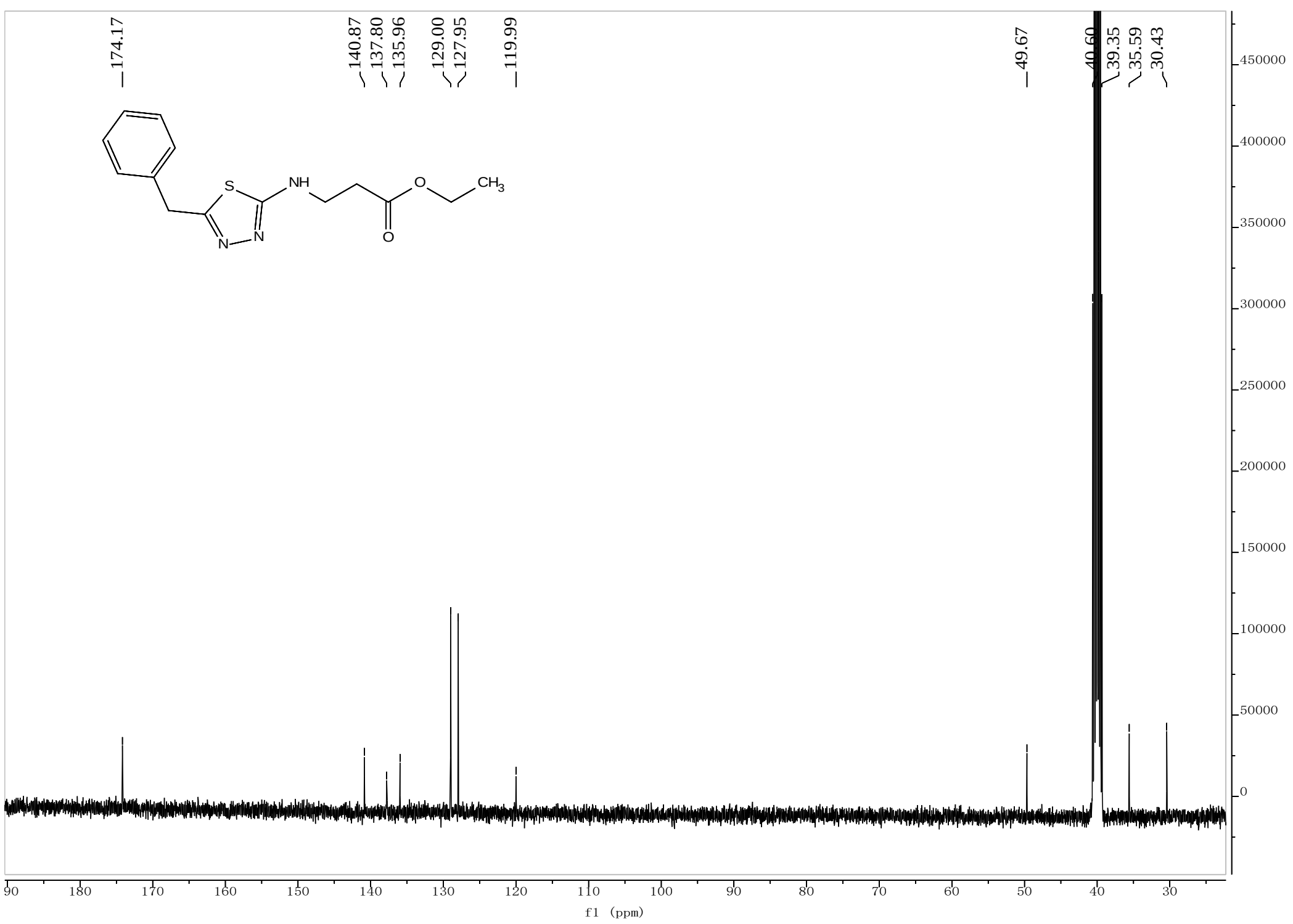

^{13}C NMR (101 MHz, DMSO-d_6) *δ* 174.17, 135.96, 129.00, 127.95, 119.99, 49.67, 40.60, 39.35, 35.59, 30.43.

BM-10

^{1}H-NMR

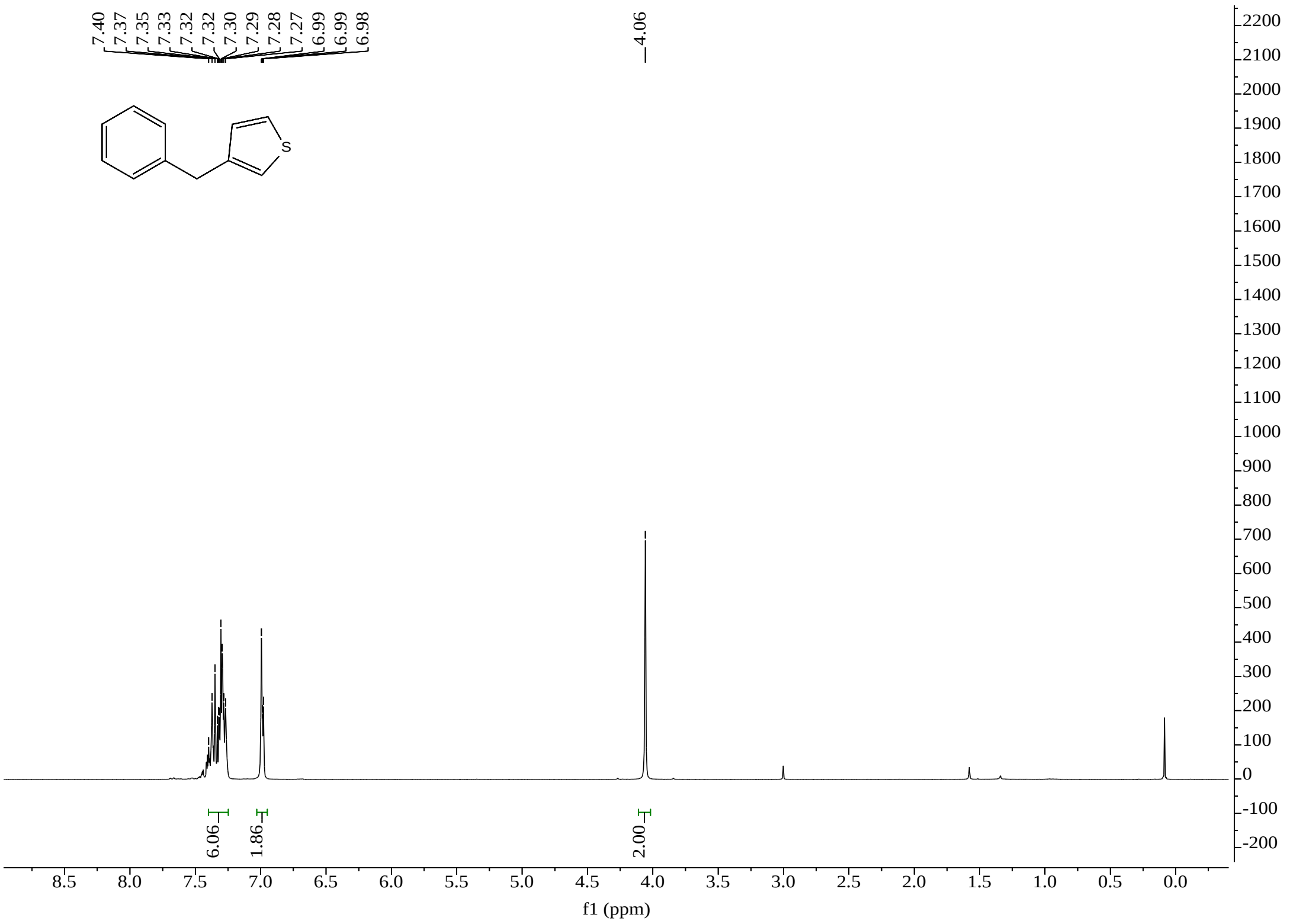

^{1}H NMR (300 MHz, Chloroform-*d*) *δ* 7.40-7.25 (m, 6H), 7.03-6.95 (m, 2H), 4.06 (s, 2H).

^{13}C NMR

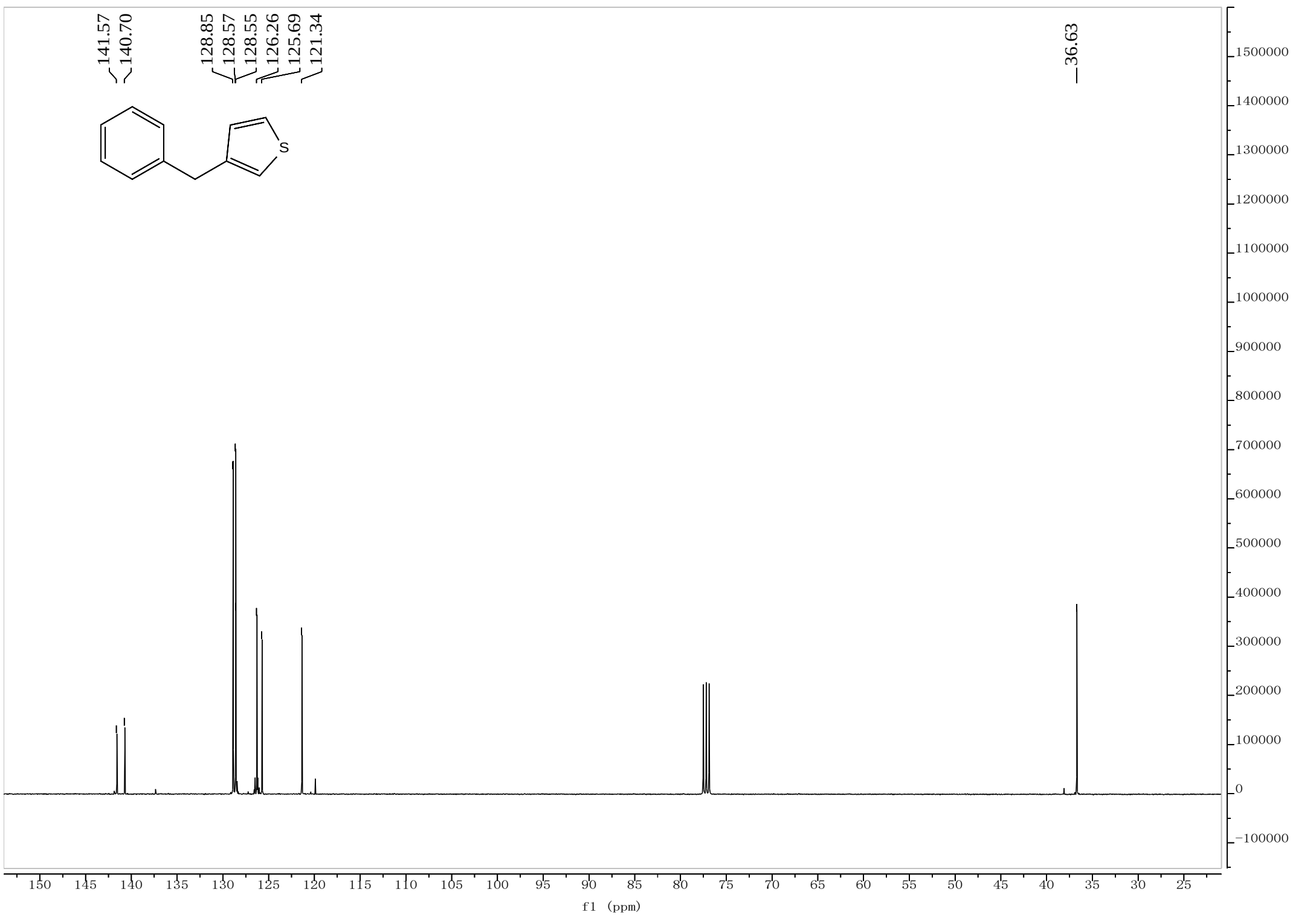

^{13}C NMR (101 MHz, Chloroform-*d*) *δ* 141.57, 140.70, 128.85, 128.57, 128.55, 126.26, 125.69, 121.34, 36.63.

BM-11

^{1}H-NMR

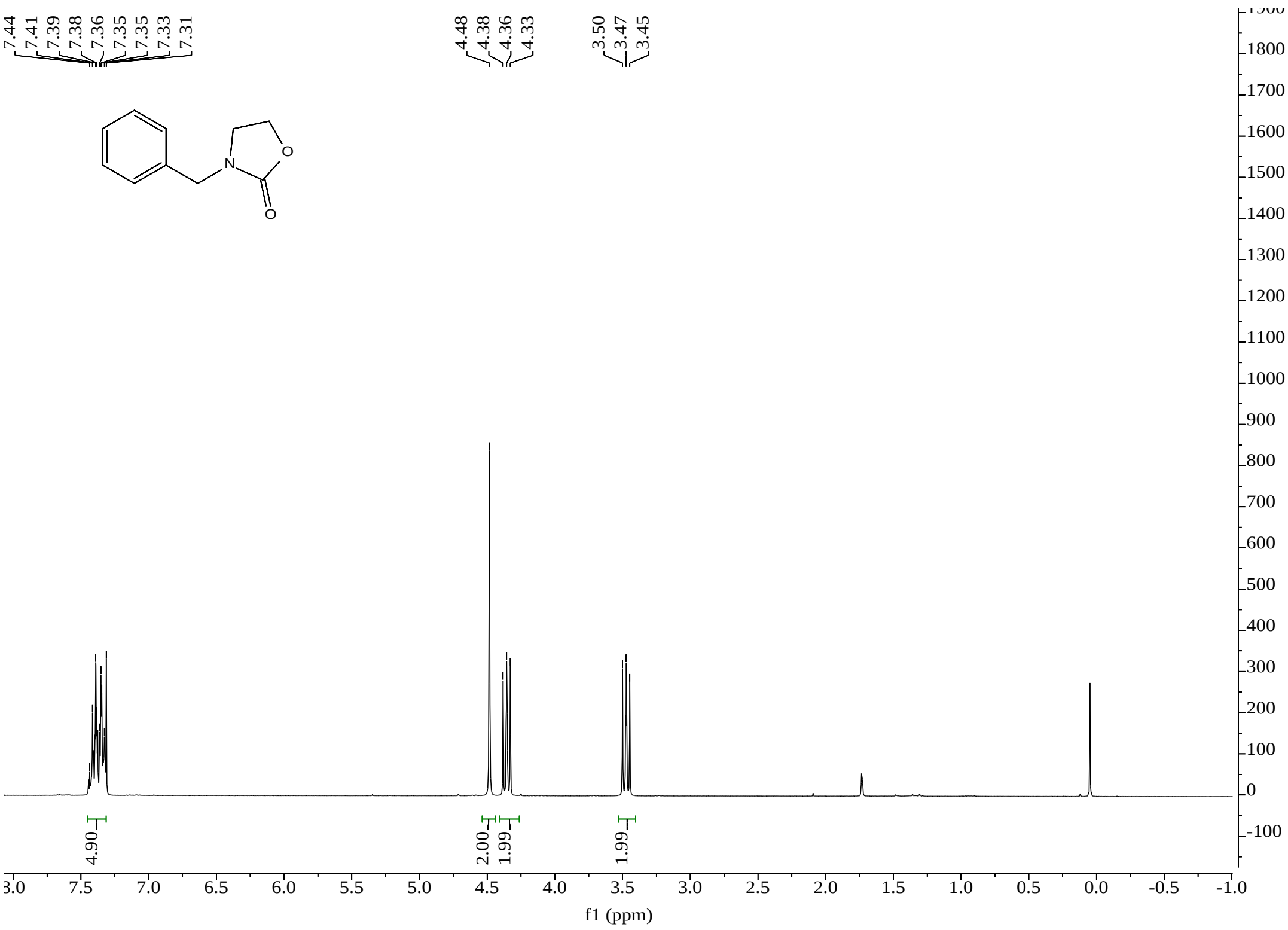

^{1}H NMR (300 MHz, Chloroform-*d*) δ 7.45-7.31 (m, 5H), 4.48 (s, 2H), 4.41-4.26 (m, 2H), 3.53-3.40 (m, 2H).

^{13}C-NMR

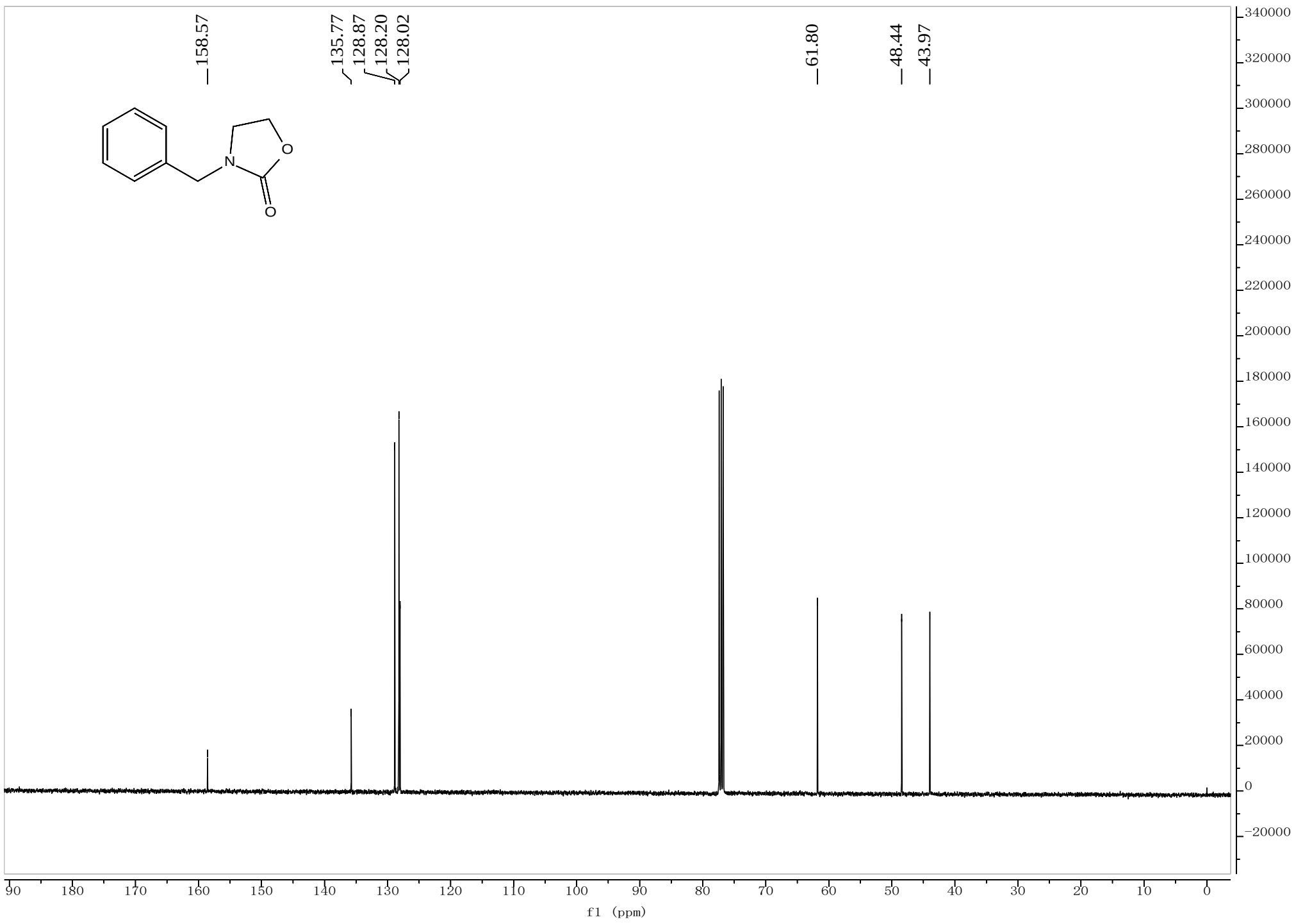

^{13}C NMR (101 MHz, Chloroform-*d*) δ 158.57, 135.77, 128.87, 128.20, 128.02, 61.80, 48.44, 43.97.

BM-12

^{1}H-NMR

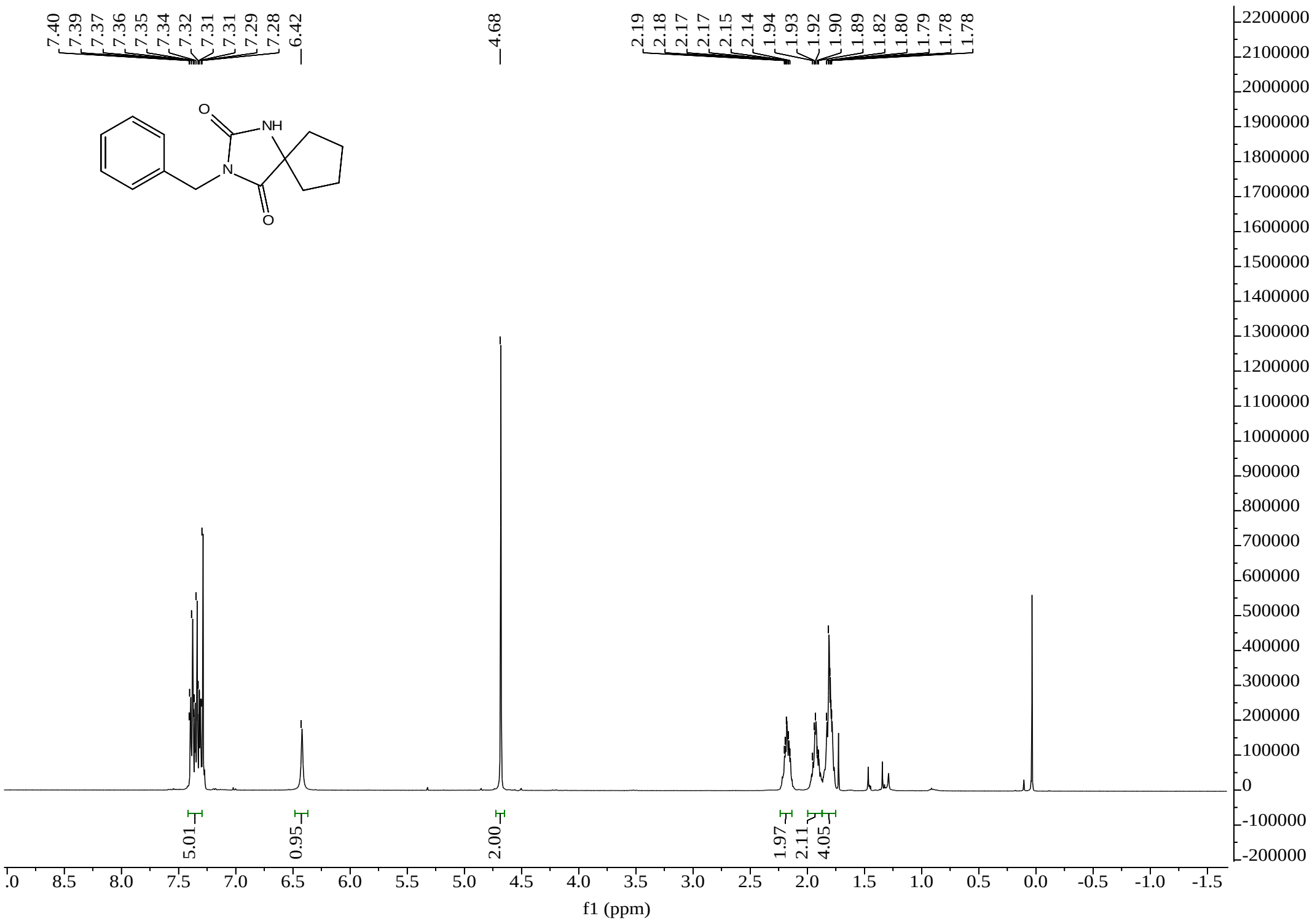

^{1}H NMR (400 MHz, Chloroform-*d*) *δ* 7.41-7.28 (m, 5H), 6.42 (s, 1H), 4.68 (s, 2H), 2.23-2.12 (m, 2H), 1.92 (dt, *J* = 9.9, 5.4 Hz, 2H), 1.86-1.74 (m, 4H).

^{13}C-NMR

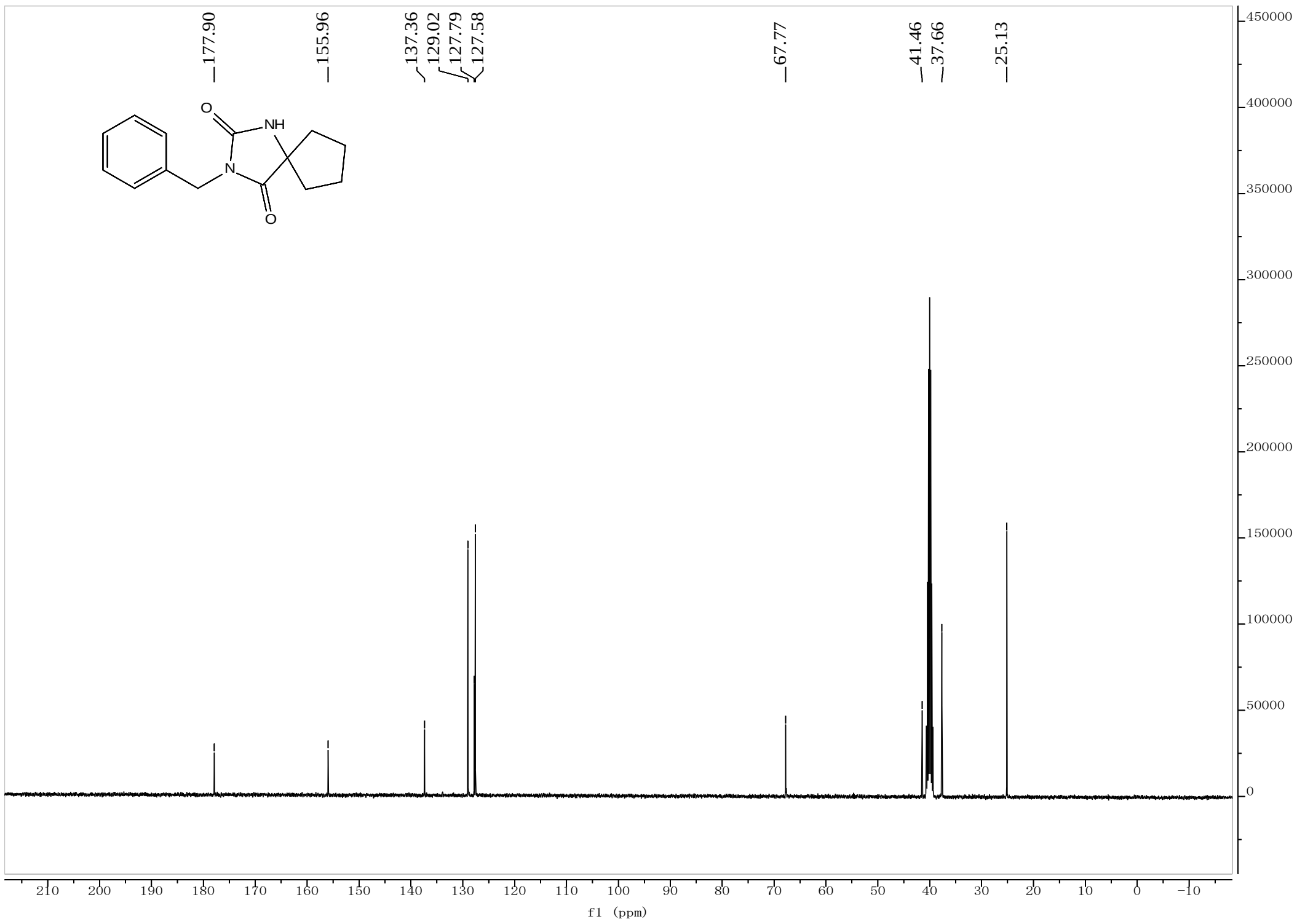

^{13}C NMR (101 MHz, DMSO-*d*$_6$) *δ* 177.90, 155.96, 137.36, 129.02, 127.79, 127.58, 67.77, 41.46, 37.66, 25.13.

BM-13

^{1}H-NMR

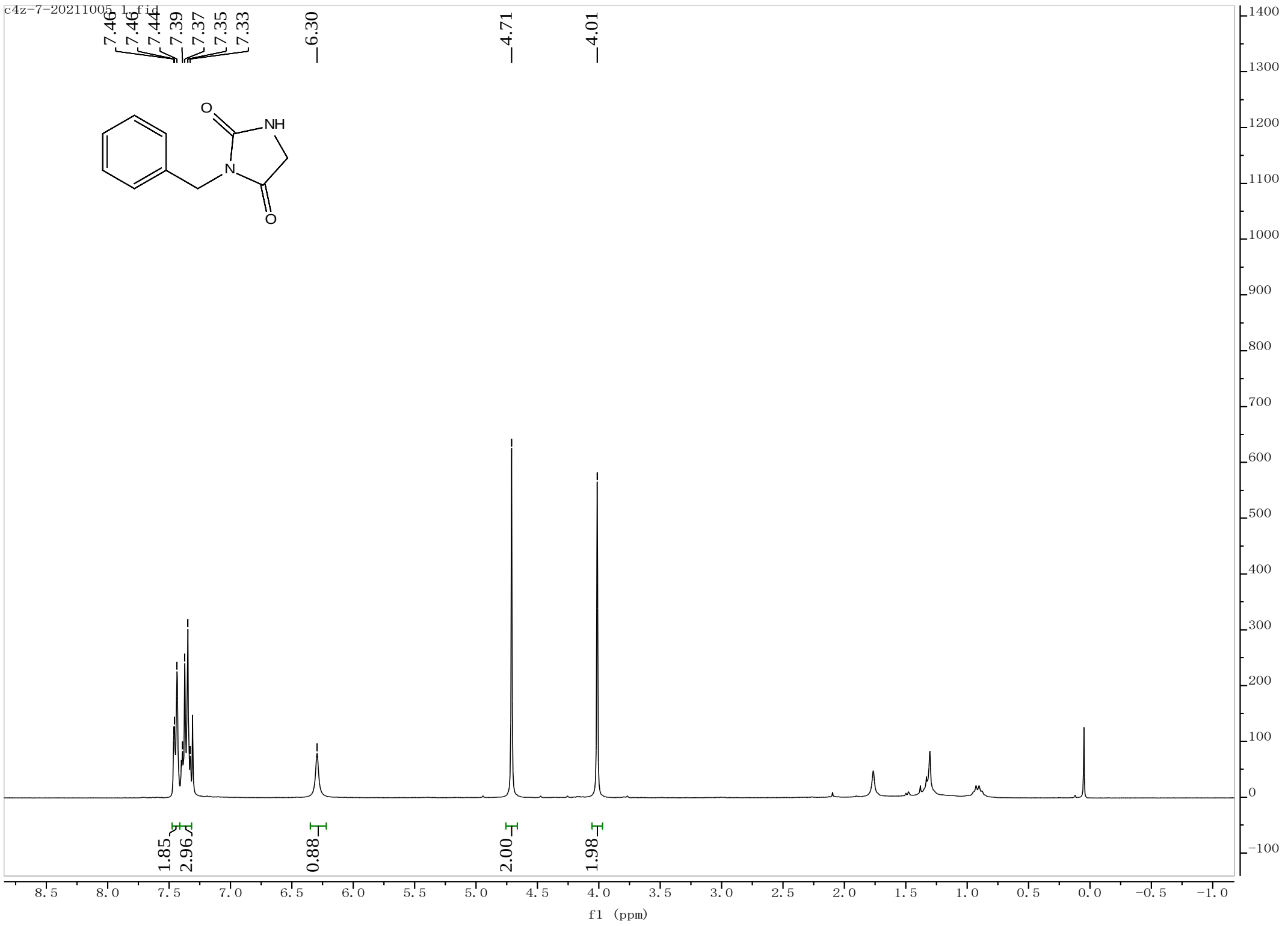

^{1}H NMR (300 MHz, Chloroform-*d*) δ 7.48-7.41 (m, 2H), 7.36 (q, J = 5.7 Hz, 3H), 6.30 (s, 1H), 4.71 (s, 2H), 4.01 (s, 2H).

^{13}C NMR

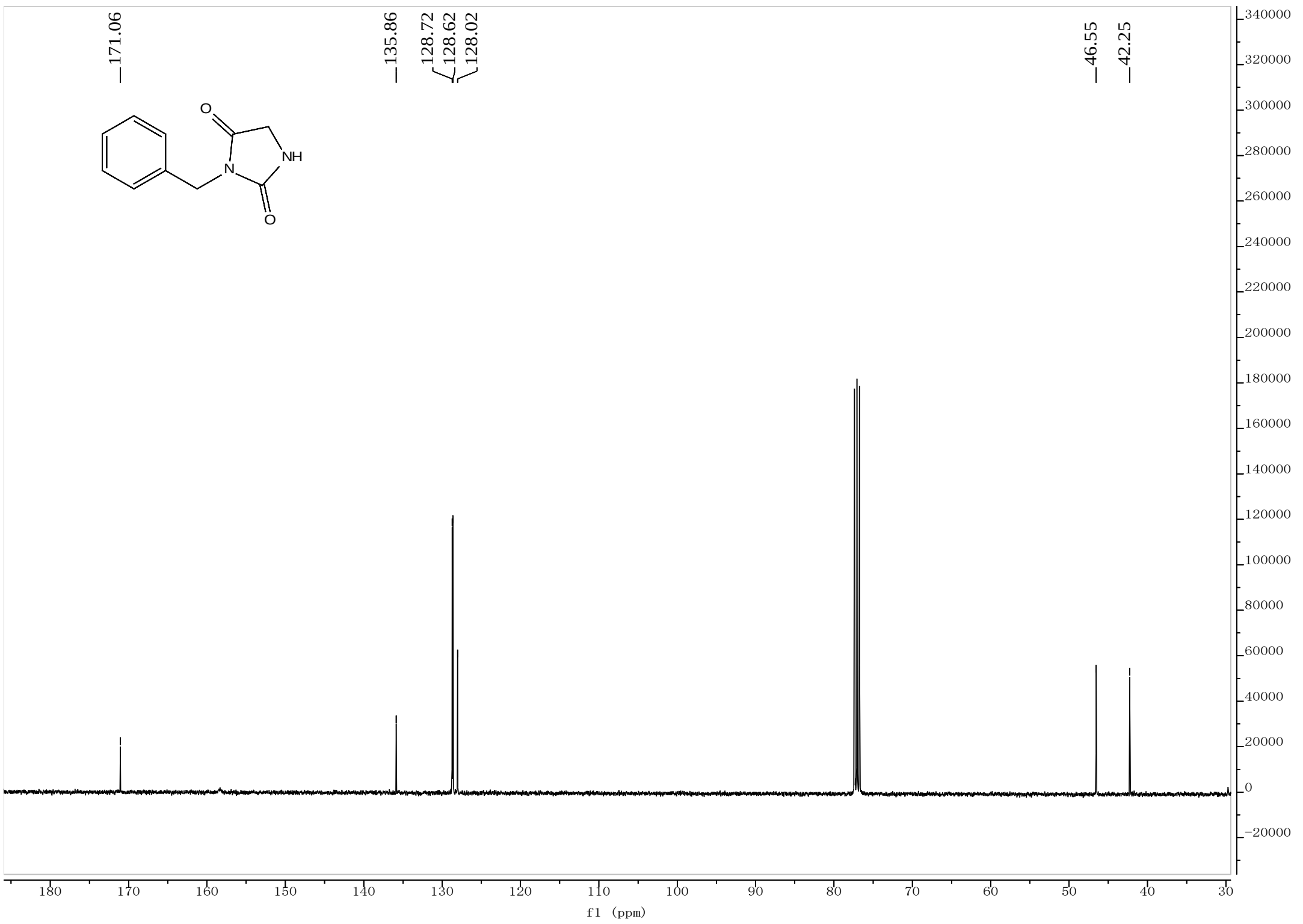

^{13}C NMR (101 MHz, Chloroform-*d*) δ 171.06, 135.86, 128.72, 128.62, 128.02, 46.55, 42.25.

BM-14

^{1}H NMR

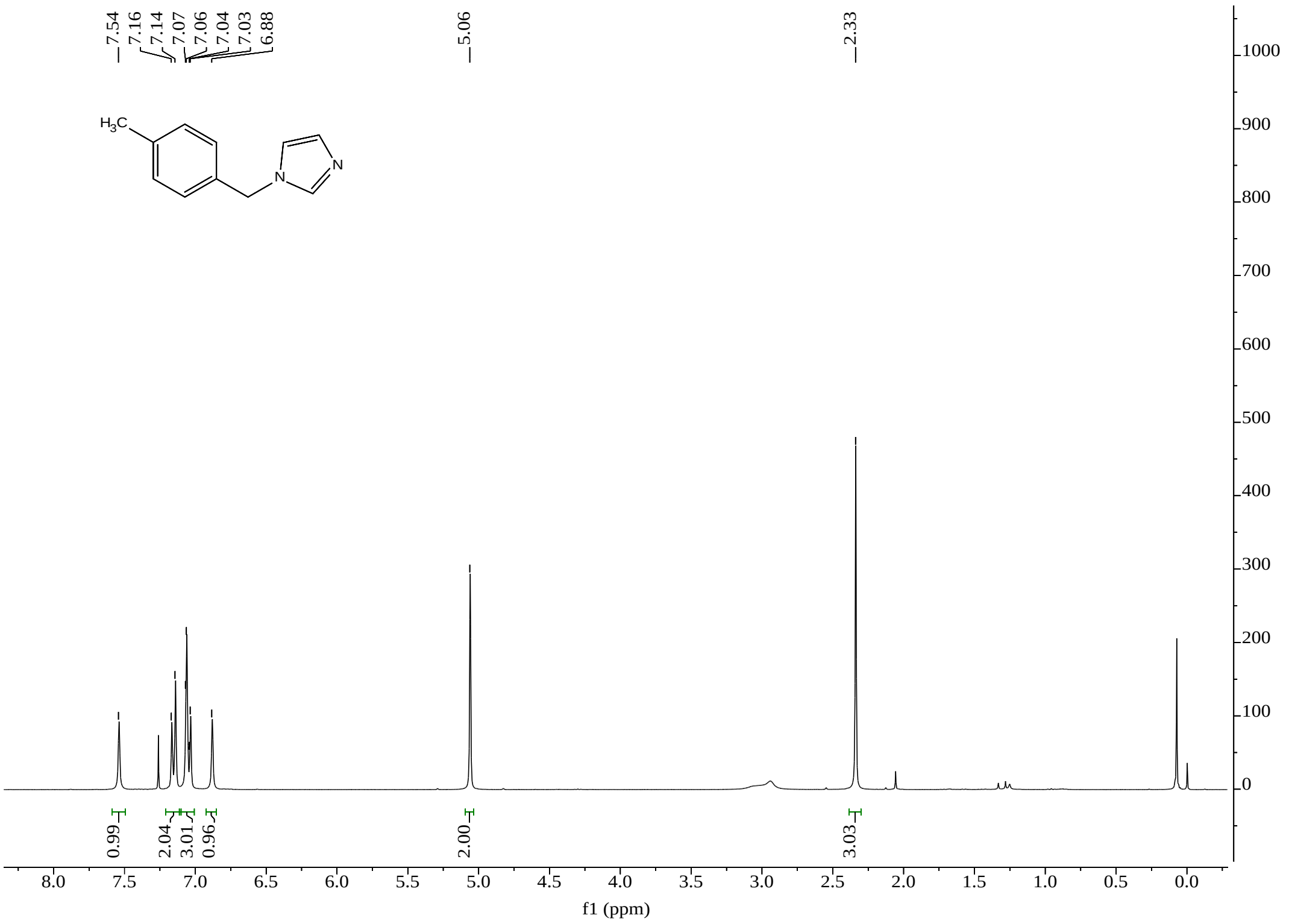

^{1}H NMR (300 MHz, Chloroform-*d*) *δ* 7.54 (s, 1H), 7.15 (d, *J* = 7.8 Hz, 2H), 7.11-7.00 (m, 3H), 6.88 (s, 1H), 5.06 (s, 2H), 2.33 (s, 3H).

^{13}C NMR

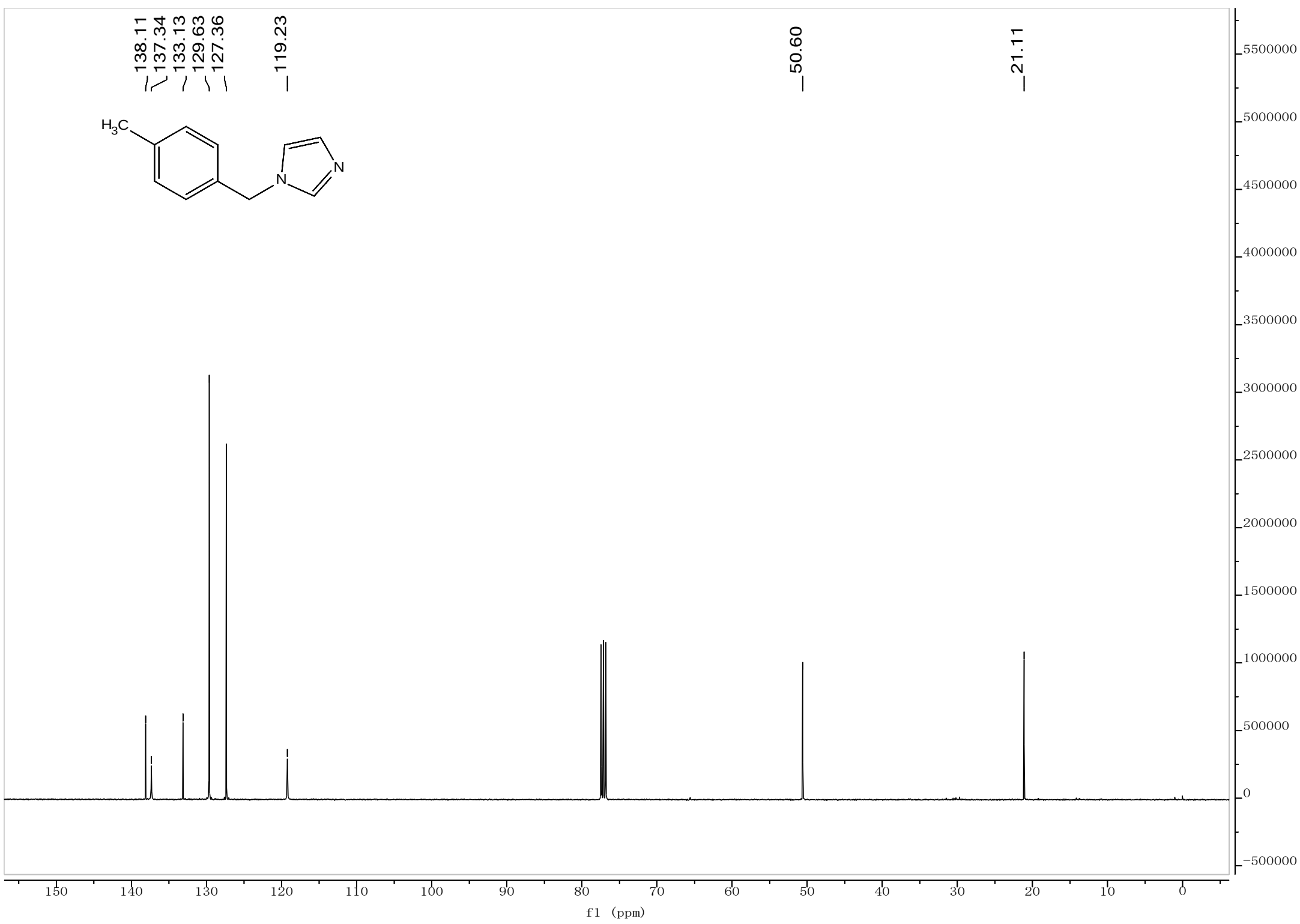

^{13}C NMR (101 MHz, Chloroform-*d*) *δ* 138.11, 133.13, 129.63, 127.36, 50.60, 21.11.

BM-15

^{1}H NMR

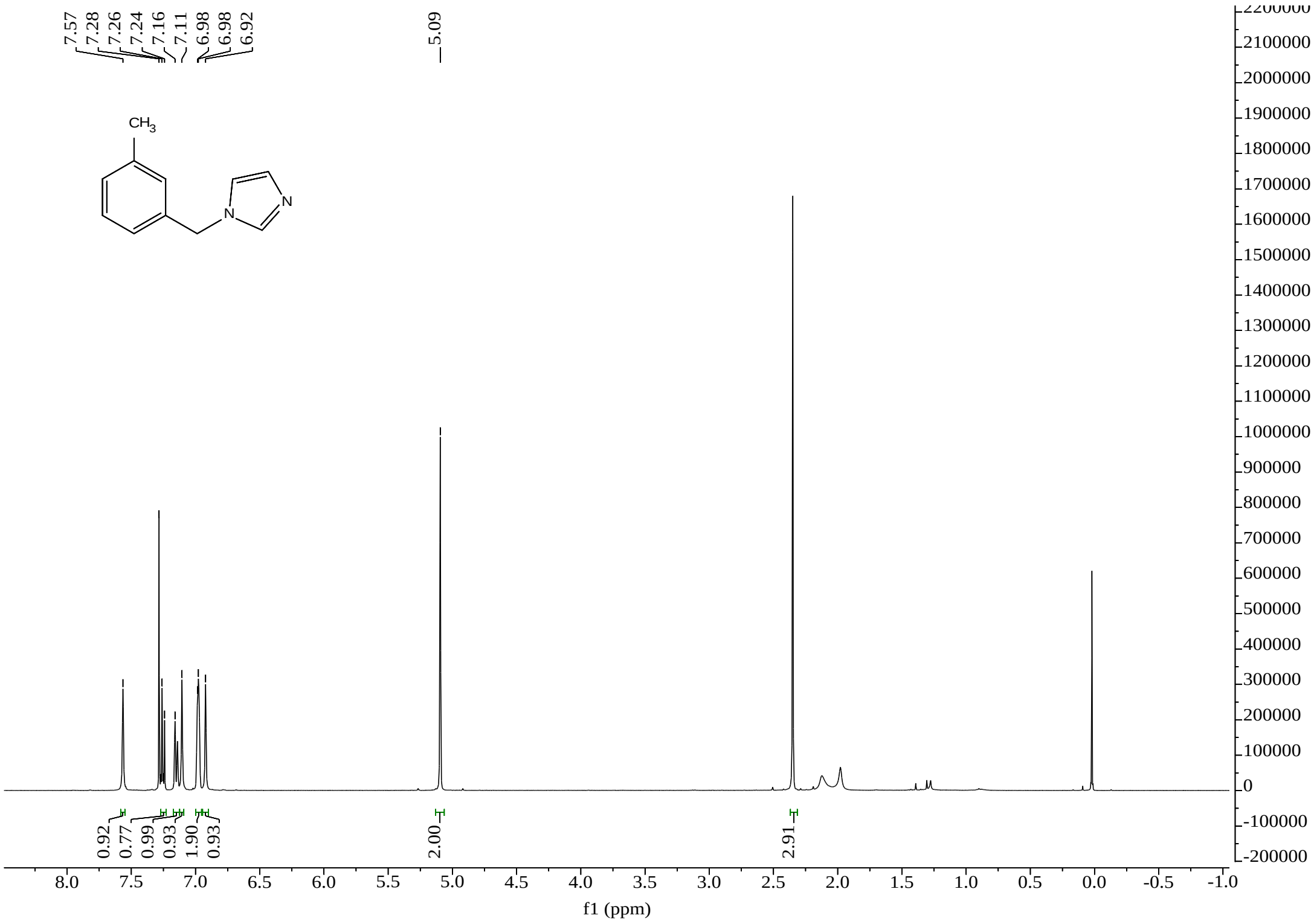

^{1}H NMR (400 MHz, Chloroform-*d*) δ 7.57 (s, 1H), 7.25 (d, J = 7.9 Hz, 1H), 7.16 (s, 1H), 7.11 (s, 1H), 7.00-6.95 (m, 2H), 6.92 (s, 1H), 5.09 (s, 2H), 2.35 (s, 3H).

^{13}C NMR

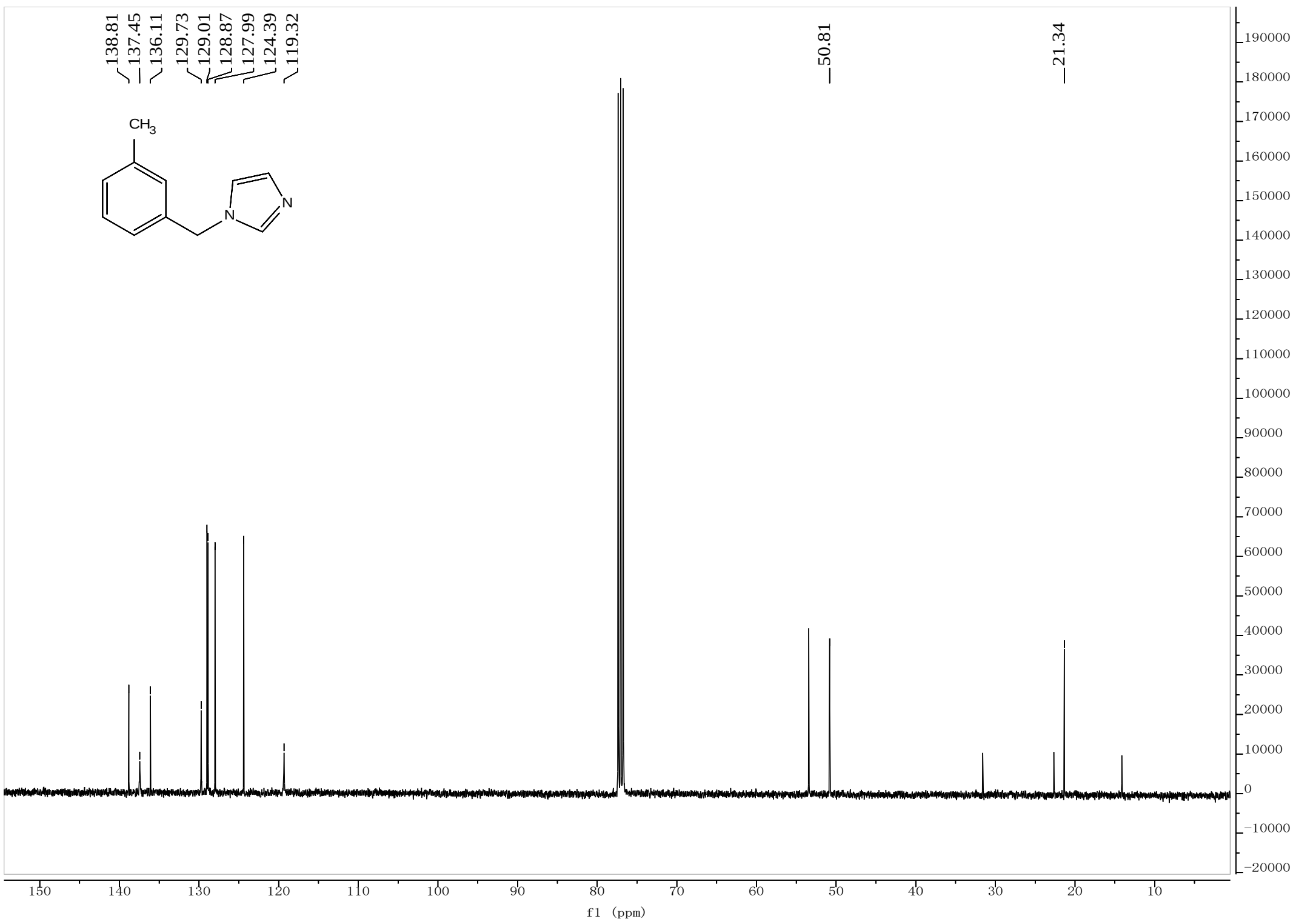

^{13}C NMR (101 MHz, Chloroform-*d*) δ 138.81, 136.11, 129.73, 129.01, 128.87, 127.99, 124.39, 50.81, 21.34.

BM-16

^{1}H NMR

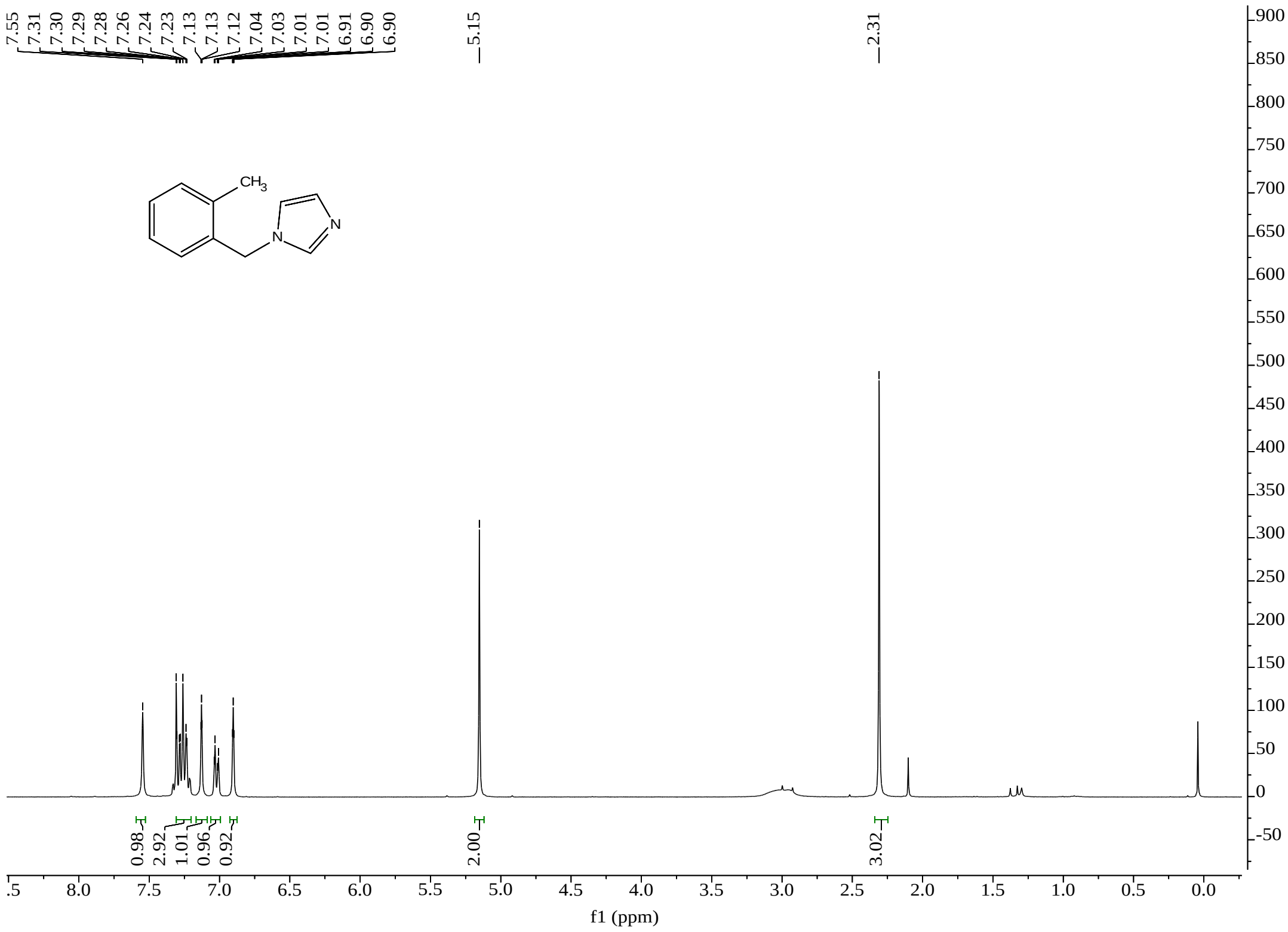

^{1}H NMR (300 MHz, Chloroform-*d*) δ 7.55 (s, 1H), 7.31-7.20 (m, 3H), 7.13 (d, J = 1.1 Hz, 1H), 7.02 (dd, J = 7.3, 2.0 Hz, 1H), 6.90 (d, J= 1.3 Hz, 1H), 5.15 (s, 2H), 2.31 (s, 3H).

^{13}C NMR

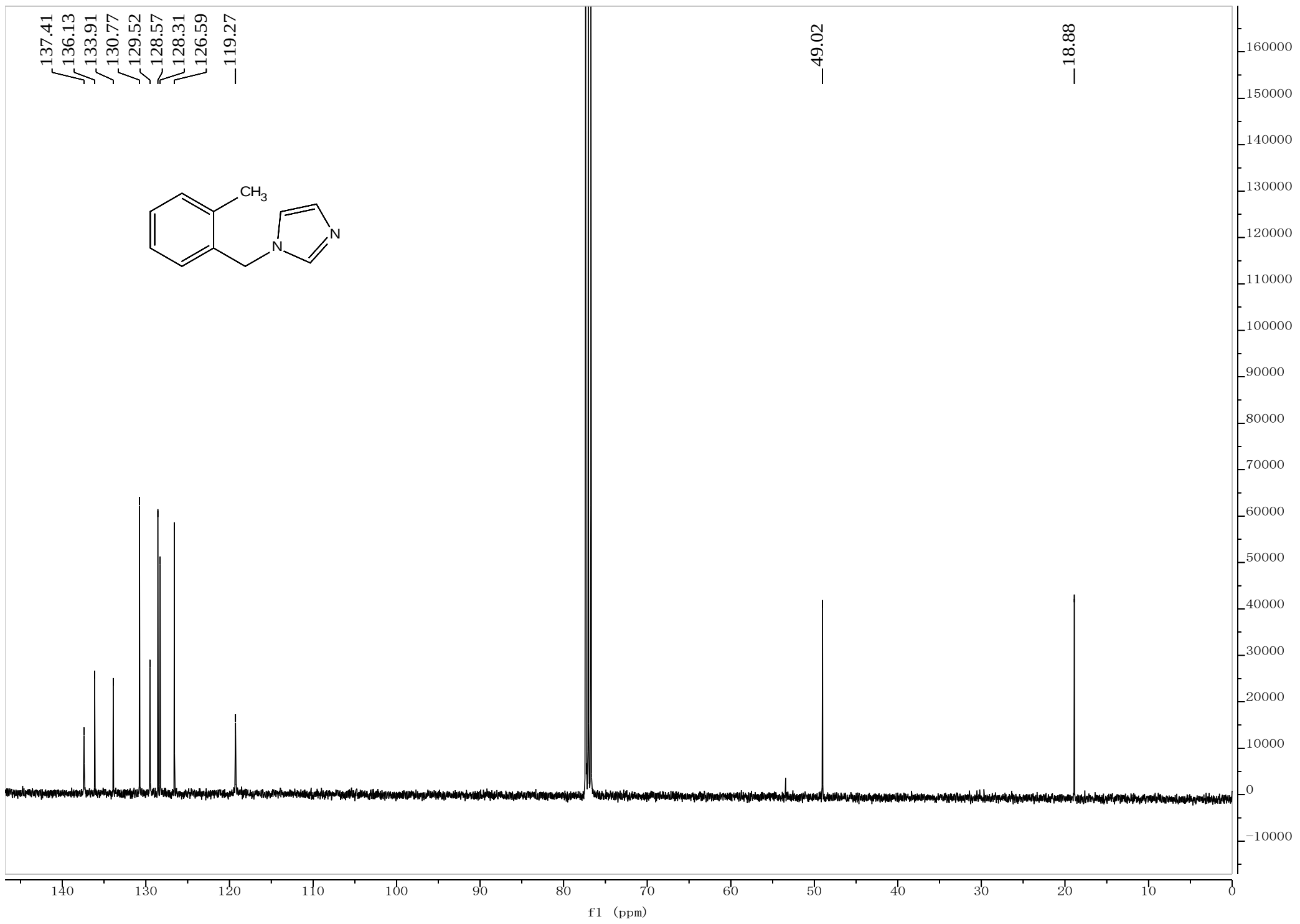

^{13}C NMR (101 MHz, Chloroform-*d*) δ 137.41, 136.13, 133.91, 130.77, 129.52,

128.57, 128.31, 126.59, 119.27, 49.02, 18.88.

BM-17

^{1}H NMR

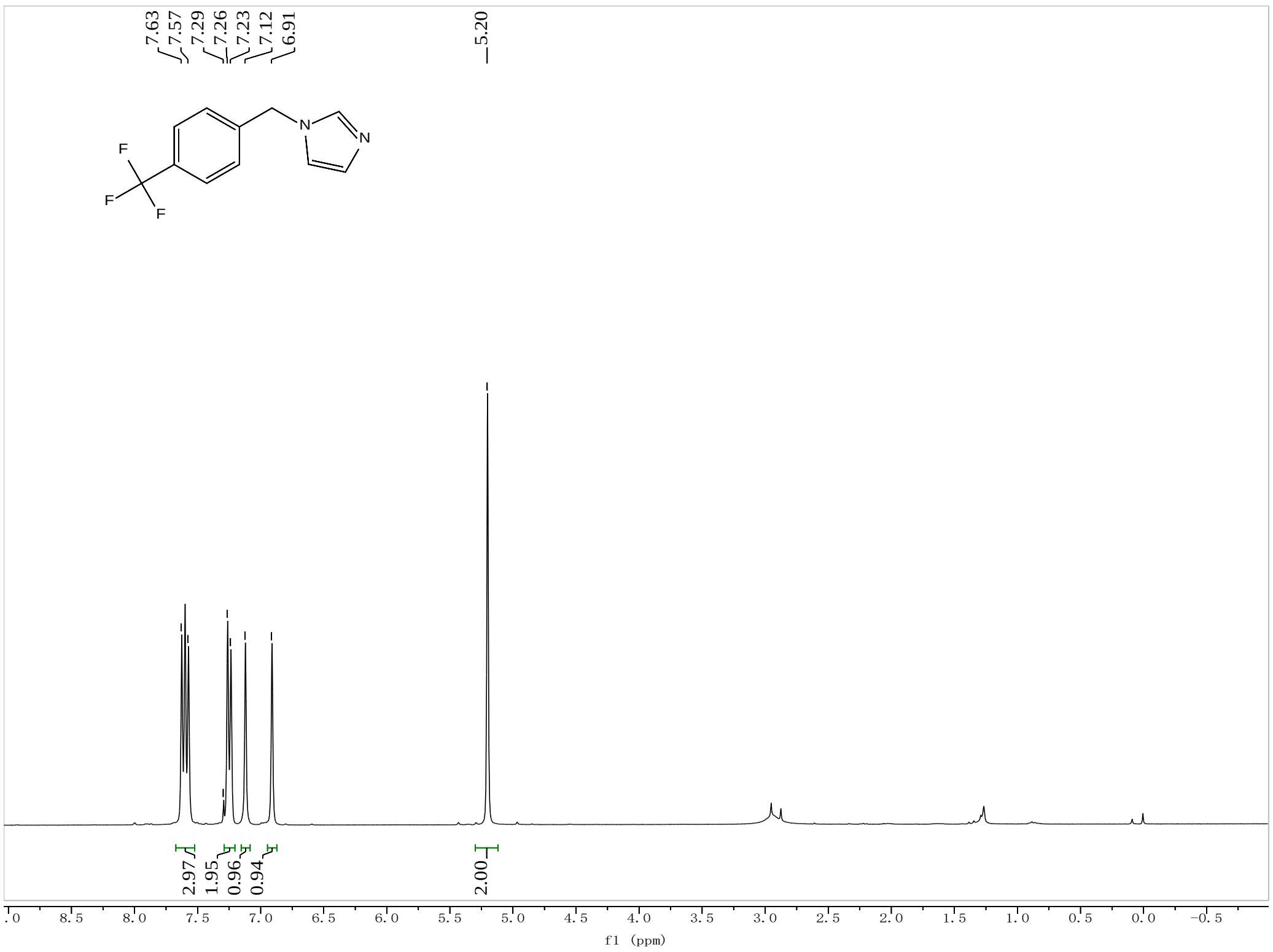

^{1}H NMR (300 MHz, Chloroform-*d*) δ 7.60 (d, J = 16.1 Hz, 3H), 7.25 (d, J = 8.0 Hz, 2H), 7.12 (s, 1H), 6.91 (s, 1H), 5.20 (s, 2H).

^{13}C NMR

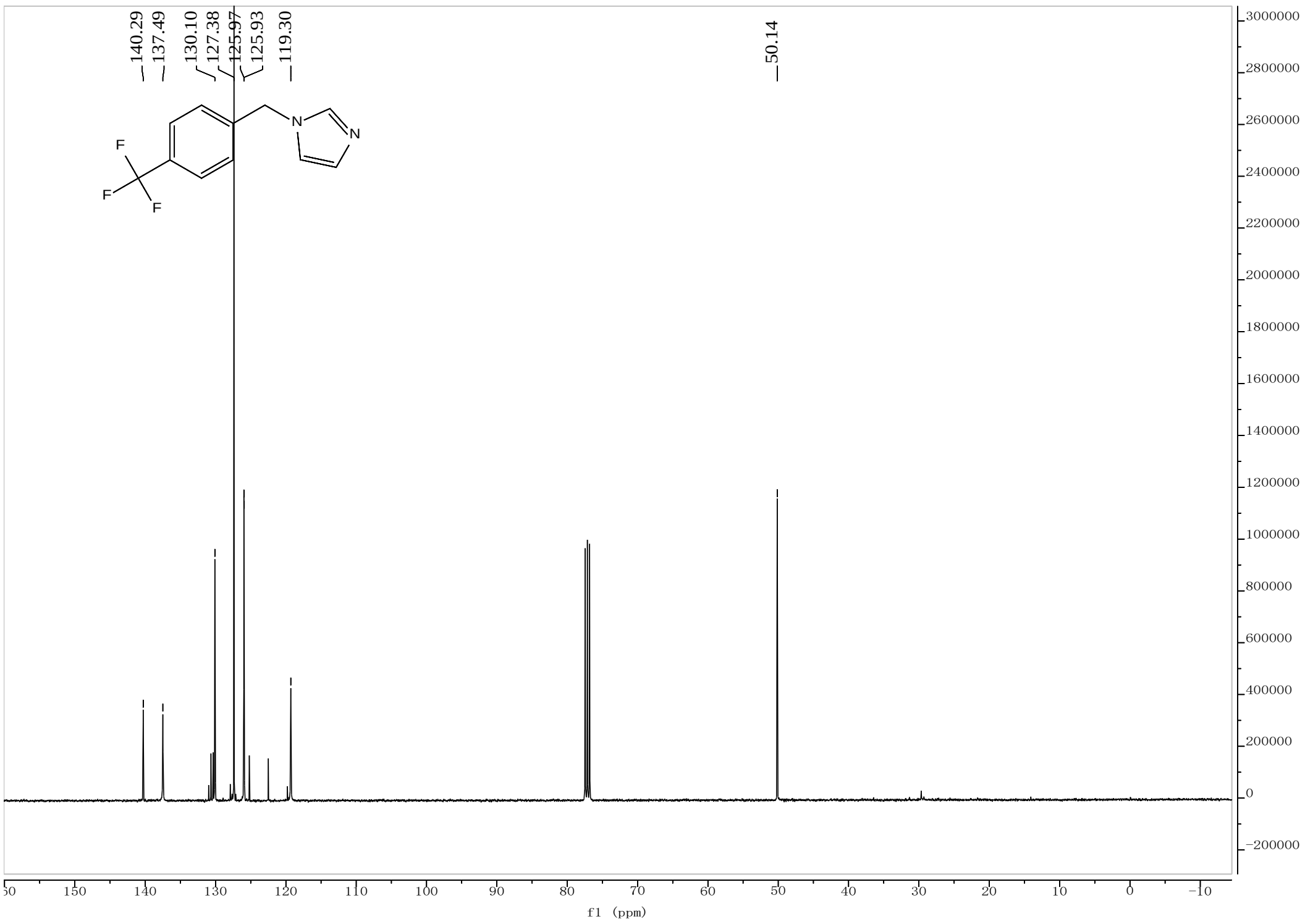

^{13}C NMR (101 MHz, Chloroform-*d*) δ 140.29, 137.49, 130.10, 127.38, 125.97, 119.30.

BM-18

^{1}H NMR

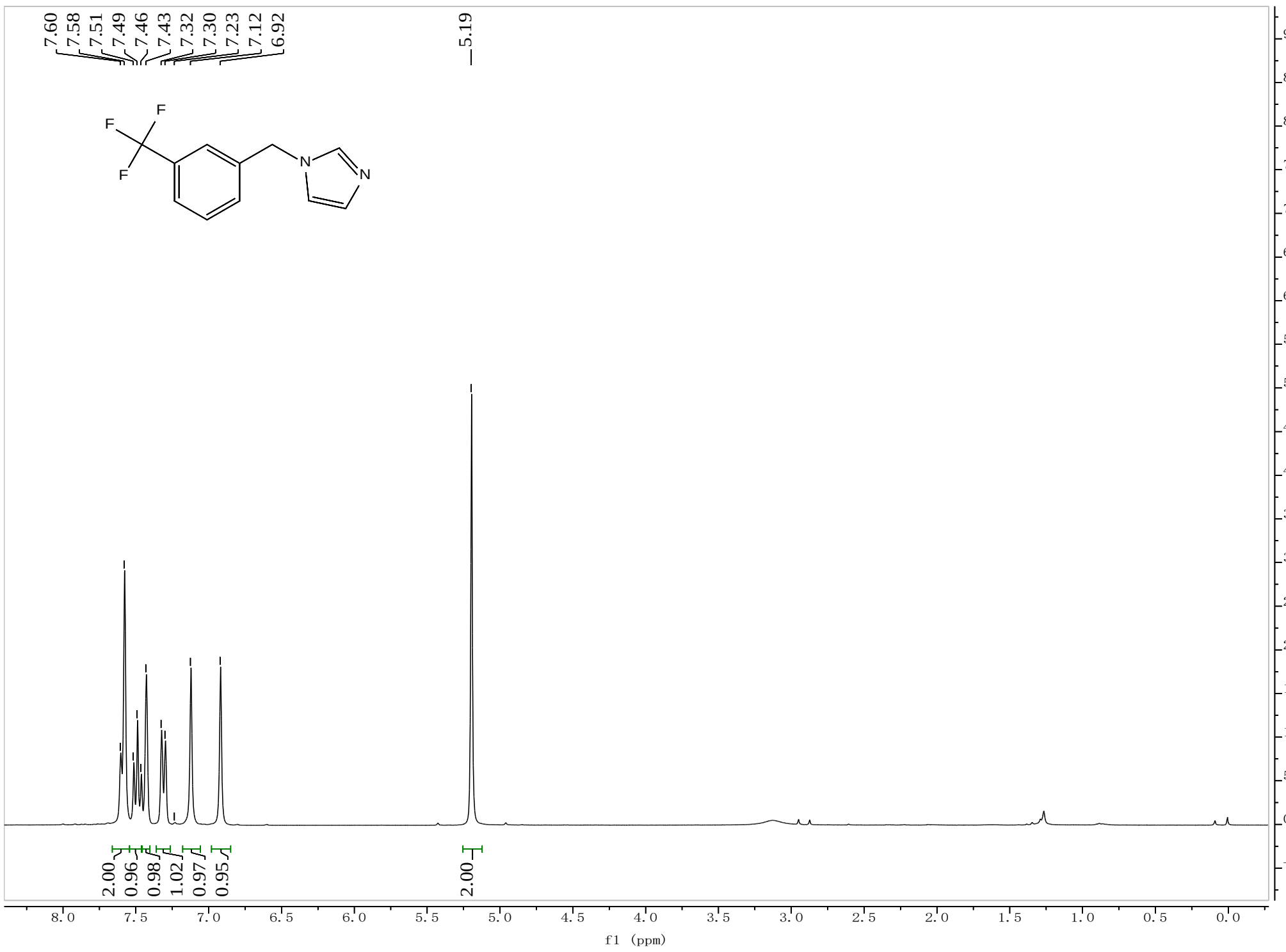

^{1}H NMR (300 MHz, Chloroform-*d*) δ 7.59 (d, J = 8.0 Hz, 2H), 7.49 (t, *J* = 7.7 Hz, 1H), 7.43 (s, 1H), 7.31 (d, *J* = 8.0 Hz, 1H), 7.12 (s, 1H), 6.92 (s, 1H), 5.19 (s, 2H).

^{13}C NMR

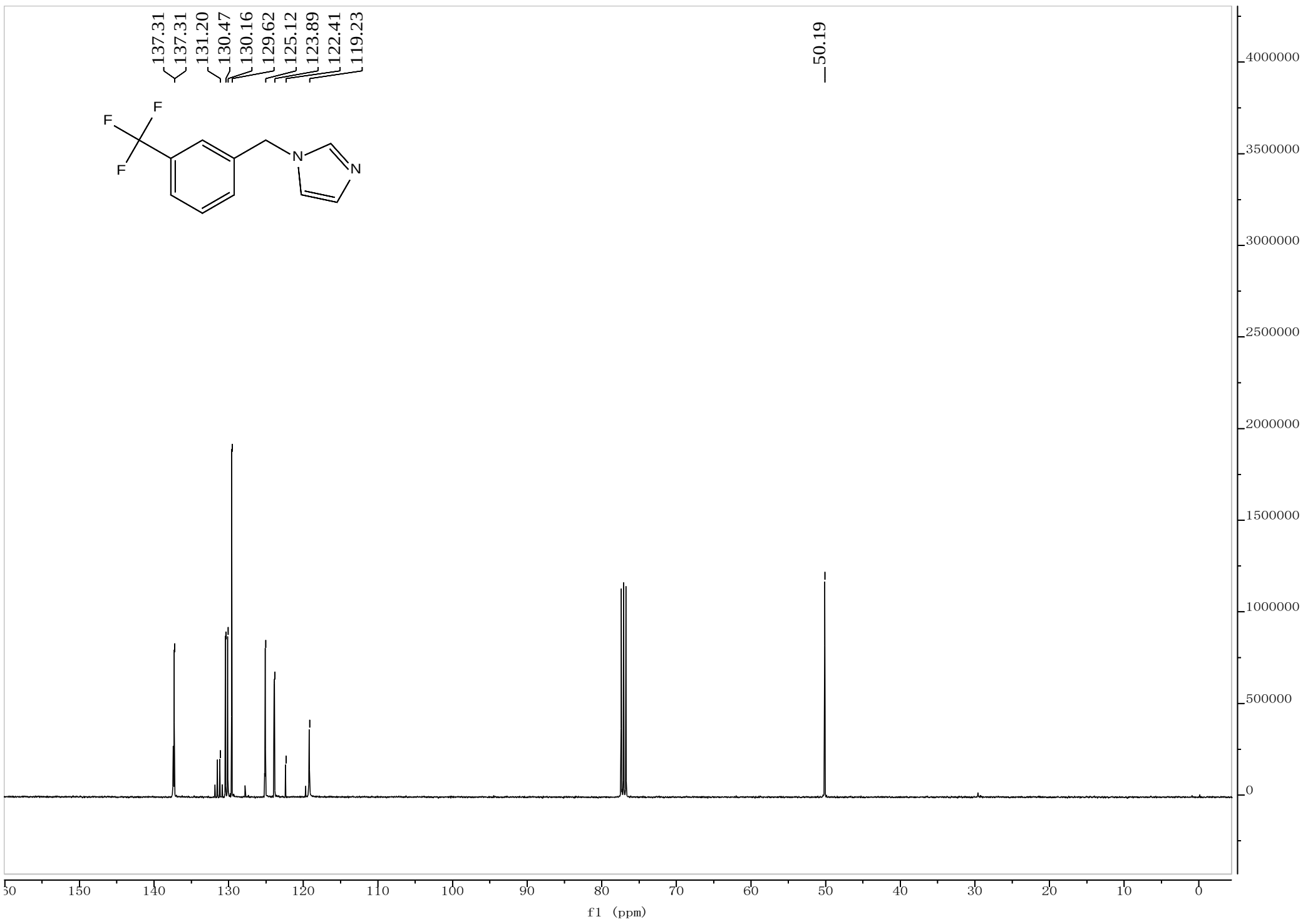

^{13}C NMR (101 MHz, Chloroform-*d*) δ 137.44, 137.31, 137.31, 131.20, 130.47, 130.16, 129.62, 125.12, 123.89, 119.23, 50.19.

BM-19

^{1}H NMR

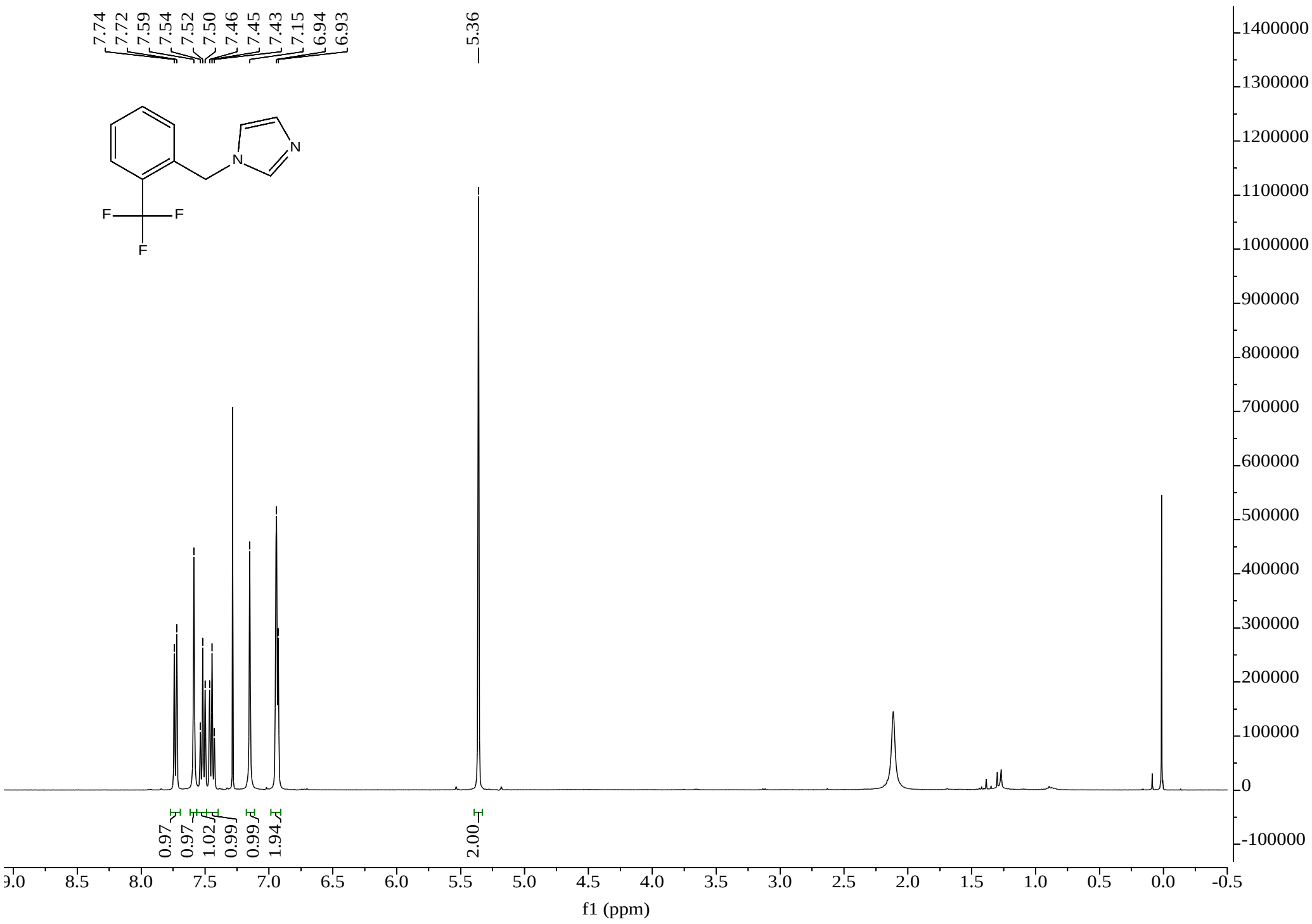

^{1}H NMR (400 MHz, Chloroform-*d*) δ 7.73 (d, J = 7.7 Hz, 1H), 7.59 (s, 1H), 7.52 (t, J = 7.4 Hz, 1H), 7.45 (t, J = 7.5 Hz, 1H), 7.15 (s, 1H), 6.93 (d, J = 5.5 Hz, 2H), 5.36 (s, 2H).

^{13}C NMR

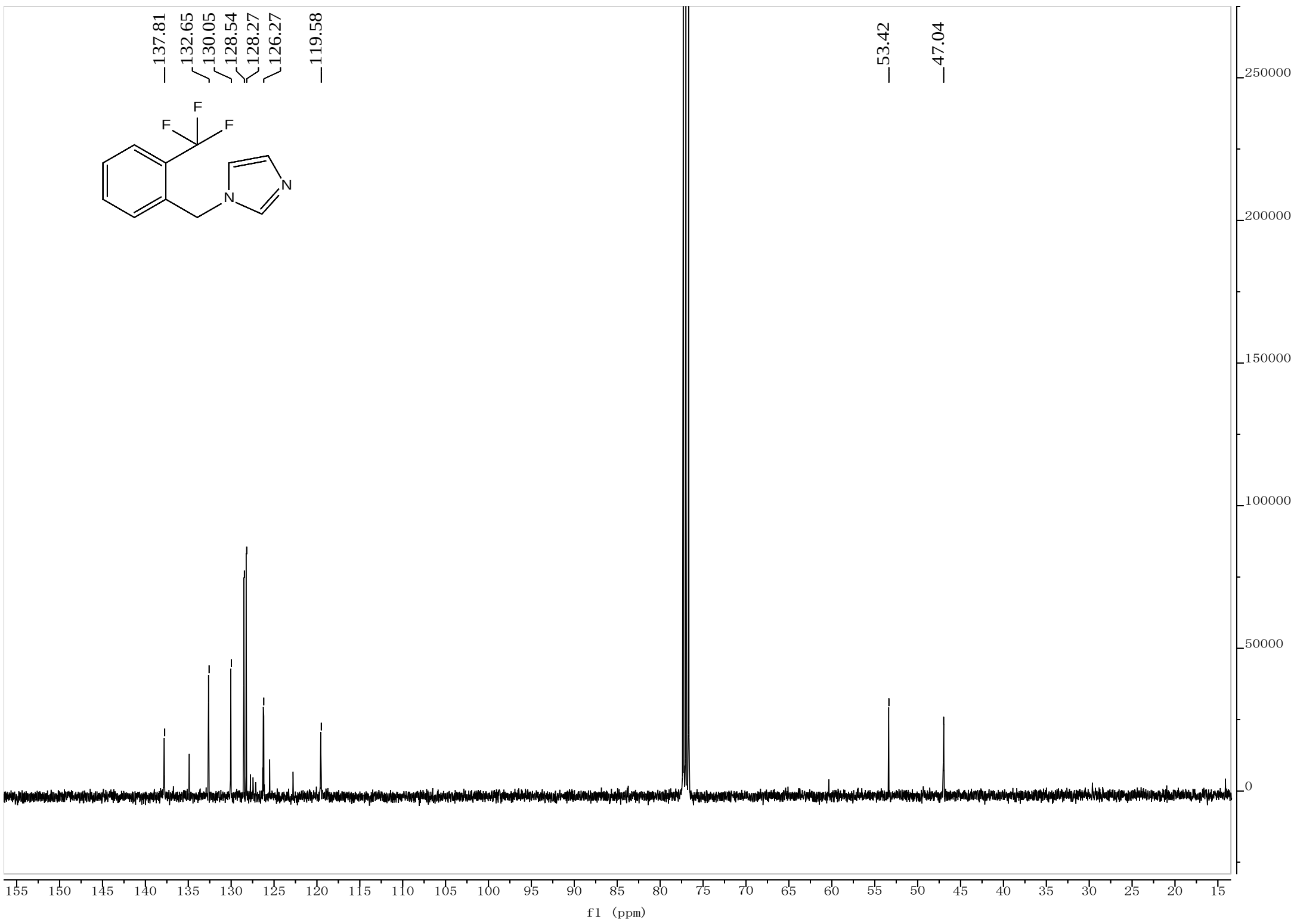

^{13}C NMR (101 MHz, Chloroform-*d*) δ 137.81, 132.65, 130.05, 128.54, 128.27, 126.27, 119.58, 53.42, 47.04.

BM-20

^{1}H NMR

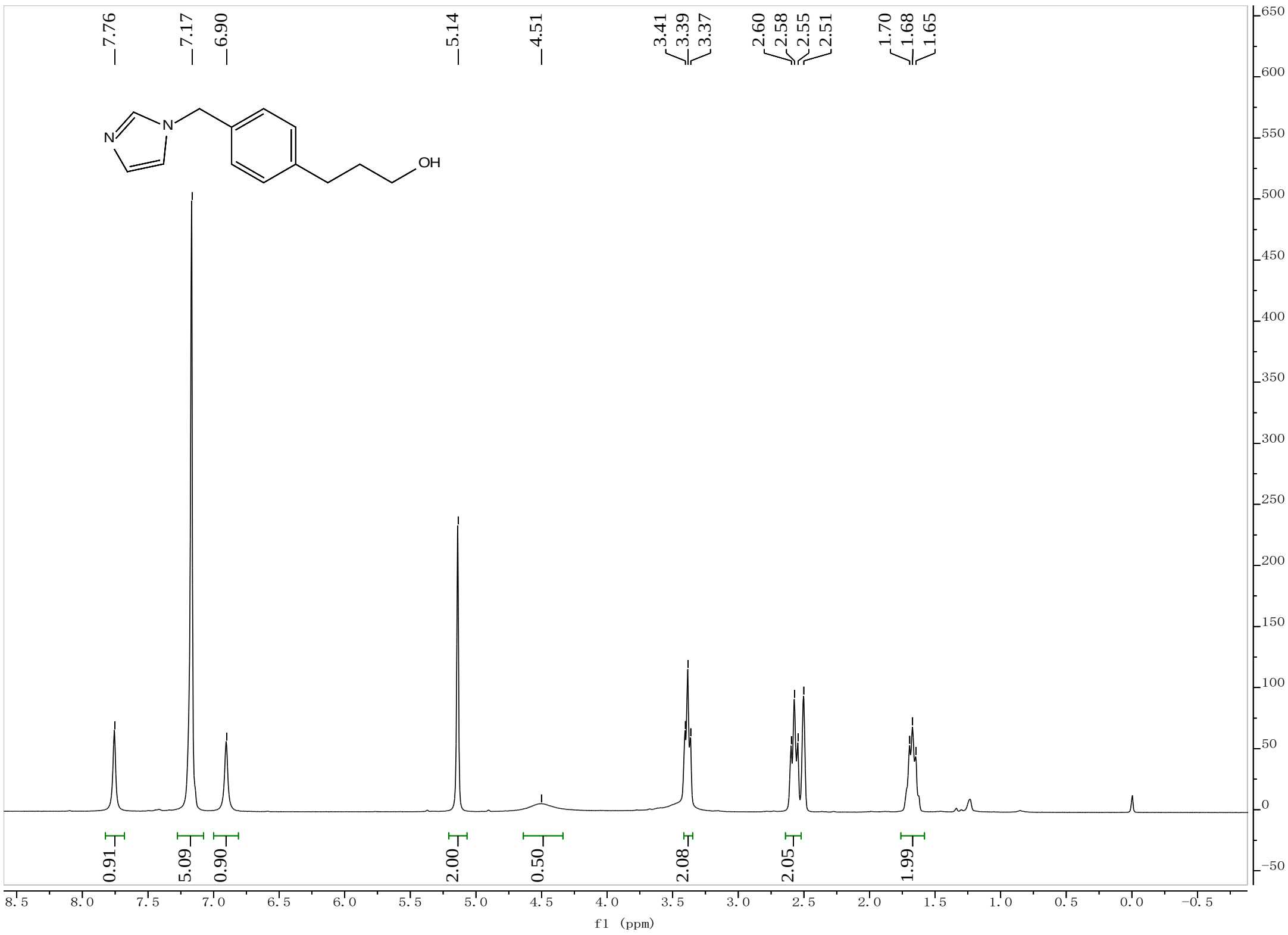

^{1}H NMR (300 MHz, DMSO-d_6) δ 7.76 (s, 1H), 7.17 (s, 5H), 6.90 (s, 1H), 5.14 (s, 2H), 3.39 (t, J = 6.1 Hz, 2H), 2.58 (t, J = 7.6 Hz, 2H), 1.77-1.59 (m, 2H).

^{13}C NMR

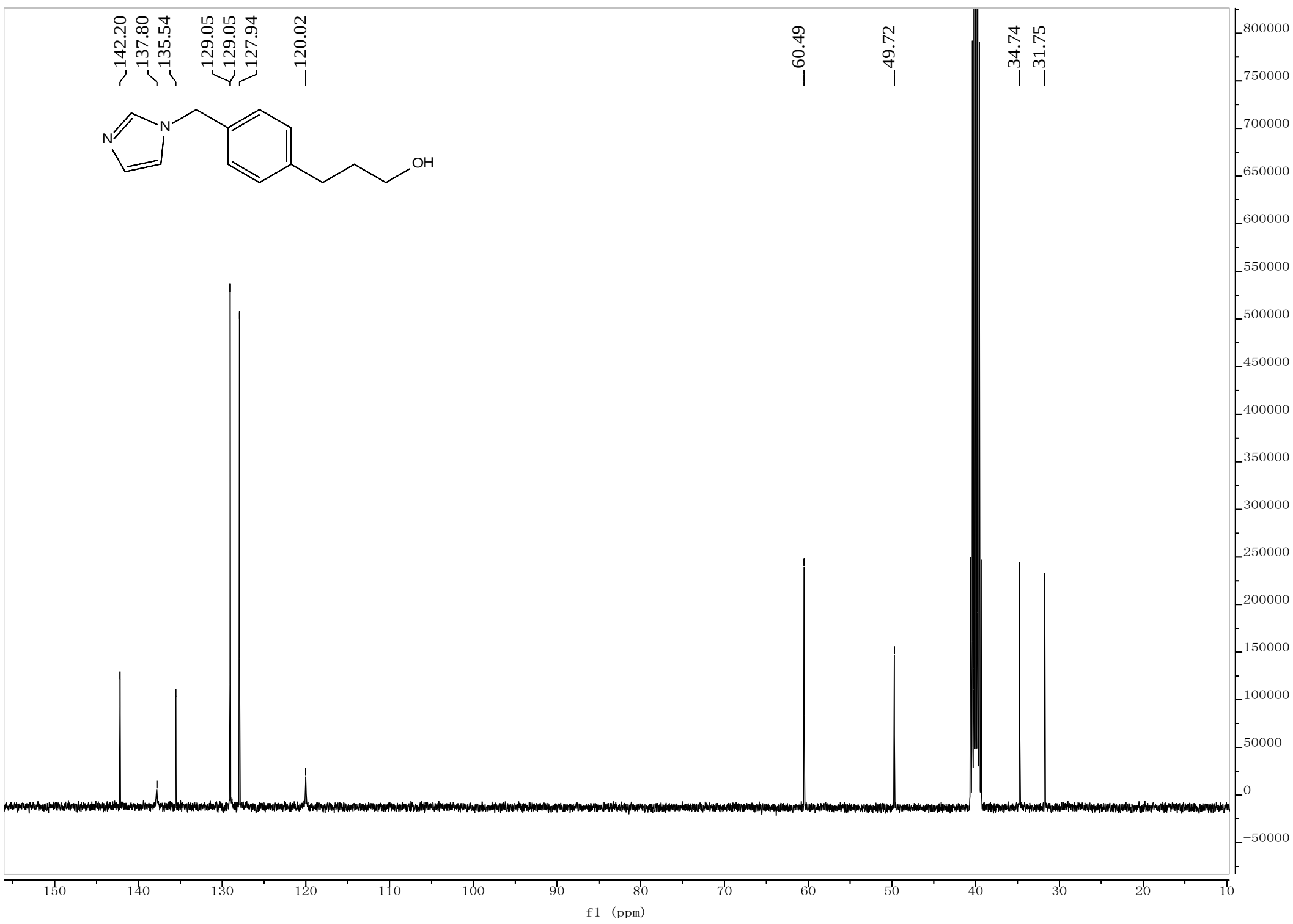

^{13}C NMR (101 MHz, DMSO-d_6) δ 142.20, 135.54, 129.05, 129.05, 127.94, 60.49, 49.72, 34.74, 31.75.

BM-21

^{1}H NMR

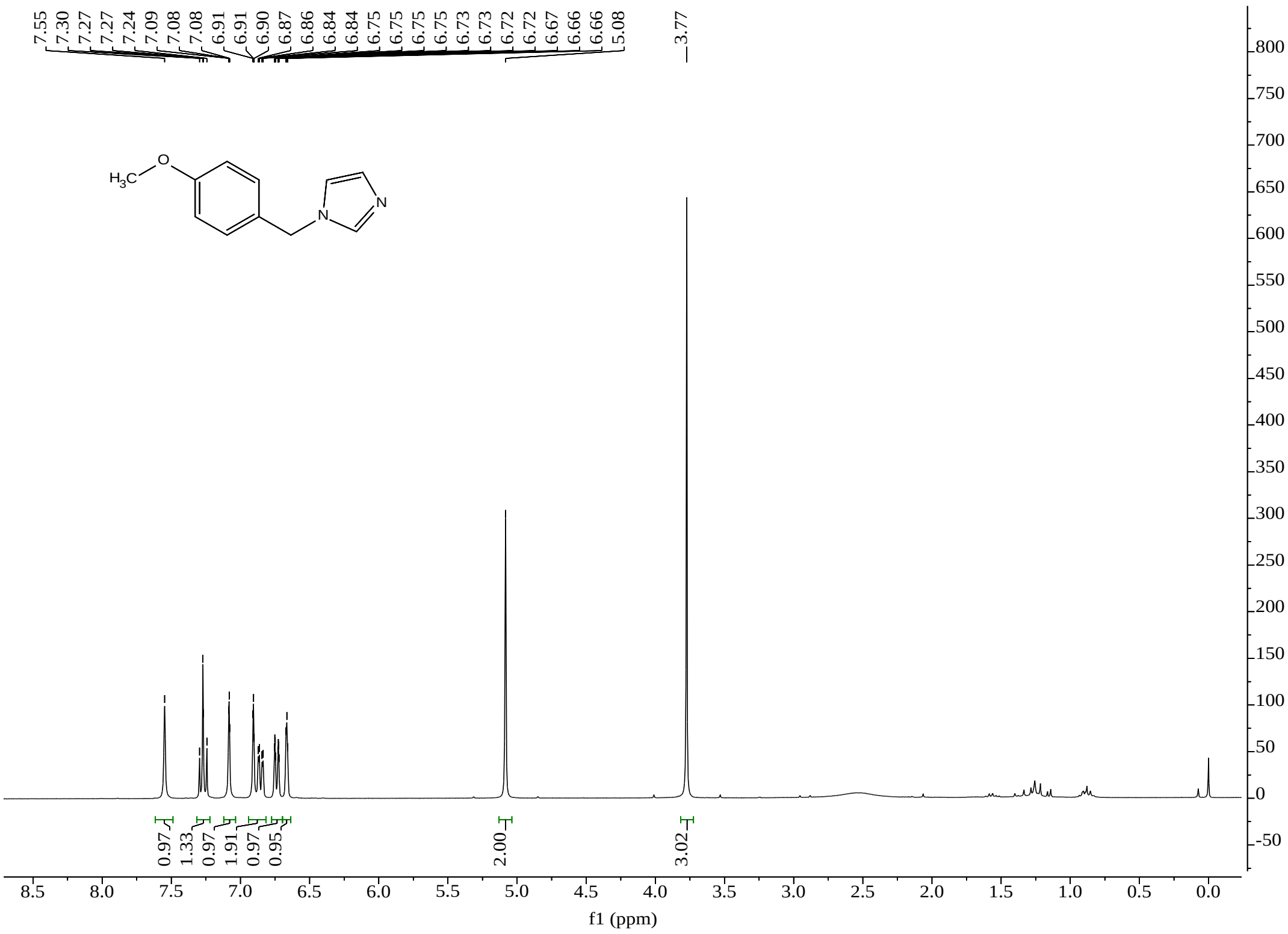

^{1}H NMR (300 MHz, Chloroform-*d*) *δ* 7.55 (s, 1H), 7.31-7.22 (m, 1H), 7.12-7.04 (m, 1H), 6.94-6.81 (m, 2H), 6.77-6.70 (m, 1H), 6.66 (t, *J* = 2.1 Hz, 1H), 5.08 (s, 2H), 3.77 (s, 3H).

^{13}C NMR

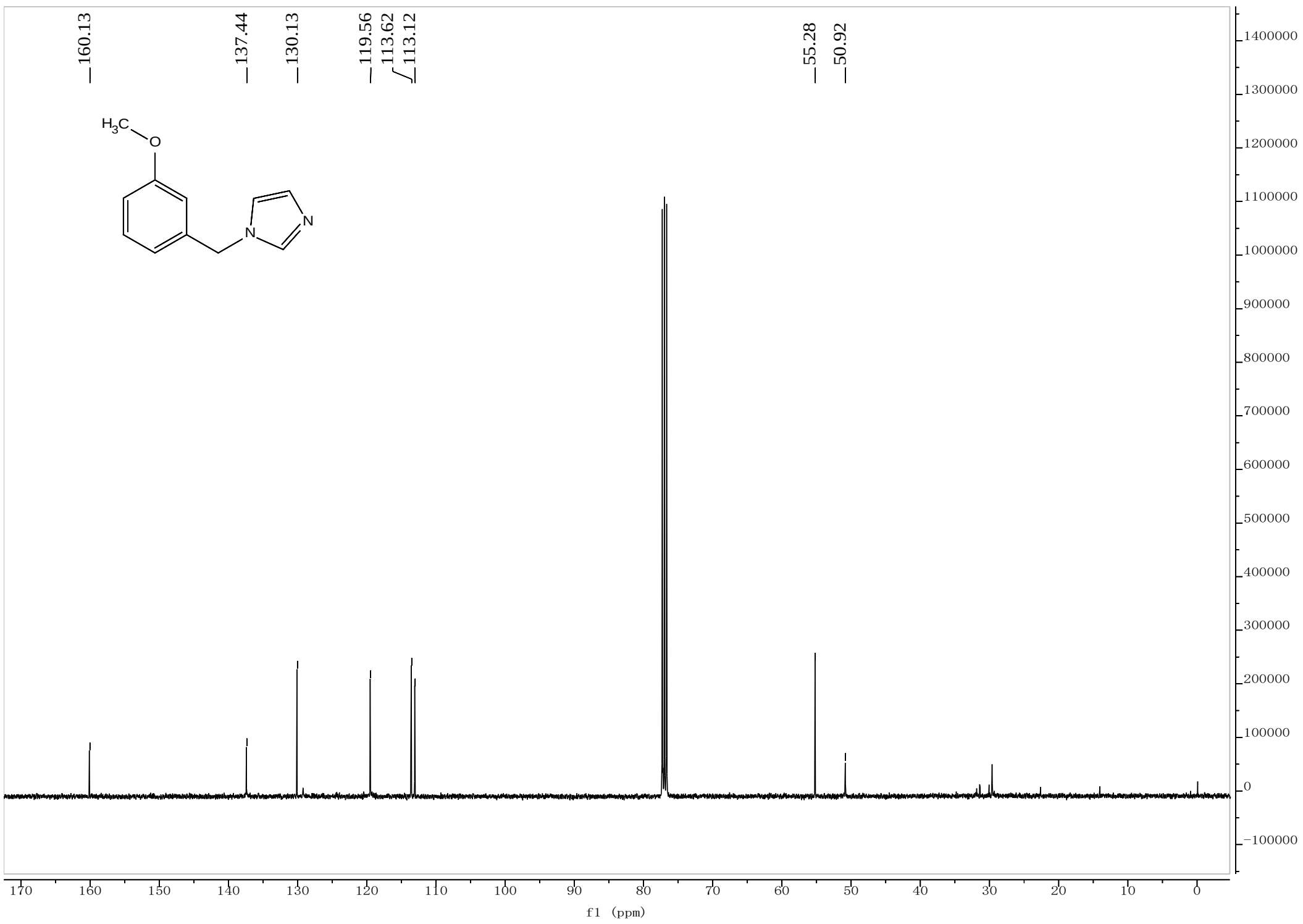

^{13}C NMR (101 MHz, Chloroform-*d*) *δ* 160.13, 137.44, 130.13, 119.56, 113.62, 113.12, 55.28.

BM-22

^{1}H NMR

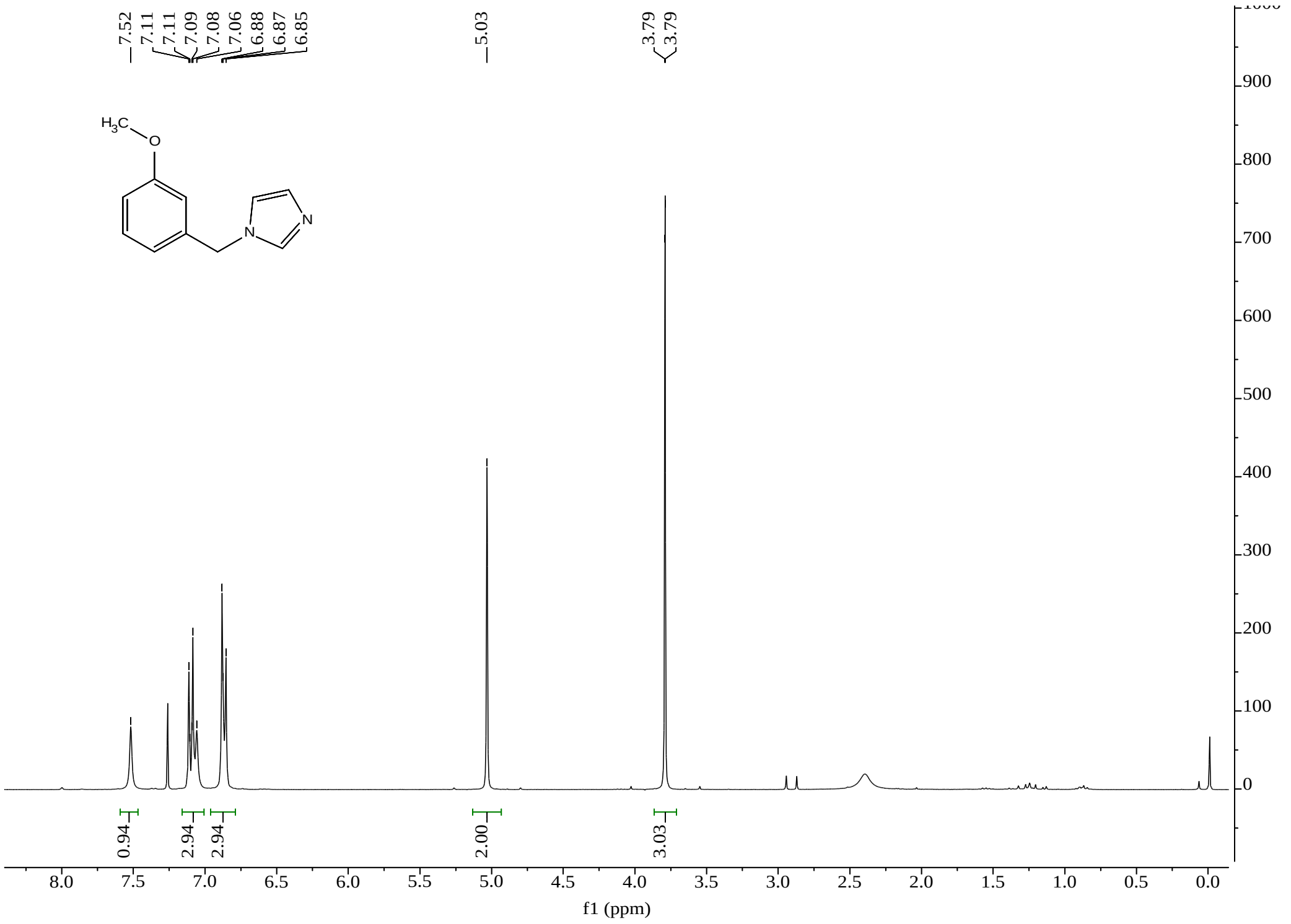

^{1}H NMR (300 MHz, Chloroform-*d*) δ 7.52 (s, 1H), 7.09 (dd, *J* = 9.4, 7.2 Hz, 3H), 6.96-6.79 (m, 3H), 5.03 (s, 2H), 3.79 (d, J = 0.8 Hz, 3H).

^{13}C NMR

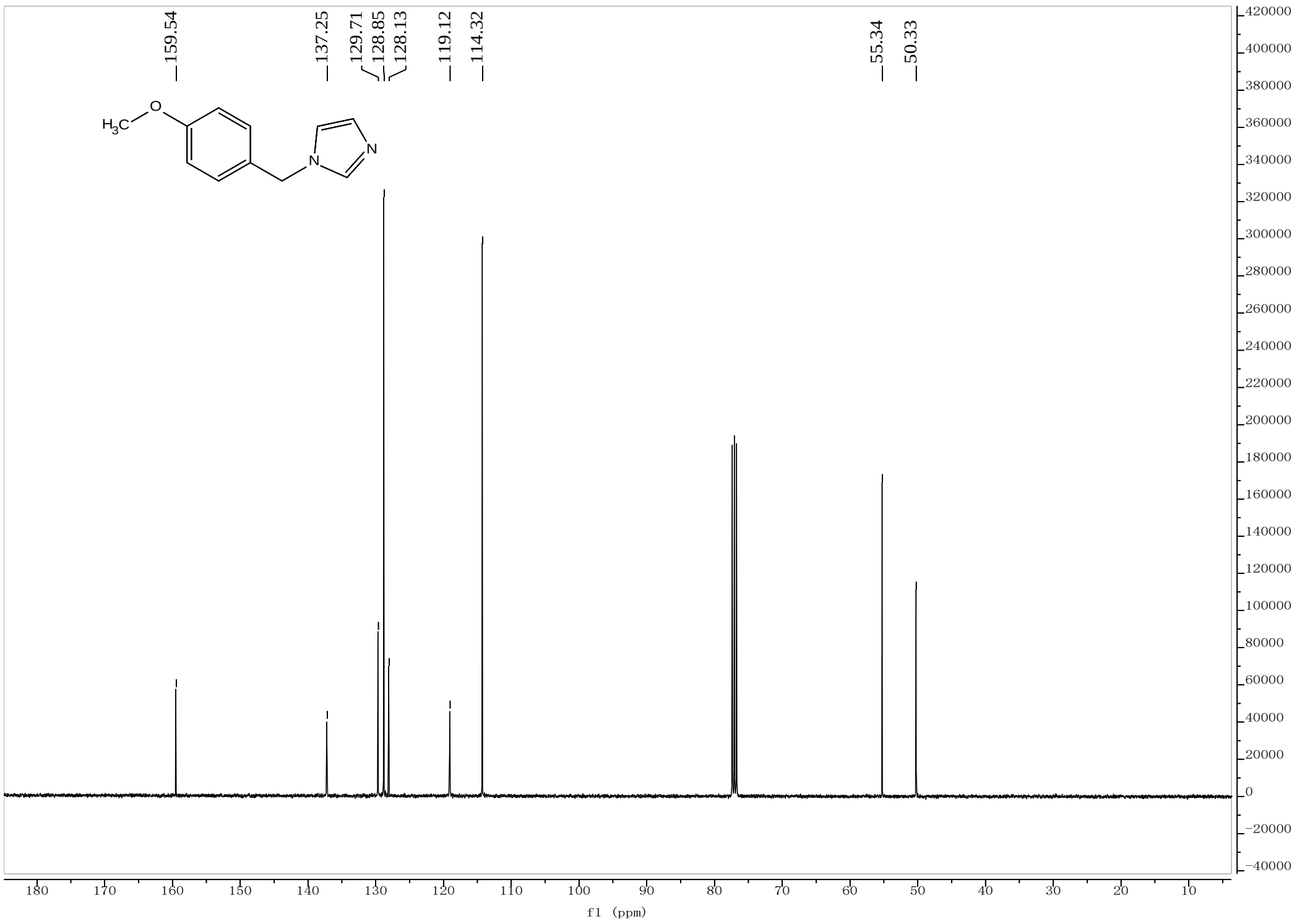

^{13}C NMR (101 MHz, Chloroform-*d*) δ 159.54, 137.25, 129.71, 128.85, 128.13, 119.12, 114.32, 55.34, 50.33.

BM-23

^{1}H NMR

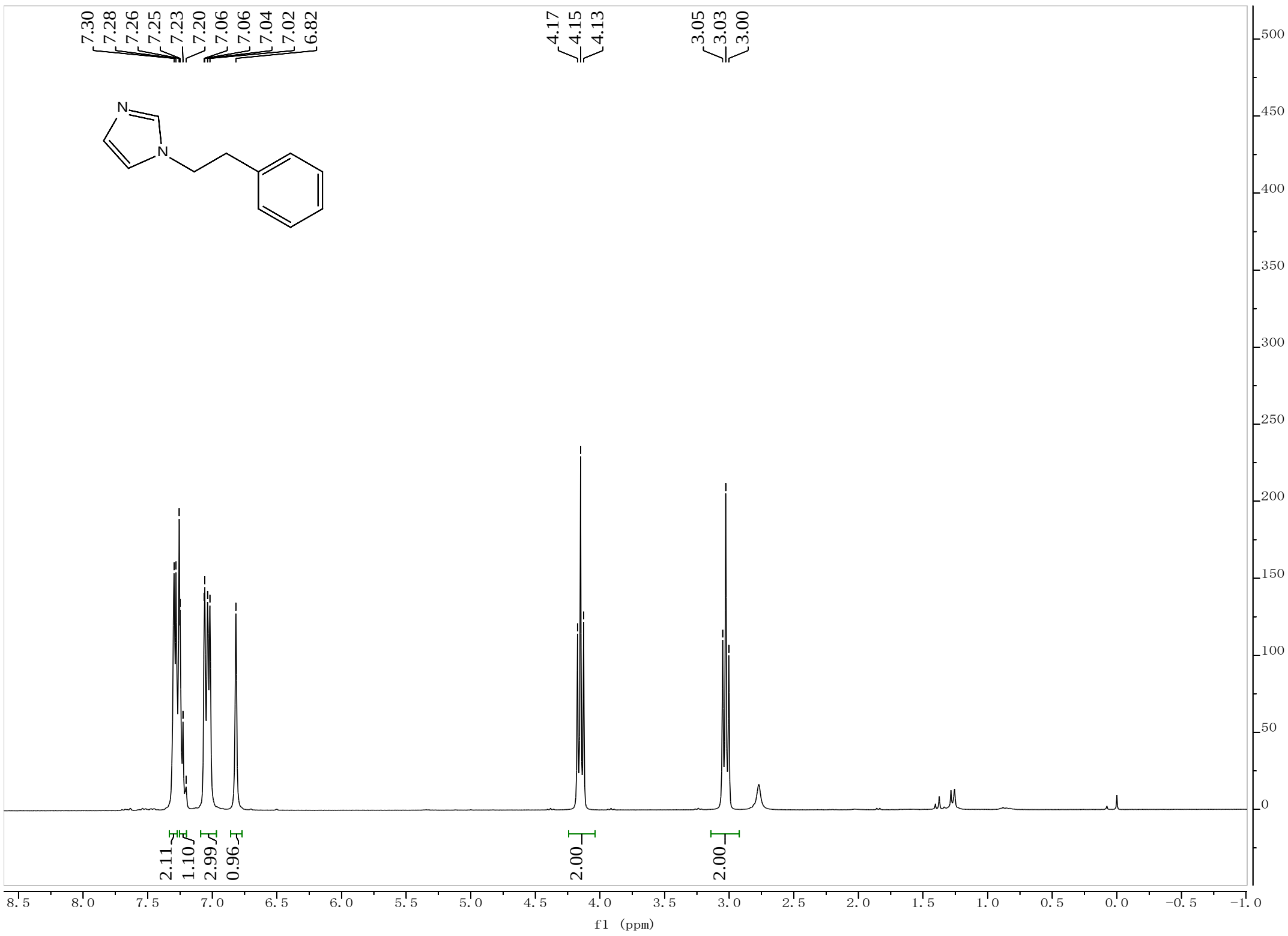

^{1}H NMR (300 MHz, Chloroform-*d*) δ 7.29 (d, J = 4.6 Hz, 2H), 7.24 (d, J = 6.6 Hz, 1H), 7.04 (dd, J = 9.8, 3.4 Hz, 3H), 6.82 (s, 1H), 4.15 (t, J = 7.1 Hz, 2H), 3.03 (t, J = 7.1 Hz, 2H).

^{13}C NMR

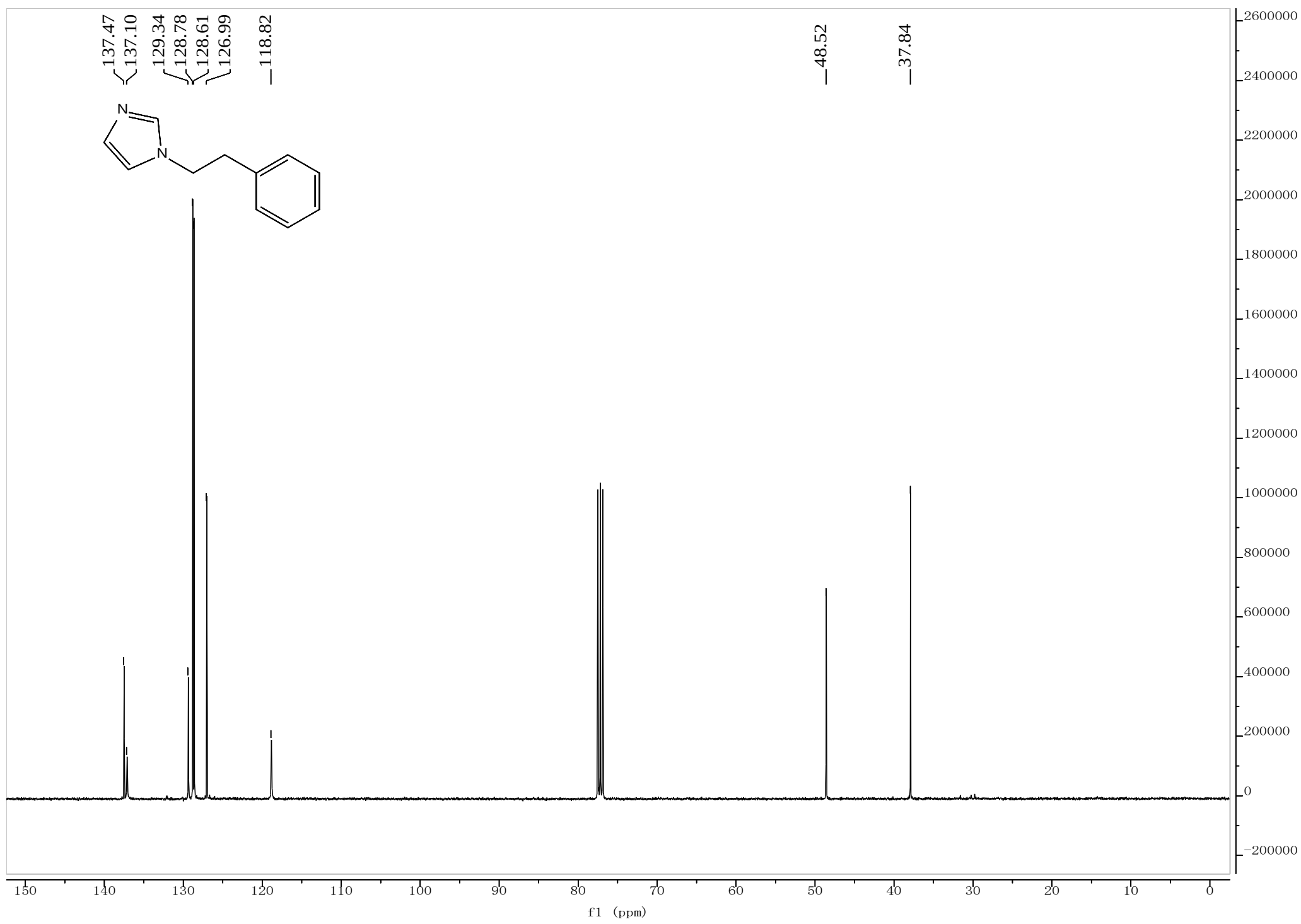

^{13}C NMR (101 MHz, Chloroform-*d*) δ 137.47, 137.10, 129.34, 128.78, 128.61, 126.99, 118.82, 48.52, 37.84.

BM-24

^{1}H NMR

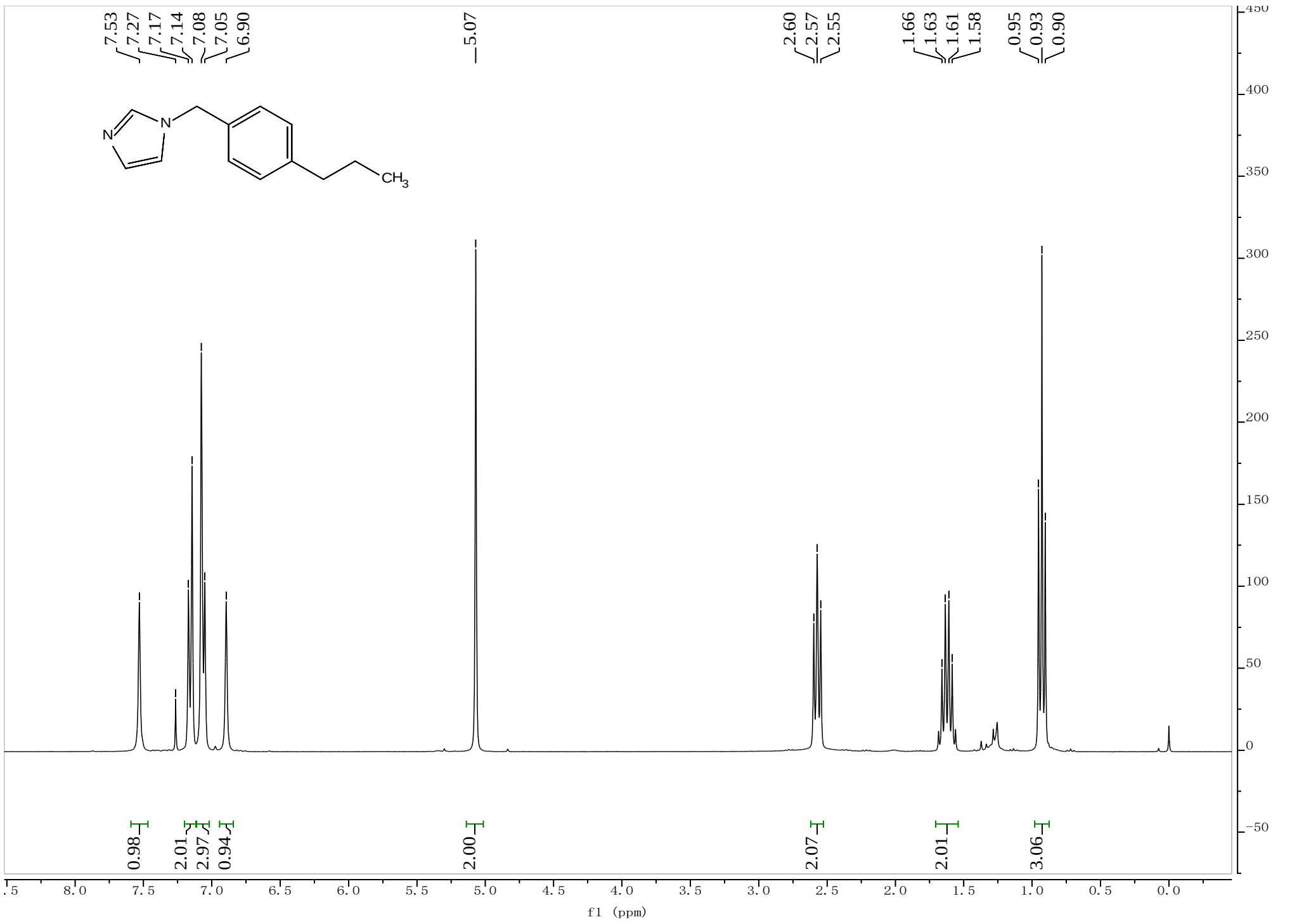

^{1}H NMR (300 MHz, Chloroform-*d*) *δ* 7.53 (s, 1H), 7.16 (d, *J* = 8.0 Hz, 2H), 7.06 (d, *J* = 7.8 Hz, 3H), 6.90 (s, 1H), 5.07 (s, 2H), 2.62-2.53 (m, 2H), 1.62 (q, *J* = 7.5 Hz, 2H), 0.93 (t, *J* = 7.3 Hz, 3H).

^{13}C NMR

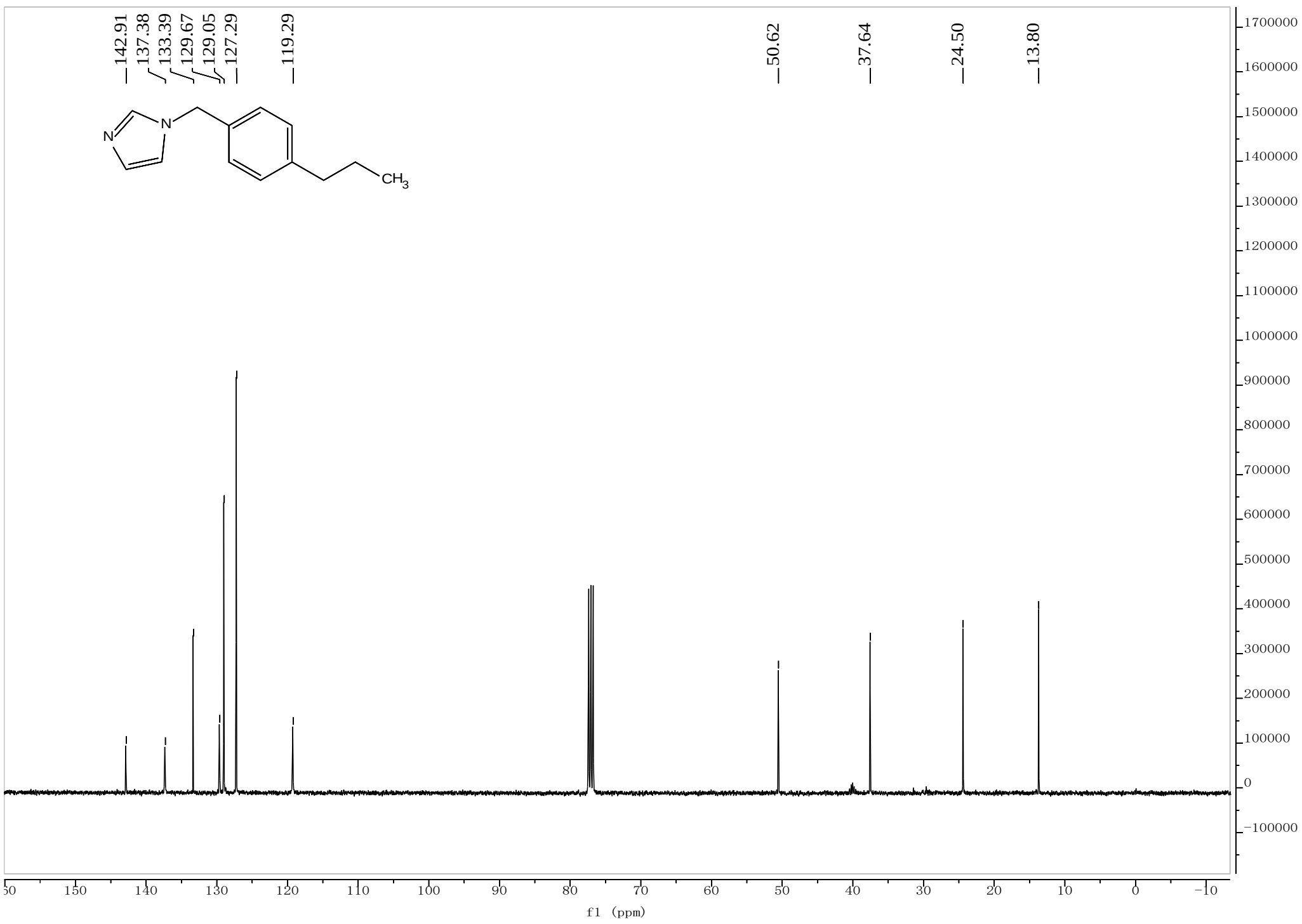

^{13}C NMR (101 MHz, Chloroform-d) *δ* 142.91, 133.39, 129.67, 129.05, 127.29, 50.62, 37.64, 24.50, 13.80.

BM-25

^1H NMR

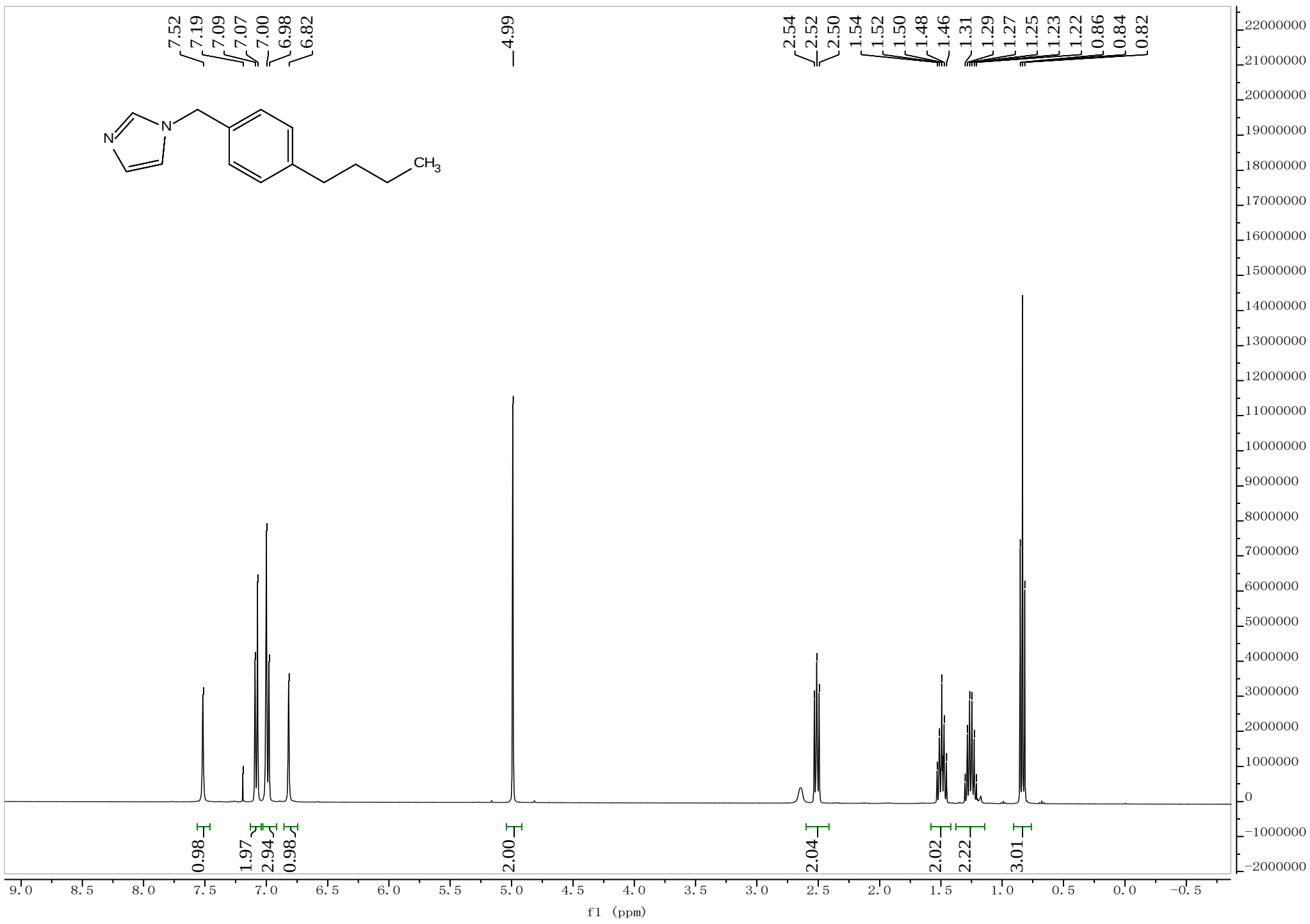

^1H NMR (400 MHz, Chloroform-*d*) δ 7.52 (s, 1H), 7.08 (d, J = 8.1 Hz, 2H), 6.99 (d, J = 8.2 Hz, 3H), 6.82 (s, 1H), 4.99 (s, 2H), 2.60-2.42 (m, 2H), 1.50 (p, J = 7.5 Hz, 2H), 1.26 (dq, J = 14.6, 7.3 Hz, 2H), 0.84 (t, J = 7.3 Hz, 3H).

^{13}C NMR

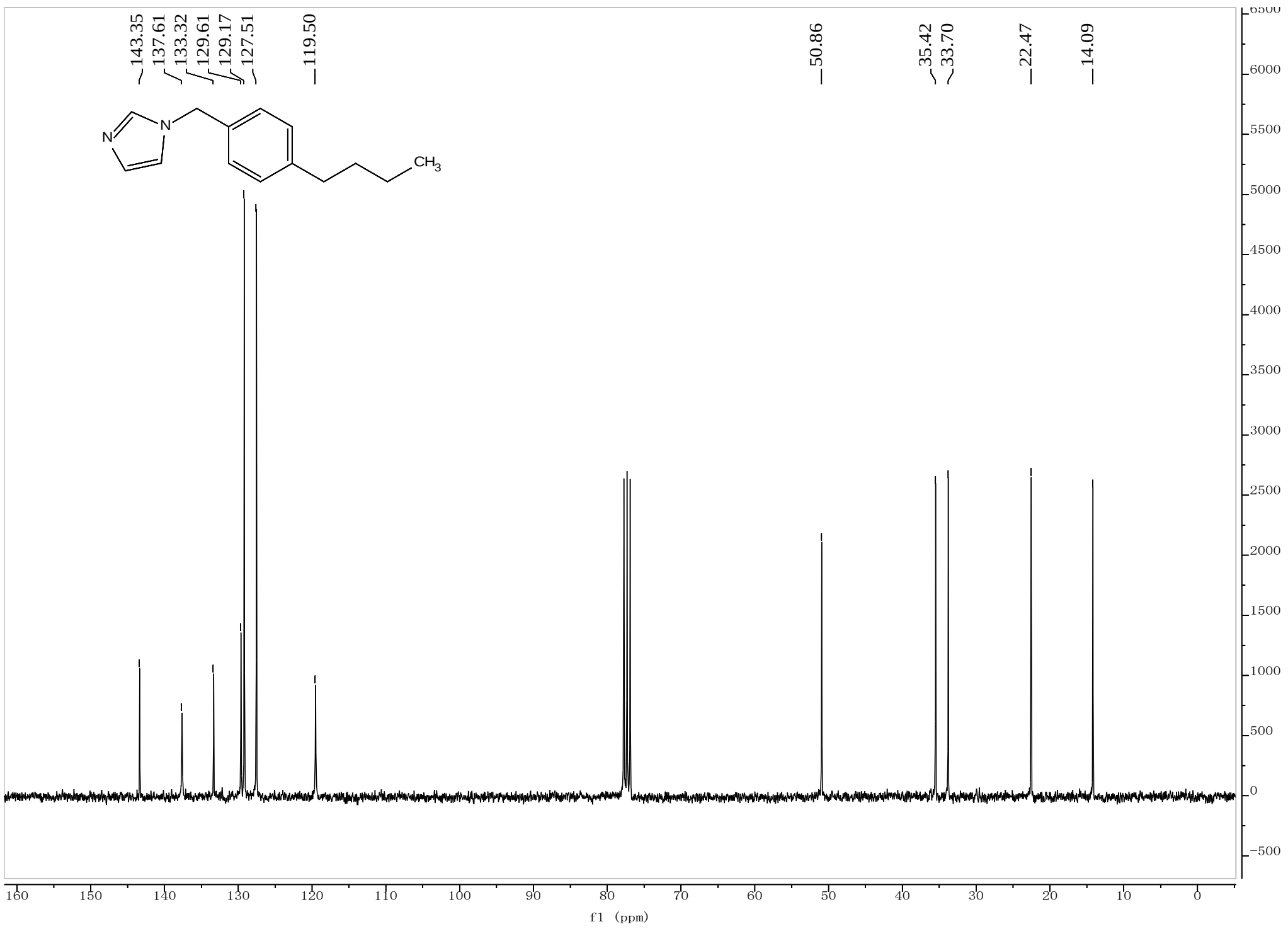

^{13}C NMR (75 MHz, Chloroform-*d*) δ 143.35, 137.61, 133.32, 129.61, 129.17, 127.51, 119.50, 50.86, 35.42, 33.70, 22.47, 14.09.

BM-26

^{1}H NMR

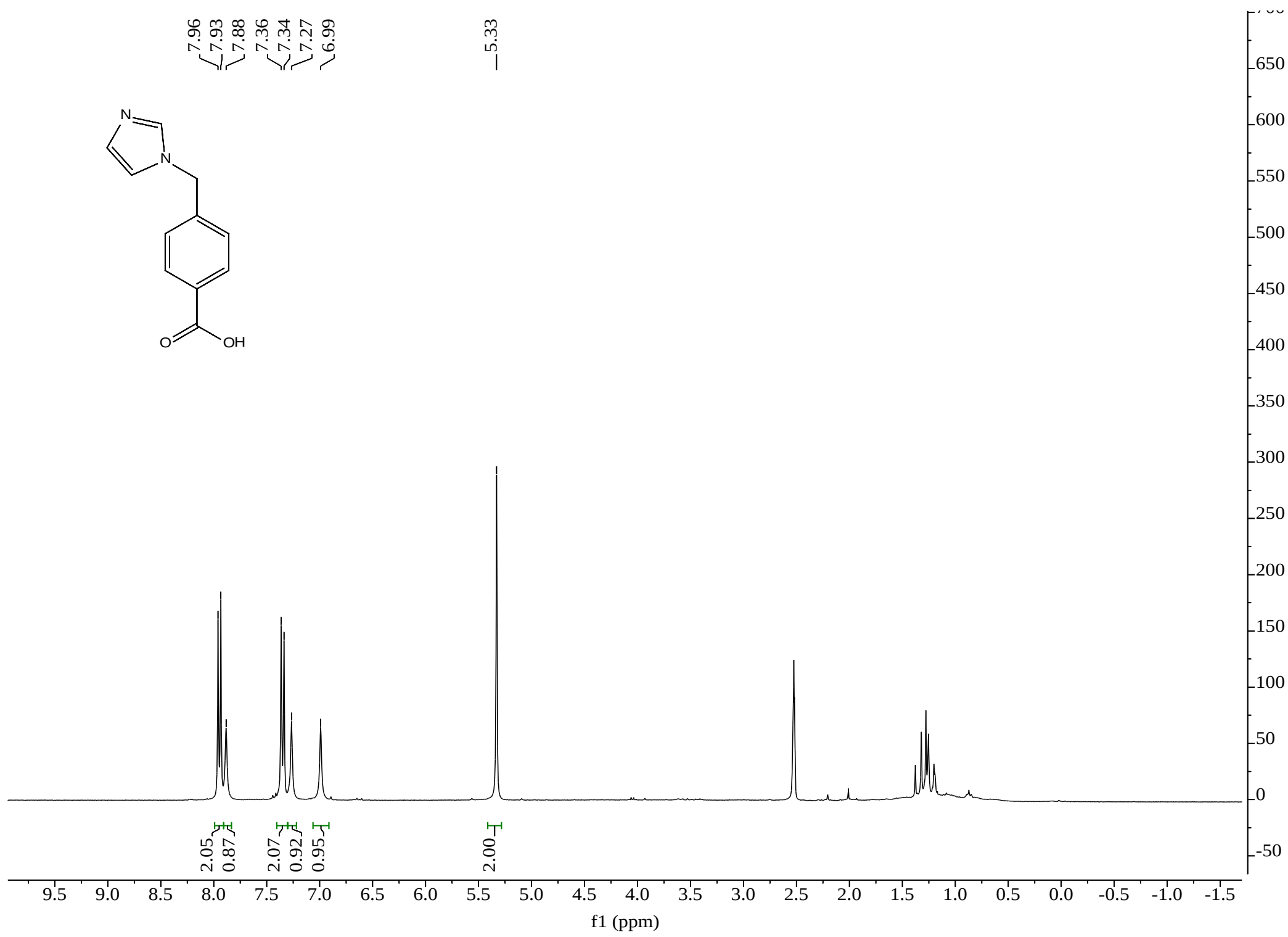

^{1}H NMR (300 MHz, DMSO-d_6) δ 7.95 (d, J = 8.2 Hz, 2H), 7.88 (s, 1H), 7.35 (d, J = 8.2 Hz, 2H), 7.27 (s, 1H), 6.99 (s, 1H), 5.33 (s, 2H).

^{13}C NMR

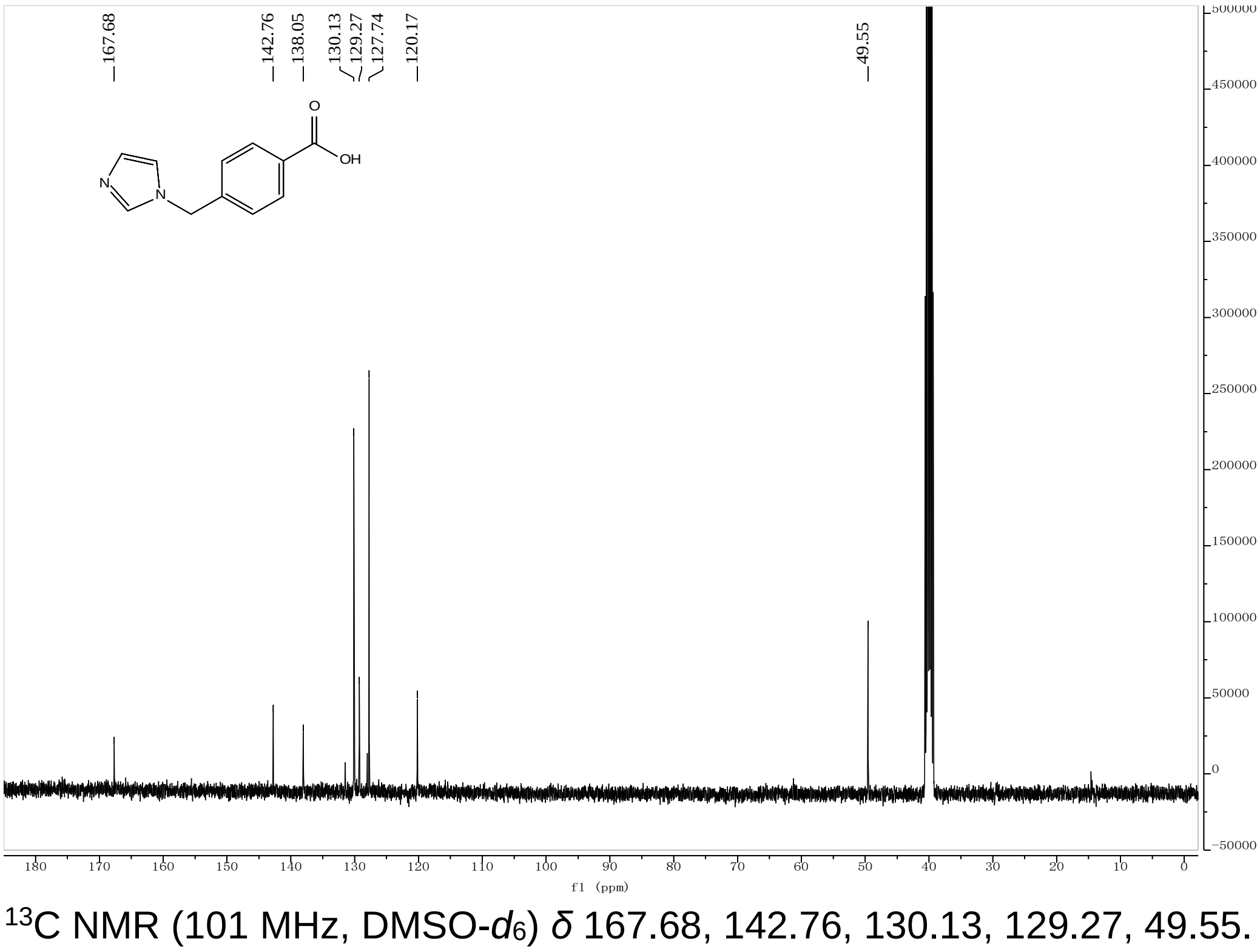

^{13}C NMR (101 MHz, DMSO-d_6) δ 167.68, 142.76, 130.13, 129.27, 49.55.

BM-27

^{1}H NMR

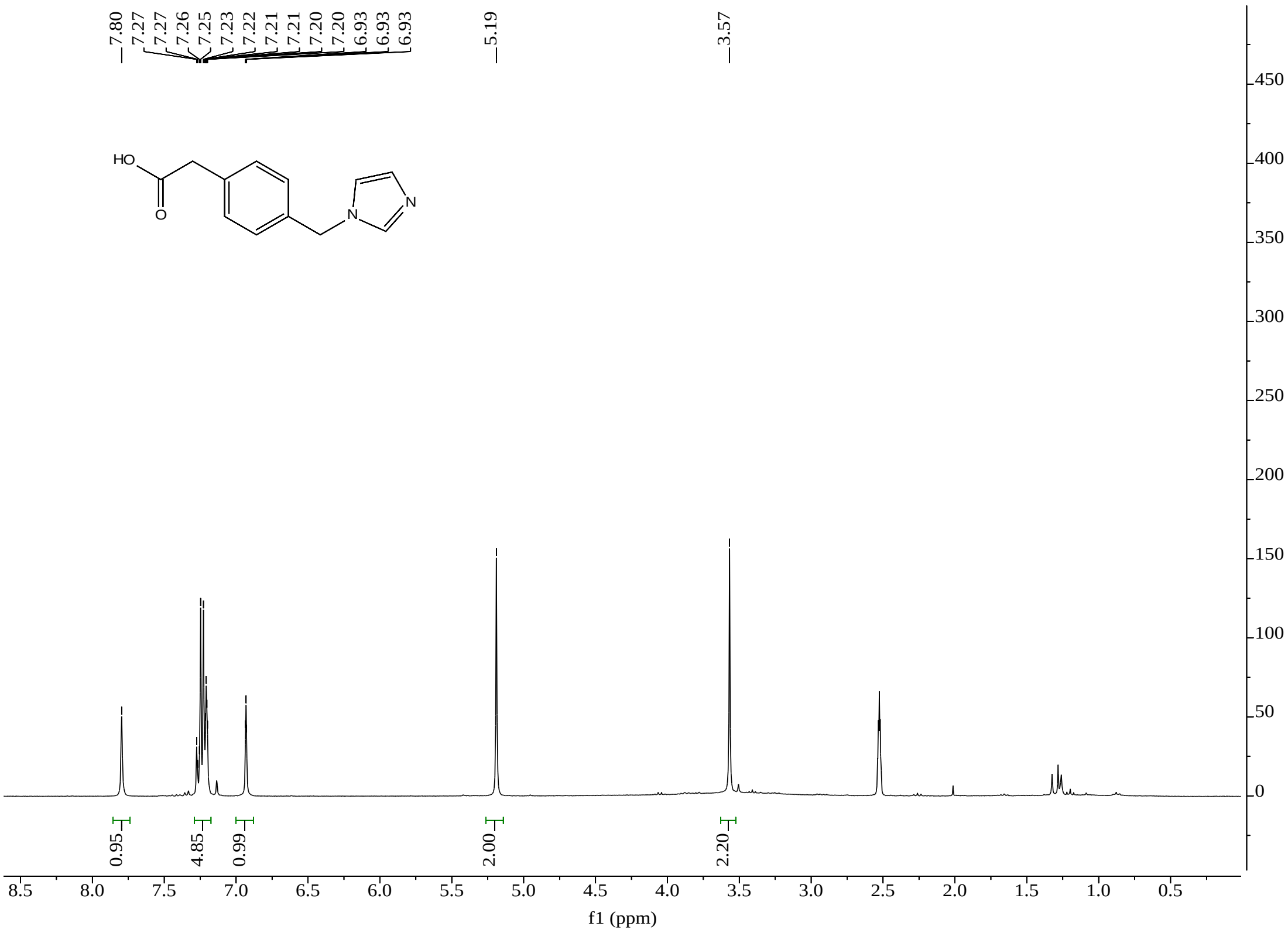

^{1}H NMR (300 MHz, DMSO-d_6) δ 7.80 (s, 1H), 7.29-7.17 (m, 5H), 6.93 (d, J = 1.1 Hz, 1H), 5.19 (s, 2H), 3.57 (s, 2H).

^{13}C NMR

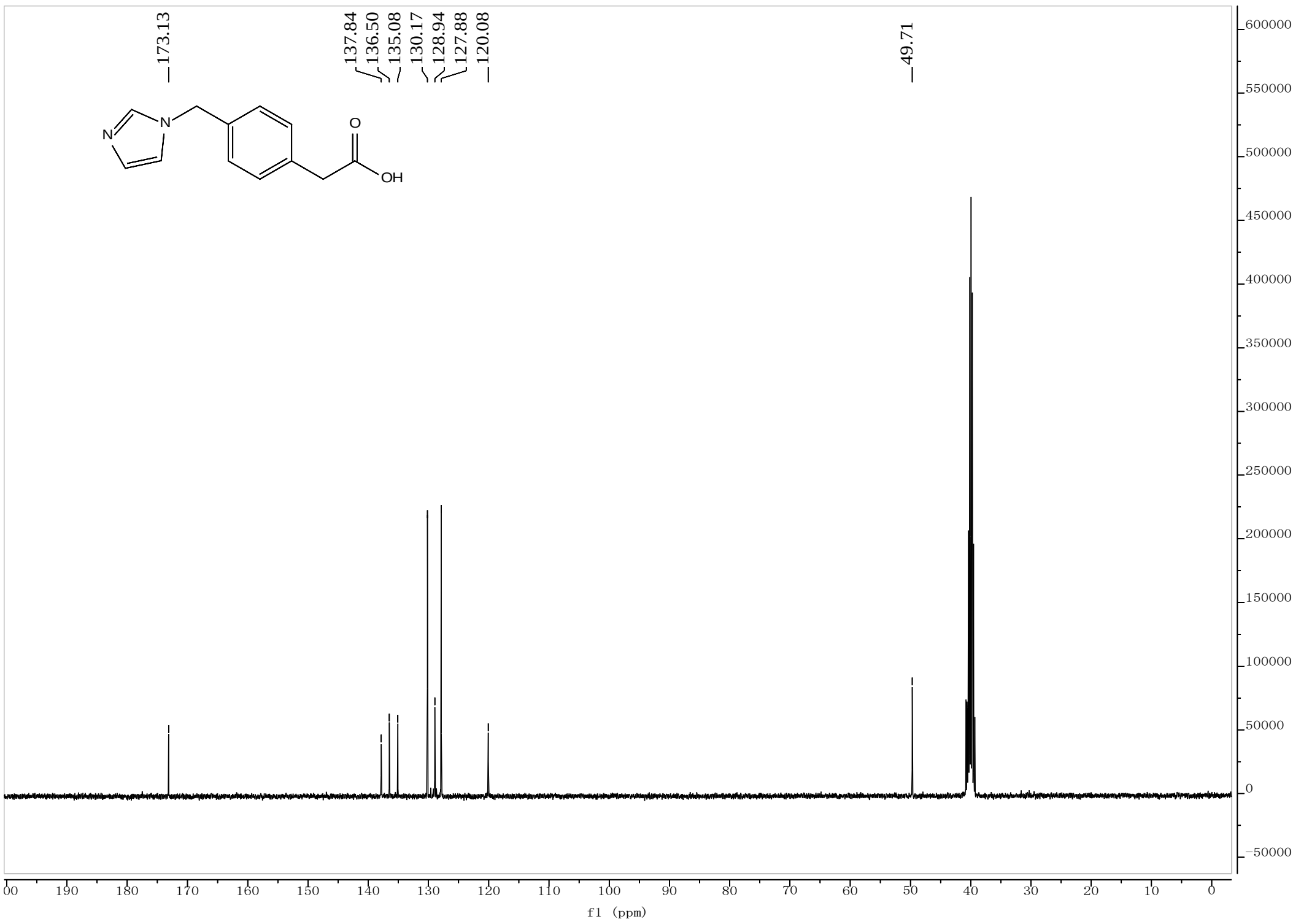

^{13}C NMR (101 MHz, DMSO-d_6) δ 173.13, 137.84, 136.50, 135.08, 130.17, 128.94, 127.88, 120.08, 49.71.

BM-28

^{1}H NMR

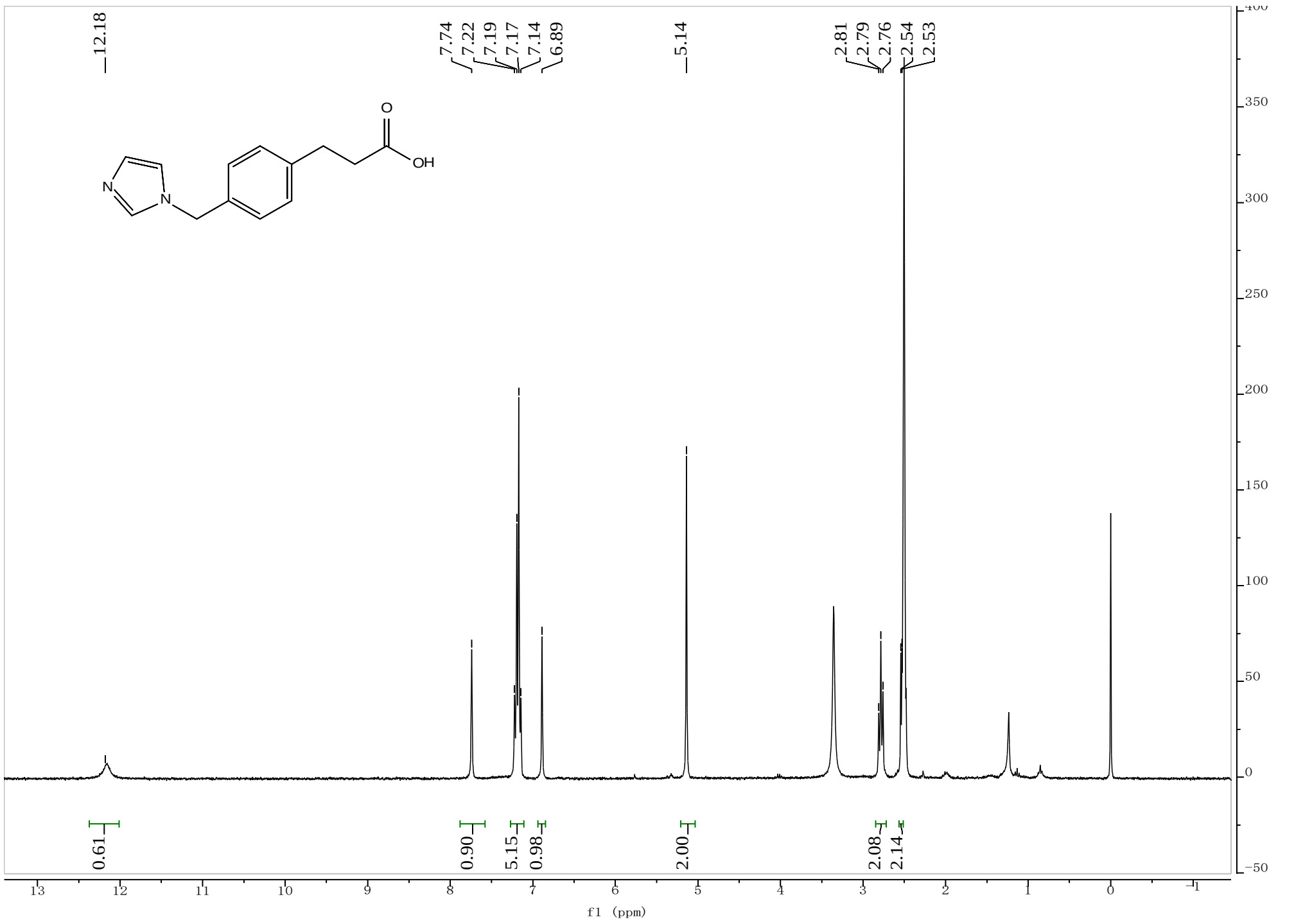

^{1}H NMR (300 MHz, DMSO-d_6) δ 12.18 (s, 1H), 7.74 (s, 1H), 7.18 (q, J = 8.2 Hz, 5H), 6.89 (s, 1H), 5.14 (s, 2H), 2.78 (t, J = 7.5 Hz, 2H), 2.54 (d, J = 4.4 Hz, 2H).

^{13}C NMR

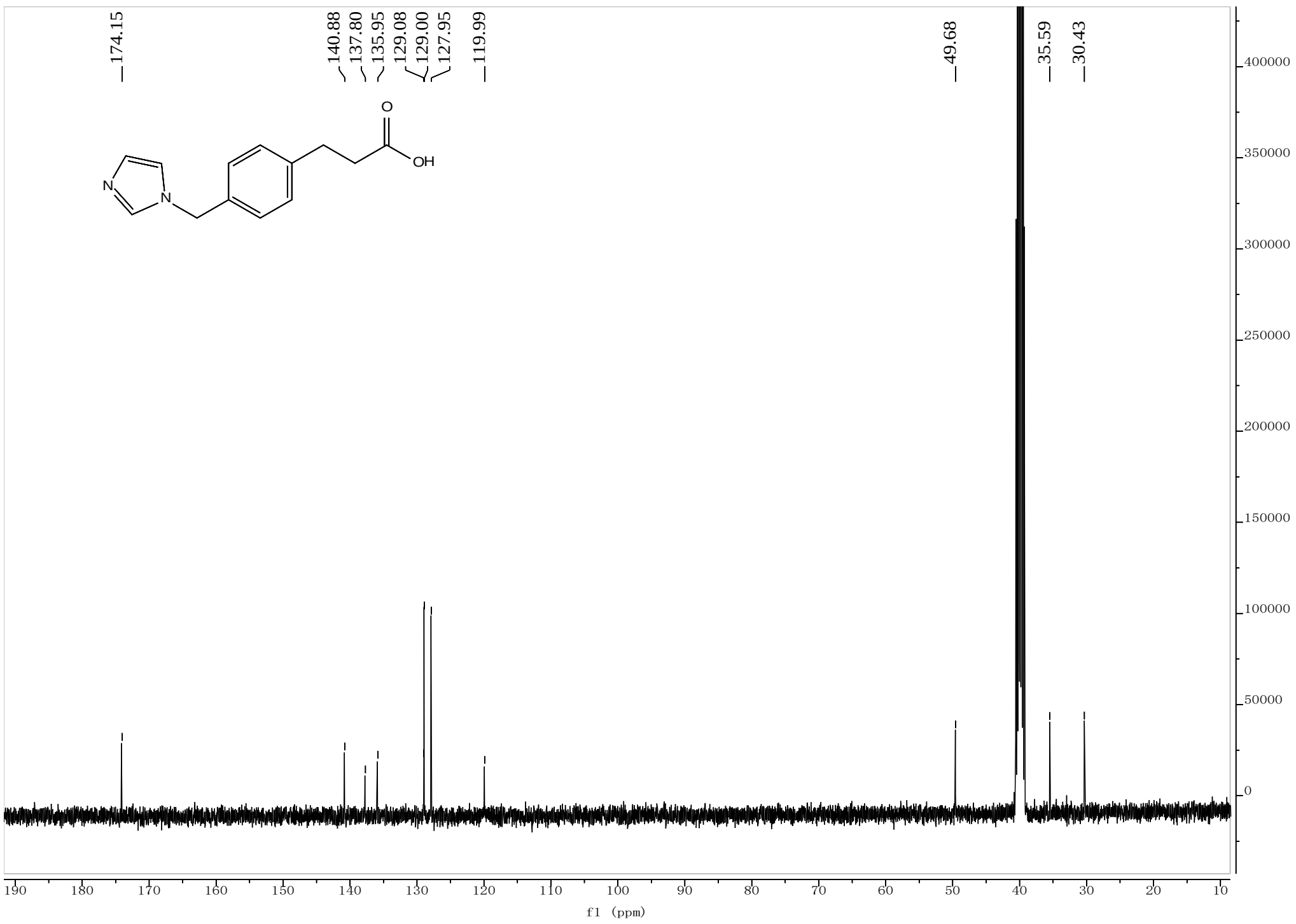

^{13}C NMR (101 MHz, DMSO-d_6) δ 174.15, 140.88, 137.80, 135.95, 129.08, 129.00, 127.95, 119.99, 49.68, 35.59, 30.43.

BM-29

^{1}H NMR

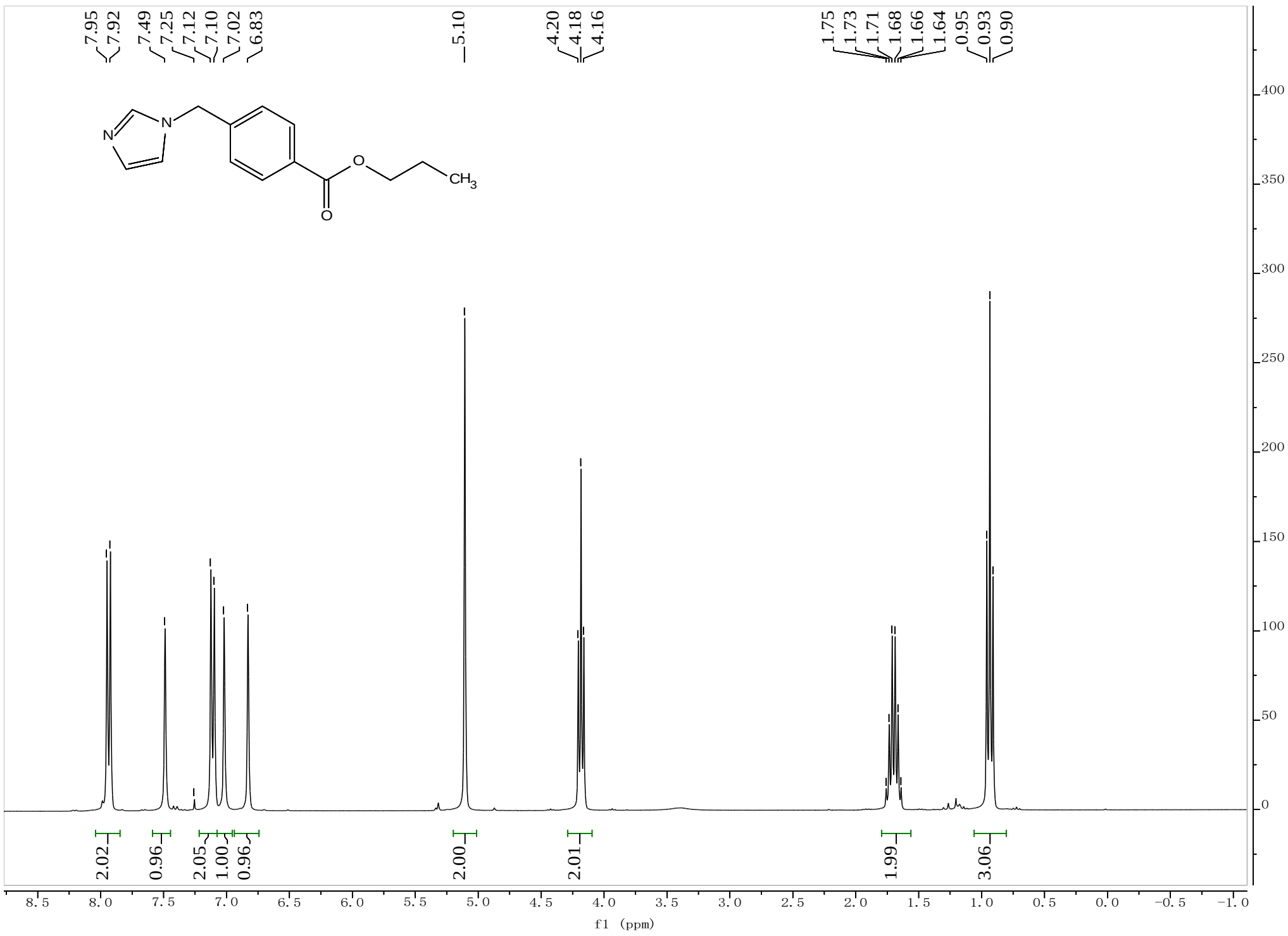

^{1}H NMR (300 MHz, Chloroform-*d*) *δ* 7.94 (d, *J* = 8.2 Hz, 2H), 7.49 (s, 1H), 7.11 (d, *J* = 8.1 Hz, 2H), 7.02 (s, 1H), 6.83 (s, 1H), 5.10 (s, 2H), 4.18 (t, *J* = 6.6 Hz, 2H), 1.69 (h, *J* = 7.1 Hz, 2H), 1.05-0.80 (m, 3H).

^{13}C NMR

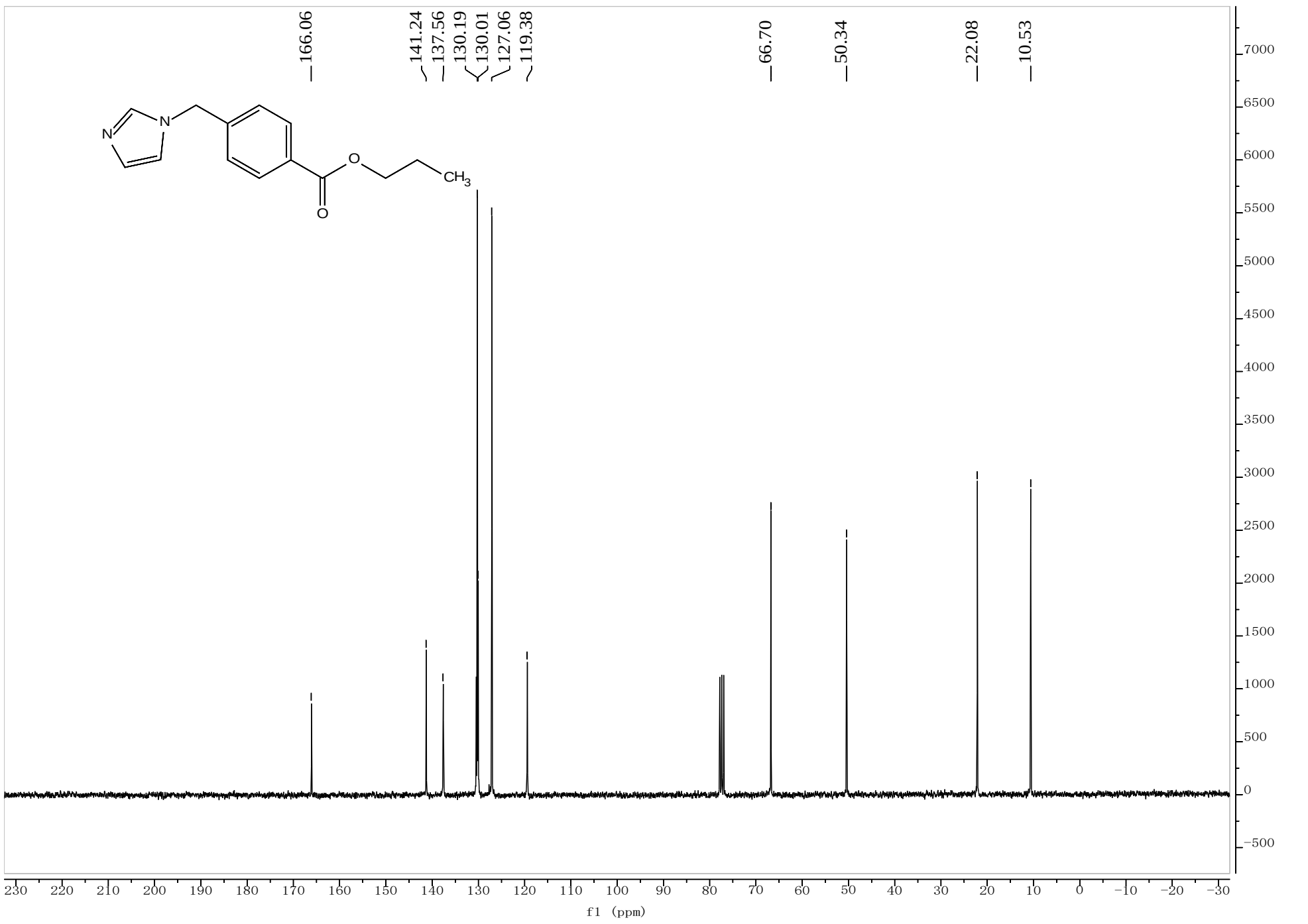

^{13}C NMR (75 MHz, Chloroform-*d*) *δ* 166.06, 141.24, 137.56, 130.19, 130.01, 127.06, 119.38, 66.70, 22.08, 10.53.

BM-30

^{1}H NMR

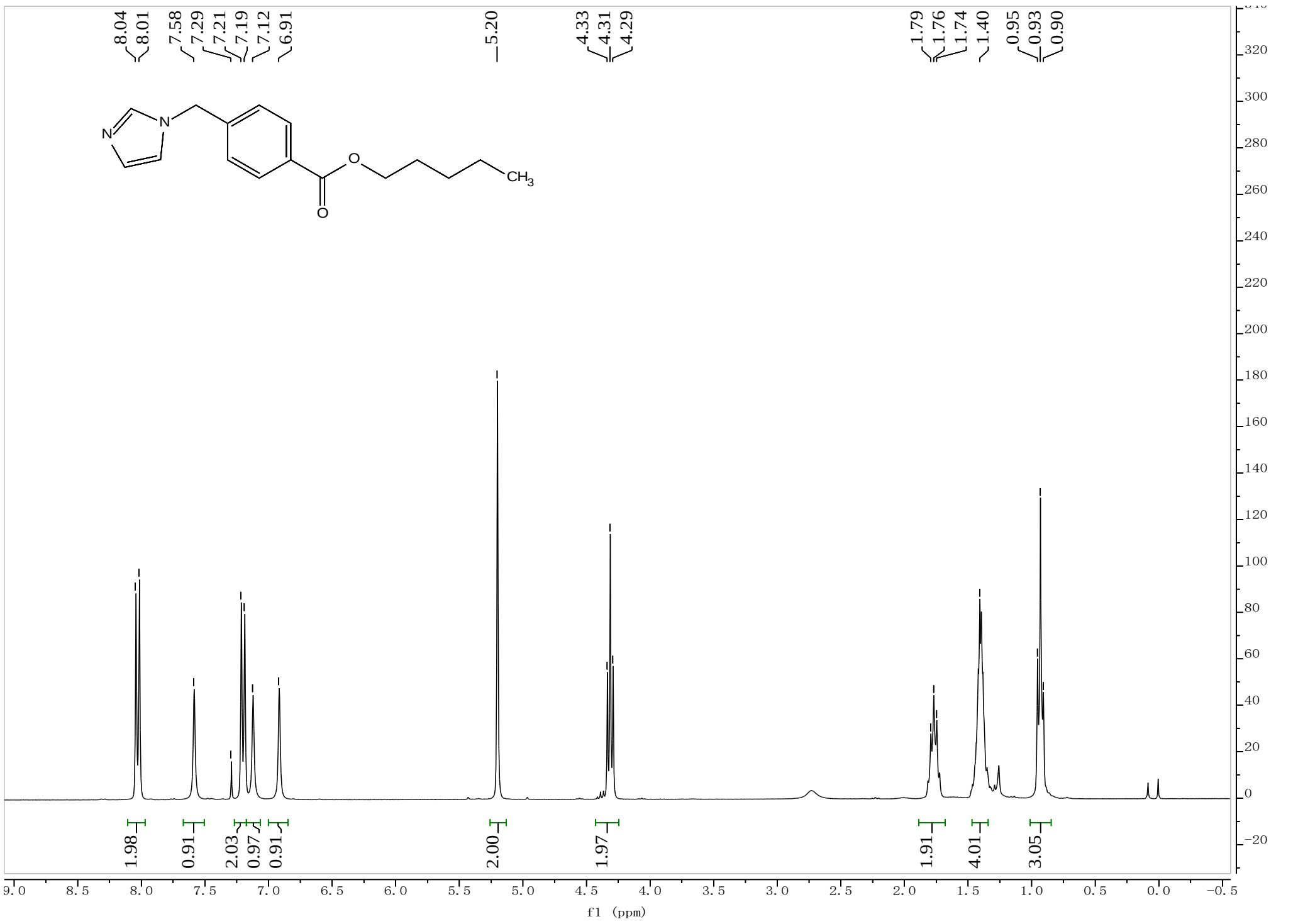

^{1}H NMR (300 MHz, Chloroform-*d*) *δ* 8.03 (d, *J* = 8.3 Hz, 2H), 7.58 (s, 1H), 7.26-7.17 (m, 2H), 7.12 (s, 1H), 6.91 (s, 1H), 5.20 (s, 2H), 4.31 (t, *J* = 6.7 Hz, 2H), 1.88-1.67 (m, 2H), 1.40 (s, 4H), 0.93 (t, *J* = 7.0 Hz, 3H).

^{13}C NMR

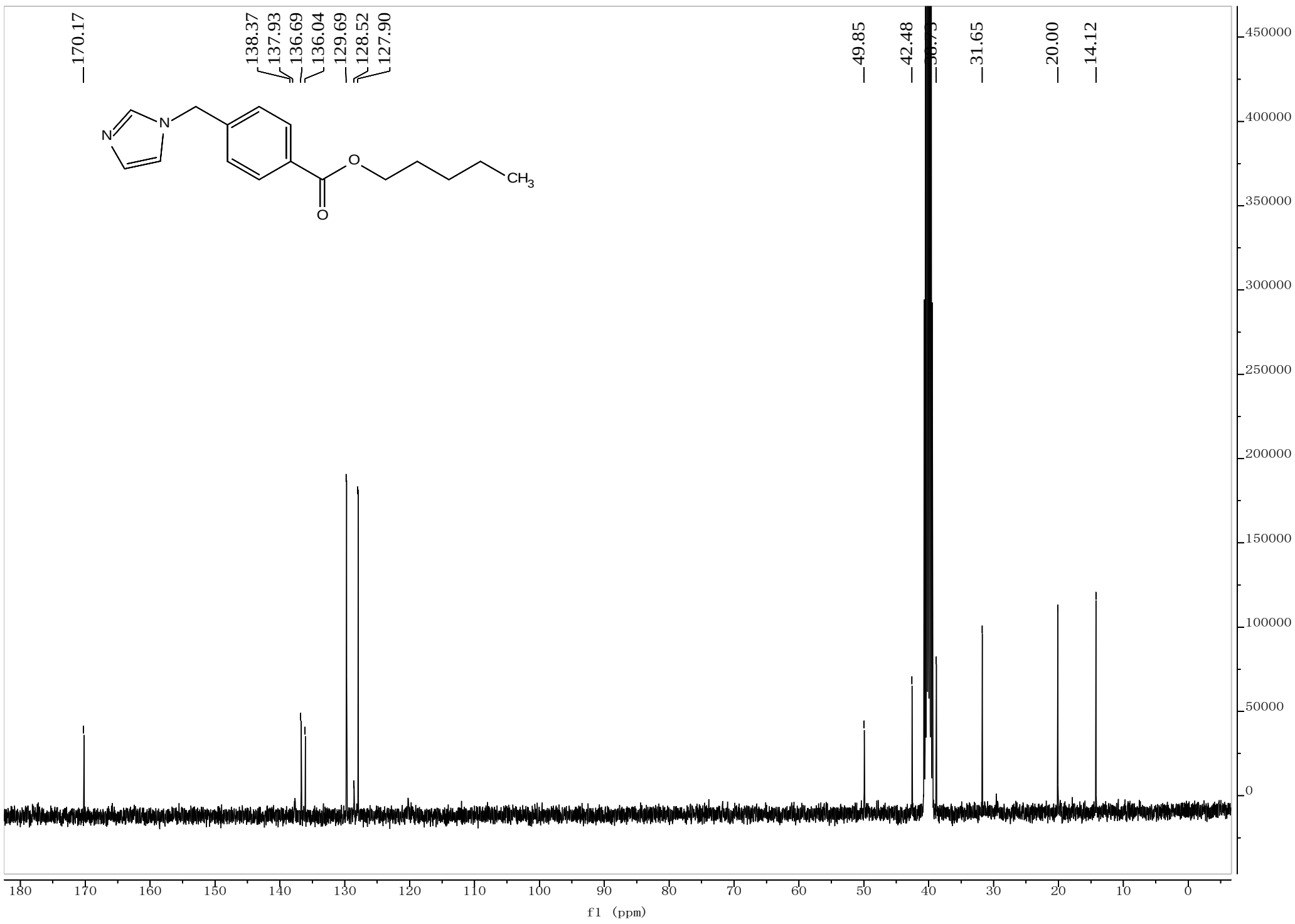

^{13}C NMR (101 MHz, DMSO-*d*$_6$) *δ* 170.17, 136.69, 136.04, 129.69, 127.90, 49.85, 38.73, 31.65, 20.00, 14.12.

BM-31

^{1}H NMR

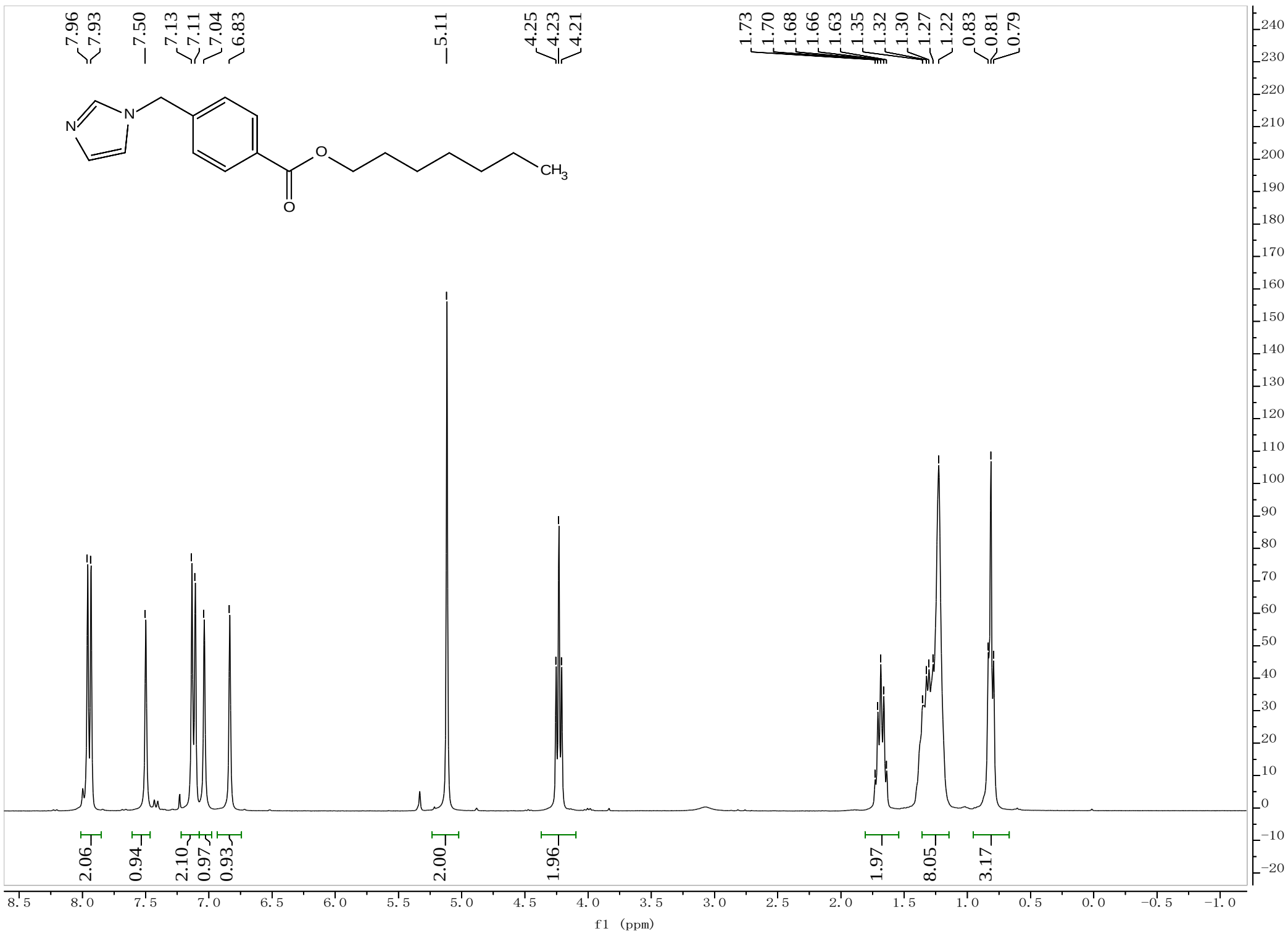

^{1}H NMR (300 MHz, Chloroform-*d*) δ 7.94 (d, J = 9.0 Hz, 2H), 7.50 (s, 1H), 7.12 (d, J = 9.0 Hz, 2H), 7.04 (S, 1H), 6.83 (s, 1H), 5.11 (s, 2H), 4.23 (t, J = 6.7 Hz, 2H), 1.68 (m, 2H), 1.27 (m, 8H), 1.35 (t, J = 6.4 Hz, 3H).

^{13}C NMR

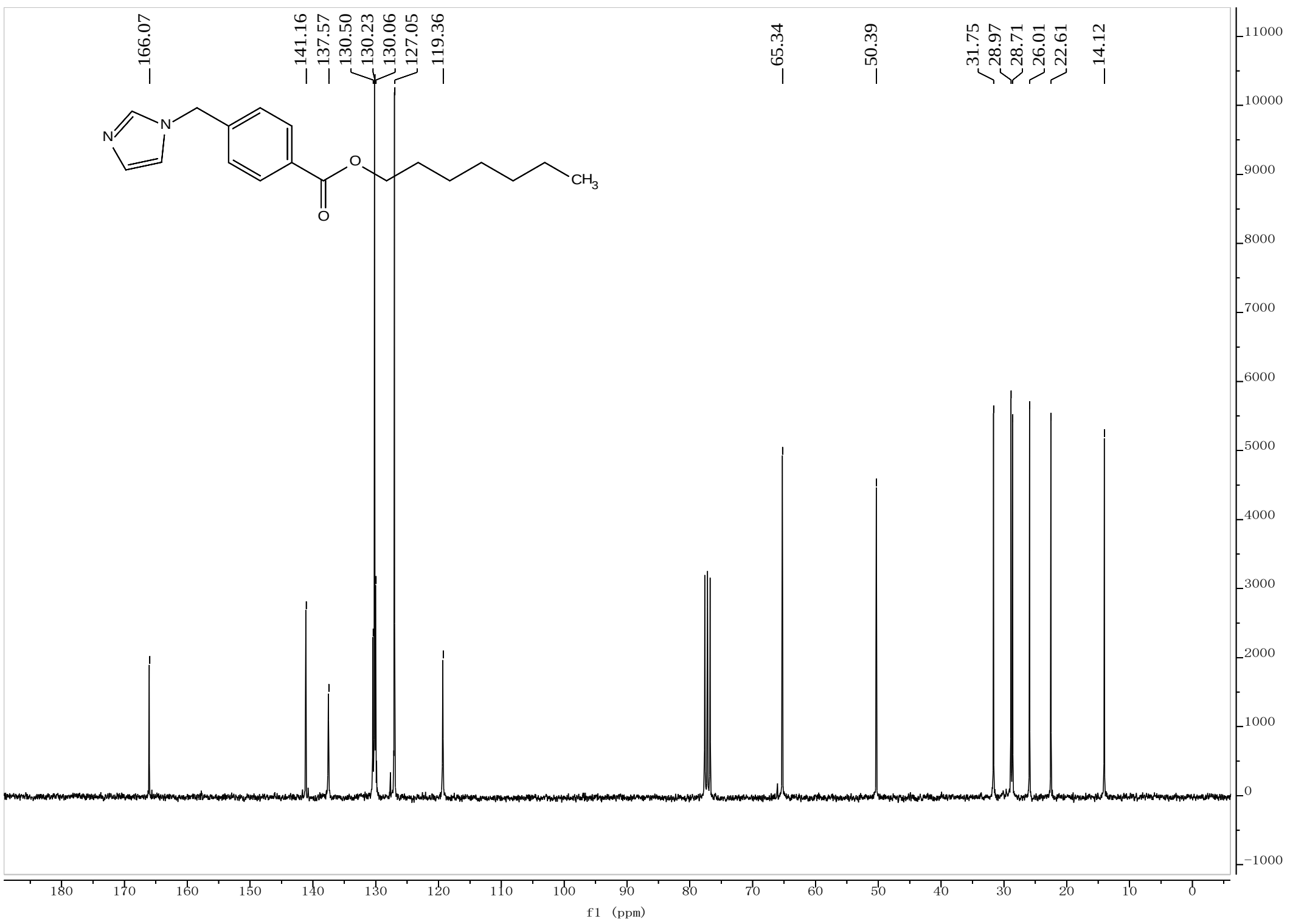

^{13}C NMR (101 MHz, Chloroform-*d*) δ 166.07, 141.16, 137.57, 130.50, 130.23,

130.06, 127.05, 119.36, 65.34, 50.39, 31.75, 28.97, 28.71, 26.06, 22.61, 14.12.

BM-32

^{1}H NMR

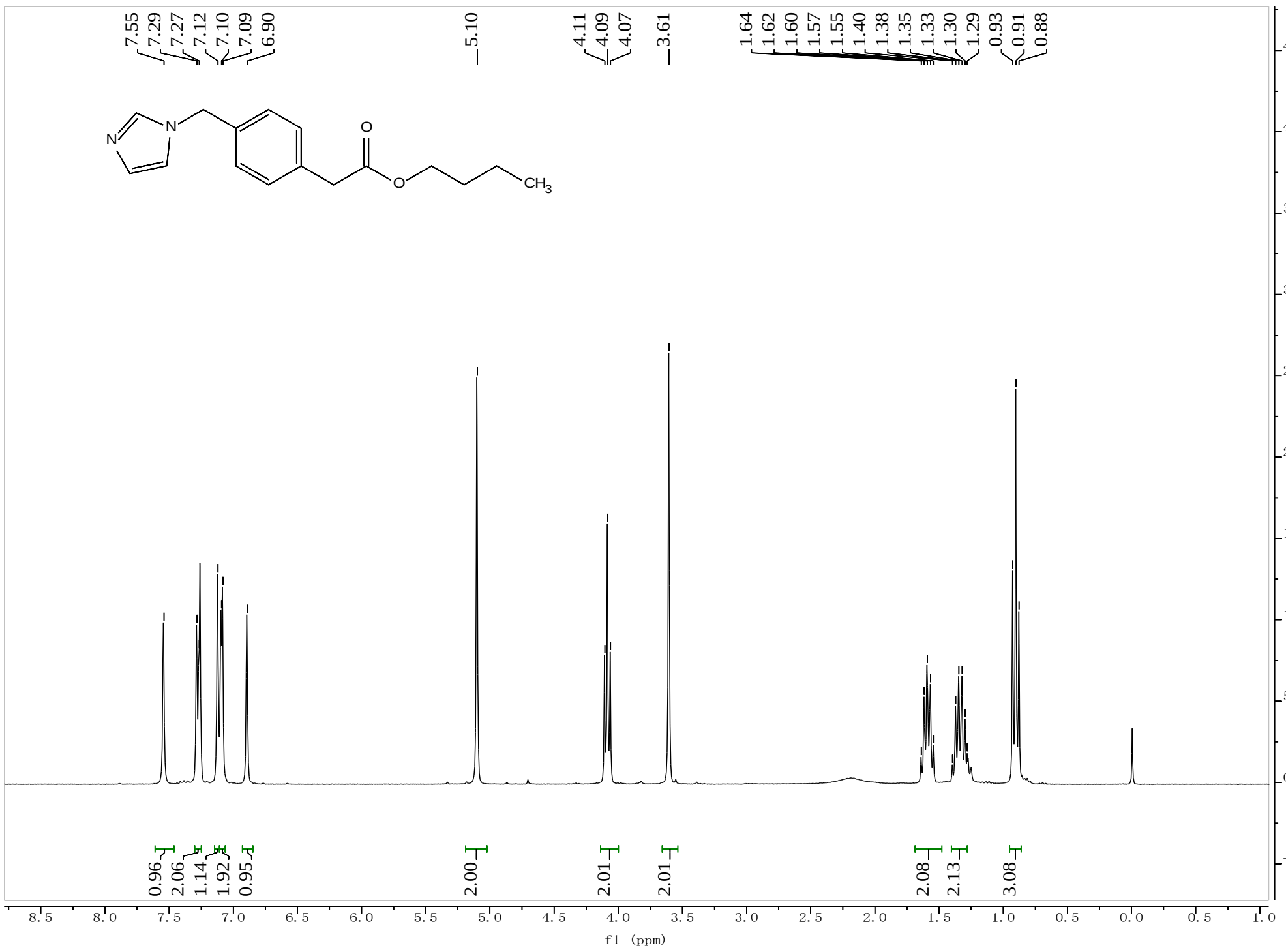

^{1}H NMR (300 MHz, Chloroform-*d*) *δ* 7.55 (s, 1H), 7.28 (d, *J* = 5.0 Hz, 2H), 7.12 (s, 1H), 7.09 (d, *J* = 3.5 Hz, 2H), 6.90 (s, 1H), 5.10 (s, 2H), 4.09 (t, *J* = 6.7 Hz, 2H), 3.61 (s, 2H), 1.60 (p, *J* = 6.8 Hz, 2H), 1.35 (dt, *J* = 14.9, 7.4 Hz, 2H), 0.91 (t, *J* = 7.3 Hz, 3H).

^{13}C NMR

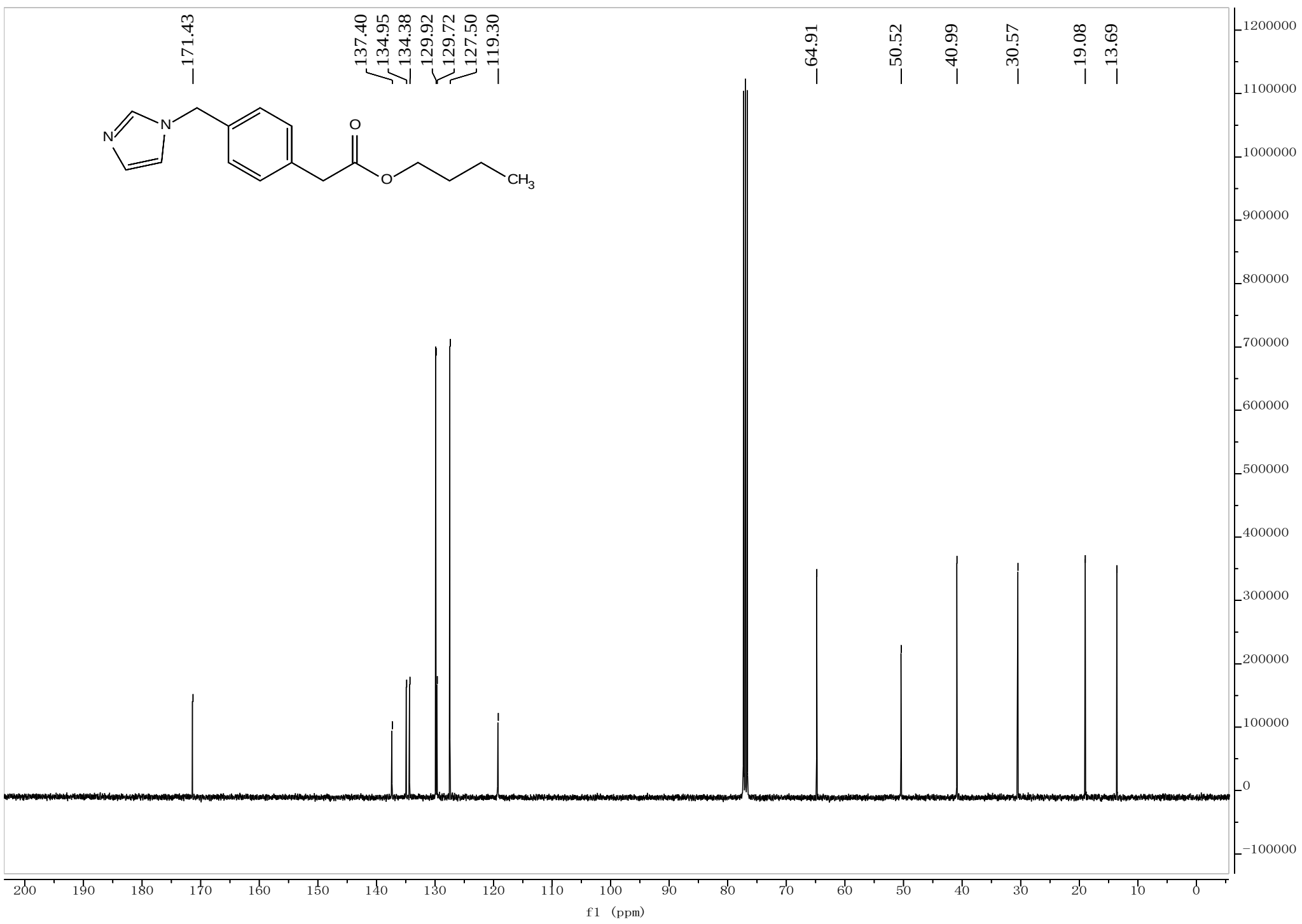

^{13}C NMR (101 MHz, Chloroform-d) *δ* 171.43, 137.40, 134.95, 134.38, 129.92, 129.72, 127.50, 64.91, 50.52, 40.99, 19.08, 13.69.

BM-33

^{1}H NMR

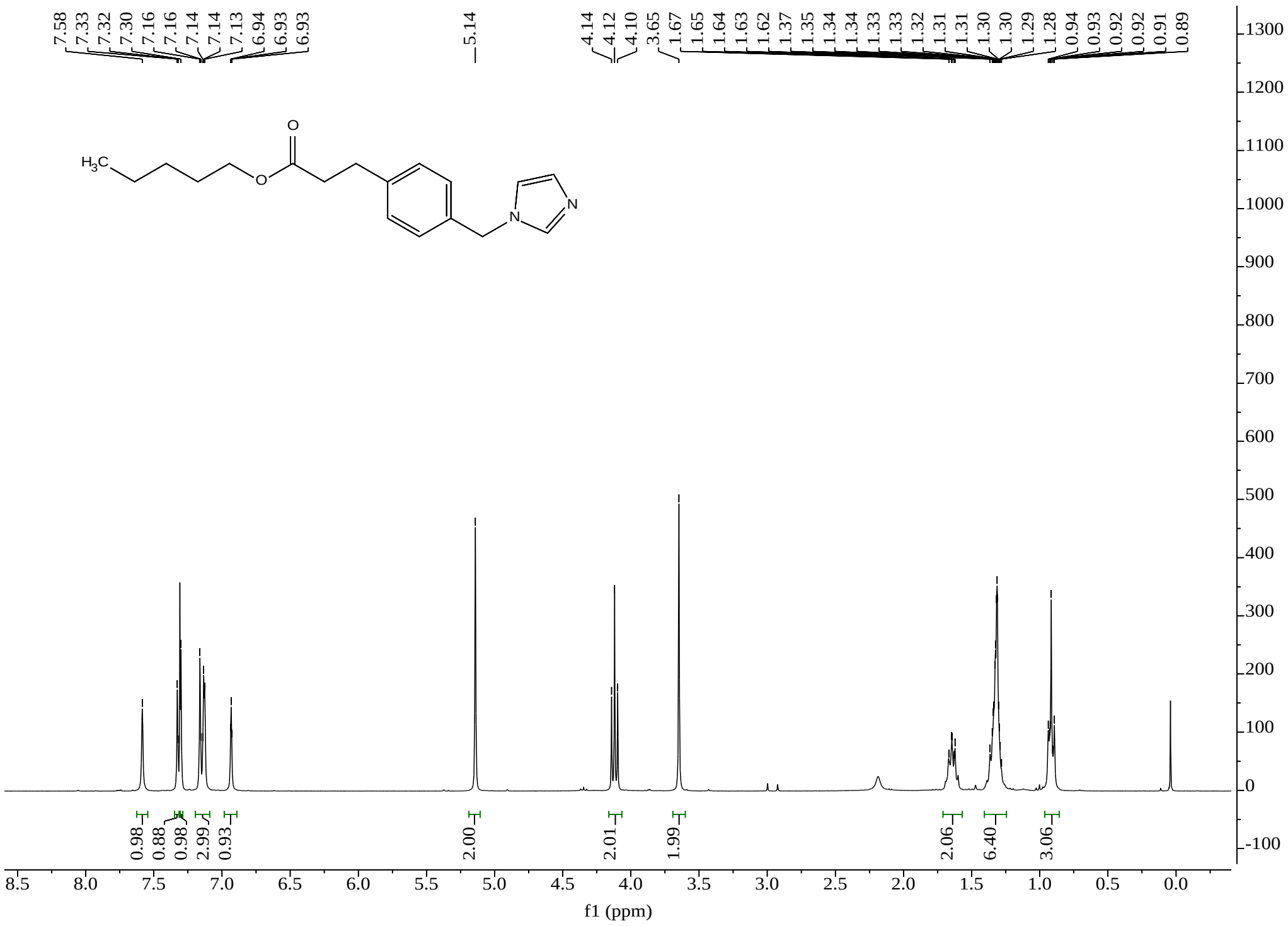

^{1}H NMR (300 MHz, Chloroform-*d*) *δ* 7.58 (s, 1H), 7.33 (d, *J* = 1.8 Hz, 1H), 7.30 (s, 1H), 7.19-7.09 (m, 3H), 6.94 (d, *J* = 1.3 Hz, 1H), 5.14 (s, 2H), 4.12 (t, *J* = 6.7 Hz, 2H), 3.65 (s, 2H), 1.71-1.57 (m, 2H), 1.41-1.24 (m, 6H), 0.96-0.86 (m, 3H).

^{13}C NMR

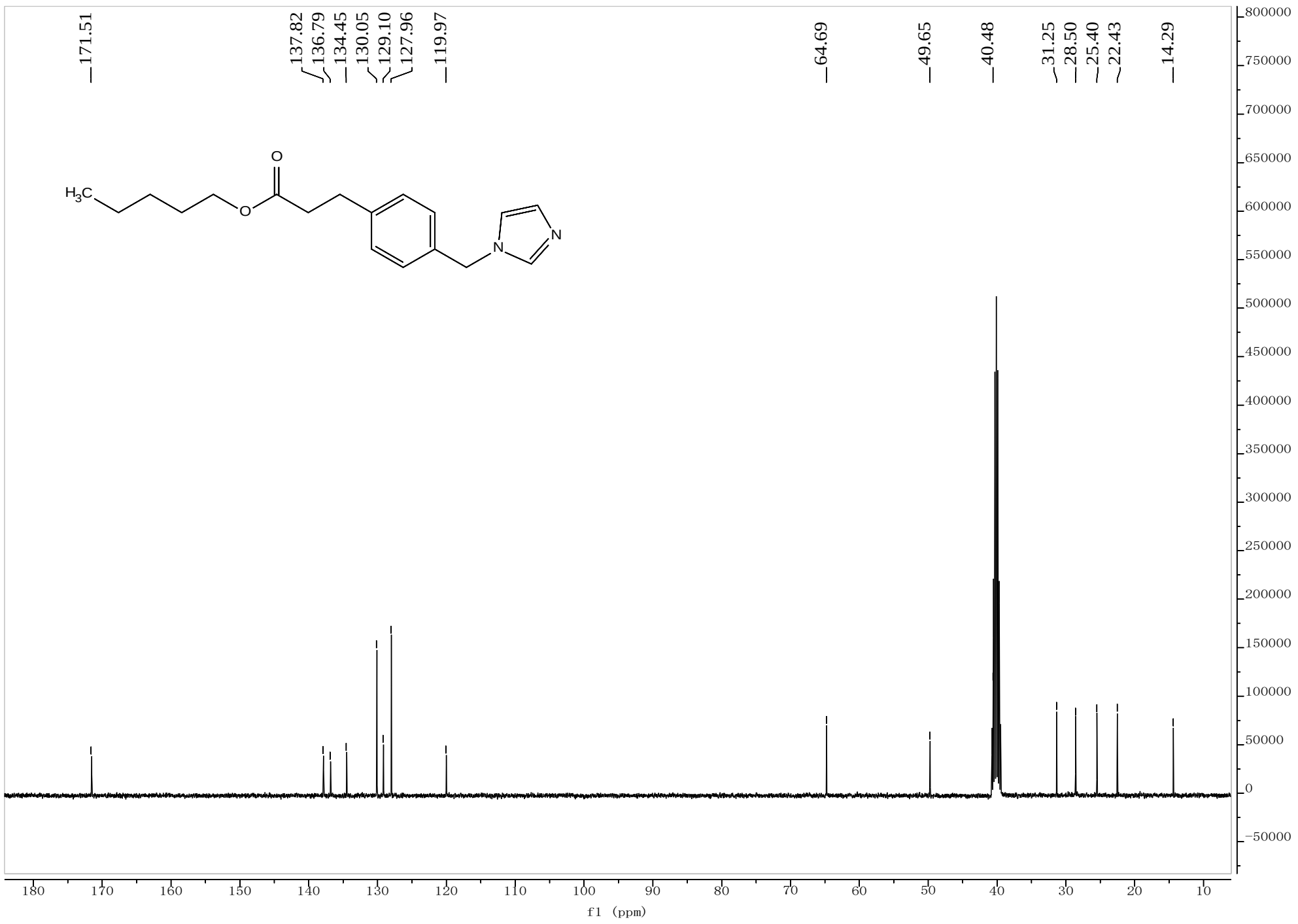

^{13}C NMR (101 MHz, DMSO-*d*$_6$) *δ* 171.51, 137.82, 136.79, 134.45, 130.05, 129.10, 127.96, 119.97, 64.69, 49.65, 40.48, 31.25, 28.50, 25.40, 22.43, 14.29.

BM-34

^{1}H NMR

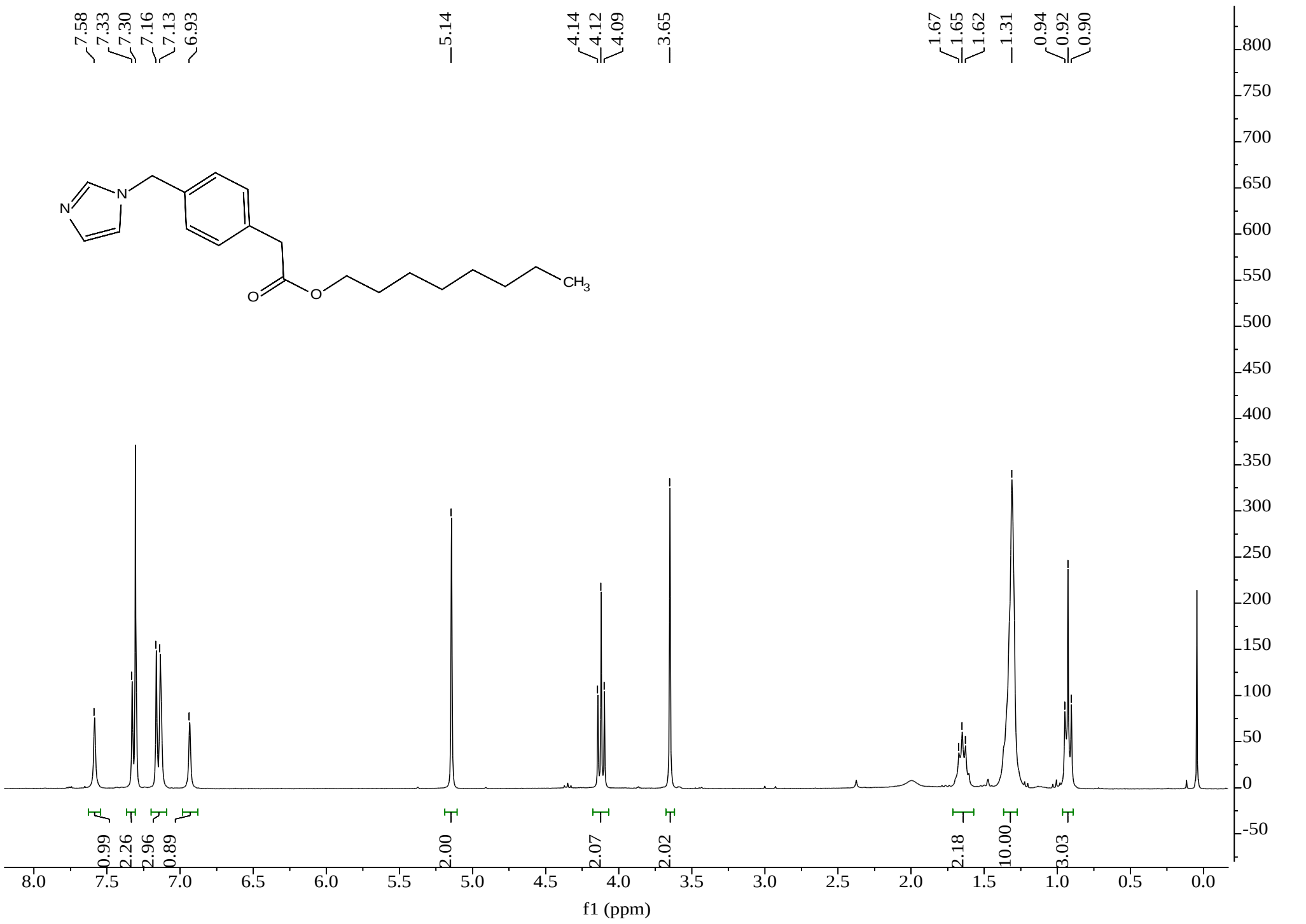

^{1}H NMR (300 MHz, Chloroform-*d*) *δ* 7.58 (s, 1H), 7.33 (s, 2H), 7.15 (d, *J* = 8.3 Hz, 3H), 6.93 (s, 1H), 5.14 (s, 2H), 4.12 (t, *J* = 6.7 Hz, 2H), 3.65 (s, 2H), 1.71-1.57 (m, 2H), 1.31 (s, 10H), 0.96-0.89 (m, 3H).

^{13}C NMR

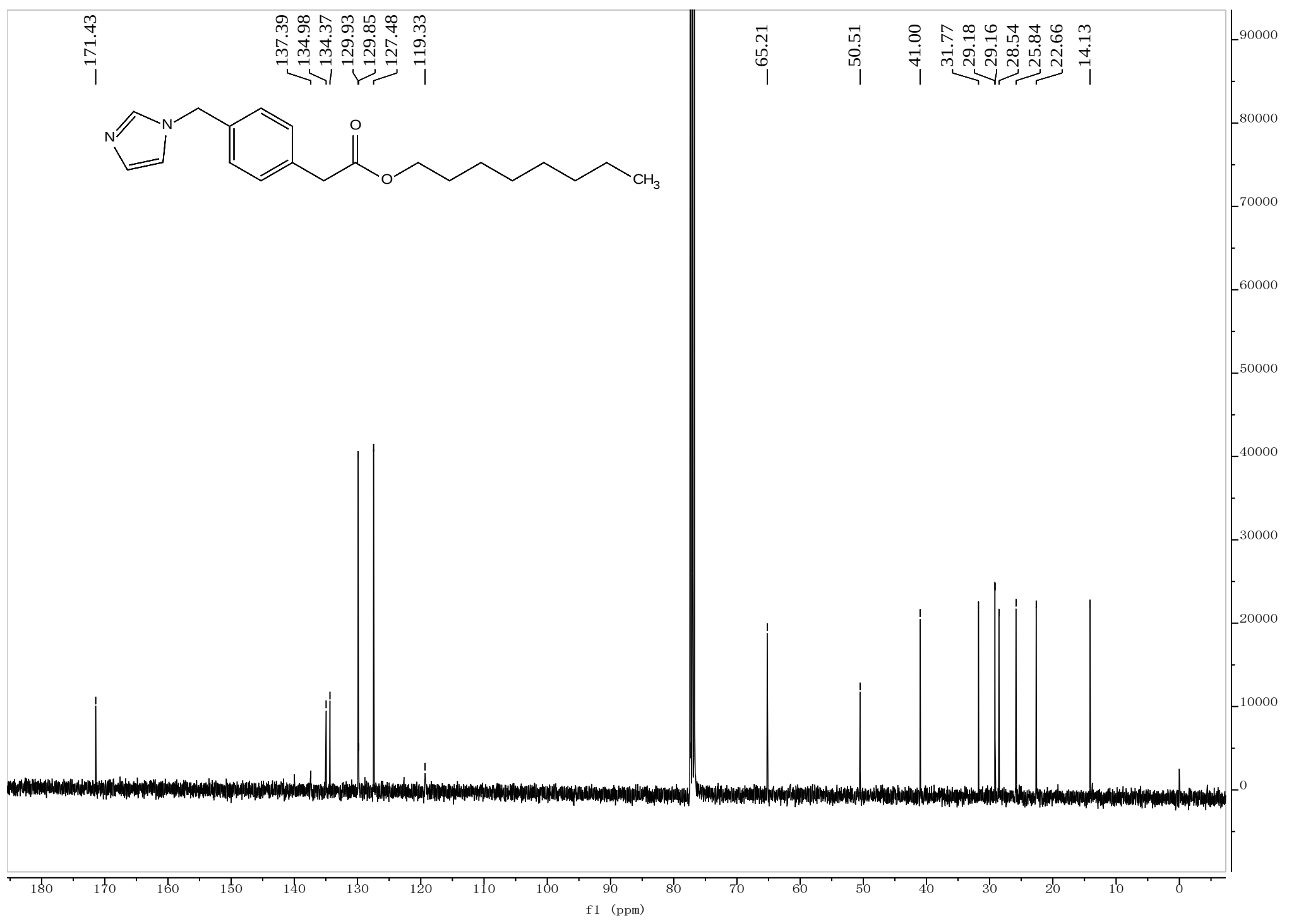

^{13}C NMR (101 MHz, Chloroform-*d*) *δ* 171.43, 134.98, 134.37, 129.93, 129.85, 127.48, 65.21, 50.51, 41.00, 31.77, 29.18, 29.16, 28.54, 25.84, 22.66, 14.13.

BM-35

^{1}H NMR

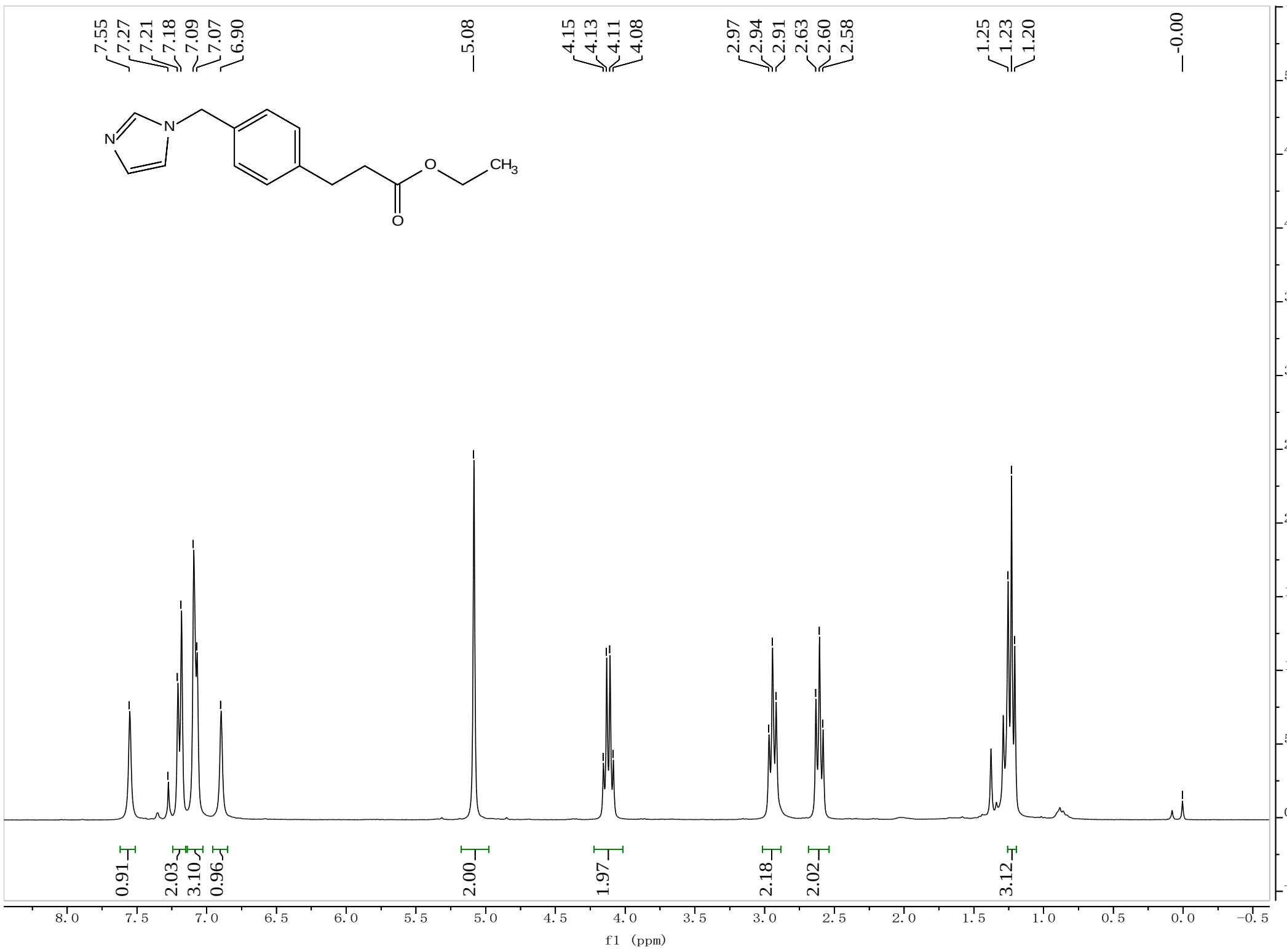

^{1}H NMR (300 MHz, Chloroform-*d*) *δ* 7.55 (s, 1H), 7.19 (d, *J* = 7.8 Hz, 2H), 7.08 (d, *J* = 7.4 Hz, 3H), 6.90 (s, 1H), 5.08 (s, 2H), 4.12 (q, *J* = 7.1 Hz, 2H), 2.94 (t, *J* = 7.7 Hz, 2H), 2.60 (t, *J* = 7.7 Hz, 2H), 1.23 (t, *J* = 7.2 Hz, 3H).

^{13}C NMR

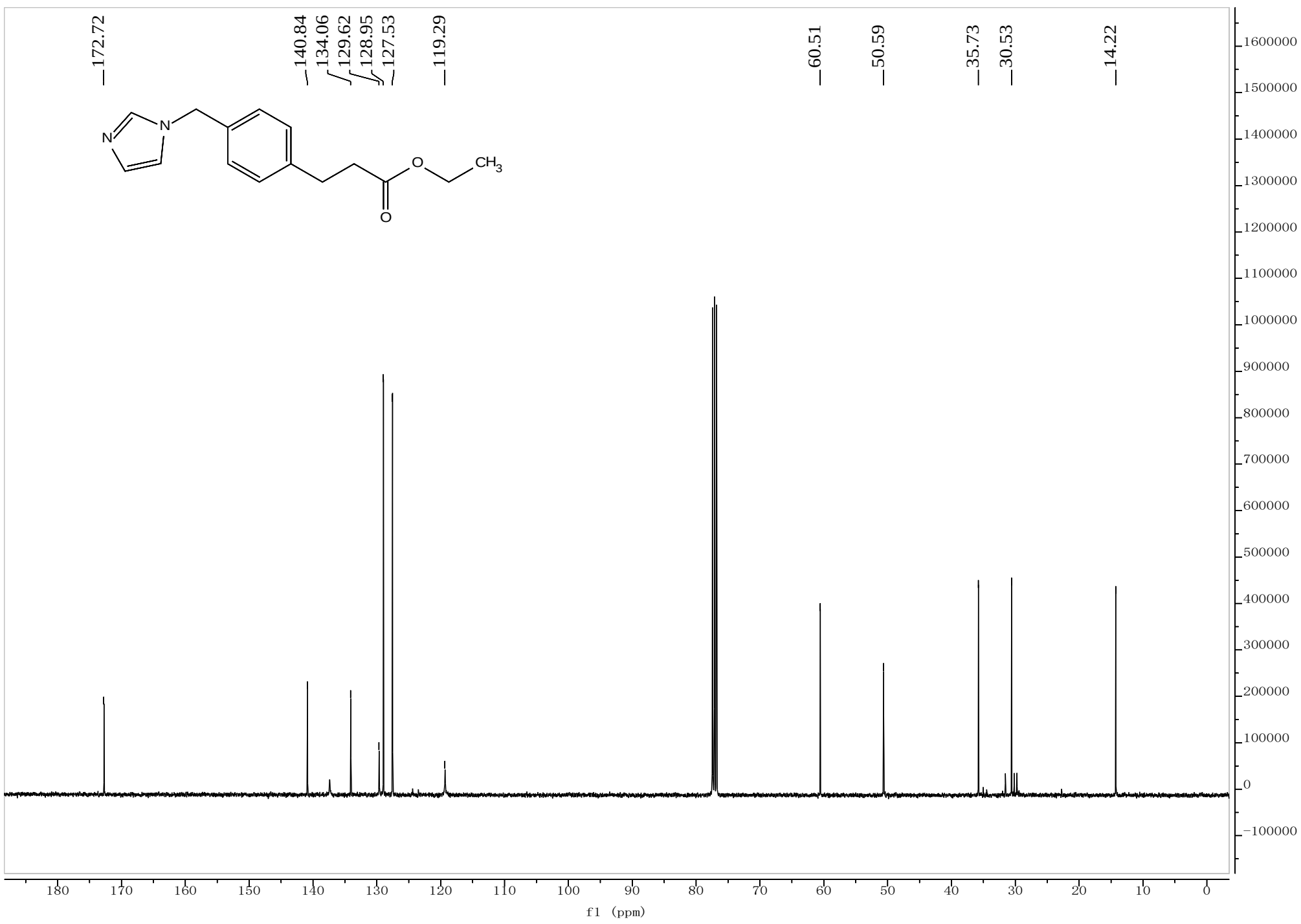

^{13}C NMR (101 MHz, Chloroform-*d*) *δ* 172.72, 140.84, 128.95, 127.53, 60.51, 50.59, 35.73, 30.53, 14.22.

BM-36

^{1}H NMR

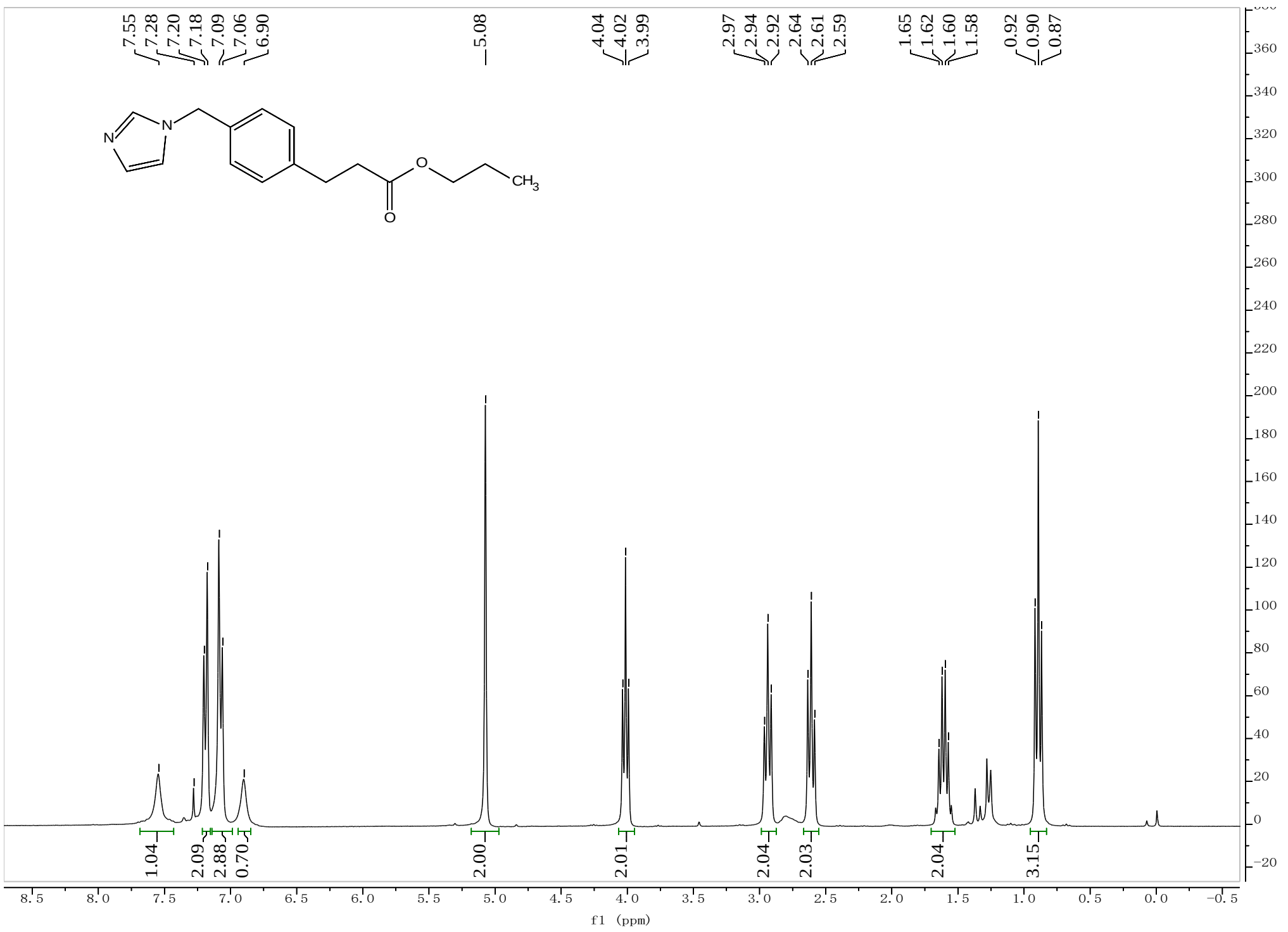

^{1}H NMR (300 MHz, Chloroform-*d*) *δ* 7.55 (s, 1H), 7.19 (d, *J* = 7.8 Hz, 2H), 7.08 (d, *J* = 7.9 Hz, 3H), 6.90 (s, 1H), 5.08 (s, 2H), 4.07-3.95 (m, 2H), 2.94 (t, *J* = 7.7 Hz, 2H), 2.61 (t, *J* = 7.7 Hz, 2H), 1.71-1.53 (m, 2H), 0.90 (t, *J* = 7.4 Hz, 3H).

^{13}C NMR

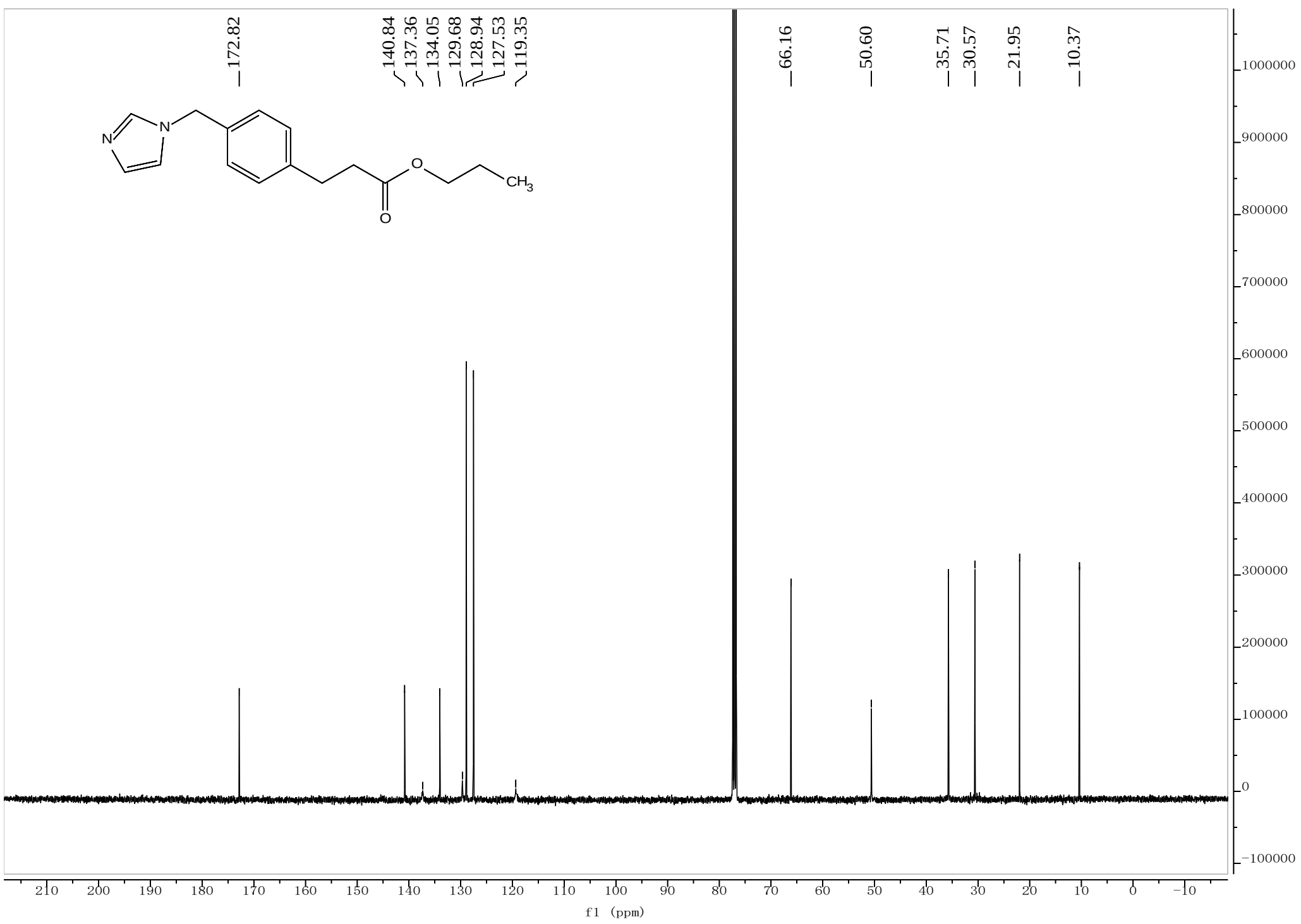

^{13}C NMR (101 MHz, Chloroform-*d*) *δ* 172.82, 140.84, 134.05, 128.94, 127.53, 66.16, 35.71, 30.57, 21.95, 10.37.

BM-37

^{1}H NMR

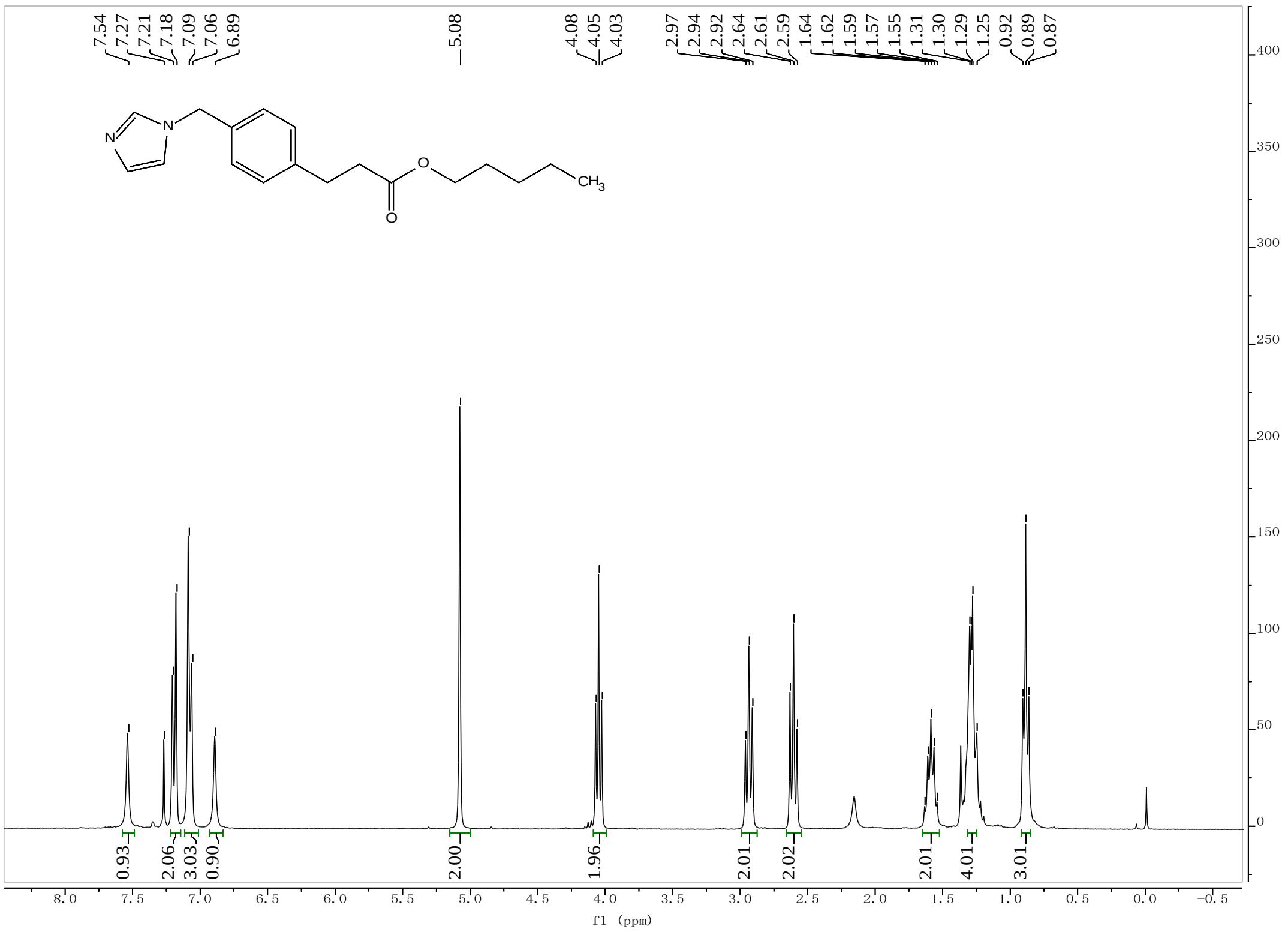

^{1}H NMR (300 MHz, Chloroform-*d*) δ 7.54 (s, 1H), 7.19 (d, J = 8.0 Hz, 2H), 7.08 (d, J = 7.8 Hz, 3H), 6.89 (s, 1H), 5.08 (s, 2H), 4.05 (t, J = 6.7 Hz, 2H), 2.94 (t, J = 7.7 Hz, 2H), 2.61 (t, J = 7.7 Hz, 2H), 1.59 (p, J = 6.8 Hz, 2H), 1.33-1.26 (m,

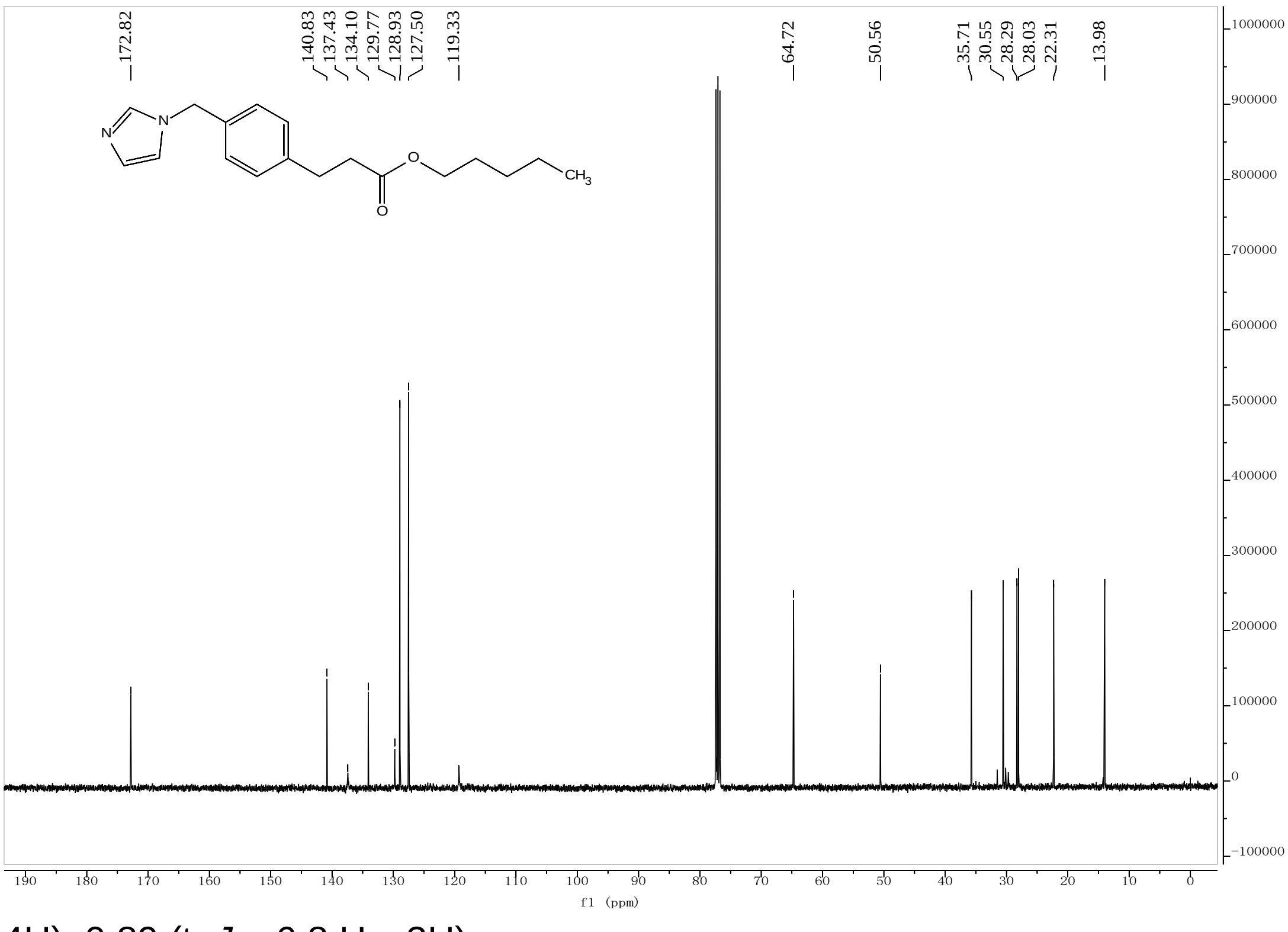

4H), 0.89 (t, J = 6.8 Hz, 3H).

^{13}C NMR (101 MHz, Chloroform-*d*) δ 172.82, 140.83, 134.10, 129.77, 128.93, 127.50, 35.71, 30.55, 28.29, 28.03, 22.31, 13.98.

BM-38

^{1}H NMR

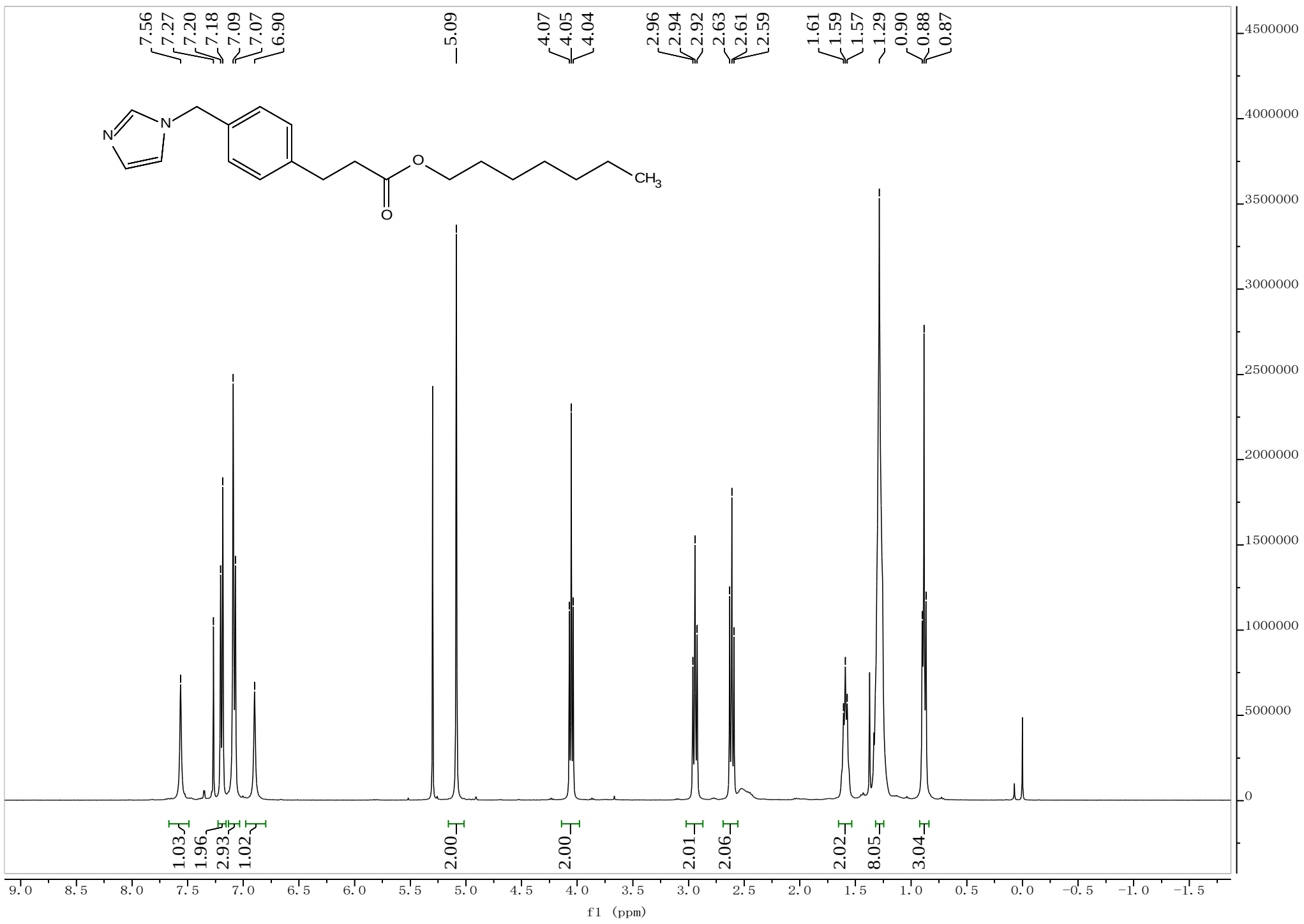

^{1}H NMR (400 MHz, Chloroform-*d*) δ 7.56 (s, 1H), 7.19 (d, *J* = 8.0 Hz, 2H), 7.08 (d, *J* = 8.0 Hz, 3H), 6.90 (s, 1H), 5.09 (s, 2H), 4.05 (t, *J* = 6.8 Hz, 2H), 2.94 (t, *J* = 7.7 Hz, 2H), 2.61 (t, *J* = 7.7 Hz, 2H), 1.65-1.53 (m, 2H), 1.29 (s, 8H), 0.88 (t, *J* = 6.8 Hz, 3H).

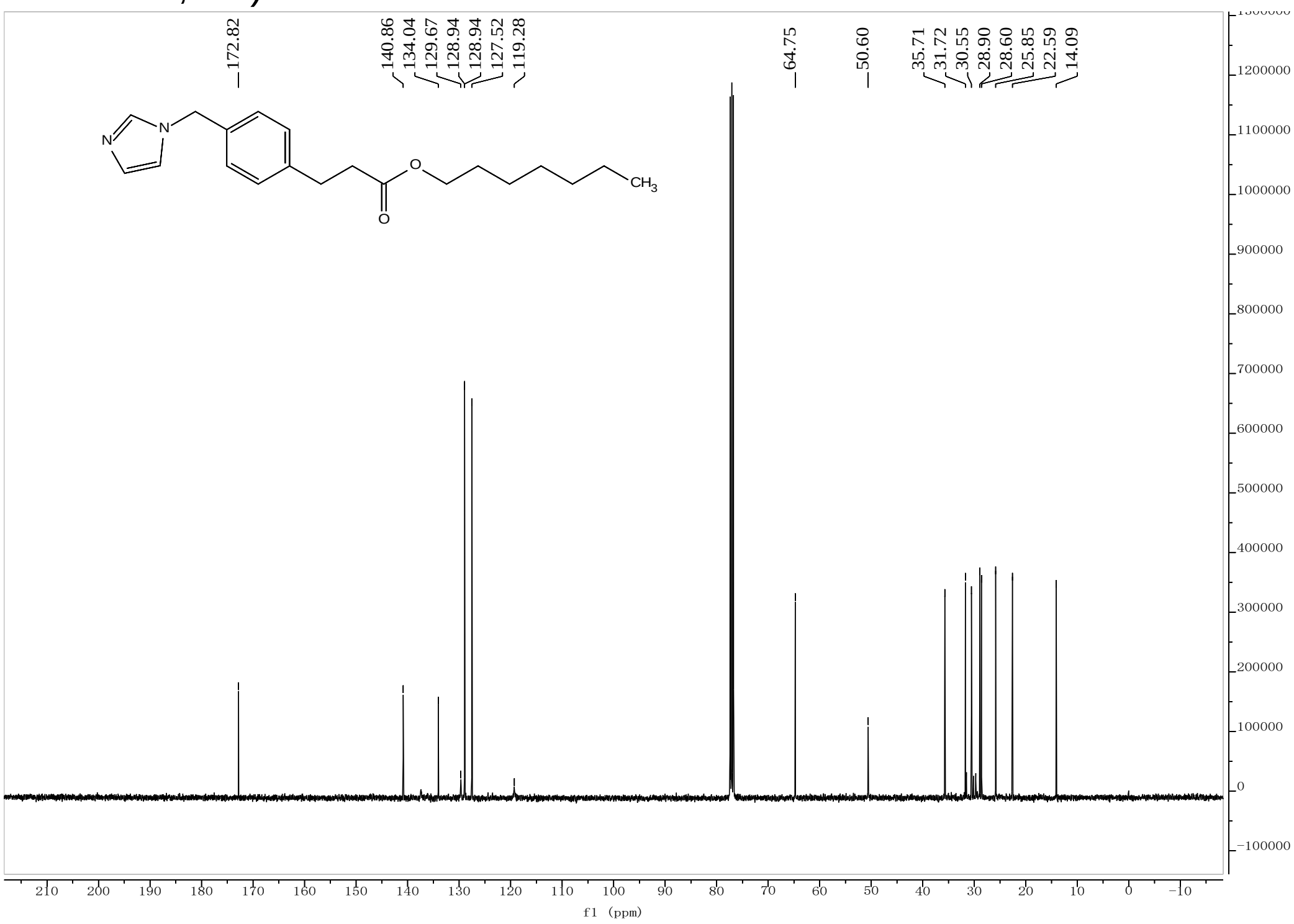

^{13}C NMR

^{13}C NMR (101 MHz, Chloroform-*d*) δ 172.82, 134.04, 128.94, 128.94, 127.52,

64.75, 50.60, 35.71, 30.55, 28.90, 28.60, 25.85, 22.59, 14.09.

BM-39

^{1}H NMR

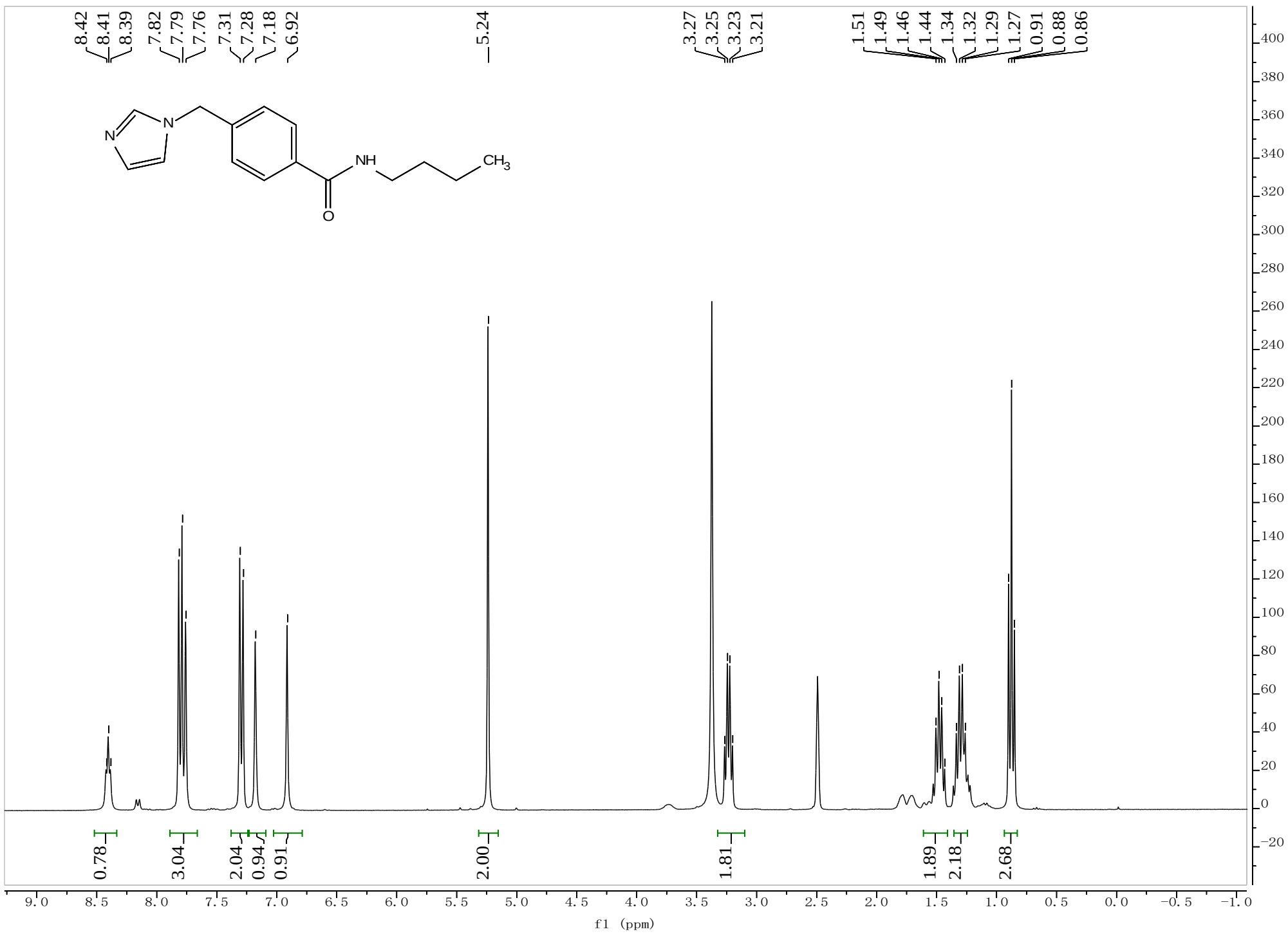

^{1}H NMR (300 MHz, DMSO-d_6) δ 8.53-8.34 (m, 1H), 7.90-7.67 (m, 3H), 7.30 (d, J = 8.1 Hz, 2H), 7.18 (s, 1H), 6.92 (s, 1H), 5.24 (s, 2H), 3.24 (q, J = 6.8 Hz, 2H), 1.47 (q, J = 7.3 Hz, 2H), 1.30 (q, J = 7.3 Hz, 2H), 0.88 (t, J = 7.3 Hz, 3H).

^{13}C NMR

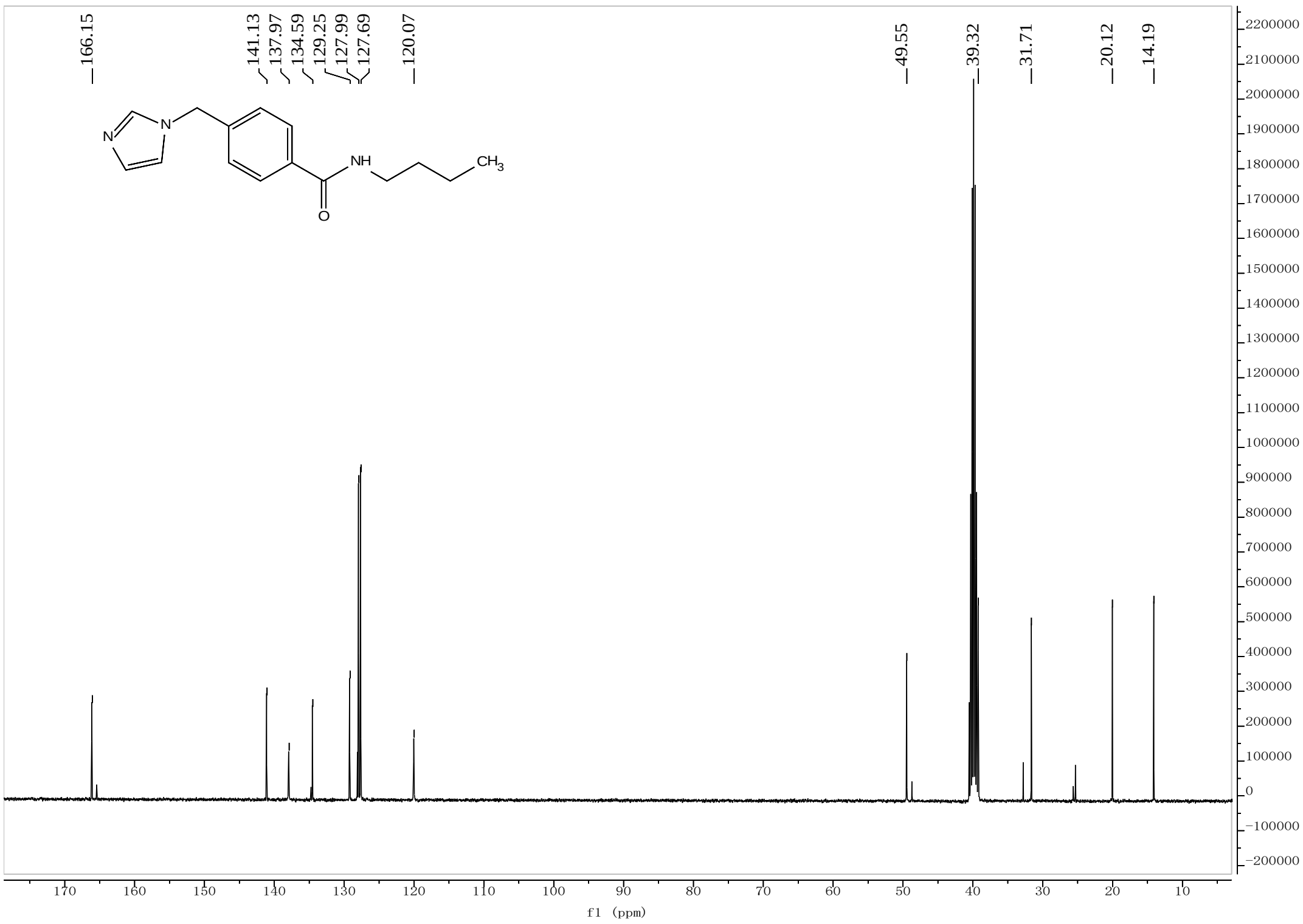

^{13}C NMR (101 MHz, DMSO-d_6) δ 166.15, 141.13, 134.59, 129.25, 127.69, 120.07, 49.55, 39.32, 31.71, 20.12, 14.19.

BM-40

^{1}H NMR

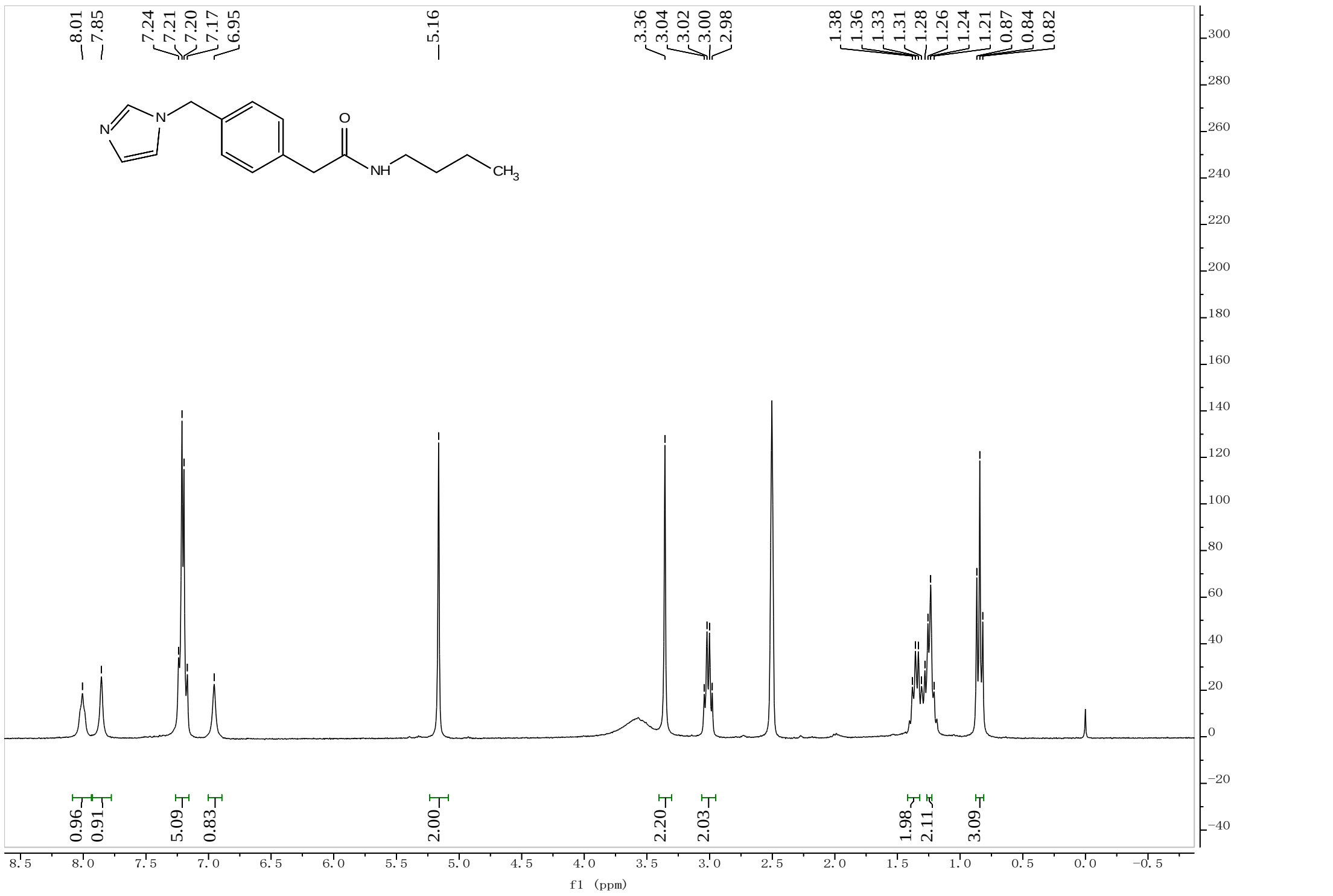

^{1}H NMR (300 MHz, DMSO-d_6) δ 8.01 (s, 1H), 7.85 (s, 1H), 7.22 (t, J = 6.4 Hz, 5H), 6.95 (s, 1H), 5.16 (s, 2H), 3.36 (s, 2H), 3.06-2.95 (m, 2H), 1.42-1.32 (m, 2H), 1.25 (d, J = 6.7 Hz, 2H), 0.84 (t, J = 7.2 Hz, 3H).

^{13}C NMR

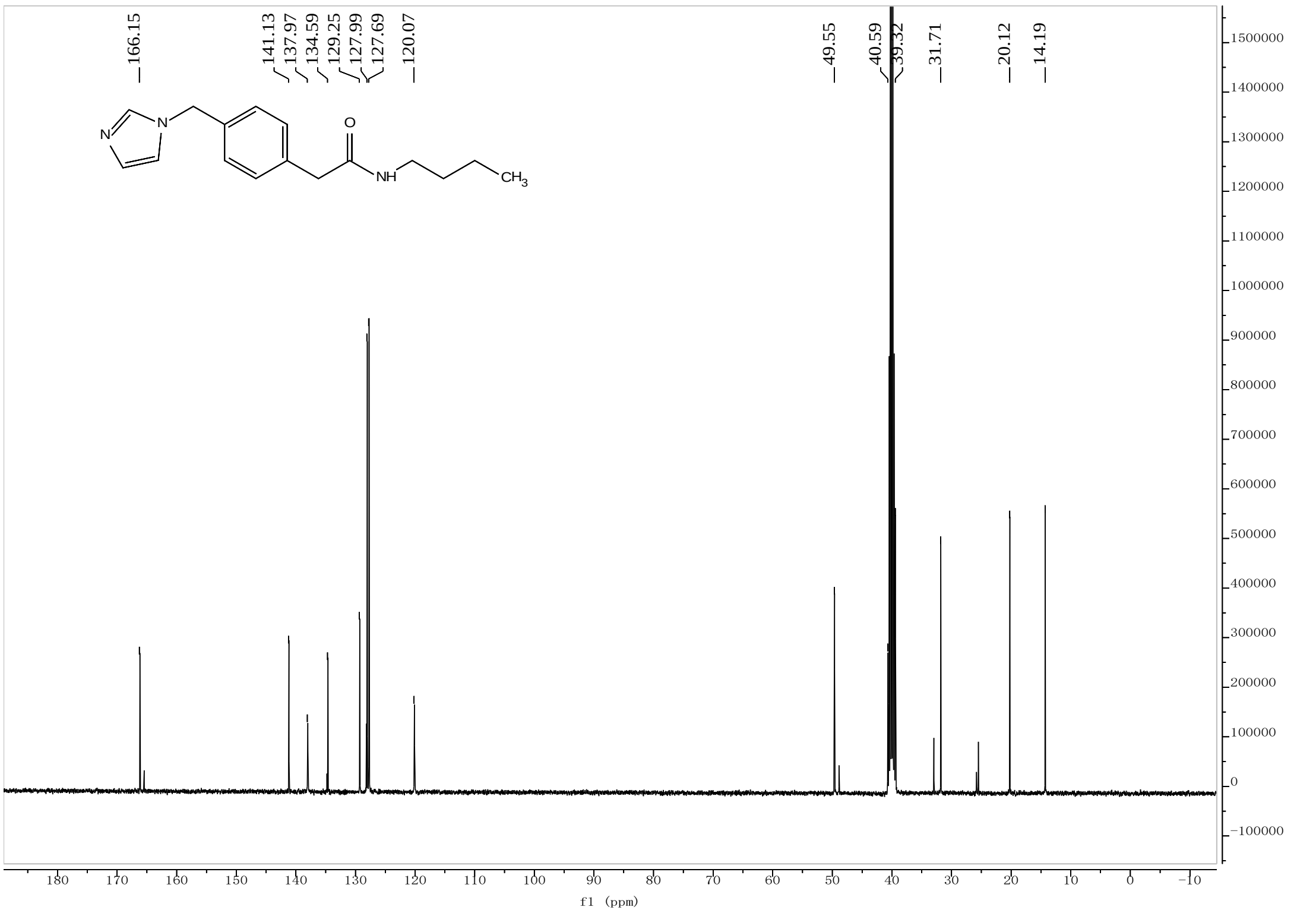

^{13}C NMR (101 MHz, DMSO-d_6) δ 166.15, 141.13, 134.59, 129.25, 127.69,

120.07, 49.55, 40.59, 39.32, 31.71, 20.12, 14.19.

BM-41

^{1}H NMR

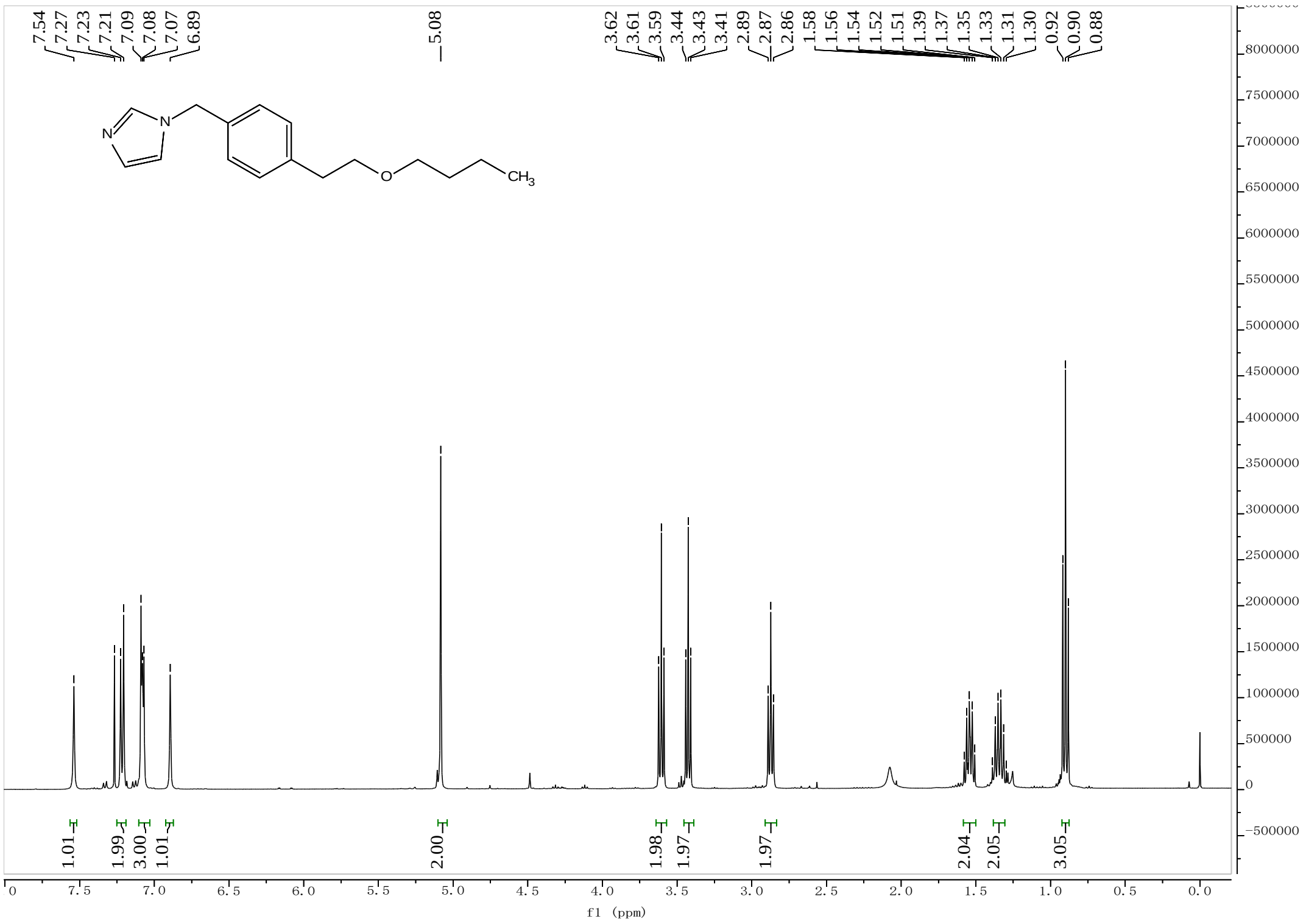

^{1}H NMR (400 MHz, Chloroform-*d*) *δ* 7.54 (s, 1H), 7.22 (d, *J* = 8.1 Hz, 2H), 7.10-7.03 (m, 3H), 6.89 (s, 1H), 5.08 (s, 2H), 3.61 (t, *J* = 7.1 Hz, 2H), 3.43 (t, *J* = 6.6 Hz, 2H), 2.87 (t, *J* = 7.1 Hz, 2H), 1.58-1.50 (m, 2H), 1.38-1.31 (m, 2H), 0.90 (t,

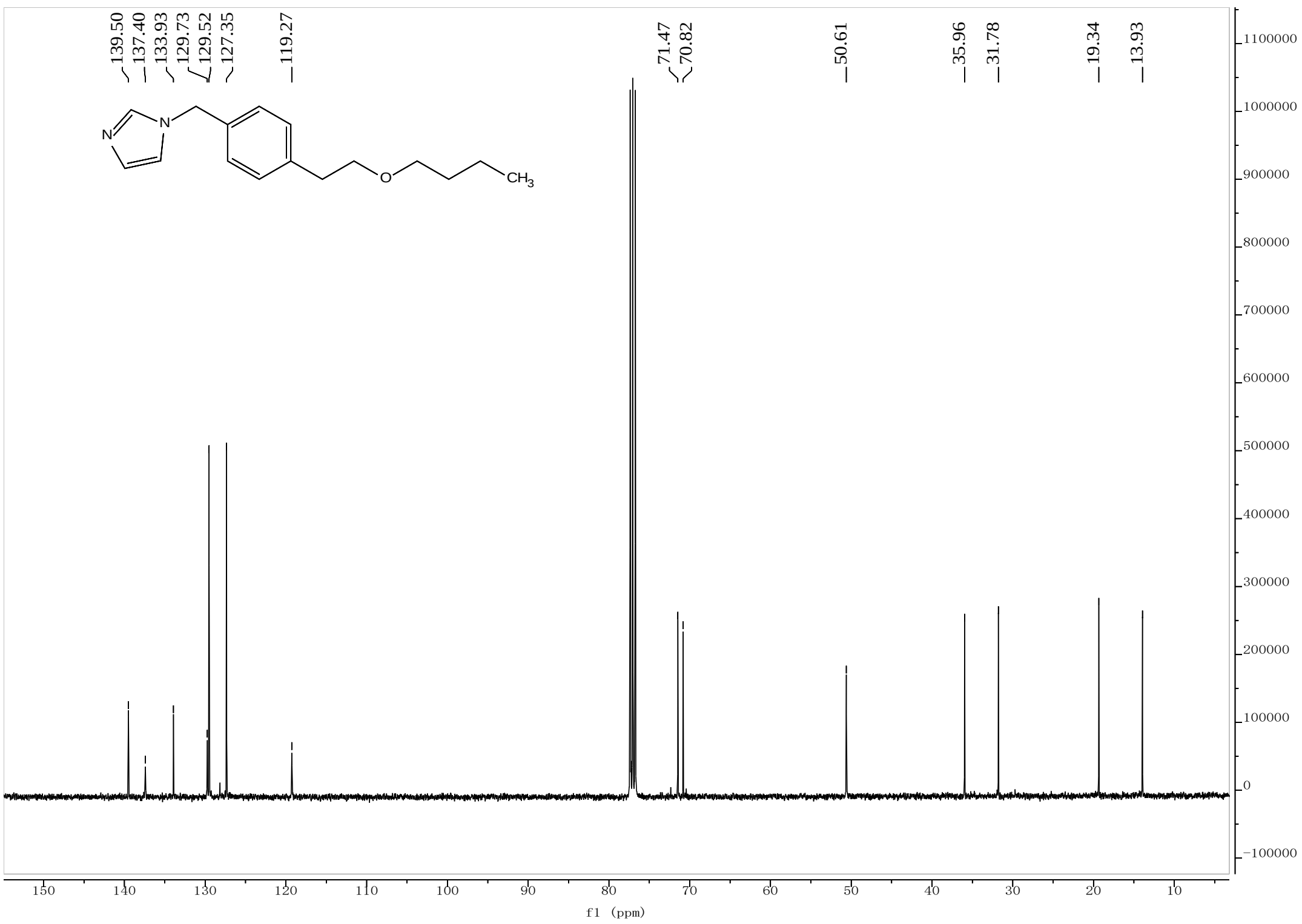

J = 7.4 Hz, 3H).

^{13}C NMR (101 MHz, Chloroform-*d*) δ 139.50, 137.40, 133.93, 129.73, 129.52, 127.35, 71.47, 50.61, 35.96, 31.78, 19.34, 13.93.

BM-42

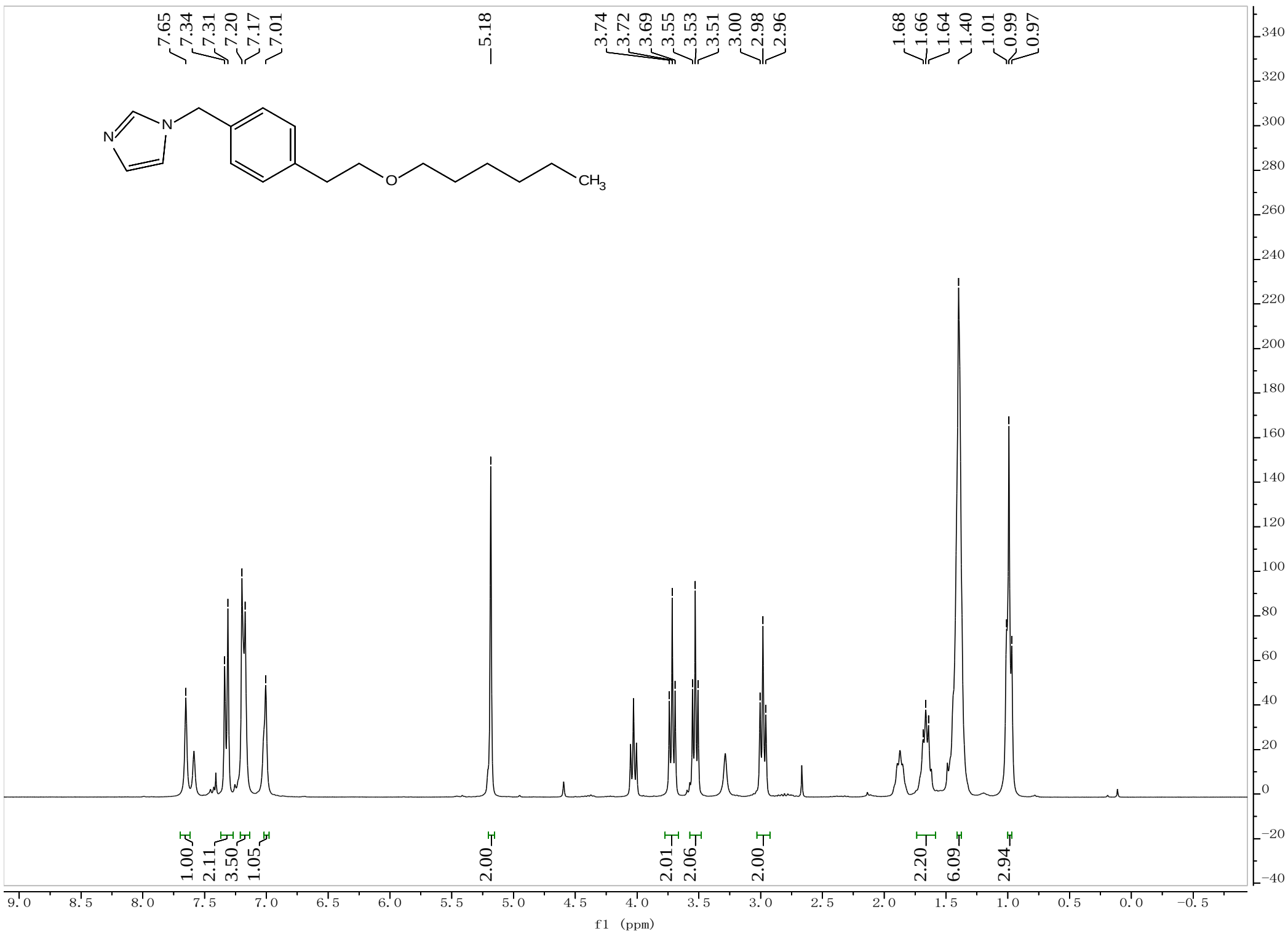

1H NMR (300 MHz, Chloroform-d) δ 7.65 (s, 1H), 7.32 (d, *J* = 7.9 Hz, 2H), 7.18 (d, *J* = 7.8 Hz, 3H), 7.01 (s, 1H), 5.18 (s, 2H), 3.72 (t, *J* = 7.1 Hz, 2H), 3.53 (t, *J* = 6.7 Hz, 2H), 3.03-2.93 (m, 2H), 1.74-1.59 (m, 2H), 1.40 (s, 6H), 0.99 (s, 3H).

^{13}C NMR

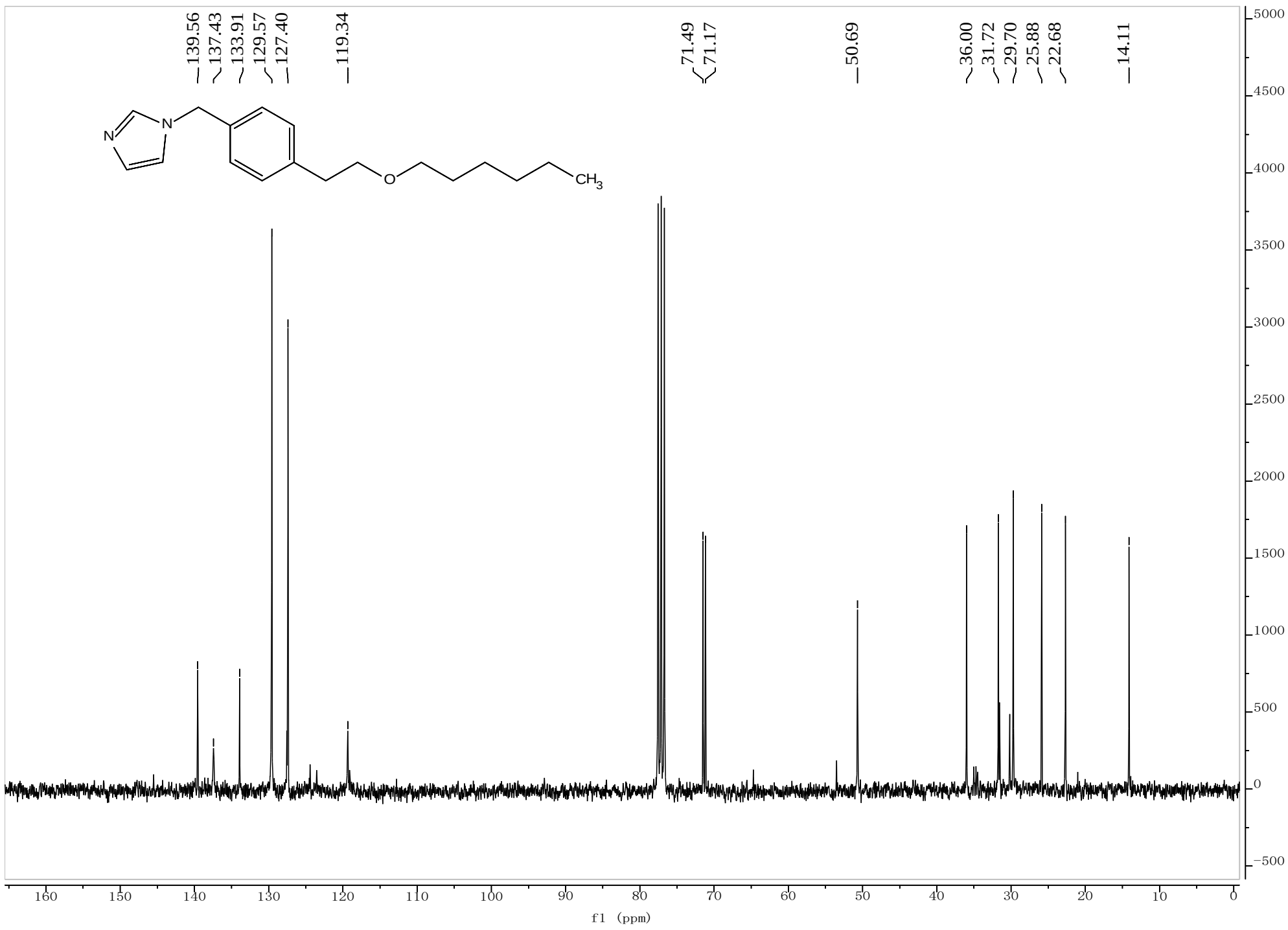

^{13}C NMR (101 MHz, Chloroform-*d*) *δ* 139.56, 137.43, 133.91, 129.57, 127.40, 119.34, 71.49, 71.17, 50.69, 36.00, 31.72, 29.70, 25.88, 22.68, 14.11.

BM-43

^{1}H NMR

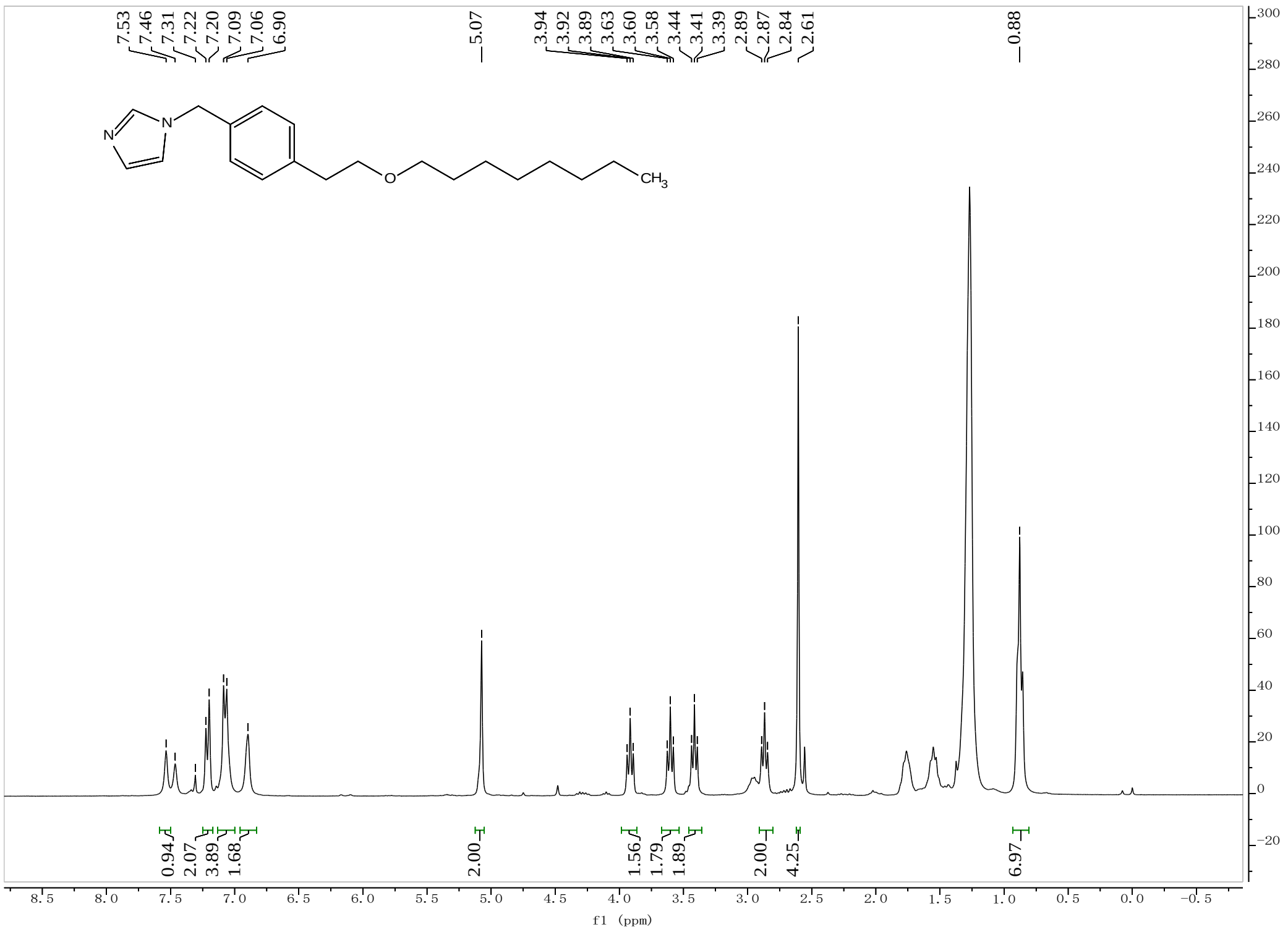

^{1}H NMR (300 MHz, Chloroform-*d*) δ 7.53 (s, 1H), 7.21 (d, J = 7.8 Hz, 2H), 7.07 (d, J = 7.4 Hz, 4H), 6.90 (s, 2H), 5.07 (s, 2H), 3.92 (t, J = 7.1 Hz, 2H), 3.67-3.53 (m, 2H), 3.46-3.36 (m, 2H), 2.91-2.80 (m, 2H), 2.61 (s, 4H), 0.88 (s, 7H).

^{13}C NMR

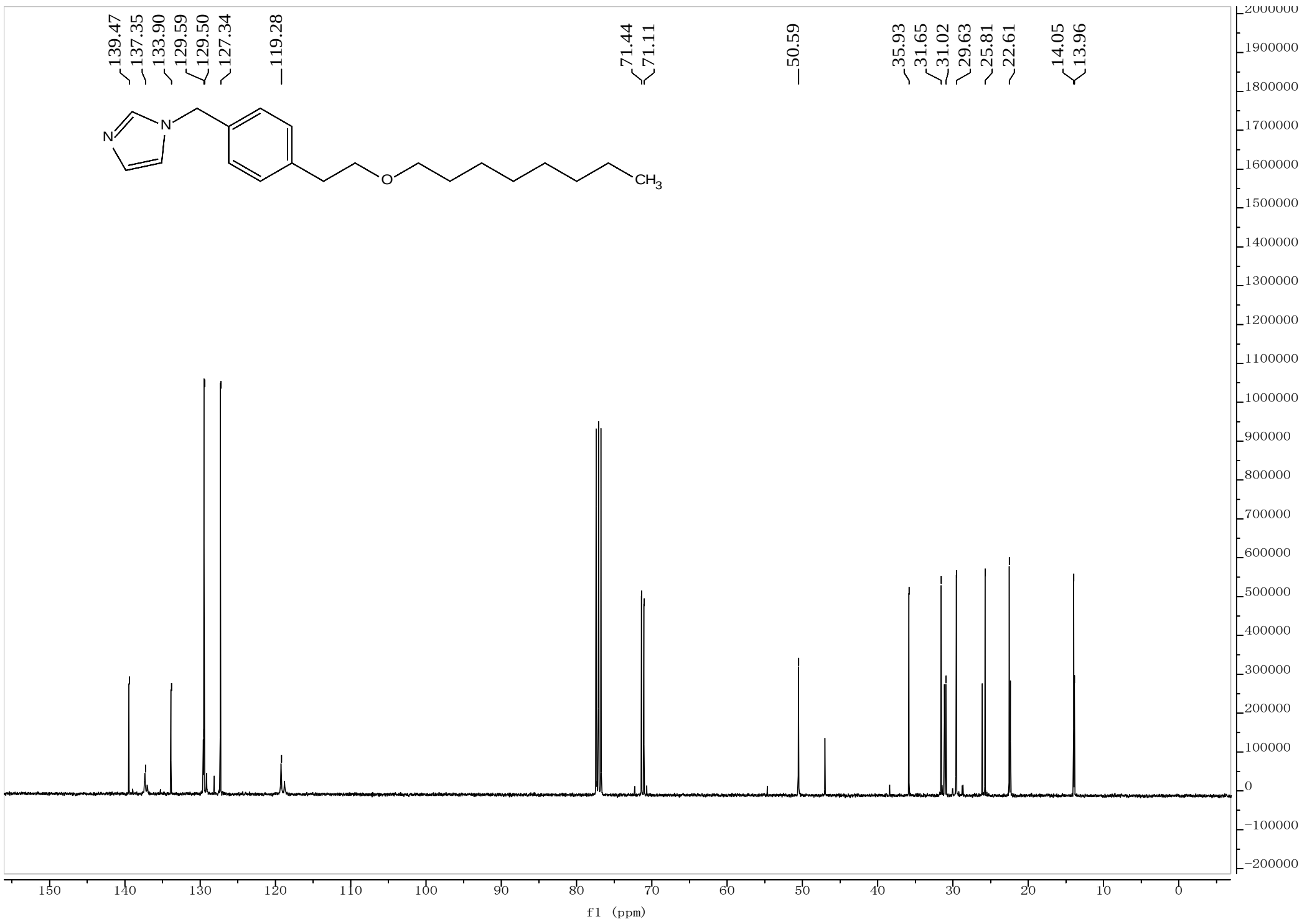

^{13}C NMR (101 MHz, Chloroform-*d*) δ 139.47, 133.90, 129.59, 129.50, 127.34, 71.44, 71.11, 35.93, 31.65, 31.02, 29.63, 25.81, 22.61, 14.05, 13.96.

BM-44

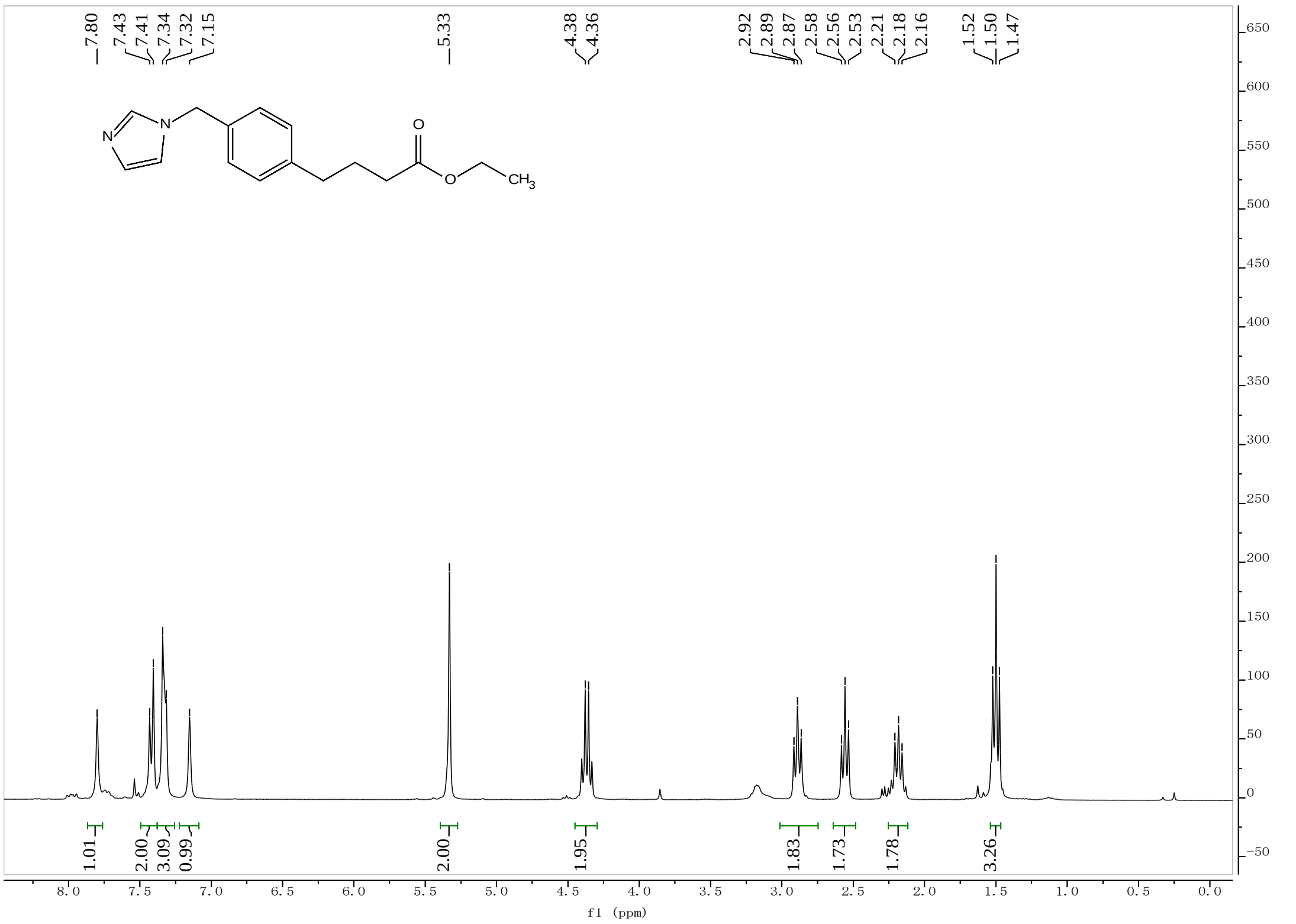

^{1}H NMR (300 MHz, Chloroform-*d*) δ 7.80 (s, 1H), 7.42 (d, J = 7.9 Hz, 2H), 7.33 (d, J = 7.5 Hz, 3H), 7.15 (s, 1H), 5.33 (s, 2H), 4.37 (d, J = 7.1 Hz, 2H), 3.01-2.75 (m, 2H), 2.56 (t, J = 7.4 Hz, 2H), 2.25-2.12 (m, 2H), 1.50 (t, J = 7.1 Hz, 3H).

^{13}C NMR

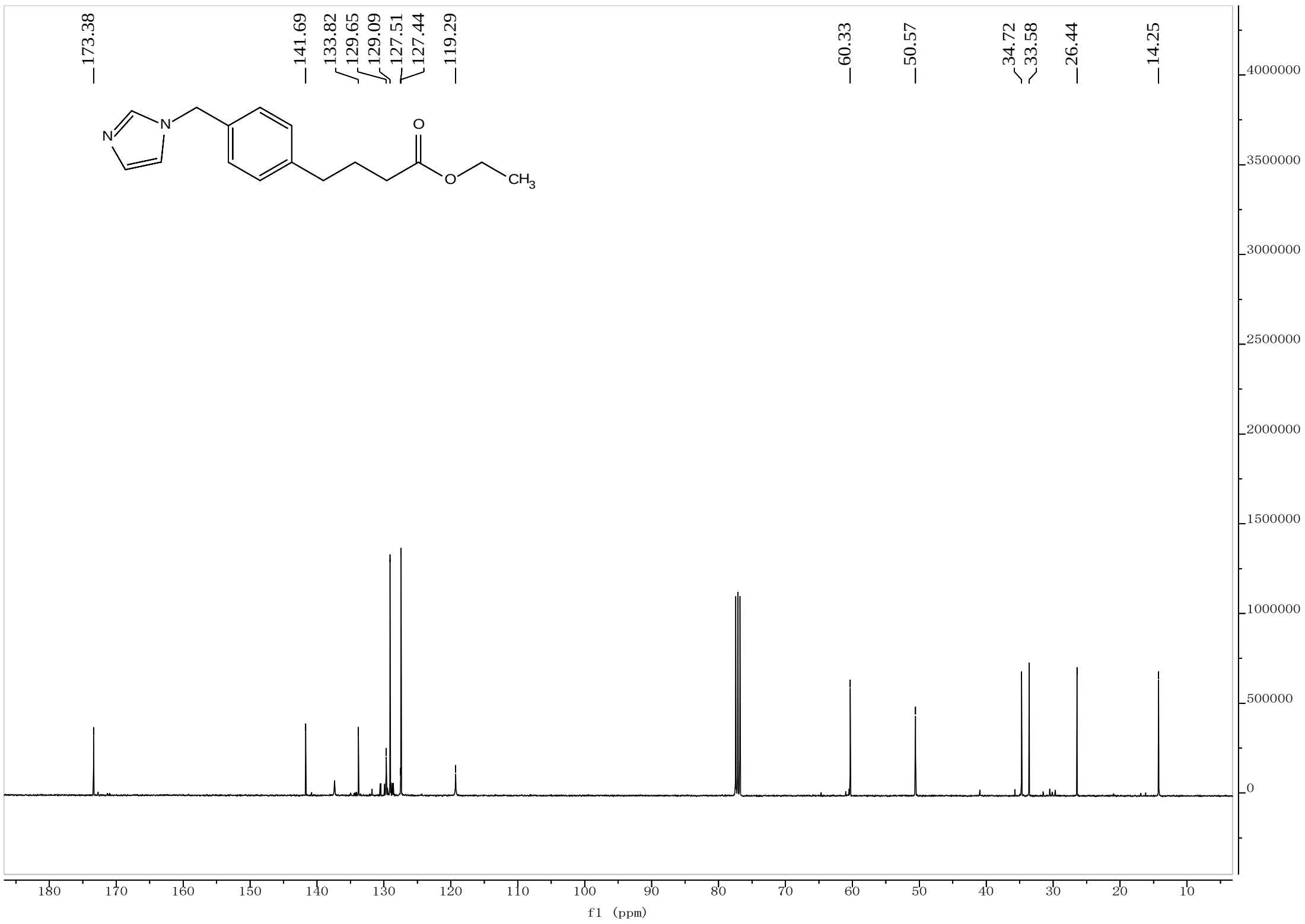

^{13}C NMR (101 MHz, Chloroform-*d*) δ 173.38, 141.69, 133.82, 129.65, 129.09,

127.44, 60.33, 50.57, 34.72, 33.58, 26.44, 14.25.

BM-45

^{1}H NMR

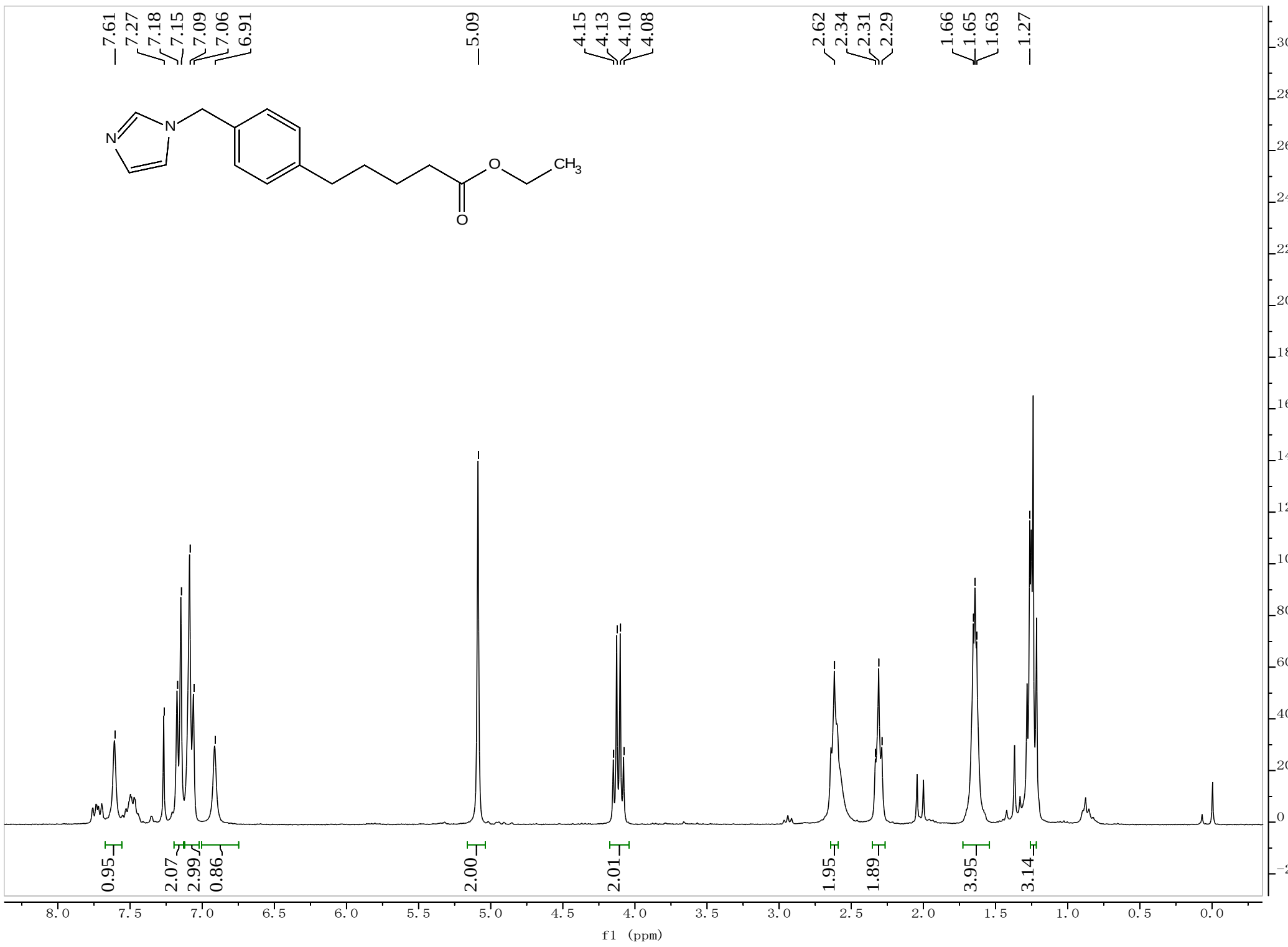

^{1}H NMR (300 MHz, Chloroform-*d*) δ 7.61 (s, 1H), 7.16 (d, J = 8.0 Hz, 2H), 7.07 (d, J = 8.0 Hz, 3H), 6.91 (s, 1H), 5.09 (s, 2H), 4.12 (q, J = 7.1 Hz, 2H), 2.62 (s, 2H), 2.31 (t, J = 6.7 Hz, 2H), 1.73-1.55 (m, 4H), 1.25 (d, J = 4.3 Hz, 3H).

^{13}C NMR

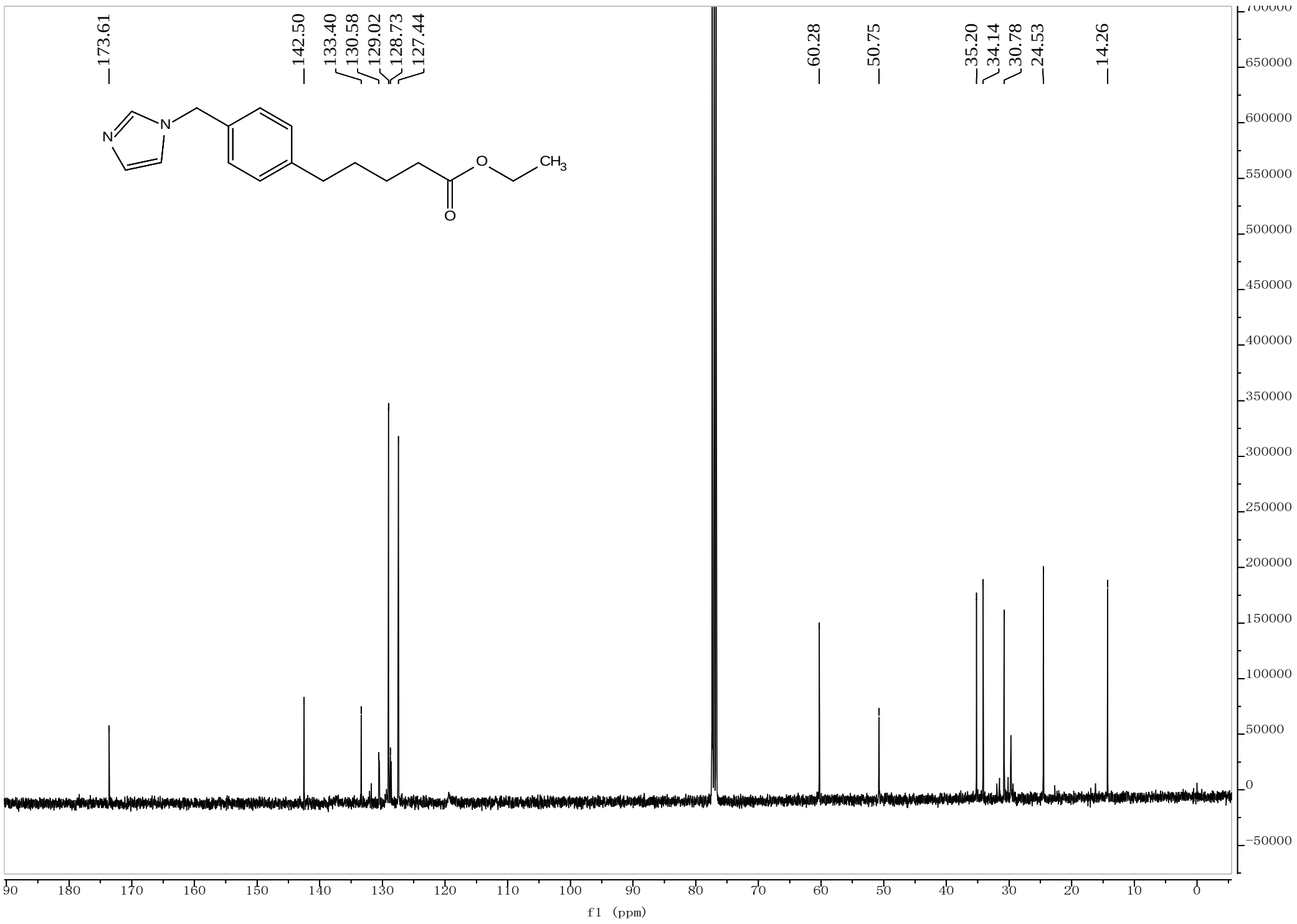

^{13}C NMR (101 MHz, Chloroform-*d*) δ 173.61, 142.50, 129.02, 60.28, 35.20, 34.14, 30.78, 24.53, 14.26.

BM-46

^{1}H NMR

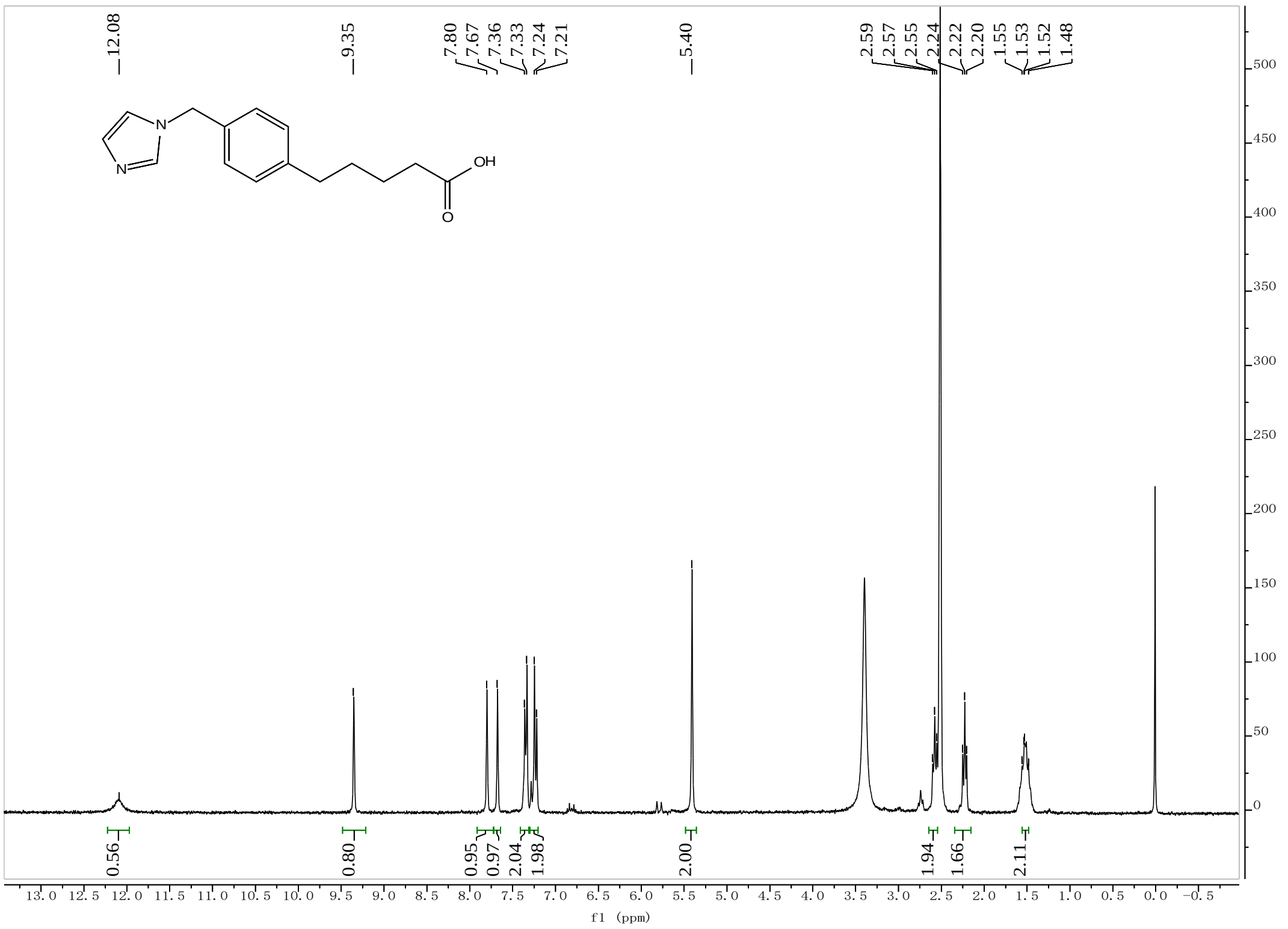

^{1}H NMR (300 MHz, DMSO-d_6) δ 12.08 (s, 1H), 9.35 (s, 1H), 7.80 (s, 1H), 7.67 (s, 1H), 7.40-7.30 (m, 2H), 7.29-7.20 (m, 2H), 5.40 (s, 2H), 2.57 (t, J = 7.3 Hz, 2H), 2.22 (t, J = 6.9 Hz, 2H), 1.55-1.47 (m, 2H).

^{13}C NMR

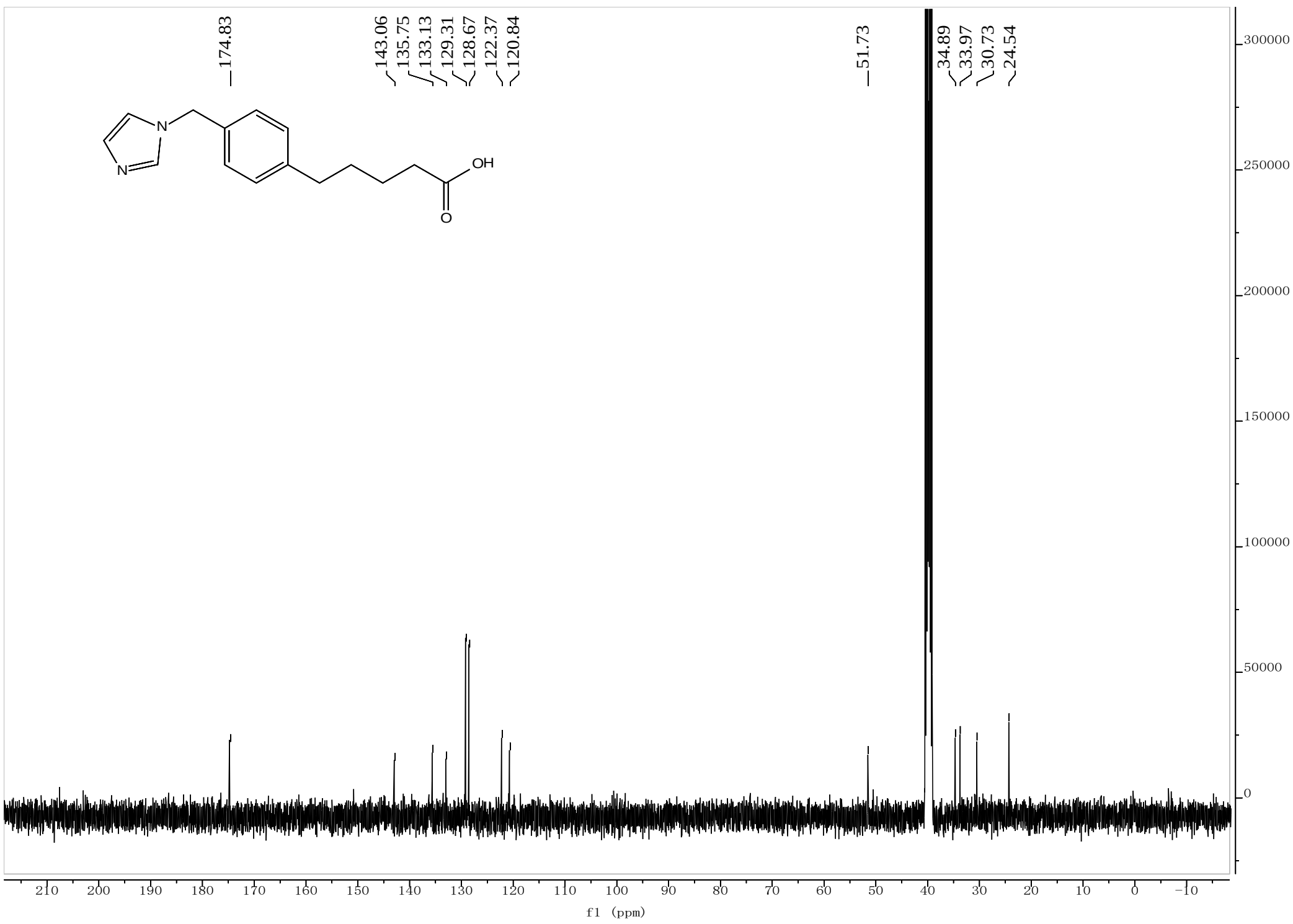

^{13}C NMR (101 MHz, DMSO-d_6) δ 174.83, 143.06, 135.75, 133.13, 129.31, 128.67, 122.37, 120.84, 51.73, 34.89, 33.97.

BM-47

^{1}H NMR

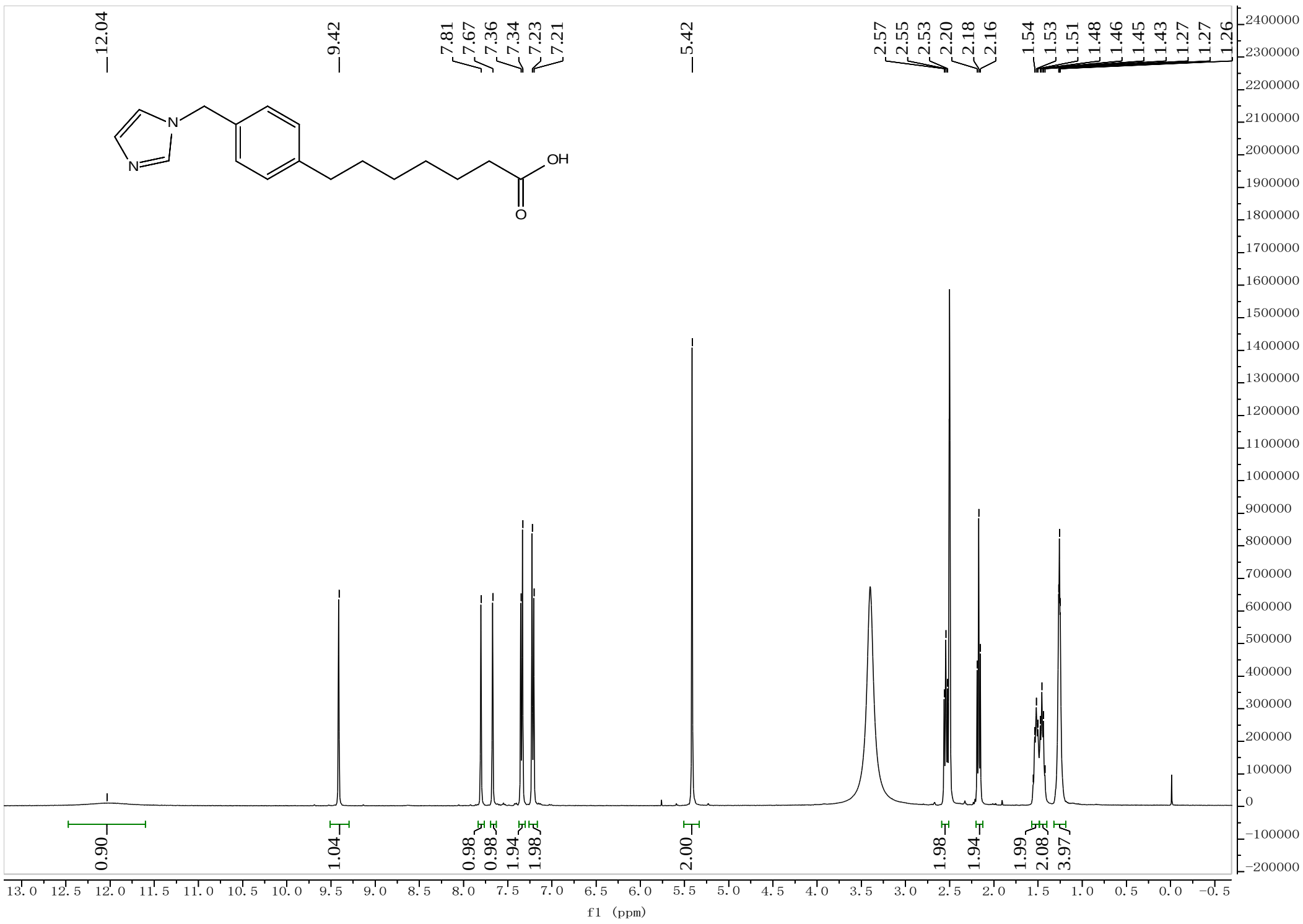

^{1}H NMR (400 MHz, DMSO-d_6) δ 12.04 (s, 1H), 9.42 (s, 1H), 7.81 (s, 1H), 7.67 (s, 1H), 7.35 (d, J = 8.0 Hz, 2H), 7.22 (d, J = 8.0 Hz, 2H), 5.42 (s, 2H), 2.60-2.52 (m, 2H), 2.18 (t, J = 7.3 Hz, 2H), 1.54 (q, J = 7.1 Hz, 2H), 1.46 (q, J = 7.1 Hz, 2H), 1.33-1.20 (m, 4H).

^{13}C NMR

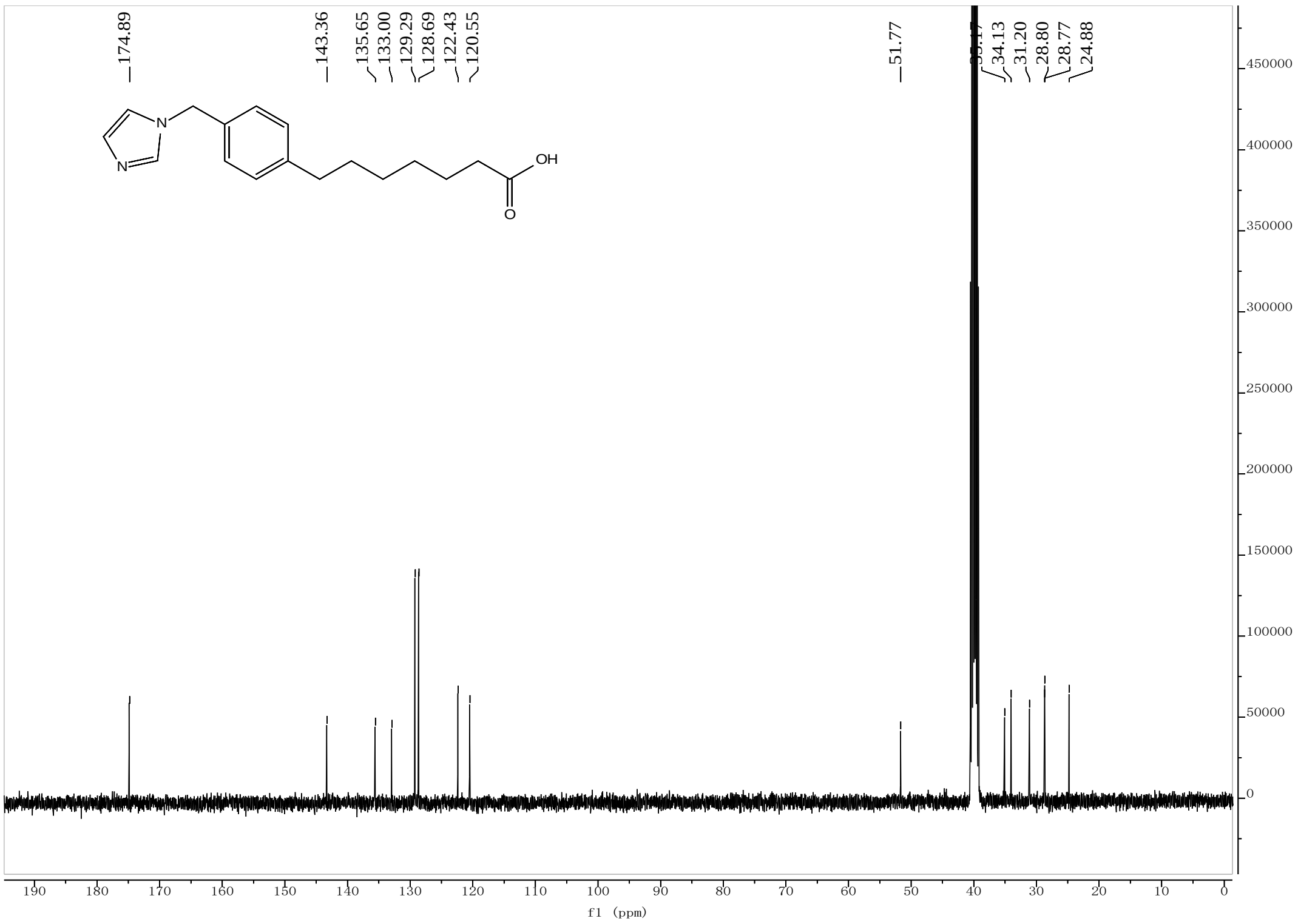

^{13}C NMR (101 MHz, DMSO-d_6) δ 174.89, 143.36, 135.65, 133.00, 129.29,

128.69, 122.43, 120.55, 51.77, 35.17, 34.13, 31.20, 28.80, 28.77, 24.88.

BM-48

^{1}H NMR

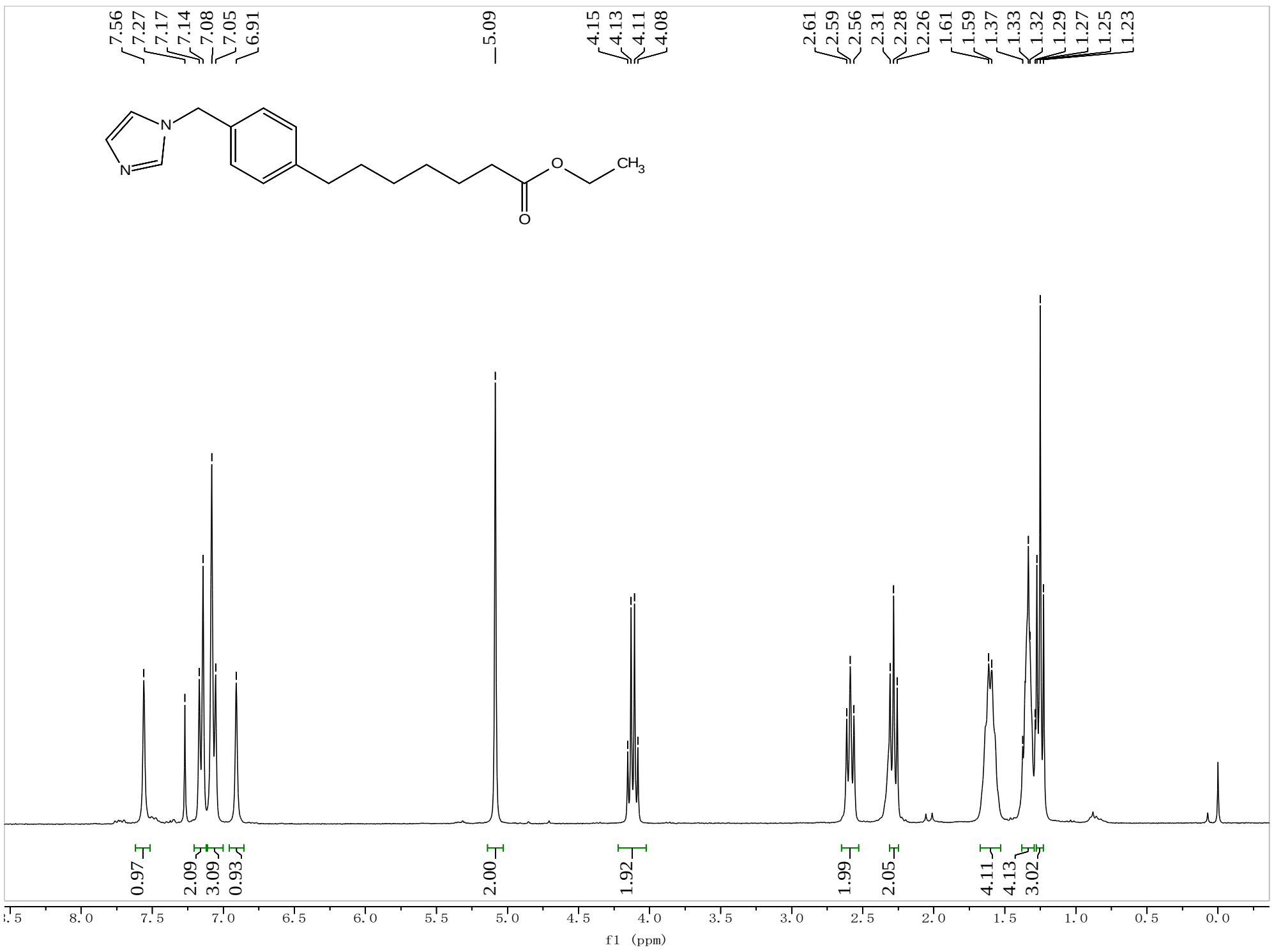

^{1}H NMR (300 MHz, Chloroform-*d*) δ 7.56 (s, 1H), 7.16 (d, J = 8.0 Hz, 2H), 7.07 (d, J = 8.2 Hz, 3H), 6.91 (s, 1H), 5.09 (s, 2H), 4.12 (q, J = 7.1 Hz, 2H), 2.65-2.53 (m, 2H), 2.28 (t, J = 7.5 Hz, 2H), 1.60 (d, J = 6.5 Hz, 4H), 1.38-1.29 (m, 4H), 1.28-1.23 (m, 3H).

^{13}C NMR

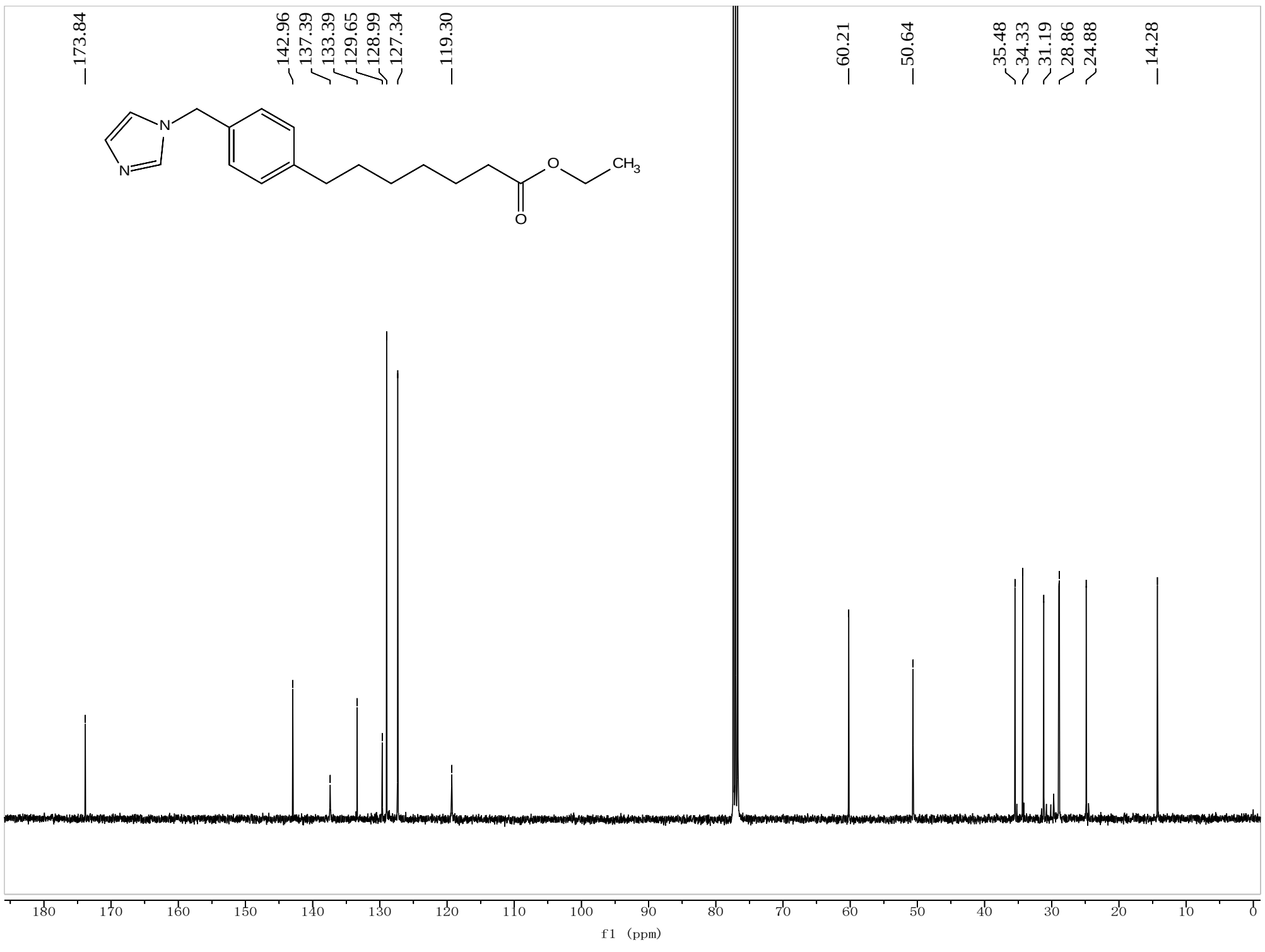

^{13}C NMR (101 MHz, Chloroform-*d*) δ 173.84, 142.96, 133.39, 129.65, 128.99,

127.34, 60.21, 35.48, 34.33, 31.19, 28.86, 24.88, 14.28.

BM-49

^{1}H NMR

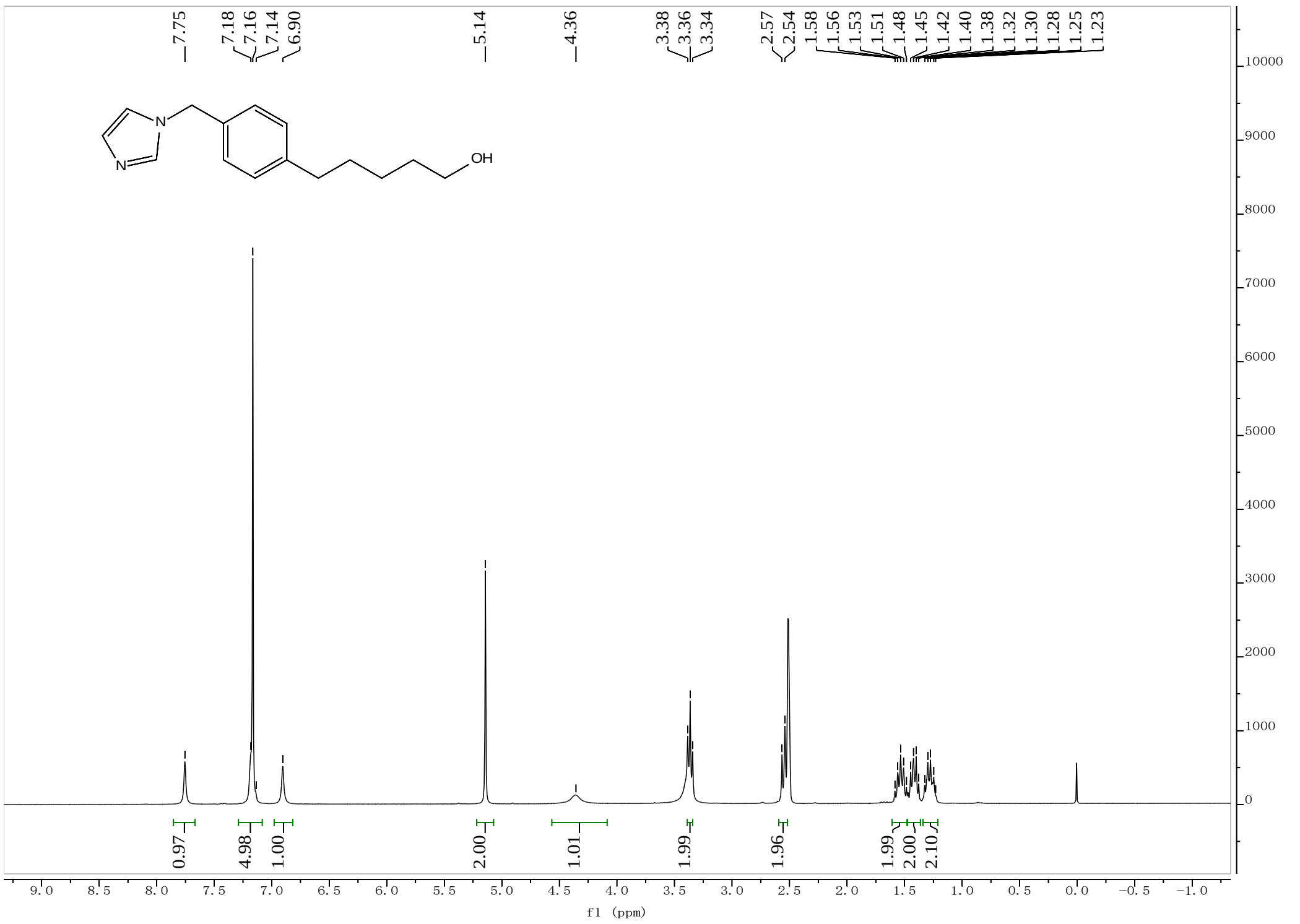

^{1}H NMR (300 MHz, DMSO-d_6) δ 7.75 (s, 1H), 7.16 (s, 5H), 6.90 (s, 1H), 5.14 (s, 2H), 4.36 (s, 1H), 3.37 (d, J = 6.4 Hz, 2H), 2.55 (d, J = 7.5 Hz, 2H), 1.53 (p, J = 7.5 Hz, 2H), 1.41 (q, J = 6.8, 6.3 Hz, 2H), 1.27 (dt, J = 13.8, 6.3 Hz, 2H).

^{13}C NMR

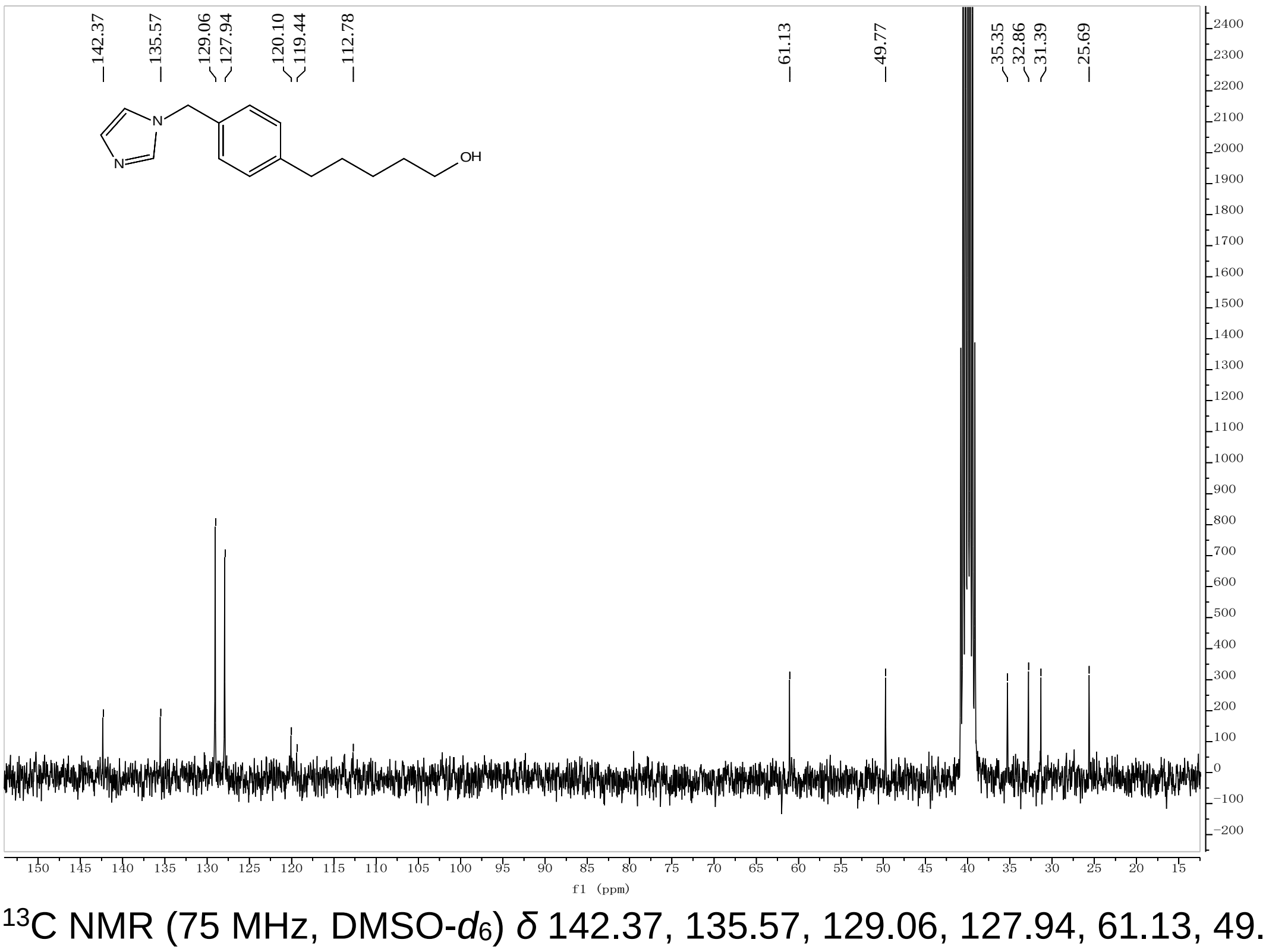

^{13}C NMR (75 MHz, DMSO-d_6) δ 142.37, 135.57, 129.06, 127.94, 61.13, 49.77, 35.35, 32.86, 31.39, 25.69.

BM-50

^{1}H NMR

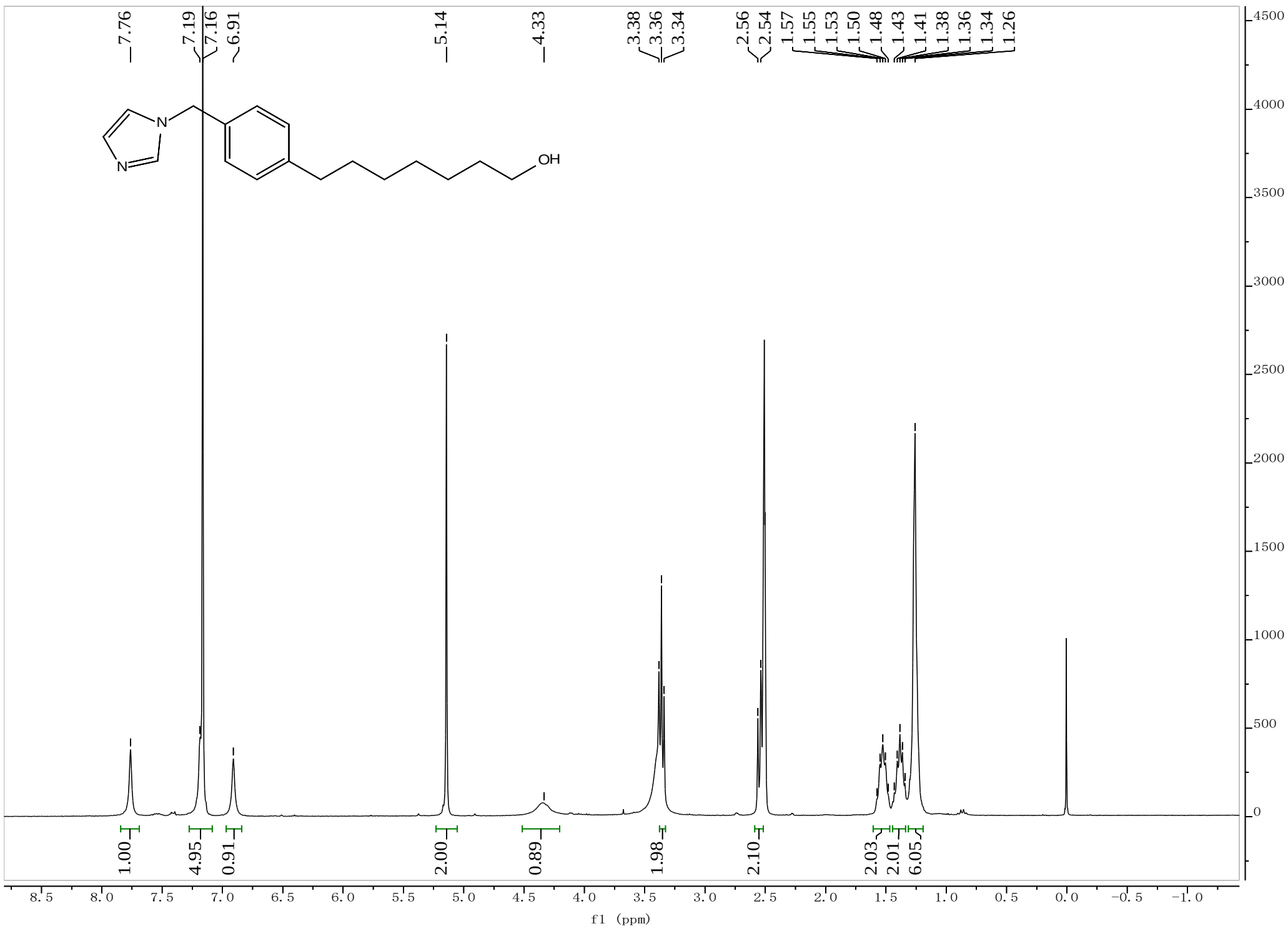

^{1}H NMR (300 MHz, DMSO-d_6) δ 7.76 (s, 1H), 7.16 (s, 5H), 6.91 (s, 1H), 5.14 (s, 2H), 4.33 (s, 1H), 3.35 (d, J = 6.5 Hz, 2H), 2.55 (d, J = 7.4 Hz, 2H), 1.53 (p, J = 7.0, 6.6 Hz, 2H), 1.39 (p, J = 6.9, 6.3 Hz, 2H), 1.26 (s, 6H).

^{13}C NMR

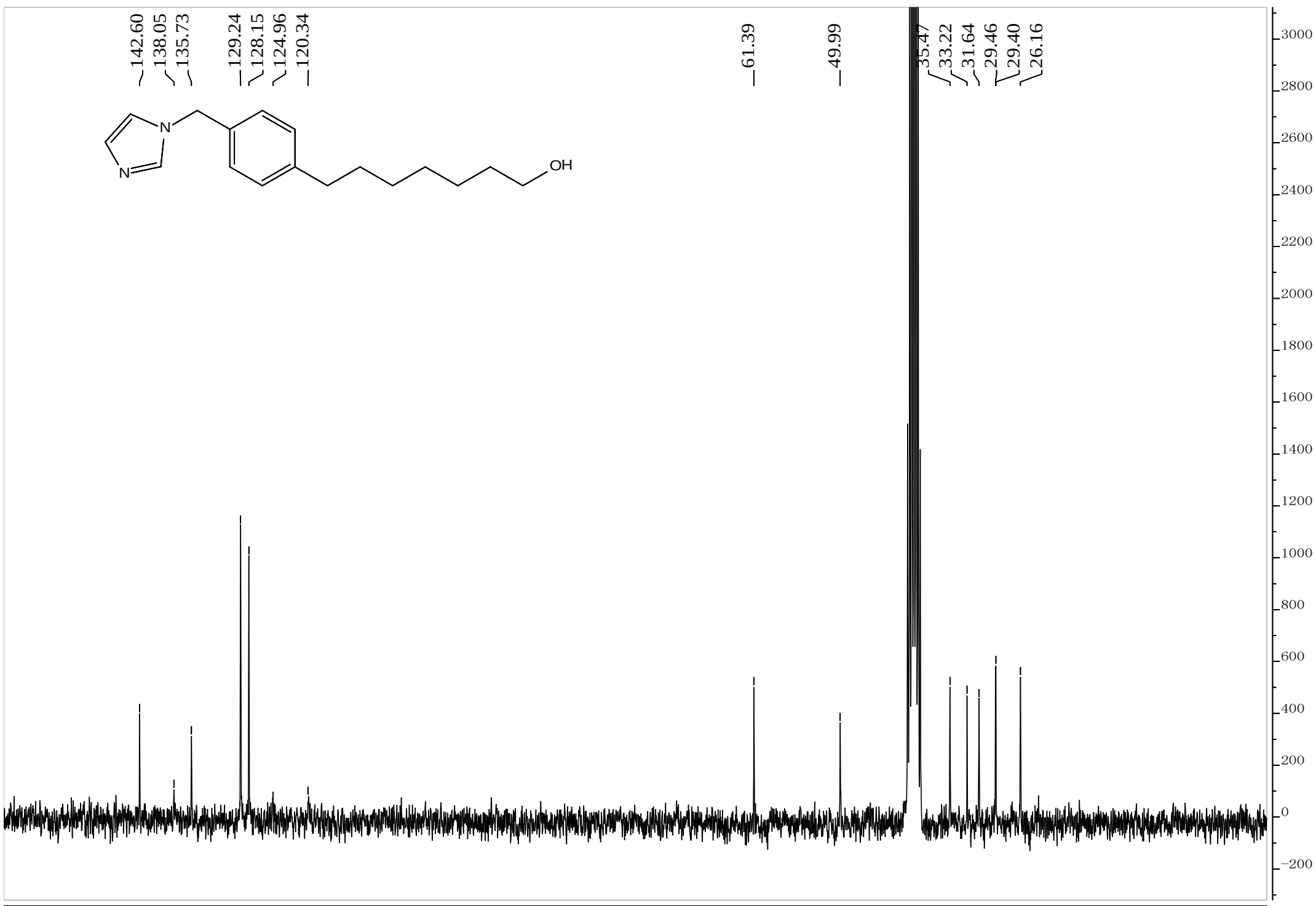

^{13}C NMR (75 MHz, DMSO-d_6) δ 142.60, 135.73, 129.24, 128.15, 61.39, 49.99,

35.47, 33.22, 31.64, 29.46, 29.40, 26.16.

BM-51

^{1}H NMR

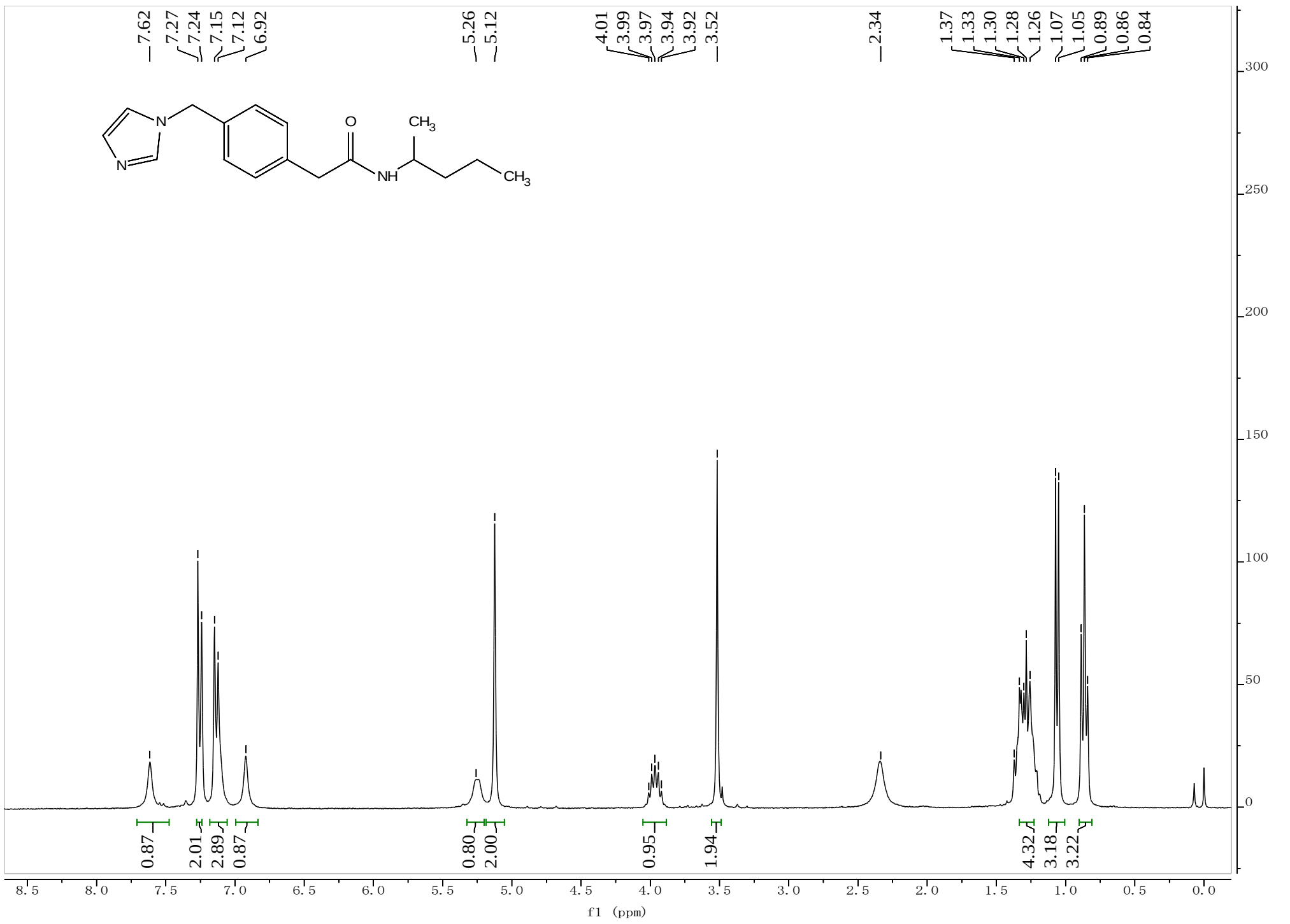

^{1}H NMR (300 MHz, Chloroform-*d*) δ 7.62 (s, 1H), 7.26 (d, J = 8.3 Hz, 2H), 7.14 (m, 3H), 6.92 (s, 1H), 5.26 (S, 1H), 5.12 (s, 2H), 3.97 (dt, J = 14.6, 6.4 Hz, 1H), 3.52 (s, 2H), 1.29 (dd, J = 13.9, 8.8 Hz, 4H), 1.06 (d, J = 6.6 Hz, 3H), 0.86 (t, J = 7.0 Hz, 3H).

^{13}C NMR

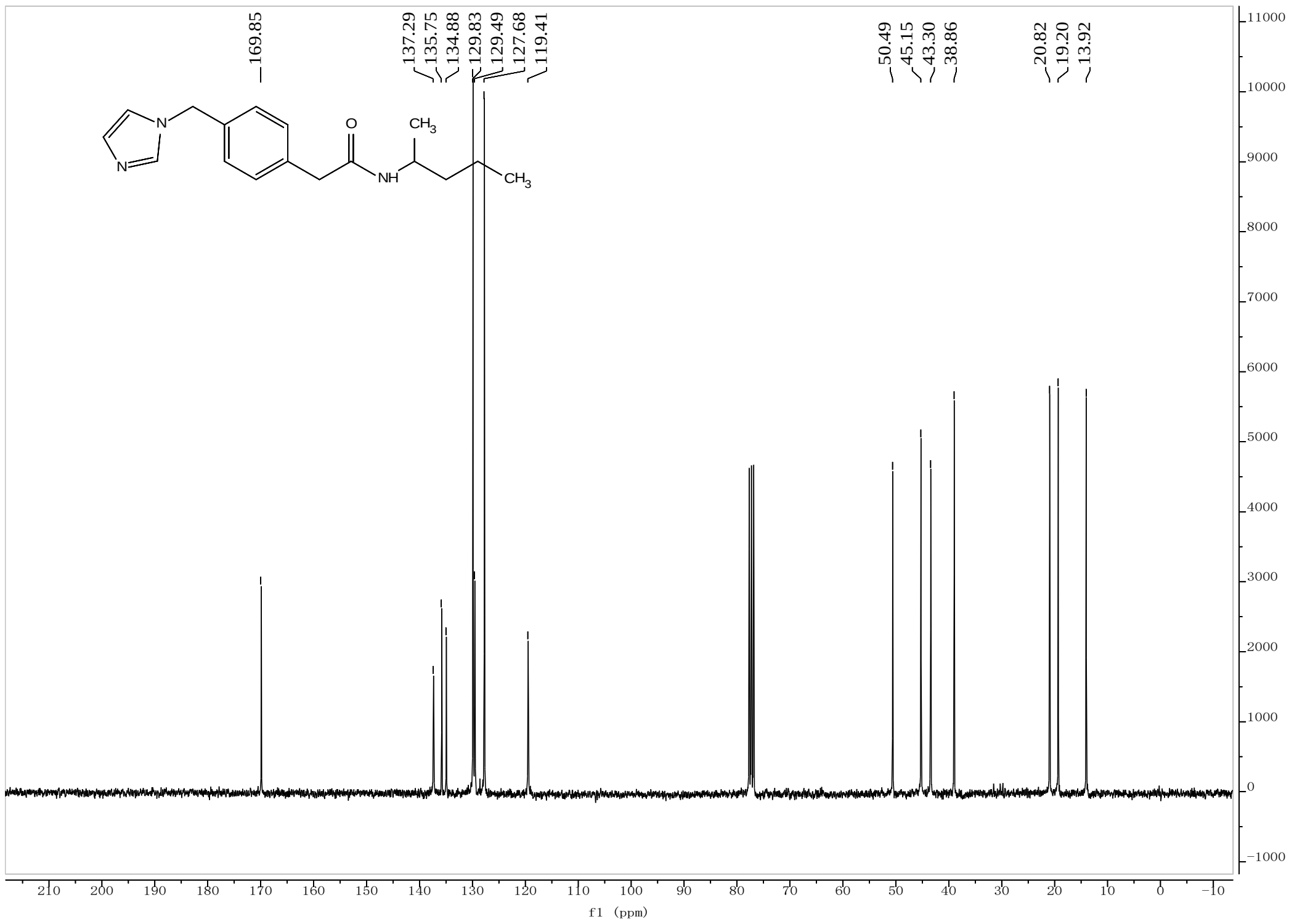

^{13}C NMR (75 MHz, Chloroform-*d*) δ 169.85, 137.29, 135.75, 134.88, 129.83, 129.49, 127.68, 119.41, 50.49, 45.15, 43.30, 38.86, 20.82, 19.20, 13.92.

BM-52

^{1}H NMR

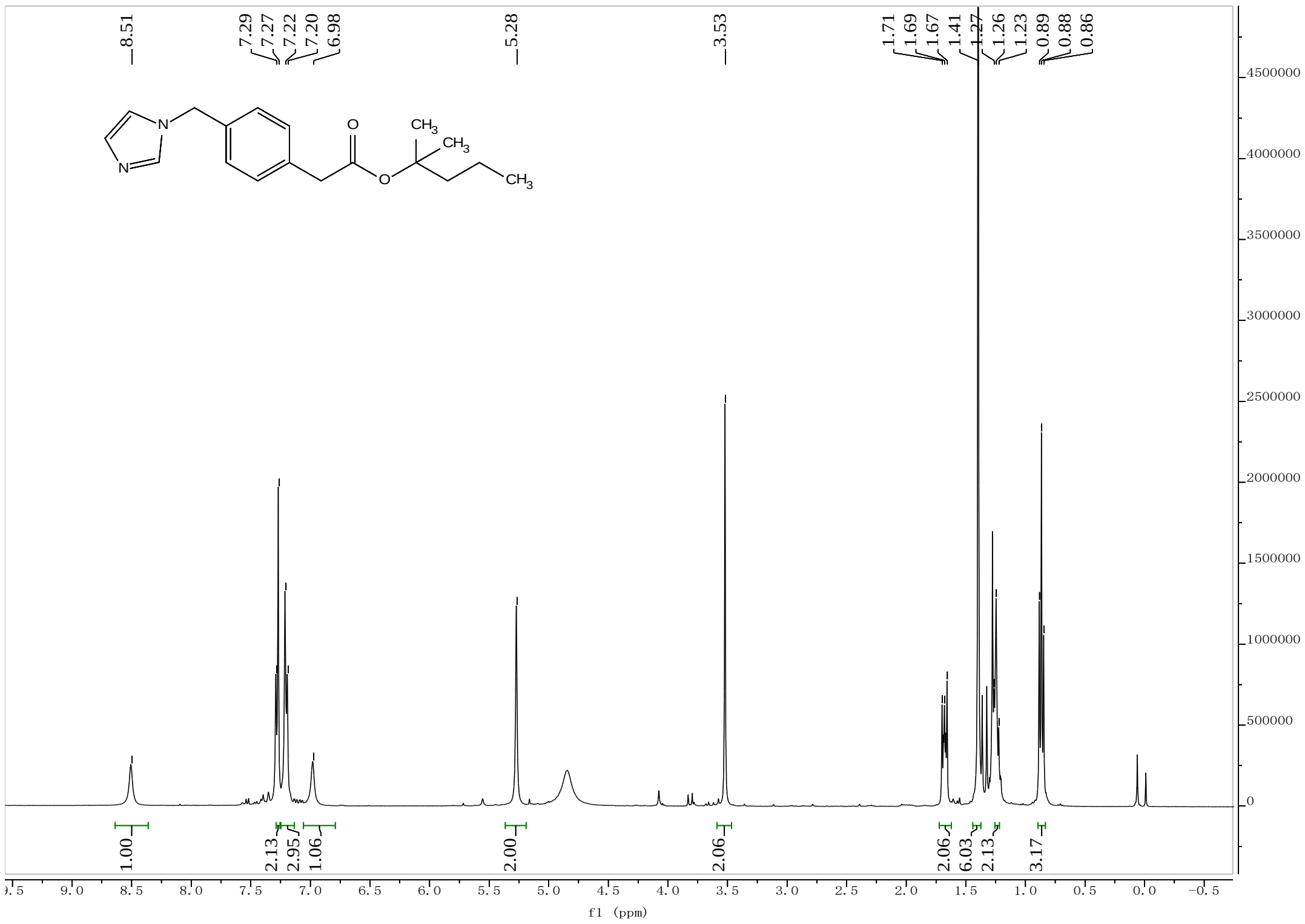

1H NMR (400 MHz, Chloroform-*d*) δ 8.51 (s, 1H), 7.28 (d, *J* = 8.1 Hz, 2H), 7.21 (d, *J* = 7.8 Hz, 3H), 6.98 (s, 1H), 5.28 (s, 2H), 3.53 (s, 2H), 1.73-1.63 (m, 2H), 1.41 (s, 6H), 1.24 (d, *J* = 9.0 Hz, 2H), 0.88 (t, *J* = 7.3 Hz, 3H).

^{13}C NMR

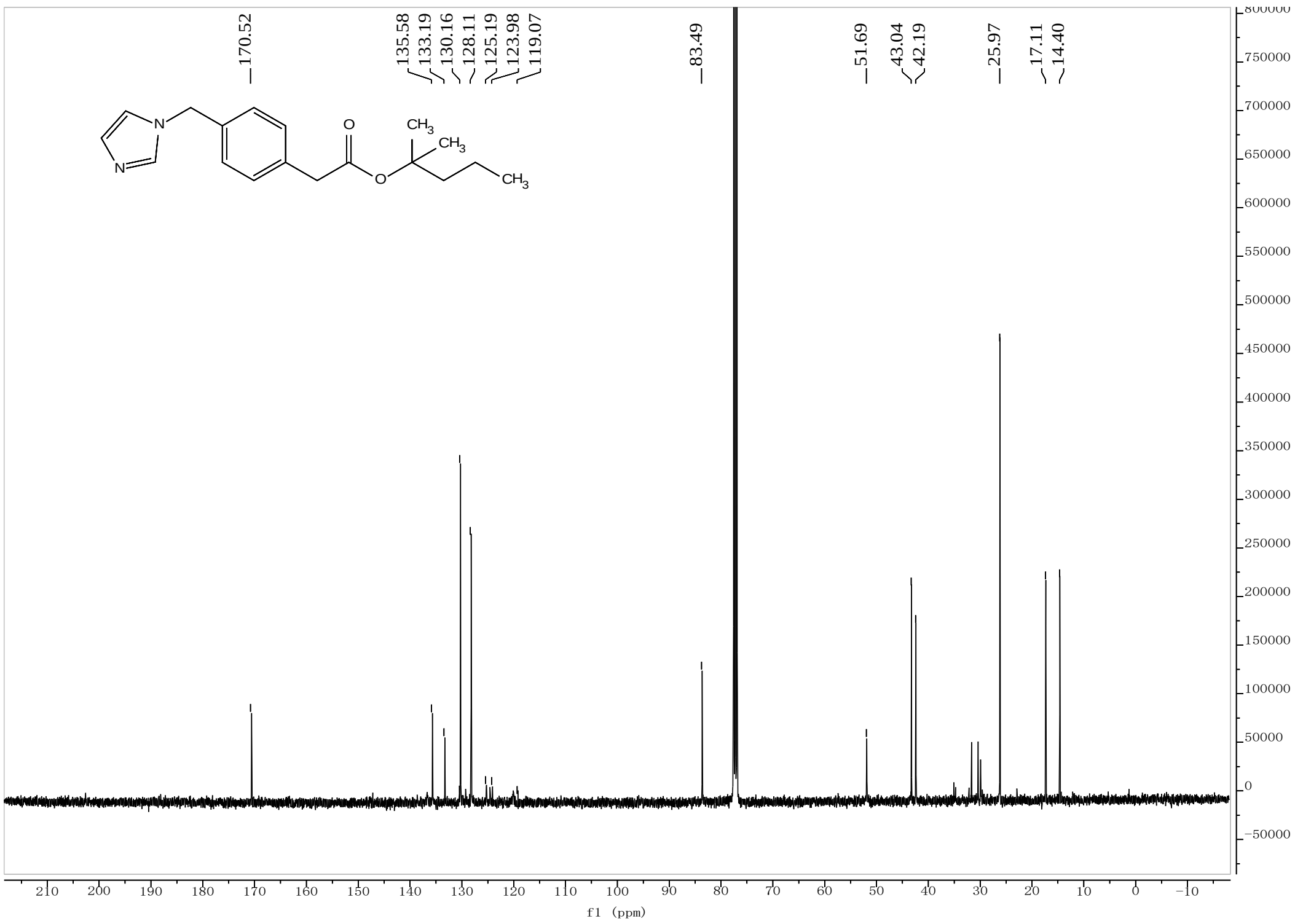

^{13}C NMR (101 MHz, Chloroform-*d*) *δ* 170.52, 135.58, 133.19, 130.16, 128.11, 83.49, 51.69, 43.04, 42.19, 25.97, 17.11, 14.40.

BM-53

^{1}H NMR

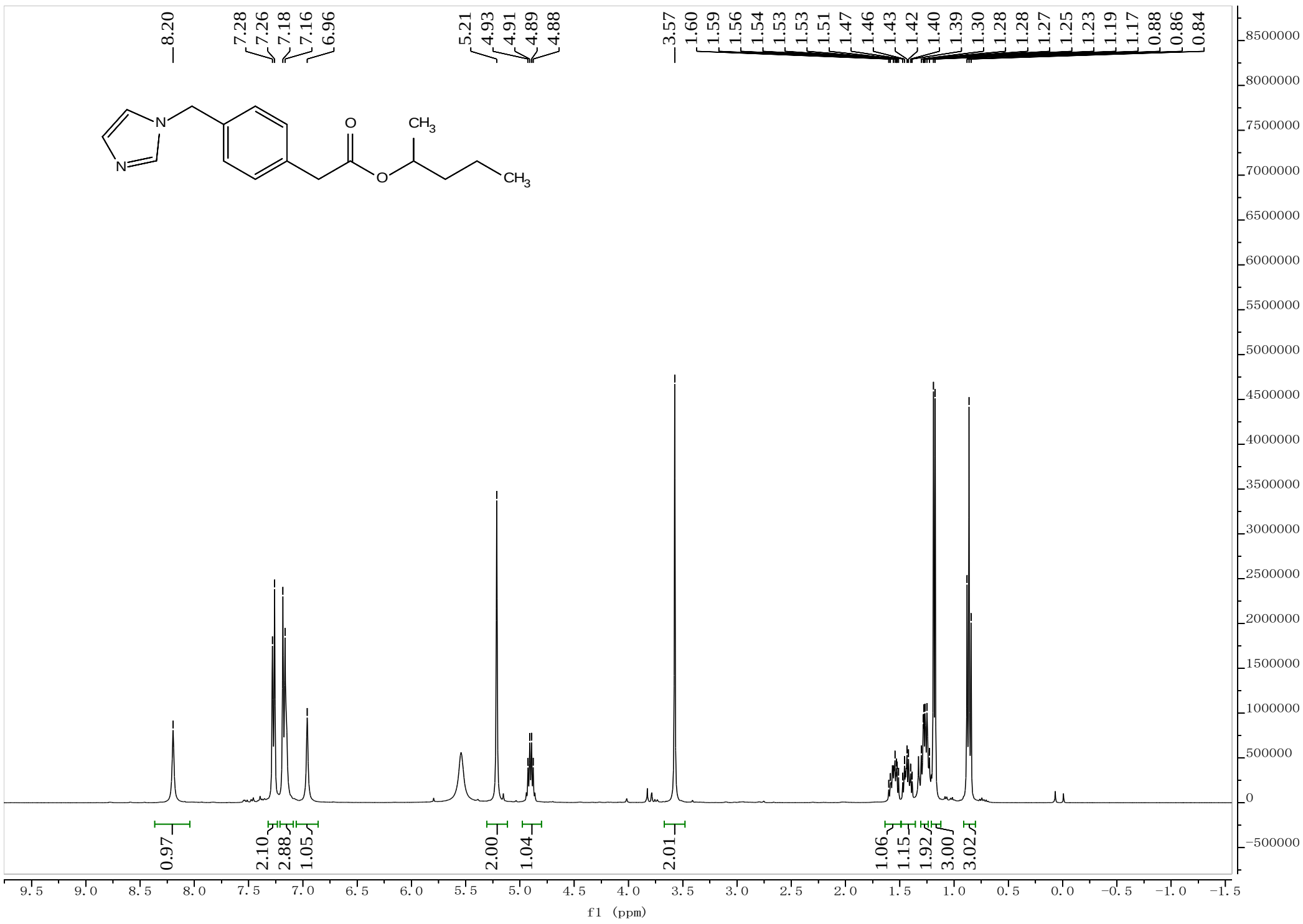

^{1}H NMR (400 MHz, Chloroform-*d*) *δ* 8.20 (s, 1H), 7.27 (d, *J* = 8.0 Hz, 2H), 7.17 (d, *J* = 8.0 Hz, 3H), 6.96 (s, 1H), 5.21 (s, 2H), 4.90 (q, *J* = 6.3 Hz, 1H), 3.57 (s, 2H), 1.64-1.49 (m, 1H), 1.43 (td, *J* = 14.4, 13.6, 6.0 Hz, 1H), 1.31-1.24 (m, 2H), 1.18 (d, *J* = 6.3 Hz, 3H), 0.86 (t, *J* = 7.3 Hz, 3H).

^{13}C NMR

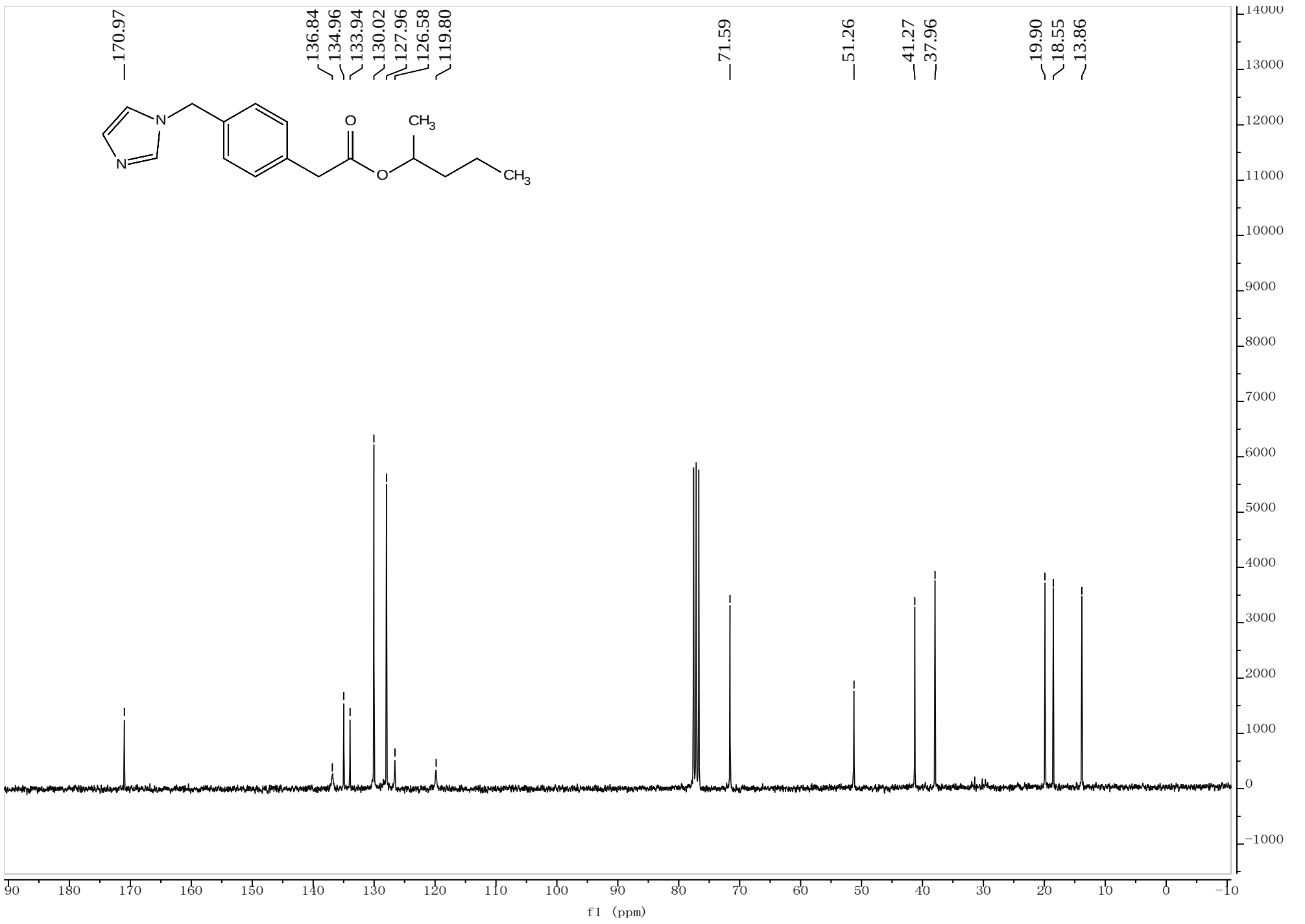

^{13}C NMR (75 MHz, Chloroform-*d*) *δ* 170.97, 136.84, 134.96, 133.94, 130.02, 127.96, 126.58, 119.80, 71.59, 51.26, 41.27, 37.96, 19.90, 18.55, 13.86.
